# Carboxylic Acid Deoxyfluorination and One-Pot Amide Bond Formation Using Pentafluoropyridine (PFP)

**DOI:** 10.1021/acs.orglett.1c01953

**Published:** 2021-07-12

**Authors:** William D. G. Brittain, Steven L. Cobb

**Affiliations:** Department of Chemistry, Durham University, South Road, Durham DH1 3LE, United Kingdom

## Abstract

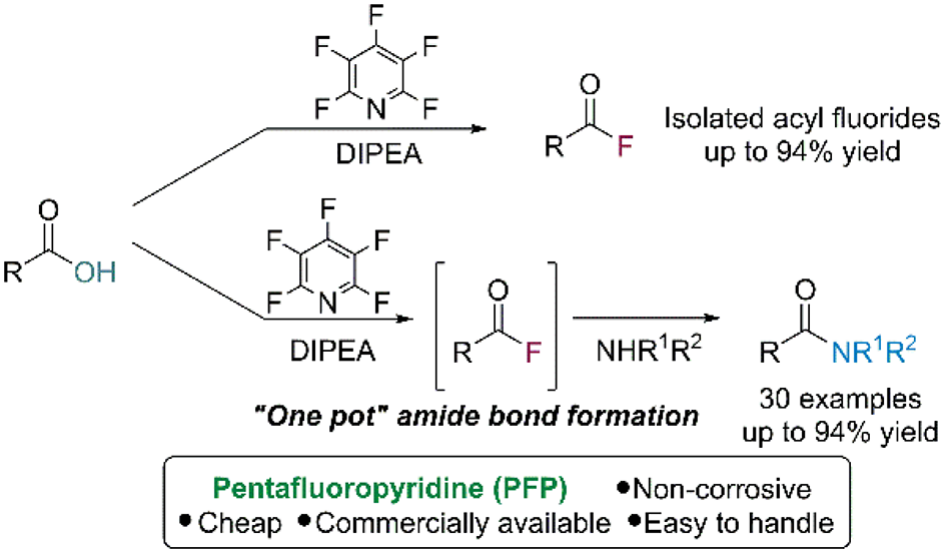

This work describes the application of pentafluoropyridine (PFP), a cheap commercially available reagent, in the deoxyfluorination of carboxylic acids to acyl fluorides. The acyl fluorides can be formed from a range of acids under mild conditions. We also demonstrate that PFP can be utilized in a one-pot amide bond formation via *in situ* generation of acyl fluorides. This one-pot deoxyfluorination amide bond-forming reaction gives ready access to amides in yields of ≤94%.

Acyl fluorides have emerged as a highly valuable class of molecules in the field of synthetic organic chemistry, and they can be applied in a wide variety of useful transformations.^1^ Acyl fluorides have been used as key reagents in challenging amidations/esterifications and coupling reactions,^2^ as a source of anhydrous fluoride ions,^3^ and more recently in nickel-catalyzed decarbonylative borylations.^4^ Despite the clear interest within the synthetic community to utilize acyl fluorides, access to this class of molecule may require the use of toxic reagents, harsh reaction conditions, or the application of approaches that have limited substrate tolerance in some cases.^1a^

The synthesis of acyl fluorides^5^ was pioneered by Olah with his use of cyanuric fluoride and SeF_4_·pyridine complexes.^6^ Ishikawa and Petrov followed this with the development of their related α-fluoroamine reagents (Scheme 1a).^7^ Issues associated with preparation and toxicity led to the development of new sulfur-based deoxyfluorination alternatives (Scheme 1a), such as DAST,^8^ XtalFluor-E,^2a,9^ and more recently (Me_4_N)SCF_3_,^10^ SO_2_F_2_,^11^ and others.^12^ However, as with the α-fluoroamines, these reagents can require bespoke synthesis, have narrow substrate tolerance, or are toxic and/or corrosive. More recently, Prakash disclosed the synthesis of acyl fluorides using triphenylphosphine, NBS, and Et_3_N·3HF.^13^ This approach used readily available commercial reagents; however, the fluoride source (HF) is toxic and corrosive. Other notable advances in the area include the work of Hu (Scheme 1b, CpFluor)^14^ and Shibata, who recently disclosed the synthesis of acyl fluorides from carboxylic acids, aldehydes, and alcohols through oxidative fluorination using trichloroisocyanuric acid (TCCA).^15^ Despite these advances in the generation of acyl fluorides, challenges remain, mostly revolving around the corrosive nature of the reagents needed and their incompatibility with other desirable one-pot processes such as amide or ester synthesis.

**Scheme 1:**
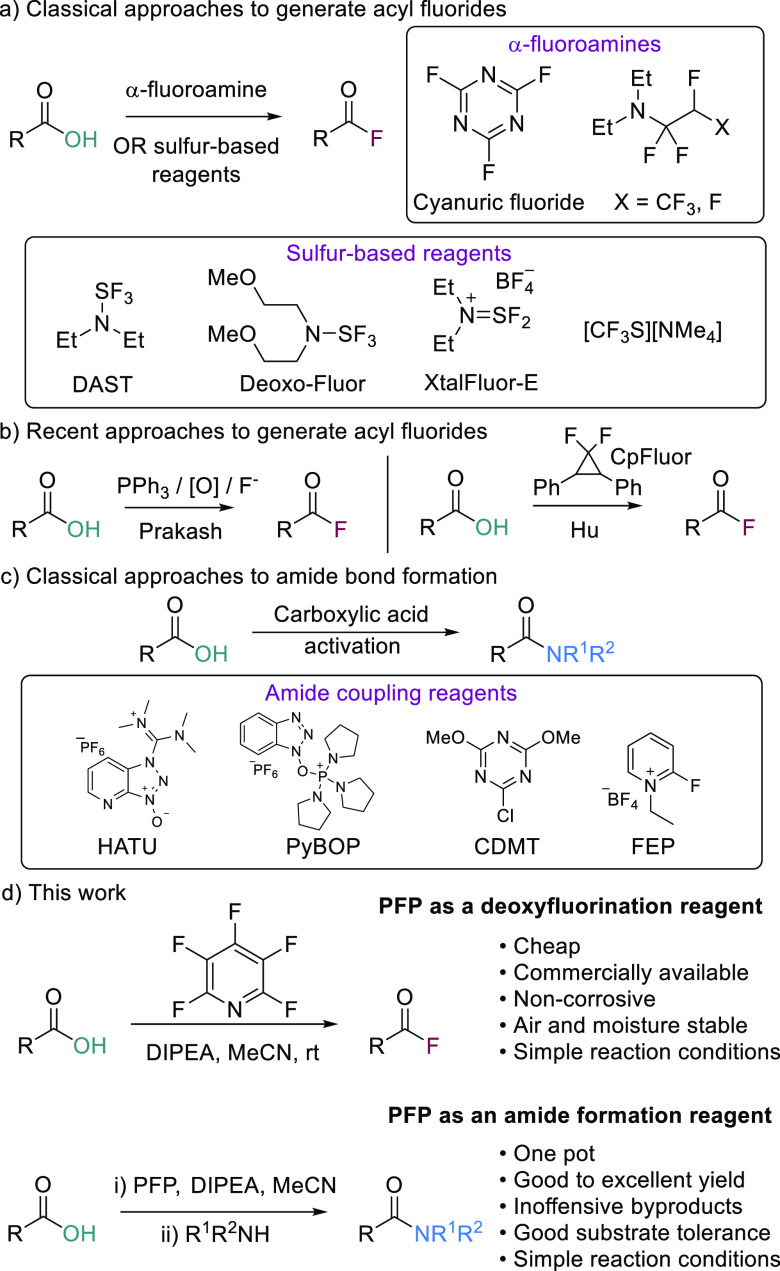
Acyl Fluoride Synthesis and Amide Bond Formation

As part of an ongoing program of work to investigate the synthetic applications of pentafluoropyridine (PFP) **2**, we hypothesized that this reagent might be capable of delivering acyl fluorides under mild reaction conditions. In this regard, it is worth noting that Crimmin has successfully generated acyl fluorides in the reaction of acetic anhydrides with PFP and DMAP.^16^ However, in this reaction sequence, addition of DMAP is required and acyl fluoride generation was not the primary focus of the work. PFP (**2**) also shares some structural similarities with other deoxyfluorination reagents, for example, cyuranic fluoride.^17^ Both PFP (**2**) and cyanuric fluoride possess aromatic fluorines that are highly susceptible to displacement via S_N_Ar reactions. Previously, the ability to undergo S_N_Ar reactions has led to applications for PFP (**2**) in protecting group chemistry,^18^ peptide modification,^19^ unsymmetrical biaryl synthesis,^20^ polymer chemistry,^21^ and macrocycle synthesis.^22^ We speculated that PFP (**2**) could be reactive enough to generate acyl fluorides directly from carboxylic acids through an S_N_Ar, deoxyfluorination sequence. In such a sequence, the PFP reagent would be acting in a dual role, providing a way to initially activate the carboxylic acid toward nucleophilic attack and acting as a fluoride source to generate the desired acyl fluorides.

On the basis of our previous studies with PFP (**2**),^19b^ we also assumed that the most likely byproduct from the proposed acyl fluoride preparation would be tetrafluorohydroxypyridine (**5**). This compound has, in our experience, been shown to be largely unreactive (at room temperature) to a wide variety of other reagents. This is due to the *p*-hydoxyl moiety on the pyridine ring deactivating the system toward additional S_N_Ar reactions. This led us to consider if PFP (**2**) could also be utilized as a one-pot amide bond-forming reagent via an *in situ* acyl fluoride generation type mechanism.^2a,2c,2e,23^ In this capacity, PFP (**2**) would offer enhanced atom economy over coupling reagents such as HATU and PyBOP (Scheme 1c) and offer an alternative to pyridine-based agents such as 2-chloro-4,6-dimethoxy-1,3,5-triazine (CDMT) or 2-fluoro-1-ethylpyridinium tetrafluoroborate (FEP) (Scheme 1c).^24^ Herein, we report the use of PFP (**2**) for the generation of acyl fluorides from carboxylic acids and in the one-pot preparation of amides via an *in situ* acyl fluoride generation (Scheme 1d).

To initially evaluate PFP as a deoxyfluorination reagent, we took a 1:1:1 mixture of benzoic acid (**1a**), PFP (**2**), and DIPEA and stirred it at room temperature for 16 h in dry MeCN. Using ^19^F NMR analysis (see pages S-123 and S-124 of the Supporting Information) of the crude reaction mixtures, we were able to determine that acyl fluoride **3** had been successfully generated (18.1 ppm). We were then able to use these reaction conditions to prepare acyl fluorides **3a–3h** in yields that were comparable to those obtained using previously reported methods (Scheme 2).^13^ We were also able to apply the PFP methodology to access biologically relevant substrates such as ibuprofen (**3g**) and naproxen (**3h**), giving the acyl fluoride analogues in 93% and 94% yields, respectively. These findings confirmed our initial hypothesis that PFP (**2**) could be used as a mild deoxyfluorination reagent to generate acyl fluorides. Following the successful generation of **3a–3h**, we turned our attention toward investigating the application of PFP (**2**) for the *in situ* generation of acyl fluorides in amide bond formations.

**Scheme 2:**
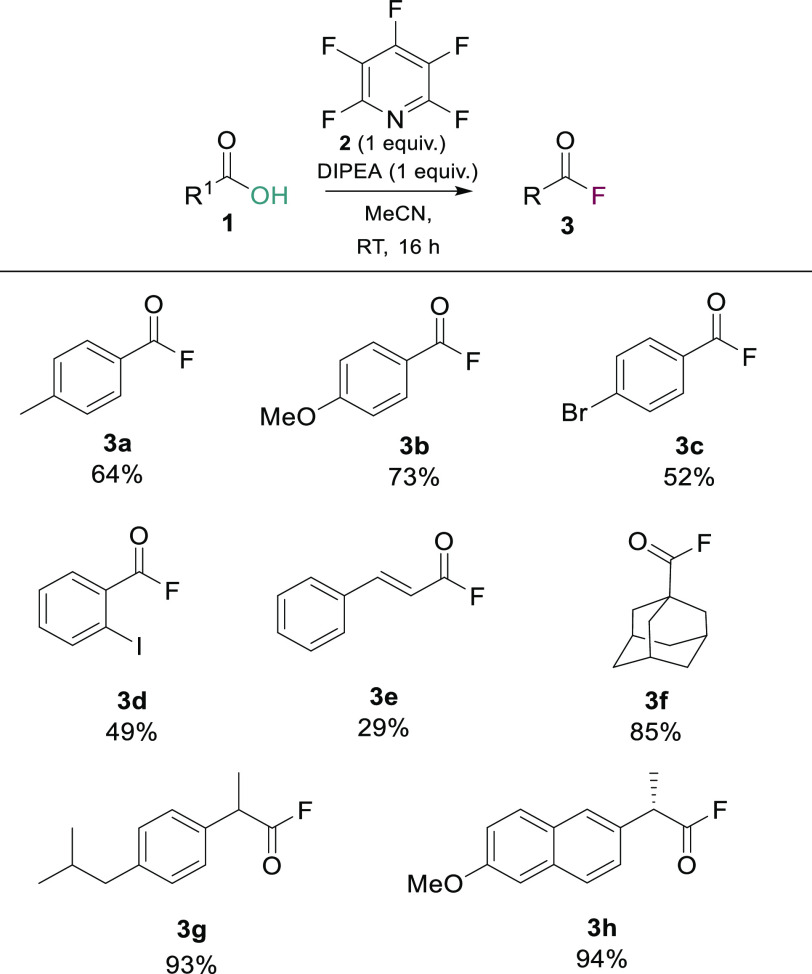
Synthesis of Acyl Fluorides from Carboxylic Acids

To start our optimization of the proposed one-pot deoxyfluorination, amide bond-forming reaction, we picked a benchmark reaction of benzoic acid (**1a**) and benzylamine. With our first set of reaction conditions (PFP **2** and DIPEA) (Table 1, entry 1), all reagents were added simultaneously, including the benzylamine; this reaction led to a 32% yield (isolated) of the desired amide **4a**. The low yield was attributed to the formation of a byproduct that had arisen due to the unwanted S_N_Ar reaction that had occurred between PFP (**2**) and benzylamine; a similar byproduct had been observed previously in the crude LCMS of a test reaction using aniline as the amine component (see page S-127 of the Supporting Information). To minimize this side reaction, an activation period of 30 min was included to allow the generation of the acyl fluoride prior to amine addition (Table 1, entry 2). In addition, the numbers of equivalents of PFP (**2**) and base were decreased to help minimize byproduct formation. This led to a greatly increased yield of 94% of the desired amide product **4a**. Decreasing the amount of based used, i.e., to 1.1 equiv, had a deleterious effect on the product yield (Table 1, entry 3), and when no base was included, no reaction was observed (Table 1, entry 4). Changing the identity of the base (Table 1, entries 5–7) or the solvent (Table 1, entries 9–12) was found not to improve the observed amide yield. This led us to taking the conditions from entry 2 of Table 1 forward for further exploitation and substrate scope evaluation.

**Table 1:**
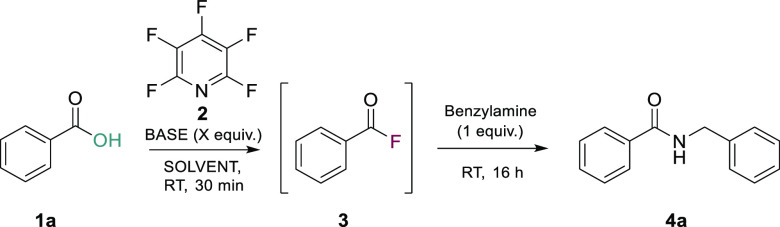
Optimization of a Tandem Deoxyfluorination Amidation Sequence

entry	PFP (equiv)	base (equiv)	solvent	yield (%)^a^
1^b^	3.0	DIPEA (3)	MeCN	32
2	1.1	DIPEA (2)	MeCN	94
3	1.1	DIPEA (1)	MeCN	44
4	1.1	none	MeCN	–
5	1.1	TEA (2)	MeCN	14
6	1.1	K_2_CO_3_ (2)	MeCN	4
7	1.1	pyridine (2)	MeCN	7
8	1.1	DIPEA (2)	DCM	16
9	1.1	DIPEA (2)	1,4-dioxane	–
10	1.1	DIPEA (2)	THF	–
11	1.1	DIPEA (2)	DMF	2
12	1.1	DIPEA (2)	NMP	–

a Isolated yield following column chromatography.

b No activation period; i.e., amine was added at the same time as PFP.

We then probed the mechanism of amide bond formation to confirm that *in situ* acyl fluoride generation was occurring. To do this, we employed both LCMS and ^19^F NMR techniques to probe the makeup of the species present within the reaction mixture at various times. After obtaining a ^19^F NMR spectrum of the activated mixture, we performed spiking experiments with reference compounds, including isolated benzoyl fluoride (**3**) and 2,3,5,6-tetrafluoro-4-hydroxypyridine (**5**) (see pages S-123 and S-124 of the Supporting Information). From this, we were able to unambiguously confirm the presence of compounds **3** and **5** after the initial activation period (30 min) (Scheme 3a). The ^19^F NMR observations were confirmed by LCMS analysis of a crude reaction mixture (see pages S-125–S-127 of the Supporting Information). From the analysis, we were able to propose a mechanism for the one-pot deoxyfluorination amide bond-forming reaction (Scheme 3b). This mechanism also considers the need for a minimum of 2.0 equiv of base that was seen in the optimization experiments (Table 1).

**Scheme 3:**
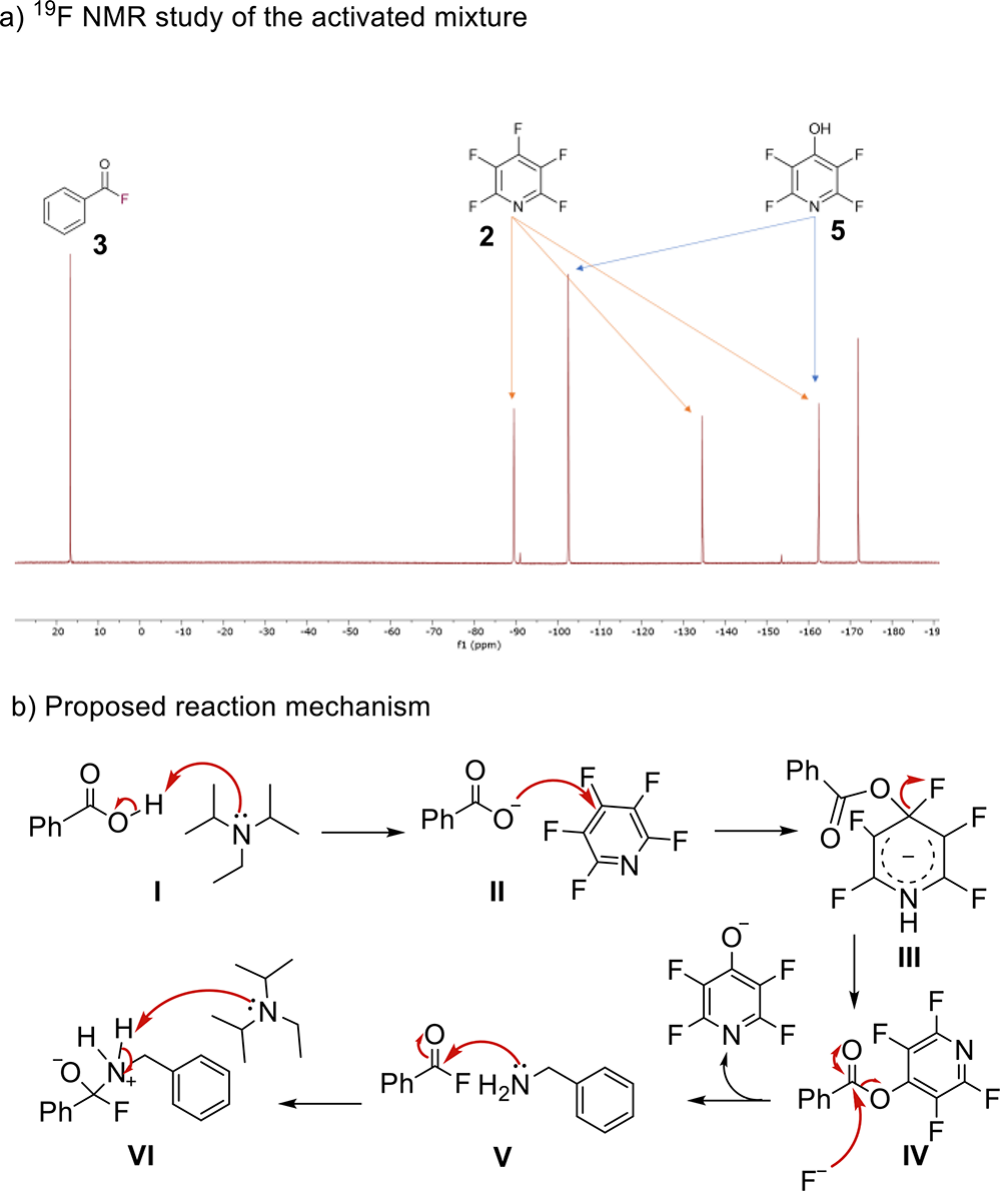
(a) ^19^F NMR Study of Acyl Fluoride Formation and (b) Proposed Mechanism for the Deoxyfluorination Amidation One-Pot Reaction

Following confirmation that PFP (**2**) could readily generate acyl fluorides *in situ* for amidation reactions, we explored the substrate scope of the process. A range of aliphatic and aromatic carboxylic acids and amines were employed using the developed conditions (Scheme 4).

**Scheme 4:**
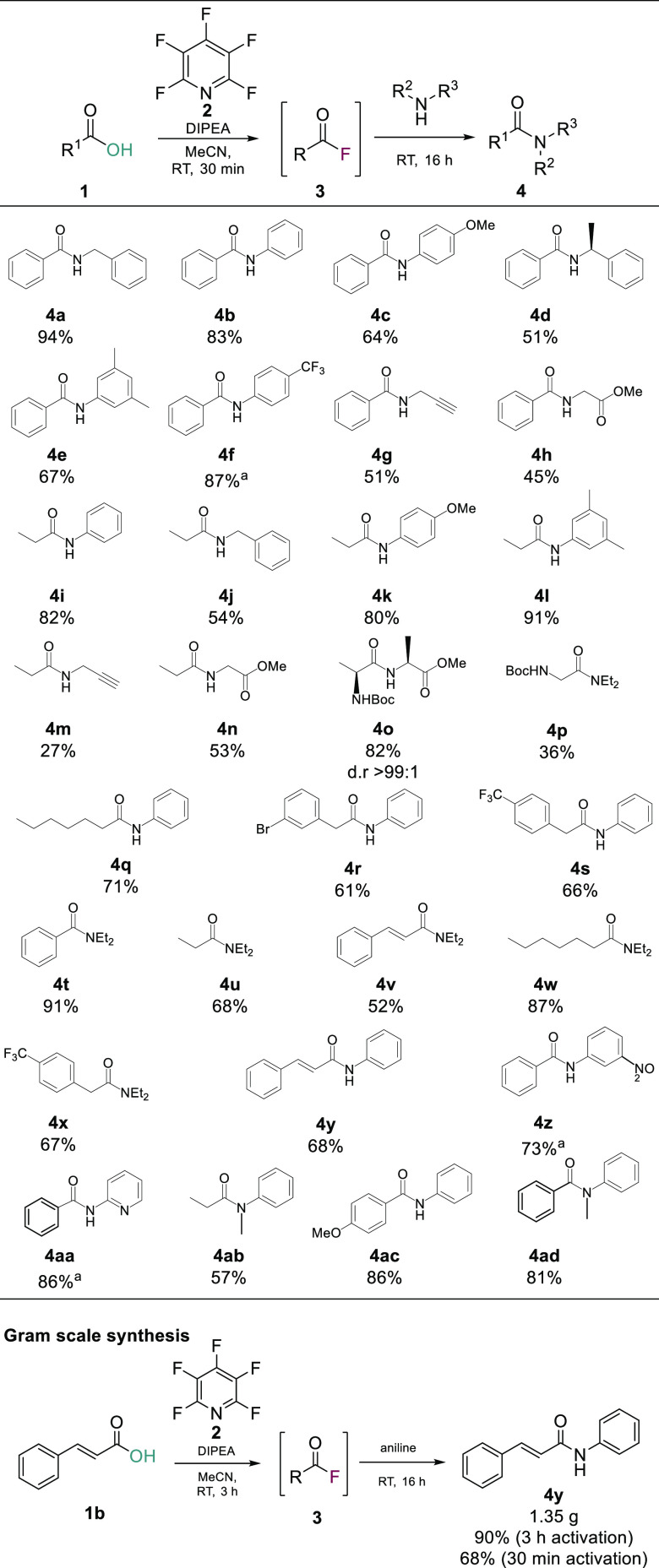
Scope of One-Pot Deoxyfluorination Amidation After the activation period, the reaction mixture was heated to 100 °C in a sealed tube for 16 h.

It was found that amides **4a–4ad** were isolated in good to excellent yields with electron rich amines (e.g., 4-methoxyaniline and 3,5-dimethylaniline) in general giving the best yields at room temperature. The methodology was also found to be applicable for the preparation of both secondary and tertiary amides with both aliphatic and aromatic amines.

Electron deficient anilines and aminopyridines, which are less nucleophilic entities, did require heating in a sealed tube following acyl fluoride formation to generate the target amides. Under these reaction conditions, 4-(trifluoromethyl)aniline (**4f**), 2-aminopyridine (**4aa**), and 3-nitroaniline (**4z**) gave the corresponding amines in 87%, 86%, and 73% yields, respectively. The use of 4-nitroaniline was also attempted; however, this gave very poor conversion (>5%) even under sealed tube conditions. ^19^F NMR monitoring of the reaction with 4-nitroaniline showed that the intermediate acyl fluoride was still present in the reaction mixture even after heating (see pages S-128 and S-129 of the Supporting Information). This confirmed that as expected the inherent lack of nucleophilicity of the amine was the reason for the poor observed reaction conversion. This result mirrors previous observations in this area that showed that increased temperatures are required for electron poor or highly sterically hindered amine substrates.^2c^

From the differences seen in the amide yields obtained among the various acid substrates used, we also hypothesized that the activation time for acyl fluoride formation may also be important. To this end, we selected a representative example from the substrate scope study and increased the activation time from 30 min to 3 h. In addition, to show the applicability of the methodology to gram scale synthesis we also increased the scale of the reaction. We chose to repeat the synthesis of **4y** using 1 g of *trans*-cinnamic acid. Increasing the initial activation period to 3 h was found, in this specific case, to increase the yield, and **4y** was isolated in 90% yield (Scheme 4). However, it should be noted that increasing the activation period for all substrates may not increase the amide yield as there is a balance to be struck between acyl fluoride formation and acyl fluoride degradation. Therefore, it is suggested that a 30 min activation should be tried in the first instance before increasing the activation window.

In addition to amide bond formation, we were interested in seeing if this *insitu* acyl fluoride generation method was applicable to other nucleophilic addition/elimination processes such as ester formation. In a small scale proof of principle study, we were able to generate esters from both electron poor and electron rich phenols with benzoic acid such as **6a** (68%) and **6b** (50%) (Scheme 5). We were also able to demonstrate the synthesis of esters from aliphatic acids to generate compounds **6c** and **6d** in 23% and 24% yields, respectively. It should be noted that the reaction conditions used were directly transferred from the amide bond formation protocols, and thus, further optimization for ester formation is required. In addition to optimizing the reaction conditions for accessing esters, we are currently studying other addition/elimination processes and will look to report the outcomes from this work in due course.

**Scheme 5:**
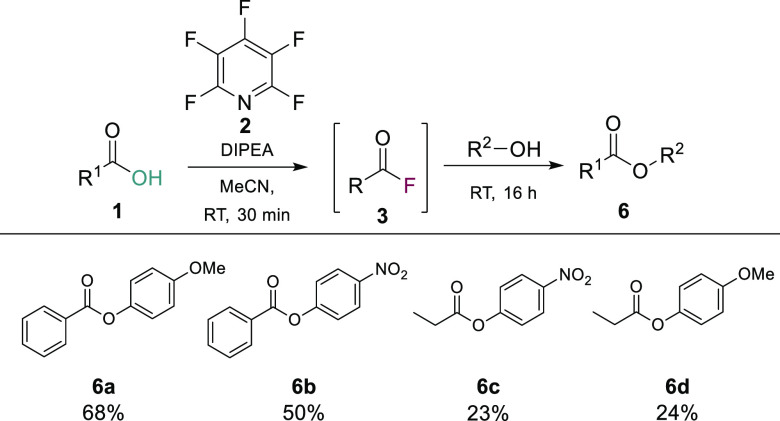
PFP-Enabled One-Pot Synthesis of Esters

In conclusion, PFP (**2**) has been shown to function as a deoxyfluorination reagent allowing the generation of acyl fluorides from a range of carboxylic acids under mild reaction conditions. Given that PFP is cheap, commercially available, non-corrosive, and bench stable, we see it as a useful alternative to other reagents currently used in the field. In addition, we have demonstrated that PFP can be utilized in a one-pot amide bond formation process via the *in situ* formation of acyl fluorides. This reaction between unactivated carboxylic acids and amines gives ready access to amides in good to excellent yields. The application of the methodology to ester formation is reported, but further optimization is required.

## Supporting Information Available

Experimental procedures and NMR spectra (PDF)
