# Supplementary material for: Carboxylic Acid Deoxyfluorination and One-Pot Amide Bond Formation Using Pentafluoropyridine (PFP)
Source: Org Lett. 2021 Jul 12;23(15):5793–8. doi: 10.1021/acs.orglett.1c01953 (PMC8397423; doi:10.1021/acs.orglett.1c01953)
Supplement: Supplementary file 1 — ol1c01953_si_001.pdf [file ol1c01953_si_001.pdf]

Supporting Information

Carboxylic Acid Deoxyfluorination and One-pot Amide Bond Formation using Pentafluoropyridine (PFP)

William D. G. Brittain*[a] and Steven L. Cobb*[a]

[a]*Department of Chemistry, Durham University, South Road, Durham DH1 3LE, United Kingdom*

Email: william.d.brittain@durham.ac.uk, s.l.cobb@durham.ac.uk

Contents

General 1

General Method for the Synthesis of Acyl Fluorides 2

General Method for the Synthesis of Amides and Esters *via in situ* Acyl Fluoride Generation 7

NMR Data for Synthesised Compounds 29

NMR Study of Acyl Fluoride Formation 123

LCMS of Crude Reaction Mixture 125

4-Nitroaniline Sealed Tube Reaction Crude NMRs 128

References 130

General

All starting materials and reagents were bought from commercial sources and used as received. All reagents were purchased from Sigma Aldrich or Fluorochem and used as received. All reactions apart from where noted were carried out in air. MeCN was dried over activated 4 Å molecular sieves which had been dried under vacuum at 150 °C for 3 h. All flash column chromatography was carried out using silica purchased from Fluorochem using the solvent system noted. ^{1}H NMR spectra were recorded at 400 MHz using a Bruker Avance III spectrometer. ^{13}C NMR spectra were recorded at 101 MHz using a Bruker Avance III spectrometer. ^{19}F NMR spectra were recorded at 376 MHz using a Bruker Avance III spectrometer. All coupling constants are reported in Hertz (Hz). In cases where it was required 2D NMR techniques were used to confirm compound identity. Chemical shifts are reported in ppm and are referenced to residual solvent peaks; $CHCl_3$ (^{1}H 7.26 ppm, ^{13}C 77.0 ppm) and CH_3CN (^{1}H 1.96 ppm,

^{13}C 118.3). Mass spectra were collected using ESI-LC in MeCN using a Waters TQD mass spectrometer with a Acquity UPLC BEH C18 1.7 µm (2.1 mm x 50 mm). ESI-LC was collected using water containing formic acid (0.1% v/v) and MeCN mixture in a 95:5 to 5:95 gradient over 5 min.

General Method for the Synthesis of Acyl Fluorides

To a solution of carboxylic acid (1 equiv.) in MeCN was added pentafluoropyridine (1 equiv.) and DIPEA (1 equiv.) and the reaction mixture stirred at room temperature for 16 h. Following this time the reaction mixture was concentrated under reduced pressure and the residue purified directly via either flash column chromatography or by passing through a silica plug (3 cm wide by 3 cm deep). The solution was then concentrated under reduced pressure to yield the desired acyl fluorides.

NOTE: Acyl fluorides are prone to hydrolysis over time thus we suggest that purification of the reaction mixtures takes place as quickly as possible following the end of the reaction period. Any hydrolysed materials may be repurified by passing through a second silica plug. Also, some acyl fluorides are known to be unstable on silica and thus we suggest using the in-situ generation presented below if there are stability problems with substrates.

4-methylbenzoyl fluoride 2a

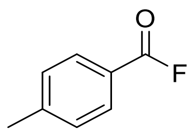

Synthesised according to the general method for the synthesis of acyl fluorides from 4-methylbenzoic acid (0.25 g, 1.84 mmol). The crude material was purified by flash column chromatography (100% hexane to 50% hexane 50% toluene) to give the desired product as a clear oil in 64% yield (0.162 g).

Characterisation data were consistent with the previously reported literature values.[1]

^{1}H NMR (400 MHz, $CDCl_3$) δ 7.96 (d, J = 8.2, 2H), 7.35 (d, J = 8.2 , 2H), 2.48 (s, 4H).

^{19}F NMR (376 MHz, $CDCl_3$) δ 17.44 (s, 1F).

^{13}C NMR (101 MHz, $CDCl_3$) δ 157.6 (d, J = 342.8), 146.6, 131.5 (d, J = 4.0), 129.8 (d, J = 1.3), 122.1 (d, J = 60.9), 21.9.

4-methoxybenzoyl fluoride 2b

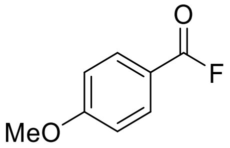

Synthesised according to the general method for the synthesis of acyl fluorides from 4-methoxybenzoic acid (0.25 g, 1.64 mmol). The crude material was purified by flash column chromatography (100% hexane to 90% hexane 10% toluene) to give the desired product as a clear oil in 73% yield (0.185 g).

Characterisation data were consistent with the previously reported literature values.[2]

^{1}H NMR (400 MHz, $CDCl_3$) δ 8.02 (d, J = 8.7, 2H), 7.01 (dd, J = 8.7, 1.2, 2H), 3.92 (s, 3H).
^{19}F NMR (376 MHz, $CDCl_3$) δ 15.95 (s, 1F).
^{13}C NMR (101 MHz, $CDCl_3$) δ 165.2, 157.3 (d, J = 339.9), 133.8 (d, J = 4.2), 116.8 (d, J = 61.6), 114.4 (d, J = 1.4), 55.7.

4-bromobenzoyl fluoride 2c

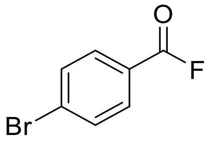

Synthesised according to the general method for the synthesis of acyl fluorides from 4-bromobenzoic acid (0.25 g, 1.24 mmol). The crude material was purified by passing the crude material through a silica

plug (2 cm × 3 cm) and washing with hexane (25 mL) to give the desired product as a white solid in 52% yield (0.128 g).

Characterisation data were consistent with the previously reported literature values.[3]

^{1}H NMR (400 MHz, $CDCl_3$) δ 7.93 (d, *J* = 8.5, 2H), 7.71 (dd, *J* = 8.5, 1.2, 3H).

^{19}F NMR (376 MHz, $CDCl_3$) δ 18.44 (s, 1F).

2-iodobenzoyl fluoride 2d

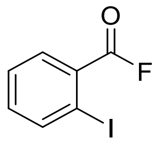

Synthesised according to the general method for the synthesis of acyl fluorides from 2-iodobenzoic acid (0.25 g, 1.04 mmol). The crude material was purified by passing the crude material through a silica plug (2 cm × 3 cm) and washing with hexane (25 mL) to give the desired product as a white solid in 49% yield (0.127 g).

Characterisation data were consistent with the previously reported literature values.[1]

^{1}H NMR (400 MHz, $CDCl_3$) δ 8.16 (dt, *J* = 7.9, 1.2, 1H), 8.06 (dd, *J* = 7.9, 1.7, 1H), 7.53 (td, *J* = 7.7, 1.2, 1H), 7.33 (td, *J* = 7.7, 1.7, 1H).

^{19}F NMR (376 MHz, $CDCl_3$) δ 28.63 (s, 1F).

^{13}C NMR (101 MHz, $CDCl_3$) δ 155.3 (d, *J* = 345.0), 142.7 (d, *J* = 3.9), 135.2, 133.5 (d, *J* = 2.0), 128.4.

cinnamoyl fluoride 2e

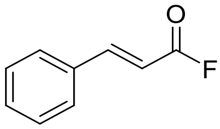

Synthesised according to the general method for the synthesis of acyl fluorides from *trans*-cinnamic acid (0.25 g, 1.68 mmol). The crude material was purified by passing the crude material through a silica plug (2 cm × 3 cm) and washing with hexane (25 mL) to give the desired product as a low melting point solid in 29% yield (0.074 g).

Characterisation data were consistent with the previously reported literature values.[1]

^{1}H NMR (400 MHz, $CDCl_3$) δ 7.87 (d, *J* = 16.0, 1H), 7.62 – 7.57 (m, 2H), 7.51 – 7.43 (m, 3H), 6.40 (dd, *J* = 16.0, 7.4, 1H).

^{19}F NMR (376 MHz, $CDCl_3$) δ 25.59 (d, *J* = 7.4, 1F).

^{13}C NMR (101 MHz, $CDCl_3$) δ 157.1 (d, *J* = 338.8), 151.5 (d, *J* = 6.3), 133.2, 131.9, 129.2, 128.8, 112.1 (d, *J* = 67.4).

(3r,5r,7r)-adamantane-1-carbonyl fluoride 2f

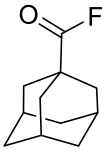

Synthesised according to the general method for the synthesis of acyl fluorides from 1-adamantanecarboxylic acid (0.25 g, 1.39 mmol). The crude material was purified by passing the crude material through a silica plug (2 cm × 3 cm) and washing with hexane (25 mL) to give the desired product as a white solid in 85% yield (0.215 g).

NOTE: A small amount of hydrolysis was observed following purification, leading to minor impurities in obtained spectra.

Characterisation data were consistent with the previously reported literature values.[1]

^{1}H NMR (400 MHz, $CDCl_3$) δ 2.11 – 2.04 (m, 3H), 2.00 – 1.93 (m, 6H), 1.82 – 1.69 (m, 6H).

^{19}F NMR (376 MHz, $CDCl_3$) δ 23.77 (s, 1F).

^{13}C NMR (101 MHz, $CDCl_3$) δ 167.0 (d, J = 371.6), 40.5 (d, J = 44.6), 37.9, 36.1, 27.4.

2-(4-isobutylphenyl)propanoyl fluoride 2g

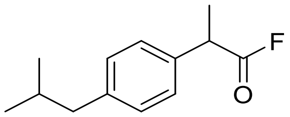

Synthesised according to the general method for the synthesis of acyl fluorides from (±)-2-(4-Isobutylphenyl)propanoic acid (0.25 g, 1.21 mmol). The crude material was purified by passing the crude material through a silica plug (2 cm × 3 cm) and washing with a 1:1 mixture of hexane:DCM (20 mL) to give the desired product as a yellow oil in 93% yield (0.232 g).

Characterisation data were consistent with the previously reported literature values.[3]

^{1}H NMR (400 MHz, $CDCl_3$) δ 7.27 – 7.07 (m, 4H), 3.87 (q, J = 7.1), 2.49 (d, J = 7.2, 2H), 1.97 – 1.78 (m, 1H), 1.61 (dd, J = 7.2, 0.9, 3H), 0.93 (d, J = 6.6, 6H).

^{19}F NMR (376 MHz, $CDCl_3$) δ 39.28 (s, 1F).

^{13}C NMR (101 MHz, $CDCl_3$) δ 164.5 (d, J = 367.1), 134.6, 129.8, 127.2, 45.0, 43.9 (d, J = 49.3), 30.2, 22.4, 18.1 (d, J = 1.4).

(S)-2-(6-methoxynaphthalen-2-yl)propanoyl fluoride 2h

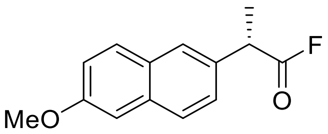

Synthesised according to the general method for the synthesis of acyl fluorides from (*S*)-(+)-2-(6-methoxy-2-naphthyl)propionic acid (0.25 g, 1.08 mmol). The crude material was purified by passing the crude material through a silica plug (2 cm × 3 cm) and washing with a 1:1 mixture of hexane:DCM (20 mL) to give the desired product as a white solid in 94% yield (0.236 g).

Characterisation data were consistent with the previously reported literature values.[3]

^{1}H NMR (400 MHz, $CDCl_3$) δ 7.81 – 7.70 (m, 3H), 7.40 (dd, *J* = 8.5, 1.9, 1H), 7.22 (dd, *J* = 8.9, 2.6, 1H), 7.17 (d, *J* = 2.6, 1H), 4.03 (q, *J* = 7.2, 1H), 3.95 (s, 3H), 1.70 (dd, *J* = 7.2, 0.9, 3H).

^{19}F NMR (376 MHz, $CDCl_3$) δ 39.70 (s, 1F).

^{13}C NMR (101 MHz, $CDCl_3$) δ 164.6 (d, *J* = 367.3), 158.0, 134.1, 132.4, 129.4, 128.9, 127.8, 126.1 (d, *J* = 73.3), 119.5, 105.7, 55.4, 44.2 (d, *J* = 49.3), 18.1.

General Method for the Synthesis of Amides and Esters *via in situ* Acyl Fluoride Generation

To a solution of carboxylic acid (1 equiv.) in MeCN was added pentafluoropyridine (1.1 equiv.) and DIPEA (2 equiv.) and the mixture was stirred for 30 min at rt. Following this the desired amine or alcohol (1 equiv.) was added and the reaction stirred for 16 h at rt. The reaction mixture was then concentrated under reduced pressure and the residue was directly purified using flash column chromatography to yield the respective amides or esters.

N-benzylbenzamide 4a

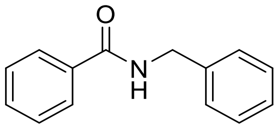

Synthesised according to the general method for the synthesis of amides from benzoic acid (0.100 g, 0.82 mmol) and benzylamine (0.088 g, 0.82 mmol). The crude material was purified by flash column chromatography (100% hexane to 50% hexane 50% ethyl acetate) to give the desired product as a white solid in 94% yield (0.155 g).

Characterisation data were consistent with the previously reported literature values.[4]

^{1}H NMR (400 MHz, $CDCl_3$) δ 7.84 – 7.80 (m, 2H), 7.55 – 7.49 (m, 1H), 7.47 – 7.41 (m, 2H), 7.39 – 7.36 (m, 4H), 7.35 – 7.30 (m, 1H), 6.59 (brs, 1H), 4.66 (d, *J* = 5.7, 2H).

^{13}C NMR (101 MHz, $CDCl_3$) δ 167.5, 138.2, 134.4, 131.6, 128.8, 128.6, 128.0, 127.7, 127.0, 44.2.

LCMS (ESI$^+$) rt = 2.0 min, m/z = 212.1 [M+H]$^+$

N-phenylbenzamide 4b

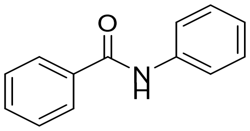

Synthesised according to the general method for the synthesis of amides from benzoic acid (0.100 g, 0.82 mmol) and aniline (0.076 g, 0.82 mmol). The crude material was purified by flash column chromatography (100% hexane to 50% hexane 50% ethyl acetate) to give the desired product as a white solid in 83% yield (0.134 g).

Characterisation data were consistent with the previously reported literature values.[5]

^{1}H NMR (400 MHz, $CDCl_3$) δ 7.94 – 7.83 (m, 3H), 7.71 – 7.64 (m, 2H), 7.61 – 7.56 (m, 1H), 7.54 – 7.49 (m, 2H), 7.44 – 7.36 (m, 2H), 7.22 – 7.15 (m, 1H).

^{13}C NMR (101 MHz, $CDCl_3$) δ 165.7, 137.9, 135.0, 131.9, 129.1, 128.8, 127.0, 124.6, 120.2.

LCMS (ESI^+) rt = 2.1 min, m/z = 198.2 $[M+H]^+$

N-(4-methoxyphenyl)benzamide 4c

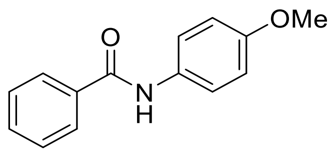

Synthesised according to the general method for the synthesis of amides from benzoic acid (0.100 g, 0.82 mmol) and 4-methoxyaniline (0.101 g, 0.82 mmol). The crude material was purified by flash column chromatography (100% hexane to 50% hexane 50% ethyl acetate) to give the desired product as a colourless crystalline solid in 64% yield (0.118 g).

Characterisation data were consistent with the previously reported literature values.[6]

^{1}H NMR (400 MHz, $CDCl_3$) δ 7.92 – 7.84 (m, 2H), 7.78 (s, 1H), 7.61 – 7.54 (m, 3H), 7.54 – 7.48 (m, 2H), 6.94 (appd, *J* = 9.0, 2H), 3.84 (s, 3H).

^{13}C NMR (101 MHz, $CDCl_3$) δ 156.7, 135.0, 131.8, 131.0, 128.8, 127.0, 122.1, 114.3, 55.5.

LCMS (ESI^+) rt = 2.0 min, m/z = 228.1 $[M+H]^+$

(*S*)-*N*-(1-phenylethyl)benzamide 4d

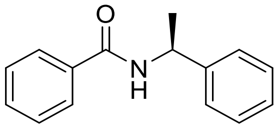

Synthesised according to the general method for the synthesis of amides from benzoic acid (0.100 g, 0.82 mmol) and (*R*)-(+)-α-methylbenzylamine (0.099 g, 0.82 mmol). The crude material was purified

by flash column chromatography (100% hexane to 50% hexane 50% ethyl acetate) to give the desired product as a white solid in 51% yield (0.094 g).

Characterisation data were consistent with the previously reported literature values.[7]

^{1}H NMR (400 MHz, $CDCl_3$) δ 7.86 – 7.76 (m, 2H), 7.54 – 7.47 (m, 1H), 7.45 – 7.34 (m, 6H), 7.34 – 7.26 (m, 1H), 6.59 (appd, *J* = 7.9, 1H), 5.37 (p, *J* = 7.1, 1H), 1.62 (d, *J* = 6.9, 3H).

^{13}C NMR (101 MHz, $CDCl_3$) δ 166.8, 143.2, 134.6, 131.5, 128.8, 128.6, 127.5, 127.0, 126.3, 49.3, 21.8.

LCMS (ESI$^+$) rt = 2.2 min, m/z = 226.1 $[M+H]^+$

N-(3,5-dimethylphenyl)benzamide 4e

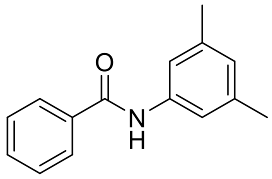

Synthesised according to the general method for the synthesis of amides from benzoic acid (0.100 g, 0.82 mmol) and 3,5-dimethylaniline (0.099 g, 0.82 mmol) The crude material was purified by flash column chromatography (100% hexane to 50% hexane 50% ethyl acetate) to give the desired product as a white solid in 67% yield (0.123 g).

Characterisation data were consistent with the previously reported literature values.[8]

^{1}H NMR (400 MHz, $CDCl_3$) δ 7.98 (brs, 1H), 7.91 – 7.83 (m, 2H), 7.59 – 7.52 (m, 1H), 7.52 – 7.43 (m, 2H), 7.31 (s, 2H), 6.81 (s, 1H), 2.32 (s, 6H).

^{13}C NMR (101 MHz, $CDCl_3$) δ 165.8, 138.8, 137.8, 135.1, 131.7, 128.7, 127.1, 126.3, 118.1, 21.4.

LCMS (ESI$^+$) rt = 2.6 min, m/z = 226.1 $[M+H]^+$

N-(4-(trifluoromethyl)phenyl)benzamide 4f

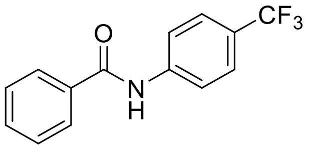

Synthesised according to the general method for the synthesis of amides from benzoic acid (0.100 g, 0.82 mmol) and 4-(trifluoromethyl)aniline (0.132 g, 0.82 mmol) with the following modification, after addition of the amine the reaction mixture was heated to 100 °C in a sealed tube using a hotplate. The crude material was purified by flash column chromatography (100% hexane to 50% hexane 50% ethyl acetate) to give the desired product as a yellow solid in 87% yield (0.190 g).

Characterisation data were consistent with the previously reported literature values.[9]

^{1}H NMR (400 MHz, $CDCl_3$) δ 7.98 (brs, 1H), 7.94 – 7.88 (m, 2H), 7.84 – 7.78 (m, 2H), 7.69 – 7.59 (m, 3H), 7.58 – 7.51 (m, 2H).

^{19}F NMR (376 MHz, $CDCl_3$) δ -62.09 (s, 3F).

LCMS (ESI^+) rt = 2.8 min, m/z = 266.1 $[M+H]^+$

N-(prop-2-yn-1-yl)benzamide 4g

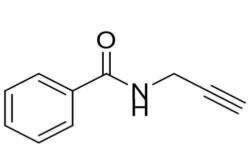

Synthesised according to the general method for the synthesis of amides from benzoic acid (0.100 g, 0.82 mmol) and propargyl amine (0.045 g, 0.82 mmol). The crude material was purified by flash column chromatography (100% hexane to 50% hexane 50% ethyl acetate) to give the desired product as a white solid in 51% yield (0.094 g).

Characterisation data were consistent with the previously reported literature values.

^{1}H NMR (400 MHz, $CDCl_3$) δ 7.85 – 7.77 (m, 2H), 7.56 – 7.50 (m, 1H), 7.49 – 7.40 (m, 2H), 6.56 (brs, 1H), 4.27 (dd, *J* = 5.2, 2.6, 2H), 2.30 (t, *J* = 2.6, 1H).

^{13}C NMR (101 MHz, $CDCl_3$) δ 167.2, 133.7, 131.8, 128.6, 127.1, 79.5, 71.9, 29.8.

LCMS (ESI^+) rt = 1.3 min, m/z = 160.1 $[M+H]^+$

methyl benzoylglycinate 4h

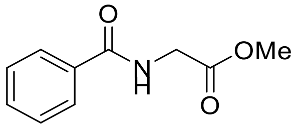

Synthesised according to the general method for the synthesis of amides from benzoic acid (0.100 g, 0.82 mmol) and glycine methyl ester (0.103 g, 0.82 mmol). The crude material was purified by flash column chromatography (100% hexane to 50% hexane 50% ethyl acetate) to give the desired product as a yellow oil in 45% yield (0.072 g).

Characterisation data were consistent with the previously reported literature values.[10]

^{1}H NMR (400 MHz, $CDCl_3$) δ 7.85 – 7.79 (m, 2H), 7.55 – 7.49 (m, 1H), 7.47 – 7.39 (m, 2H), 6.92 (brs, 1H), 4.24 (d, *J* = 5.2, 2H), 3.79 (s, 3H).

^{13}C NMR (101 MHz, $CDCl_3$) δ 170.6, 167.6, 133.6, 131.8, 128.6, 127.1, 52.5, 41.7.

LCMS (ESI^+) rt = 1.3 min, m/z = 294.1 $[M+H]^+$

N-phenylpropionamide 4i

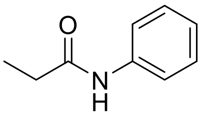

Synthesised according to the general method for the synthesis of amides from propanoic acid (0.100 g, 1.35 mmol) and aniline (0.126 g, 1.35 mmol). The crude material was purified by flash column chromatography (100% hexane to 50% hexane 50% ethyl acetate) to give the desired product as a cream coloured solid in 82% yield (0.164 g).

Characterisation data were consistent with the previously reported literature values.[11]

^{1}H NMR (400 MHz, $CDCl_3$) δ 7.71 (brs, 1H), 7.55 (appd, *J* = 7.9, 2H), 7.31 (appt, *J* = 7.9, 2H), 7.11 (appt, *J* = 7.4, 1H), 2.40 (q, *J* = 7.6, 2H), 1.25 (t, *J* = 7.6, 3H).

^{13}C NMR (101 MHz, $CDCl_3$) δ 172.4, 138.1, 128.9, 124.2, 120.0, 30.7, 9.8.

LCMS (ESI^{+}) rt = 1.5 min, m/z = 150.1 [M+H]$^{+}$

N-benzylpropionamide 4j

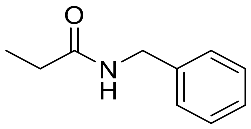

Synthesised according to the general method for the synthesis of amides from propanoic acid (0.100 g, 1.35 mmol) and benzylamine (0.144 g, 1.35 mmol). The crude material was purified by flash column chromatography (100% hexane to 50% hexane 50% ethyl acetate) to give the desired product as a white solid in 54% yield (0.119 g).

Characterisation data were consistent with the previously reported literature values.[12]

^{1}H NMR (400 MHz, $CDCl_3$) δ 7.37 – 7.31 (m, 2H), 7.31 – 7.25 (m, 3H), 6.15 (s, 1H), 4.42 (d, *J* = 5.7, 2H), 2.24 (q, *J* = 7.6, 2H), 1.17 (t, *J* = 7.6, 3H).

^{13}C NMR (101 MHz, $CDCl_3$) δ 173.8, 138.5, 128.7, 127.8, 127.4, 43.5, 29.7, 9.9.

LCMS (ESI^{+}) rt = 1.5 min, m/z = 164.1 [M+H]$^{+}$

N-(4-methoxyphenyl)propionamide 4k

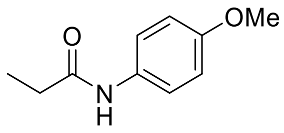

Synthesised according to the general method for the synthesis of amides from propanoic acid (0.100 g, 1.35 mmol) and 4-methoxyaniline (0.166 g, 1.35 mmol). The crude material was purified by flash column chromatography (100% hexane to 50% hexane 50% ethyl acetate) to give the desired product as a grey solid in 80% yield (0.193 g).

Characterisation data were consistent with the previously reported literature values.[13]

^{1}H NMR (400 MHz, $CDCl_3$) δ 7.94 (s, 1H), 7.41 (d, *J* = 9.1, 2H), 6.81 (d, *J* = 9.1, 2H), 3.77 (s, 3H), 2.34 (q, *J* = 7.6, 2H), 1.21 (t, *J* = 7.6, 3H).

^{13}C NMR (101 MHz, $CDCl_3$) δ 172.5, 156.2, 131.3, 122.0, 114.0, 55.5, 30.4, 9.9.

LCMS (ESI$^+$) rt = 1.5 min, m/z = 180.1 $[M+H]^+$

N-(3,5-dimethylphenyl)propionamide 4l

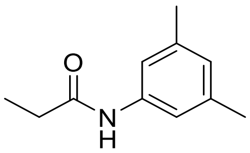

Synthesised according to the general method for the synthesis of amides from propanoic acid (0.100 g, 1.35 mmol) and 3,5-dimethylaniline (0.163 g, 1.35 mmol). The crude material was purified by flash column chromatography (100% hexane to 50% hexane 50% ethyl acetate) to give the desired product as a brown solid in 91% yield (0.217 g).

Characterisation data were consistent with the previously reported literature values.[14]

^{1}H NMR (400 MHz, $CDCl_3$) δ 7.62 (s, 1H), 7.19 (s, 2H), 6.75 (s, 1H), 2.39 (q, *J* = 7.6, 2H), 2.28 (s, 6H), 1.24 (t, *J* = 7.6, 3H).

^{13}C NMR (101 MHz, $CDCl_3$) δ 172.3, 138.6, 137.9, 125.9, 117.7, 30.7, 21.4, 9.8.

LCMS (ESI$^+$) rt = 2.1 min, m/z = 178.1 $[M+H]^+$

N-(prop-2-yn-1-yl)propionamide 4m

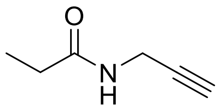

Synthesised according to the general method for the synthesis of amides from propanoic acid (0.100 g, 1.35 mmol) and propargylamine (0.074 g, 1.35 mmol). The crude material was purified by flash column chromatography (100% hexane to 50% hexane 50% ethyl acetate) to give the desired product as a clear oil in 27% yield (0.041 g).

Characterisation data were consistent with the previously reported literature values.[15]

^{1}H NMR (400 MHz, $CDCl_3$) δ 6.08 (s, 1H), 4.05 (dd, *J* = 5.3, 2.6, 2H), 2.29 – 2.19 (m, 3H), 1.16 (t, *J* = 7.6, 3H).

^{13}C NMR (101 MHz, $CDCl_3$) δ 173.6, 79.7, 71.4, 29.4, 29.1, 9.6.

LCMS (ESI$^+$) rt = 0.6 min, m/z = 112.1 $[M+H]^+$

methyl propionylglycinate 4n

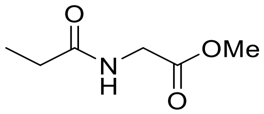

Synthesised according to the general method for the synthesis of amides from propanoic acid (0.100 g, 1.35 mmol) and glycine methyl ester (0.169 g, 1.35 mmol). The crude material was purified by flash column chromatography (100% hexane to 50% hexane 50% ethyl acetate) to give the desired product as a yellow oil in 53% yield (0.103 g).

Characterisation data were consistent with the previously reported literature values.[16]

^{1}H NMR (400 MHz, $CDCl_3$) δ 6.36 (brs, 1H), 4.02 (d, *J* = 5.3, 2H), 3.73 (s, 3H), 2.27 (q, *J* = 7.6, 2H), 1.14 (t, *J* = 7.6, 3H).

^{13}C NMR (101 MHz, $CDCl_3$) δ 174.2, 170.7, 52.3, 41.2, 29.3, 9.6.

LCMS (ESI$^+$) rt = 0.6 min, m/z = 146.1 $[M+H]^+$

methyl (*tert*-butoxycarbonyl)-L-alanyl-L-alaninate 4o

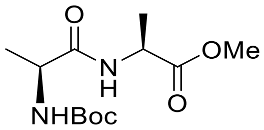

Synthesised according to the general method for the synthesis of amides from Boc-Ala-OH (0.100 g, 0.53 mmol) and alanine methyl ester (0.188 g, 1.35 mmol). The crude material was purified by flash column chromatography (100% hexane to 50% hexane 50% ethyl acetate) to give the desired product as a white solid in 82% yield (0.119 g).

Characterisation data were consistent with the previously reported literature values.[17]

^{1}H NMR (400 MHz, $CDCl_3$) δ 6.79 – 6.61 (brm, 1H), 5.14 – 4.99 (brm, 1H), 4.59 (p, *J* = 7.3, 1H), 4.26 – 4.14 (brm, 1H), 3.76 (s, 3H), 1.46 (s, 9H), 1.42 (d, *J* = 7.1, 3H), 1.38 (d, *J* = 7.1, 3H).

^{13}C NMR (101 MHz, $CDCl_3$) δ 173.2, 172.2, 155.5, 80.2, 52.5, 50.0, 48.0, 28.3, 18.4.

LCMS (ESI^+) rt = 1.6 min, m/z = 275.2 $[M+H]^+$

tert-butyl (2-(diethylamino)-2-oxoethyl)carbamate 4p

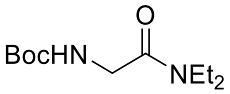

Synthesised according to the general method for the synthesis of amides from Boc-Gly-OH (0.172 g, 0.98 mmol) and diethylamine (0.072 g, 0.98 mmol). The crude material was purified by flash column chromatography (100% hexane to 50% hexane 50% ethyl acetate) to give the desired product as a clear oil in 36% yield (0.081 g).

Characterisation data were consistent with the previously reported literature values.[18]

^{1}H NMR (400 MHz, $CDCl_3$) δ 5.58 (brs, 1H), 3.99 (s, 2H), 3.42 (q, J = 7.2, 2H), 3.28 (q, J = 7.2, 2H), 1.46 (s, 9H), 1.21 (t, J = 7.2, 3H), 1.15 (t, J = 7.2, 3H).

^{13}C NMR (101 MHz, $CDCl_3$) δ 167.6, 156.0, 79.9, 42.1, 41.1, 40.6, 28.3, 14.0, 12.9.

N-phenylheptanamide 4q

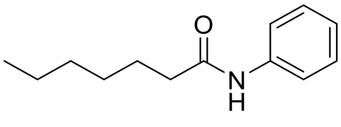

Synthesised according to the general method for the synthesis of amides from heptanoic acid (0.111 g, 0.85 mmol) and aniline (0.079 g, 0.85 mmol). The crude material was purified by flash column chromatography (100% hexane to 50% hexane 50% ethyl acetate) to give the desired product as a brown solid in 71% yield (0.124 g).

Characterisation data were consistent with the previously reported literature values.[19]

^{1}H NMR (400 MHz, $CDCl_3$) δ 7.61 – 7.49 (m, 3H), 7.32 (appt, *J* = 7.9, 2H), 7.11 (appt, *J* = 7.4, 1H), 2.42 – 2.31 (m, 2H), 1.73 (p, *J* = 7.5 Hz, 3H), 1.44 – 1.24 (m, 6H), 0.95 – 0.84 (m, 3H).

^{13}C NMR (101 MHz, $CDCl_3$) δ 171.7, 138.0, 129.0, 124.2, 119.9, 37.8, 31.6, 29.0, 25.7, 22.5, 14.1.

LCMS (ESI$^+$) rt = 2.7 min, m/z = 206.7 $[M+H]^+$

N-phenyl-2-(3-(bromo)phenyl)acetamide 4r

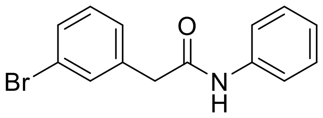

Synthesised according to the general method for the synthesis of amides from 3-bromophenylacetic acid (0.183 g, 0.85 mmol) and aniline (0.079 g, 0.85 mmol). The crude material was purified by flash column chromatography (100% hexane to 50% hexane 50% ethyl acetate) to give the desired product as a white solid in 61% yield (0.151 g).

Characterisation data were consistent with the previously reported literature values.[20]

^{1}H NMR (400 MHz, $CDCl_3$) δ 7.53 – 7.51 (m, 1H), 7.50 – 7.44 (m, 3H), 7.35 – 7.27 (m, 5H), 7.16 – 7.10 (m, 1H), 3.70 (s, 2H).

^{13}C NMR (101 MHz, $CDCl_3$) δ 168.3, 137.5, 136.7, 132.5, 130.8, 130.6, 129.0, 128.1, 124.7, 123.1, 120.0, 44.2.

LCMS (ESI$^+$) rt = 2.6 min, m/z = 290.1 $[M+H]^+$

N-phenyl-2-(4-(trifluoromethyl)phenyl)acetamide 4s

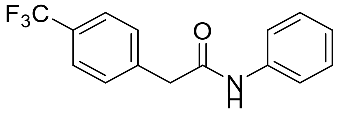

Synthesised according to the general method for the synthesis of amides from 4-(trifluoromethyl)phenylacetic acid (0.173 g, 0.85 mmol) and aniline (0.079 g, 0.85 mmol). The crude material was purified by flash column chromatography (100% hexane to 50% hexane 50% ethyl acetate) to give the desired product as a white solid in 66% yield (0.157 g).

Characterisation data were consistent with the previously reported literature values.[21]

^{1}H NMR (400 MHz, $CDCl_3$) δ 7.68 (appd, *J* = 8.0, 2H), 7.54 – 7.43 (m, 3H), 7.36 – 7.30 (m, 2H), 7.19 – 7.10 (m, 1H), 3.82 (s, 2H).

^{19}F NMR (376 MHz, $CDCl_3$) δ -62.56 (s, 3F).

^{13}C NMR (101 MHz, $CDCl_3$) δ 168.1, 137.8 (d, *J* = 103.5), 129.8, 129.1, 128.9, 126.0 (q, *J* = 3.7), 124.9, 120.0, 44.4.

LCMS (ESI^+) rt = 2.6 min, m/z = 280.2 $[M+H]^+$

***N,N*-diethylbenzamide 4t**

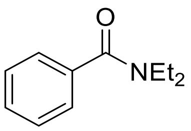

Synthesised according to the general method for the synthesis of amides from benzoic acid (0.120 g, 0.98 mmol) and diethylamine (0.072 g, 0.98 mmol). The crude material was purified by flash column chromatography (100% hexane to 50% hexane 50% ethyl acetate) to give the desired product as a clear oil in 91% yield (0.159 g).

Characterisation data were consistent with the previously reported literature values.[22]

^{1}H NMR (400 MHz, $CDCl_3$) δ 7.41 – 7.33 (m, 5H), 3.55 (q, *J* = 7.2, 2H), 3.24 (q, *J* = 7.2, 2H), 1.25 (t, *J* = 6.5, 3H), 1.10 (t, *J* = 6.5, 3H).

^{13}C NMR (101 MHz, $CDCl_3$) δ 171.4, 137.2, 129.1, 128.4, 126.2, 43.3, 39.3, 14.2, 12.9.

LCMS (ESI^+) rt = 2.1 min, m/z = 178.1 $[M+H]^+$

***N,N*-diethylpropionamide 4u**

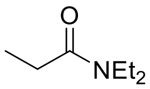

Synthesised according to the general method for the synthesis of amides from propanoic acid (0.073 g, 0.98 mmol) and diethylamine (0.072 g, 0.98 mmol). The crude material was purified by flash column chromatography (100% hexane to 50% hexane 50% ethyl acetate) to give the desired product as a clear oil in 68% yield (0.085 g).

Characterisation data were consistent with the previously reported literature values.[23]

^{1}H NMR (400 MHz, $CDCl_3$) δ 3.37 (q, *J* = 7.1, 2H), 3.30 (q, *J* = 7.1, 2H), 2.33 (q, *J* = 7.5, 2H), 1.19 – 1.08 (m, 9H).

^{13}C NMR (101 MHz, $CDCl_3$) δ 173.2, 41.9, 40.2, 26.2, 14.3, 13.0, 9.6.

LCMS (ESI^+) rt = 1.3 min, m/z = 130.1 $[M+H]^+$

***N,N*-diethylcinnamamide 4v**

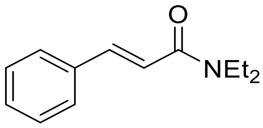

Synthesised according to the general method for the synthesis of amides from *trans*-cinnamic acid (0.145 g, 0.98 mmol) and diethylamine (0.072 g, 0.98 mmol). The crude material was purified by flash

column chromatography (100% hexane to 50% hexane 50% ethyl acetate) to give the desired product as a white solid in 52% yield (0.104 g).

Characterisation data were consistent with the previously reported literature values.[24]

^{1}H NMR (400 MHz, $CDCl_3$) δ 7.72 (d, *J* = 15.4, 1H), 7.54 (dd, *J* = 7.9, 1.7, 2H), 7.42 – 7.34 (m, 3H), 6.84 (d, *J* = 15.4, 1H), 3.55 – 3.45 (m, 4H), 1.27 (t, *J* = 7.1, 3H), 1.20 (t, *J* = 7.1, 3H).

^{13}C NMR (101 MHz, $CDCl_3$) δ 165.8, 142.4, 135.5, 129.5, 128.8, 127.8, 117.7, 42.4, 41.2, 15.1, 13.2.

LCMS (ESI$^+$) rt = 2.2 min, m/z = 204.2 [M+H]$^+$

***N*,*N*-diethylheptanamide 4w**

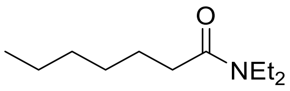

Synthesised according to the general method for the synthesis of amides from heptanoic acid (0.128 g, 0.98 mmol) and diethylamine (0.072 g, 0.98 mmol). The crude material was purified by flash column chromatography (100% hexane to 50% hexane 50% ethyl acetate) to give the desired product as a clear oil in 87% yield (0.157 g).

Characterisation data were consistent with the previously reported literature values.[25]

^{1}H NMR (400 MHz, $CDCl_3$) δ 3.36 (q, *J* = 7.1, 2H), 3.30 (q, *J* = 7.1, 2H), 2.32 – 2.24 (m, 2H), 1.68 – 1.56 (m, 2H), 1.34 – 1.27 (m, 6H), 1.17 (t, *J* = 7.1, 3H), 1.10 (t, *J* = 7.1, 3H), 0.92 – 0.83 (m, 3H).

^{13}C NMR (101 MHz, $CDCl_3$) δ 172.4, 42.0, 40.0, 33.2, 31.7, 29.2, 25.5, 22.5, 14.4, 14.0, 13.1.

LCMS (ESI$^+$) rt = 2.5 min, m/z = 186.2 [M+H]$^+$

***N*,*N*-diethyl-2-(4-(trifluoromethyl)phenyl)acetamide 4x**

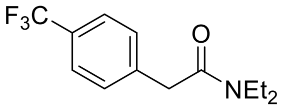

Synthesised according to the general method for the synthesis of amides from 4-(trifluoromethyl)phenylacetic acid (0.200 g, 0.98 mmol) and diethylamine (0.072 g, 0.98 mmol). The crude material was purified by flash column chromatography (100% hexane to 50% hexane 50% ethyl acetate) to give the desired product as a clear oil in 67% yield (0.167 g).

Characterisation data were consistent with the previously reported literature values.[26]

^{1}H NMR (400 MHz, $CDCl_3$) δ 7.59 (d, *J* = 7.9, 2H), 7.39 (d, *J* = 7.9, 2H), 3.77 (s, 2H), 3.42 (q, *J* = 7.1, 2H), 3.33 (q, *J* = 7.1, 2H), 1.20 – 1.11 (m, 6H).

^{19}F NMR (376 MHz, $CDCl_3$) δ -62.45 (s, 3F).

^{13}C NMR (101 MHz, $CDCl_3$) δ 169.5, 139.5 (q, *J* < 2.0), 129.2, 128.4 (q, *J* = 35.7), 125.5 (q, *J* = 3.7), 121.5 (q, *J* = 271.9), 42.5, 40.4, 40.3, 14.3, 12.9.

LCMS (ESI$^+$) rt = 2.5 min, m/z = 260.2 $[M+H]^+$

N-phenylcinnamamide 4y

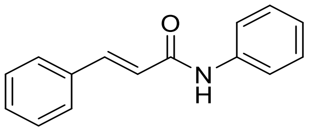

Synthesised according to the general method for the synthesis of amides from *trans*-cinnamic acid (0.126 g, 0.85 mmol) and aniline (0.079, 0.85 mmol). The crude material was purified by flash column chromatography (100% hexane to 50% hexane 50% ethyl acetate) to give the desired product as a white solid in 68% yield (0.128 g).

Characterisation data were consistent with the previously reported literature values.[27]

^{1}H NMR (400 MHz, $CDCl_3$) δ 7.82 – 7.73 (m, 2H), 7.68 (d, *J* = 8.0, 2H), 7.55 – 7.45 (m, 2H), 7.41 – 7.32 (m, 5H), 7.15 (t, *J* = 7.4, 1H), 6.63 (d, *J* = 15.5, 1H).

^{13}C NMR (101 MHz, $CDCl_3$) δ 164.1, 142.4, 138.1, 134.6, 130.0, 129.1, 128.9, 128.0, 124.5, 120.9, 120.1.

LCMS (ESI$^+$) rt = 2.4 min, m/z = 224.1 $[M+H]^+$

N-(3-nitrophenyl)benzamide 4z

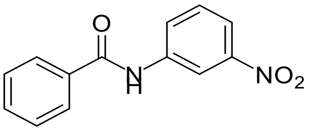

Synthesised according to the general method for the synthesis of amides from benzoic acid (0.100 g, 0.82 mmol) and 3-nitroaniline (0.113 g, 0.82 mmol) with the following modification, after addition of the amine the reaction mixture was heated to 100 °C in a sealed tube using a hotplate. The crude material was purified by flash column chromatography (100% hexane to 50% hexane 50% ethyl acetate) to give the desired product as a yellow solid in 73% yield (0.144 g).

Characterisation data were consistent with the previously reported literature values.[28]

^{1}H NMR (400 MHz, $CDCl_3$) δ 8.53 (t, *J* = 2.2, 1H), 8.13 (ddd, *J* = 8.2, 2.2, 1.0, 1H), 8.09 – 8.02 (m, 2H), 7.96 – 7.89 (m, 2H), 7.66 – 7.59 (m, 1H), 7.59 – 7.53 (m, 3H).

^{13}C NMR (101 MHz, $CDCl_3$) δ 165.9, 148.7, 139.0, 134.0, 132.5, 130.0, 129.0, 127.1, 125.8, 119.2, 114.9.

LCMS (ESI$^+$) rt = 2.3 min, m/z = 243.1 $[M+H]^+$

N-(pyridin-2-yl)benzamide 4aa

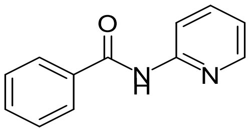

Synthesised according to the general method for the synthesis of amides from benzoic acid (0.100 g, 0.82 mmol) and 2-aminopyridine (0.077 g, 0.82 mmol) with the following modification, after addition of the amine the reaction mixture was heated to 100 °C in a sealed tube using a hotplate. The crude material was purified by flash column chromatography (100% hexane to 50% hexane 50% ethyl acetate) to give the desired product as a cream coloured solid in 86% yield (0.140 g).

Characterisation data were consistent with the previously reported literature values.[29]

^{1}H NMR (400 MHz, $CDCl_3$) δ 9.38 (brs, 1H), 8.43 (d, *J* = 8.4, 1H), 8.10 (ddd, *J* = 5.0, 1.9, 0.9, 1H), 7.97 – 7.90 (m, 2H), 7.76 (ddd, *J* = 8.4, 7.4, 1.9, 1H), 7.60 – 7.53 (m, 1H), 7.51 – 7.44 (m, 2H), 7.03 (ddd, *J* = 7.4, 5.0, 1.1, 1H).

^{13}C NMR (101 MHz, $CDCl_3$) δ 166.1, 151.8, 147.8, 138.5, 134.4, 132.2, 128.8, 127.4, 119.9, 114.4.

LCMS (ESI$^+$) rt = 1.4 min, m/z = 199.1 [M+H]$^+$

N-methyl-*N*-phenylpropionamide 4ab

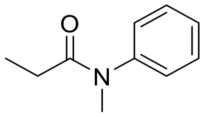

Synthesised according to the general method for the synthesis of amides from propanoic acid (0.100 g, 1.35 mmol) and *N*-methylaniline (0.144 g, 1.35 mmol). The crude material was purified by flash column chromatography (100% hexane to 50% hexane 50% ethyl acetate) to give the desired product as a white solid in 57% yield (0.126 g).

Characterisation data were consistent with the previously reported literature values.[10]

^{1}H NMR (400 MHz, $CDCl_3$) δ 7.42 (appt, *J* = 7.5, 2H), 7.34 (appt, *J* = 7.3, 1H), 7.19 (appd, *J* = 7.4, 2H), 3.27 (s, 3H), 2.08 (q, *J* = 7.6, 2H), 1.05 (t, *J* = 7.6, 3H).

^{13}C NMR (101 MHz, $CDCl_3$) δ 174.0, 144.2, 129.7, 127.7, 127.3, 37.3, 27.5, 9.7.

LCMS (ESI$^+$) rt = 1.8 min, m/z = 164.1 [M+H]$^+$

4-methoxy-*N*-phenylbenzamide 4ac

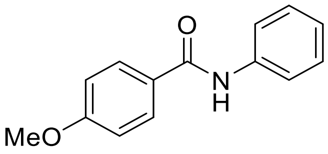

Synthesised according to the general method for the synthesis of amides from 4-methoxy benzoic acid (0.100 g, 0.66 mmol) and aniline (0.061 g, 0.66 mmol). The crude material was purified by flash column chromatography (100% hexane to 50% hexane 50% ethyl acetate) to give the desired product as a cream coloured solid in 86% yield (0.129 g).

Characterisation data were consistent with the previously reported literature values.[30]

^{1}H NMR (400 MHz, $CDCl_3$) δ 7.90 – 7.84 (m, 2H), 7.77 (brs, 1H), 7.68 – 7.63 (m, 2H), 7.43 – 7.36 (m, 2H), 7.21 – 7.13 (m, 1H), 7.04 – 6.97 (m, 2H), 3.90 (s, 3H).

^{13}C NMR (101 MHz, $CDCl_3$) δ 165.2, 162.5, 138.1, 129.1, 128.9, 127.1, 124.4, 120.1, 114.0, 55.5.

LCMS (ESI$^+$) rt = 2.2 min, m/z = 228.1 [M+H]$^+$

N-methyl-*N*-phenylbenzamide 4ad

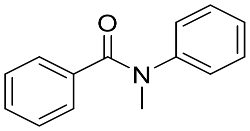

Synthesised according to the general method for the synthesis of amides from benzoic acid (0.100 g, 0.82 mmol) and *N*-methylaniline (0.088 g, 0.82 mmol). The crude material was purified by flash column chromatography (100% hexane to 50% hexane 50% ethyl acetate) to give the desired product as a white solid in 81% yield (0.141 g).

Characterisation data were consistent with the previously reported literature values.[31]

^{1}H NMR (400 MHz, $CDCl_3$) δ 7.34 – 7.29 (m, 2H), 7.27 – 7.21 (m, 3H), 7.21 – 7.15 (m, 3H), 7.08 – 7.03 (m, 2H), 3.53 (s, 3H).

^{13}C NMR (101 MHz, $CDCl_3$) δ 170.9, 144.8, 135.8, 129.7, 129.2, 128.7, 127.8, 126.9, 126.6, 38.5.

LCMS (ESI^{+}) rt = 2.1 min, m/z = 212.1 $[M+H]^{+}$

4-methoxyphenyl benzoate 6a

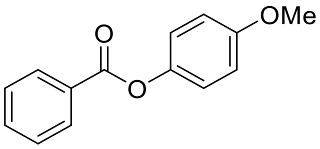

Synthesised according to the general method for the synthesis of esters from benzoic acid (0.100 g, 0.82 mmol) and 4-methoxyphenol (0.102 g, 0.82 mmol). The crude material was purified by flash column chromatography (100% hexane to 50% hexane 50% ethyl acetate) to give the desired product as a white solid in 68% yield (0.128 g).

Characterisation data were consistent with the previously reported literature values.[32]

^{1}H NMR (400 MHz, $CDCl_3$) δ 8.26 – 8.20 (m, 2H), 7.70 – 7.64 (m, 2H), 7.58 – 7.50 (m, 2H), 7.17 (d, J = 9.1, 2H), 6.98 (d, J = 9.1, 2H), 3.86 (s, 3H).

^{13}C NMR (101 MHz, $CDCl_3$) δ 165.6, 157.4, 144.4, 133.5, 130.2, 129.7, 128.6, 122.5, 114.6, 55.6.

LCMS (ESI^+) rt = 2.8 min, m/z = 229.1 $[M+H]^+$

4-nitrophenyl benzoate 6b

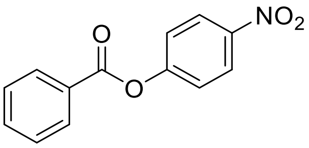

Synthesised according to the general method for the synthesis of esters from benzoic acid (0.100 g, 0.82 mmol) and 4-nitrophenol (0.114 g, 0.82 mmol). The crude material was purified by flash column chromatography (100% hexane to 50% hexane 50% ethyl acetate) to give the desired product as a yellow solid in 50% yield (0.099 g).

Characterisation data were consistent with the previously reported literature values.[33]

^1H NMR (400 MHz, $CDCl_3$) δ 8.36 (d, *J* = 9.1, 2H), 8.26 – 8.20 (m, 2H), 7.74 – 7.68 (m, 1H), 7.57 (appt, *J* = 7.7, 2H), 7.45 (d, *J* = 9.1, 2H).

^{13}C NMR (101 MHz, $CDCl_3$) δ 164.3, 155.8, 145.4, 134.3, 130.4, 128.8, 128.6, 125.3, 122.7.

LCMS (ESI^-) rt = 2.6 min, m/z = 278.1 $[M+K]^-$

4-nitrophenyl propionate 6c

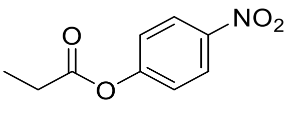

Synthesised according to the general method for the synthesis of esters from propanoic acid (0.100 g, 1.35 mmol) and 4-nitrophenol (0.188 g, 1.35 mmol). The crude material was purified by flash column chromatography (100% hexane to 50% hexane 50% ethyl acetate) to give the desired product as a white solid in 23% yield (0.060 g).

Characterisation data were consistent with the previously reported literature values.[34]

^{1}H NMR (400 MHz, $CDCl_3$) δ 8.30 (d, *J* = 9.1, 2H), 7.31 (d, *J* = 9.1, 2H), 2.67 (q, *J* = 7.5, 2H), 1.31 (t, *J* = 7.5, 3H).

^{13}C NMR (101 MHz, $CDCl_3$) δ 172.0, 155.5, 125.2, 122.4, 27.8, 8.9.

4-methoxyphenyl propionate 6d

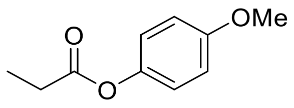

Synthesised according to the general method for the synthesis of esters from propanoic acid (0.100 g, 1.35 mmol) and 4-methoxyphenol (0.167 g, 1.35 mmol). The crude material was purified by flash column chromatography (100% hexane to 50% hexane 50% ethyl acetate) to give the desired product as a clear oil in 24% yield (0.061 g).

Characterisation data were consistent with the previously reported literature values.[35]

^{1}H NMR (400 MHz, $CDCl_3$) δ 7.03 (d, *J* = 9.1, 2H), 6.91 (d, *J* = 9.1, 2H), 3.82 (s, 3H), 2.60 (q, *J* = 7.6, 2H), 1.28 (t, *J* = 7.6, 3H).

^{13}C NMR (101 MHz, $CDCl_3$) δ 173.4, 157.2, 144.3, 122.3, 114.4, 55.6, 27.7, 9.1.

LCMS (ESI^+) rt = 2.2 min, m/z = 181.1 $[M+H]^+$

NMR Data for Synthesised Compounds

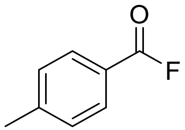

2a
400 MHz

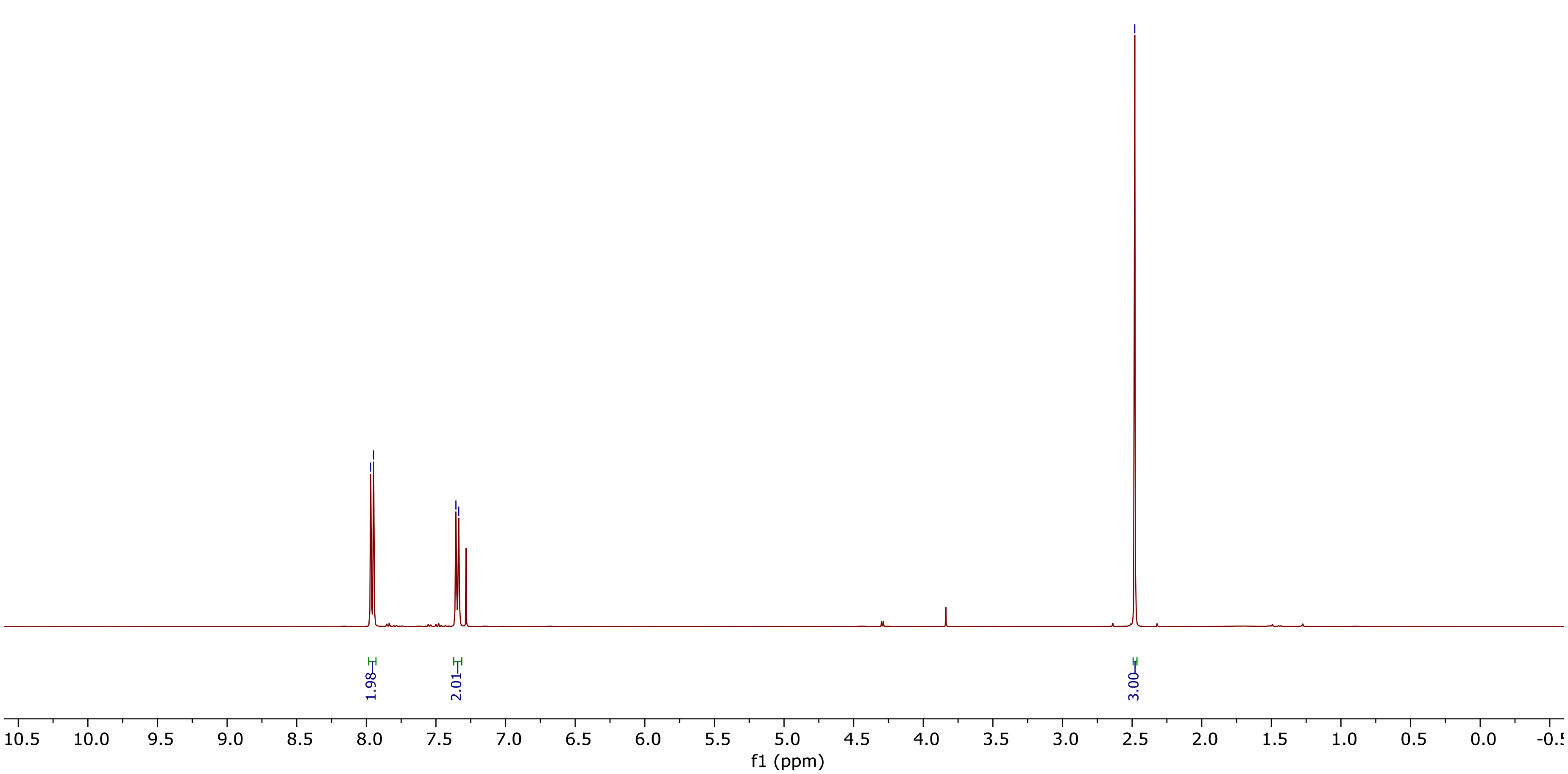
15095062.10.fid
SLC:WDB:WB7-54
Proton.dur CDCl3 /home/nmr/localdata walkup 10
7.97
7.95
7.36
7.34
2.48
1.98
2.01
3.00
10.5 10.0 9.5 9.0 8.5 8.0 7.5 7.0 6.5 6.0 5.5 5.0 4.5 4.0 3.5 3.0 2.5 2.0 1.5 1.0 0.5 0.0
f1 (ppm)

15095062.13.fid
SLC:WDB:WB7-54
F19_limits_dec.dur CDCl3 /home/nmr/localdata walkup 10

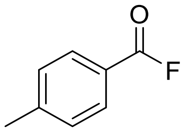

2a
376 MHz

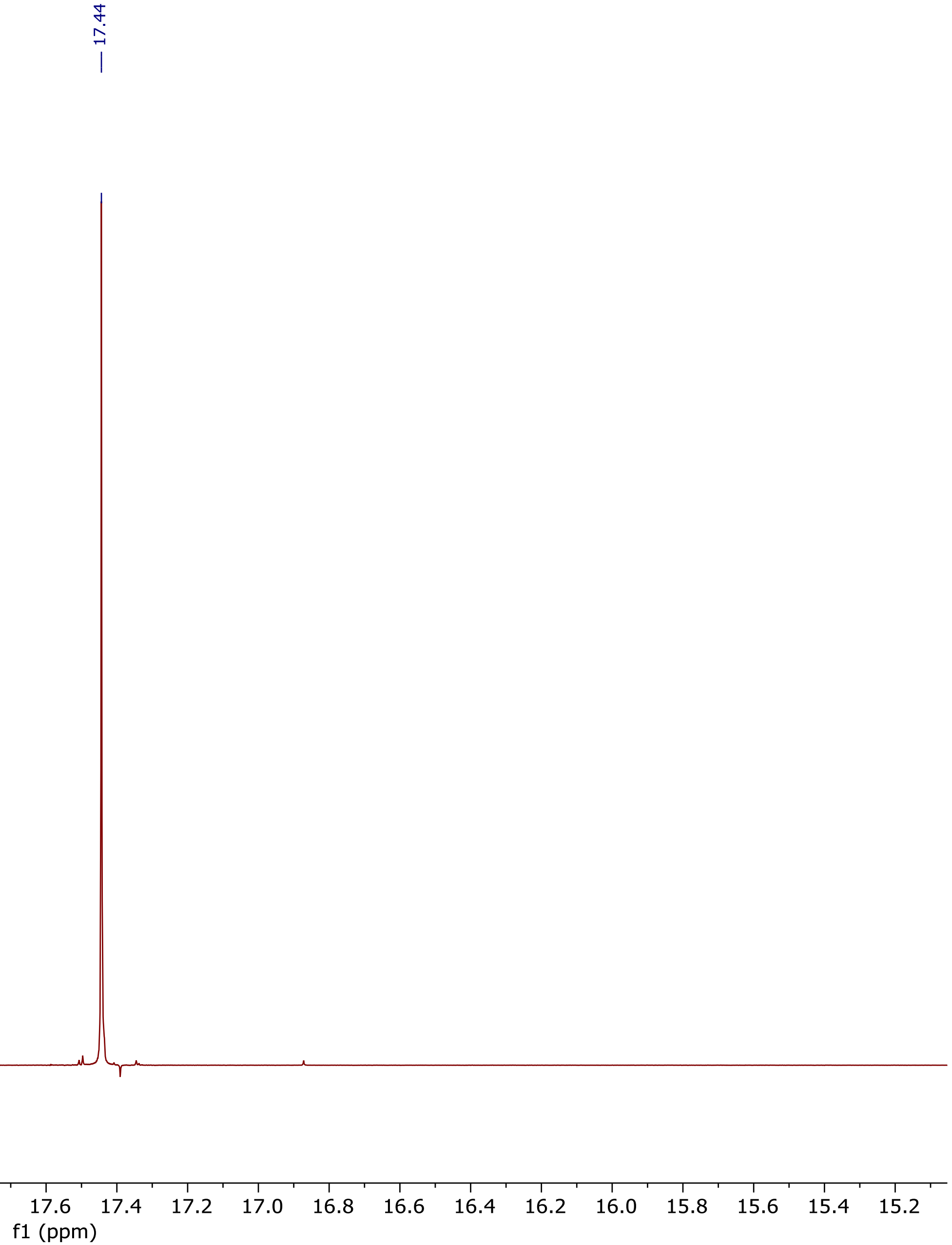

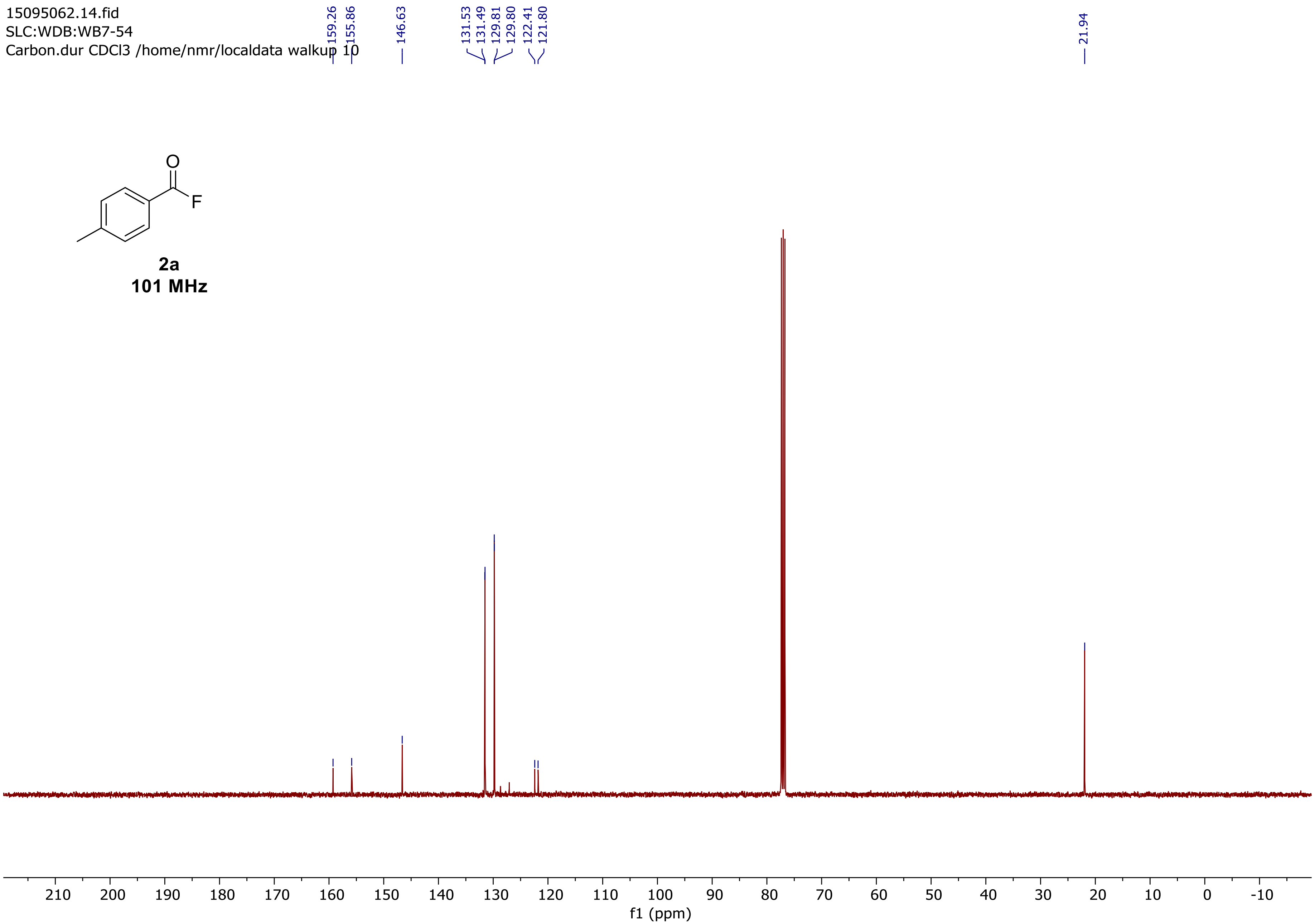
15095062.14.fid
SLC:WDB:WB7-54
Carbon.dur CDCl3 /home/nmr/localdata walkup 10
159.26
155.86
146.63
131.53
131.49
129.81
129.80
122.41
121.80
21.94
O
F
2a
101 MHz
210 200 190 180 170 160 150 140 130 120 110 100 90 80 70 60 50 40 30 20 10 0 -10
f1 (ppm)

06170330.10.fid
SLC:WDGB:WB7-41 F18-28
Proton.dur CDCl3 /home/nmr/localdata walkup 44

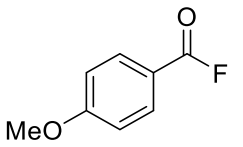

2b
400 MHz

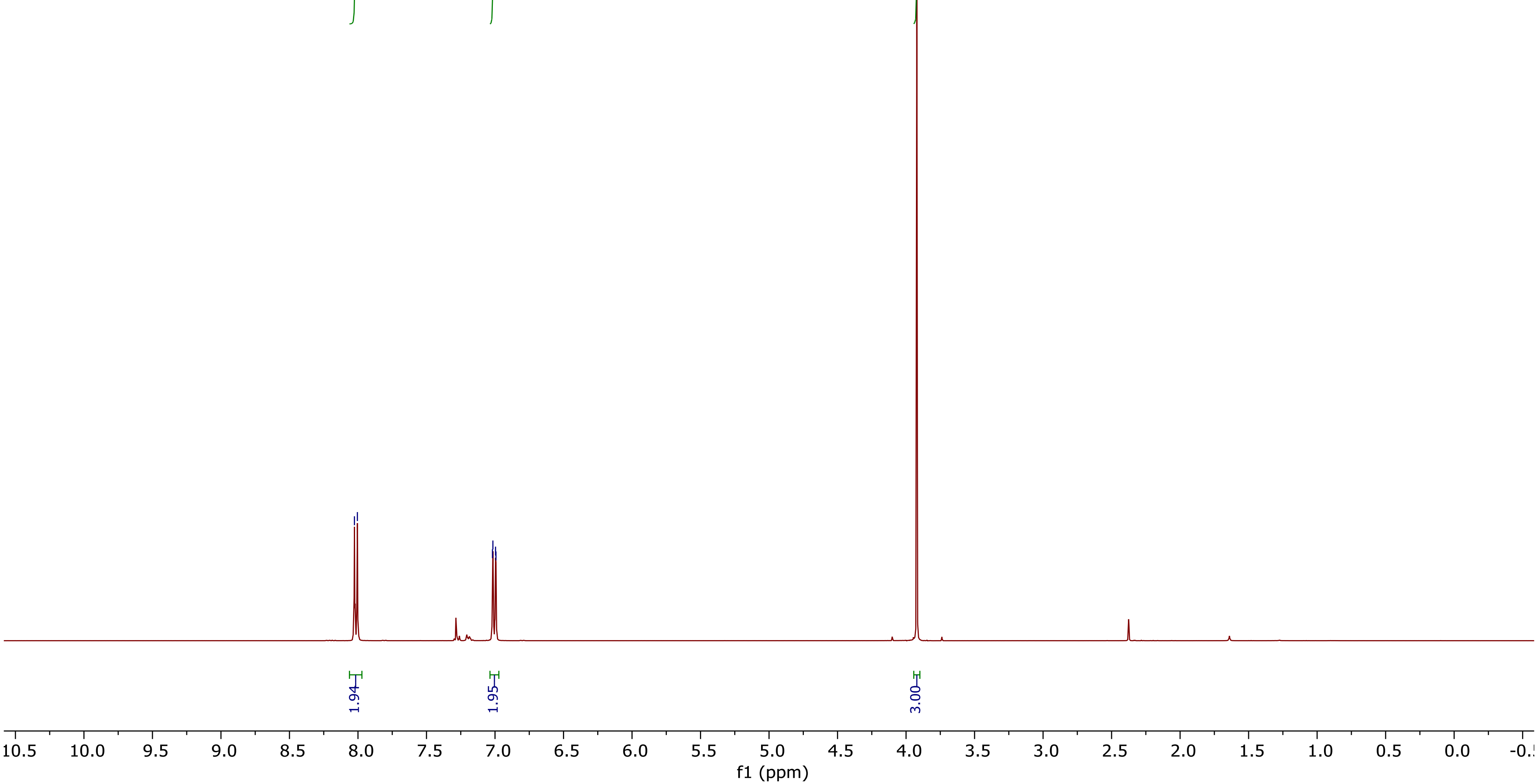

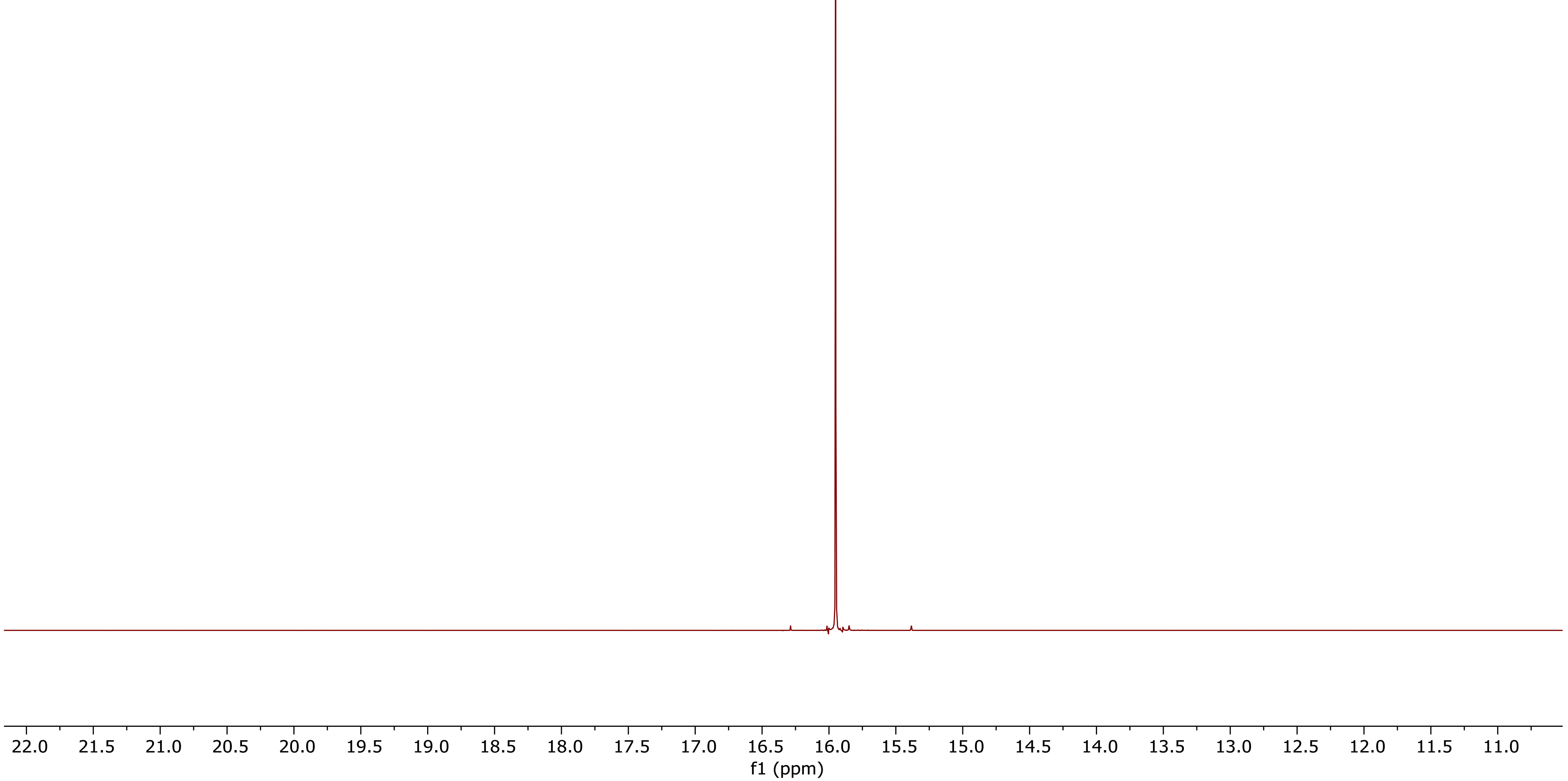
06170330.13.fid
SLC:WDGB:WB7-41 F18-28
F19_limits_dec.dur CDCl3 /home/nmr/localdata walkup 44
15.95
22.0 21.5 21.0 20.5 20.0 19.5 19.0 18.5 18.0 17.5 17.0 16.5 16.0 15.5 15.0 14.5 14.0 13.5 13.0 12.5 12.0 11.5 11.0
f1 (ppm)

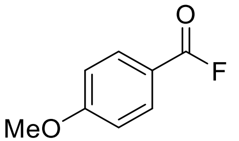
O
F
MeO

2b
376 MHz

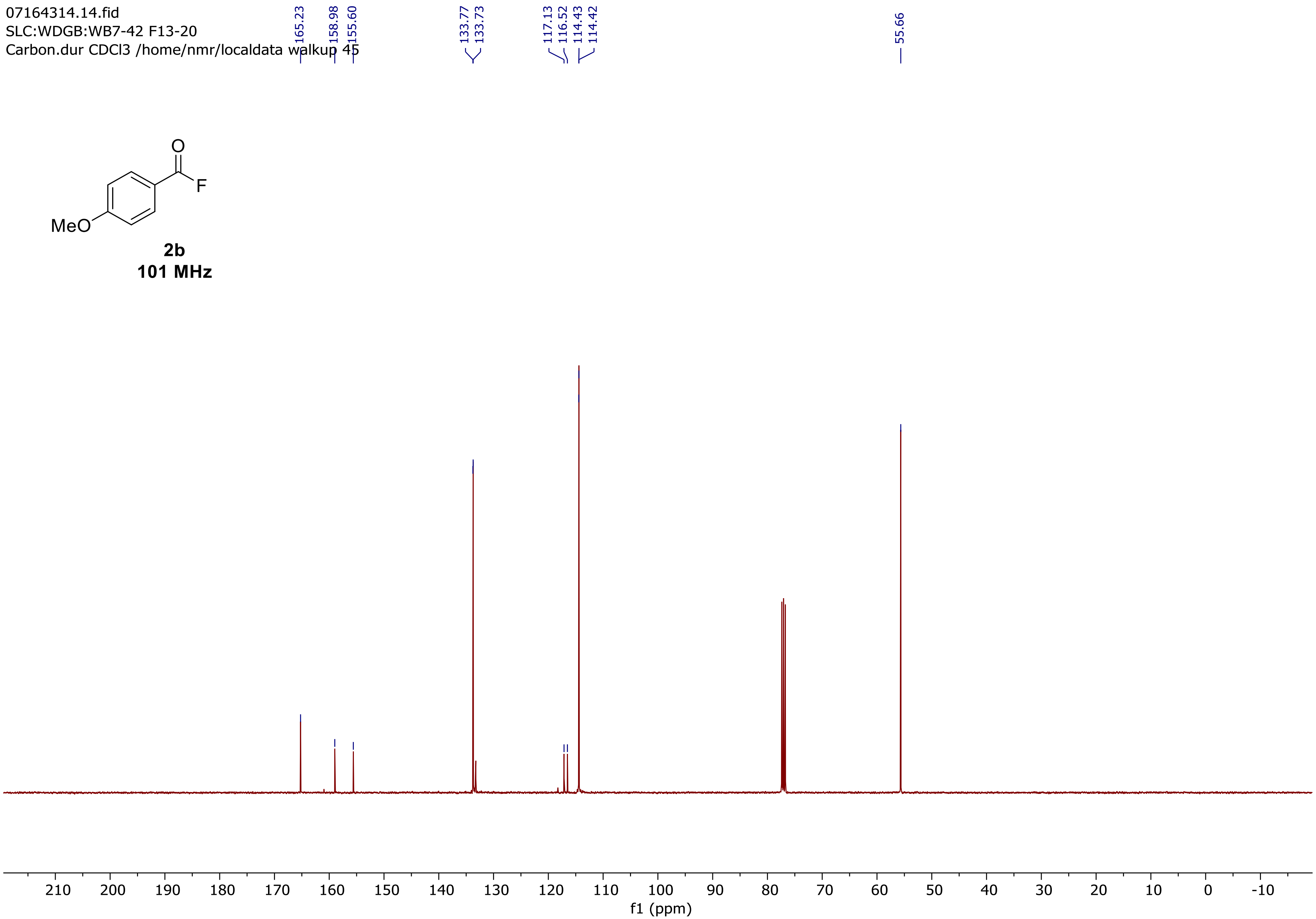

07164314.14.fid
SLC:WDGB:WB7-42 F13-20
Carbon.dur CDCl3 /home/nmr/localdata walkup 45
165.23
158.98
155.60
133.77
133.73
117.13
116.52
114.43
114.42
55.66
210 200 190 180 170 160 150 140 130 120 110 100 90 80 70 60 50 40 30 20 10 0 -10
f1 (ppm)
O
F
MeO

2b
101 MHz

28153762.10.1.1r
SLC:WBD:WB7-66-5
Proton.dur CDCl3 /home/nmr/localdata walkup 59

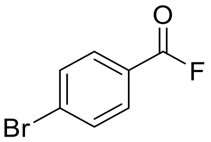

2c
400 MHz

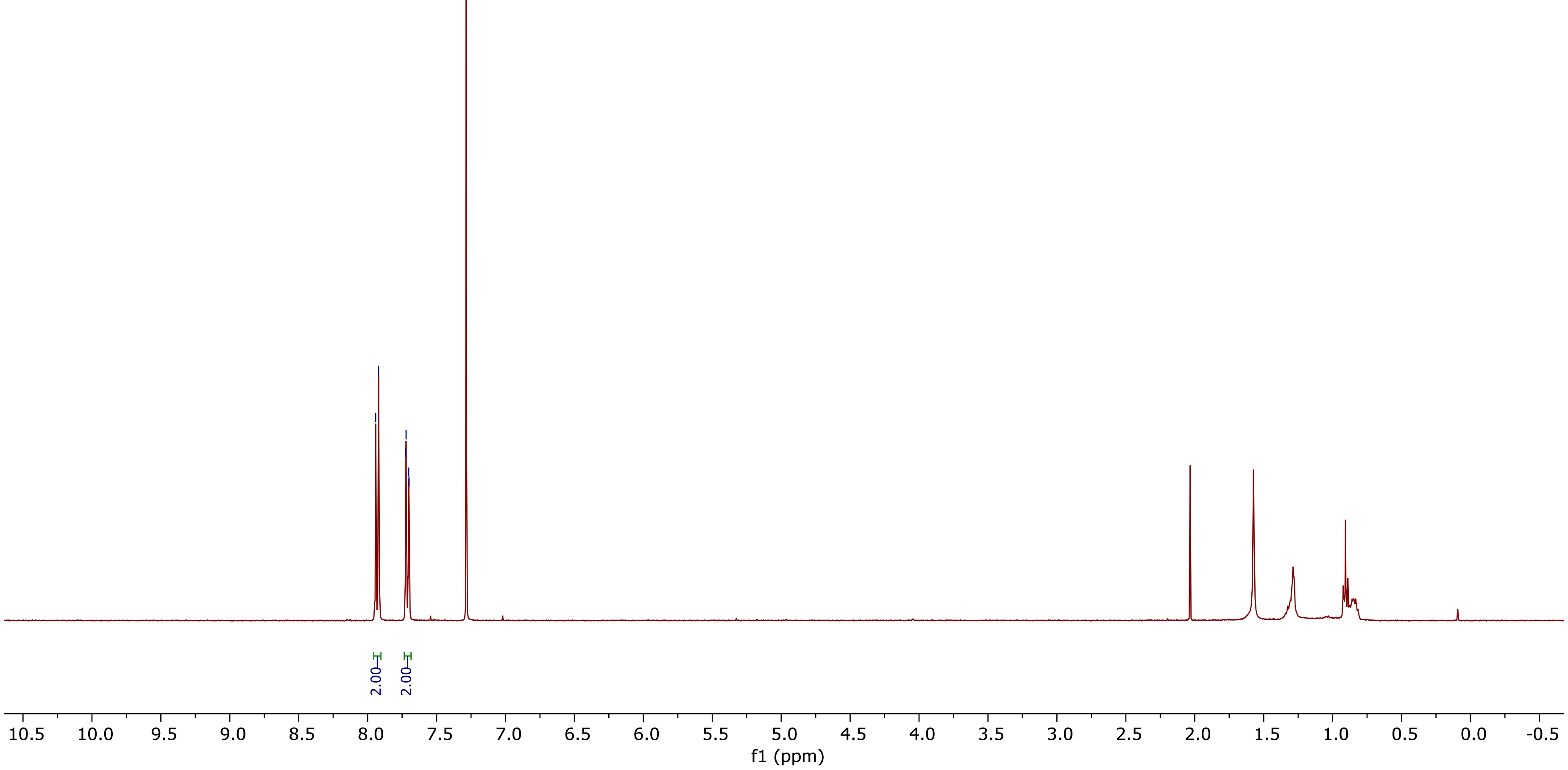

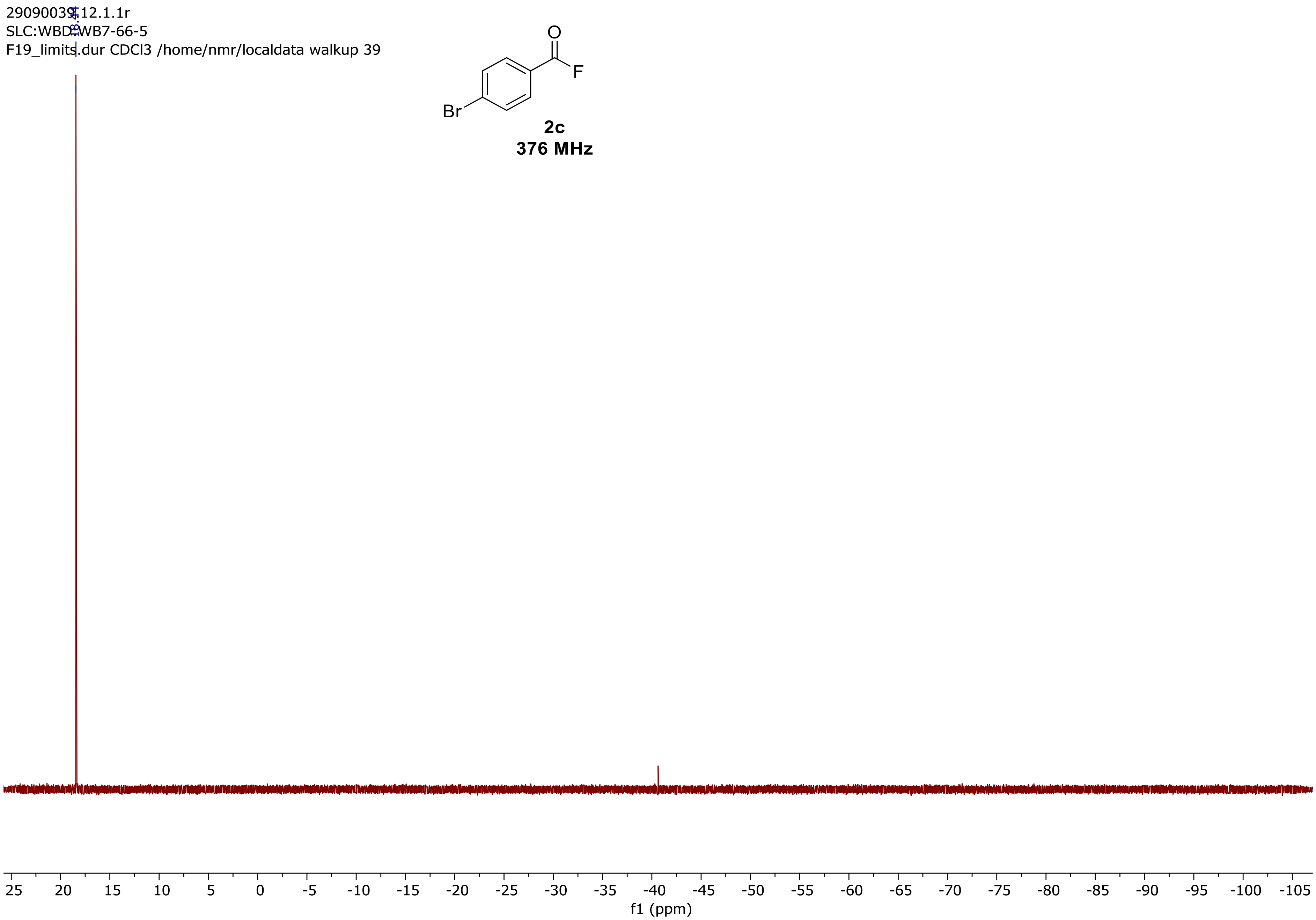
29090039.12.1.1r
SLC:WBD.WB7-66-5
F19_limits.dur CDCl3 /home/nmr/localdata walkup 39
18.44
25 20 15 10 5 0 -5 -10 -15 -20 -25 -30 -35 -40 -45 -50 -55 -60 -65 -70 -75 -80 -85 -90 -95 -100 -105
f1 (ppm)
O
F
Br

2c
376 MHz

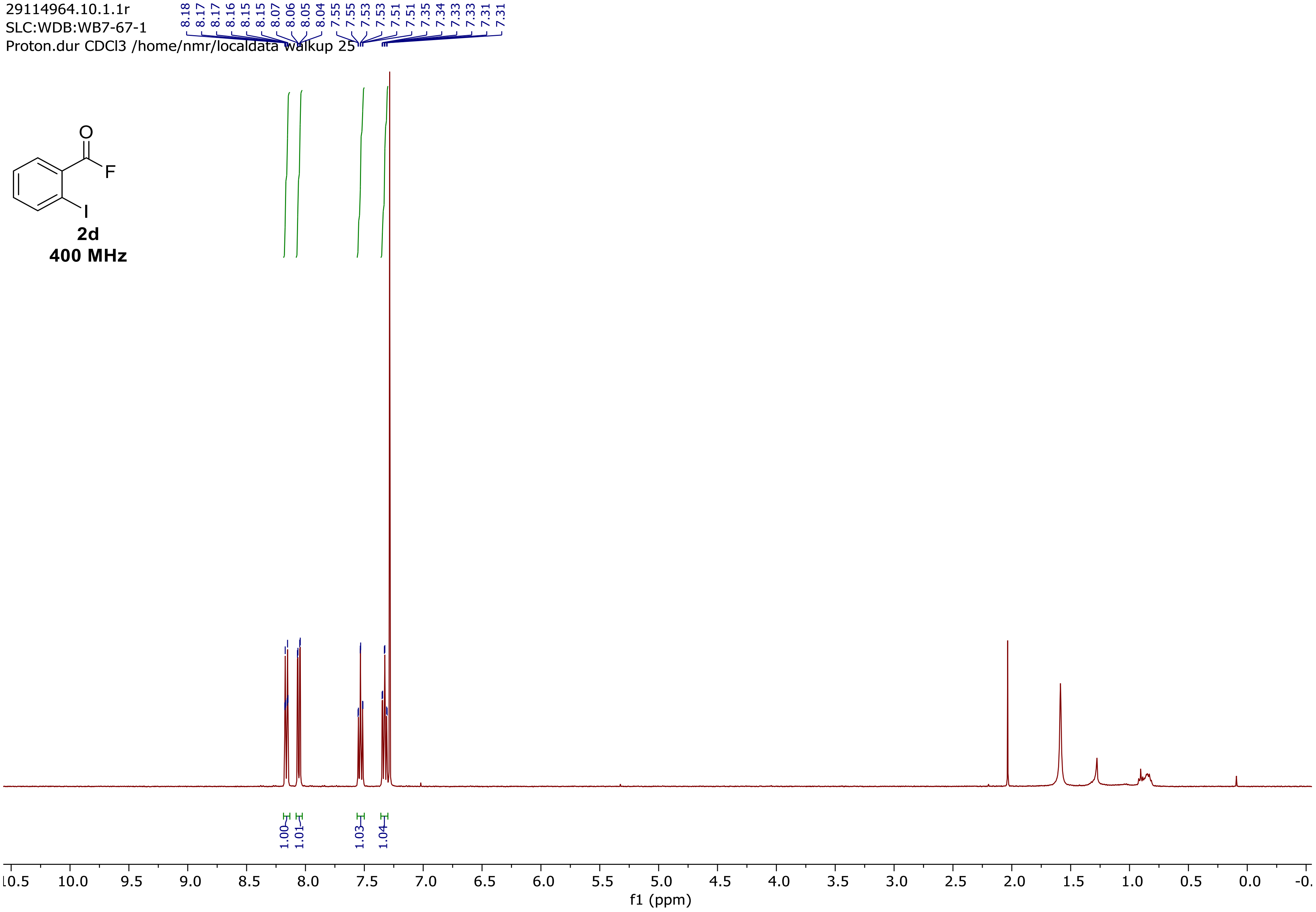
29114964.10.1.1r
SLC:WDB:WB7-67-1
Proton.dur CDCl3 /home/nmr/localdata walkup 25
8.18
8.17
8.17
8.16
8.15
8.15
8.07
8.06
8.05
8.04
7.55
7.55
7.53
7.53
7.51
7.51
7.35
7.34
7.33
7.33
7.31
7.31
O
F
I
2d
400 MHz
1.00
1.01
1.03
1.04
10.5
10.0
9.5
9.0
8.5
8.0
7.5
7.0
6.5
6.0
5.5
5.0
4.5
4.0
3.5
3.0
2.5
2.0
1.5
1.0
0.5
0.0
f1 (ppm)

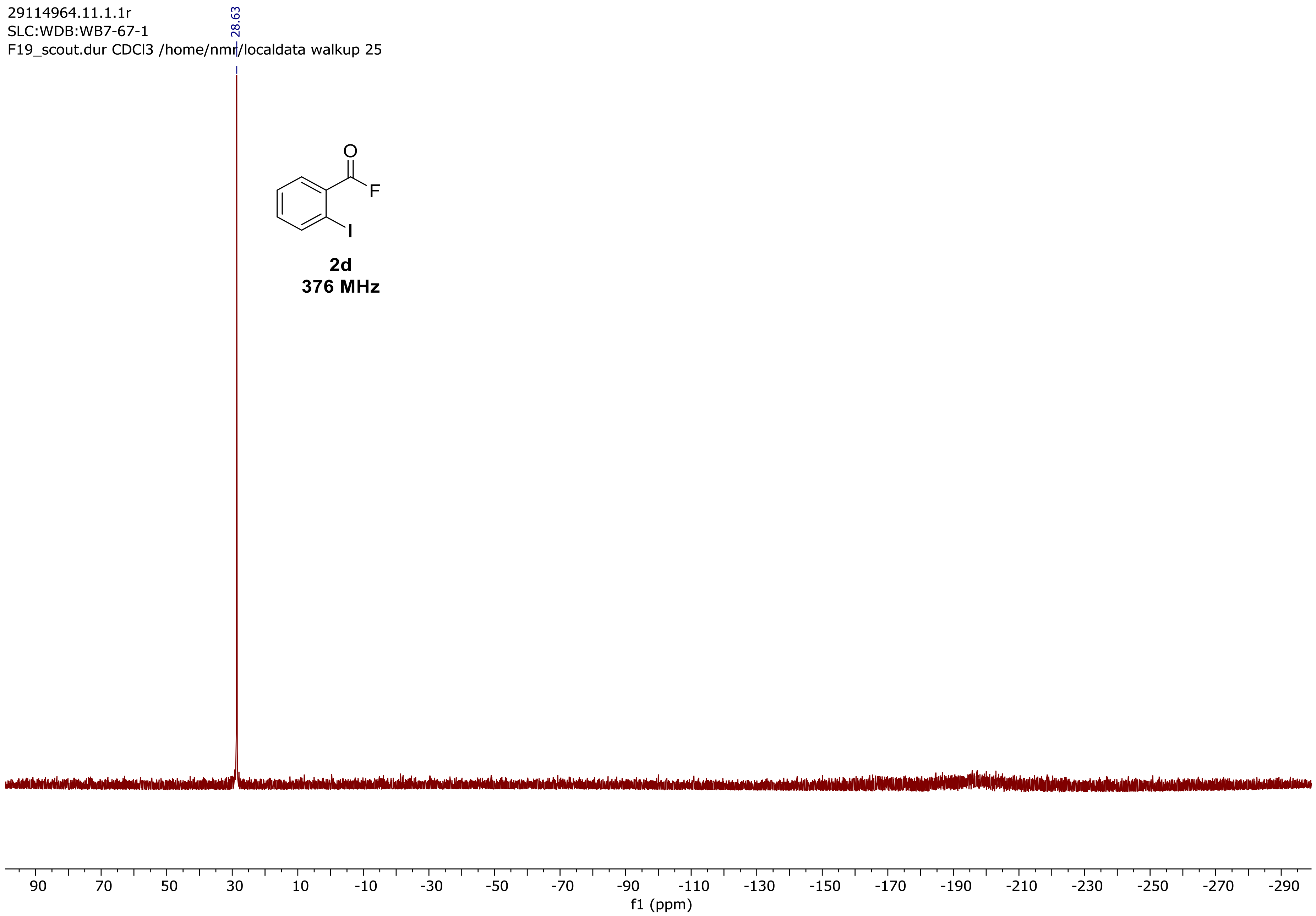
29114964.11.1.1r
SLC:WDB:WB7-67-1
F19_scout.dur CDCl3 /home/nmr/localdata walkup 25
28.63
O
F
I
2d
376 MHz
90 70 50 30 10 -10 -30 -50 -70 -90 -110 -130 -150 -170 -190 -210 -230 -250 -270 -290
f1 (ppm)

29114964.14.fid
SLC:WDB:WB7-67-1
Carbon.dur CDCl3 /home/nmr/localdata walkup 25

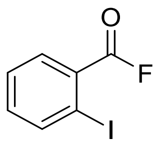

2d
101 MHz

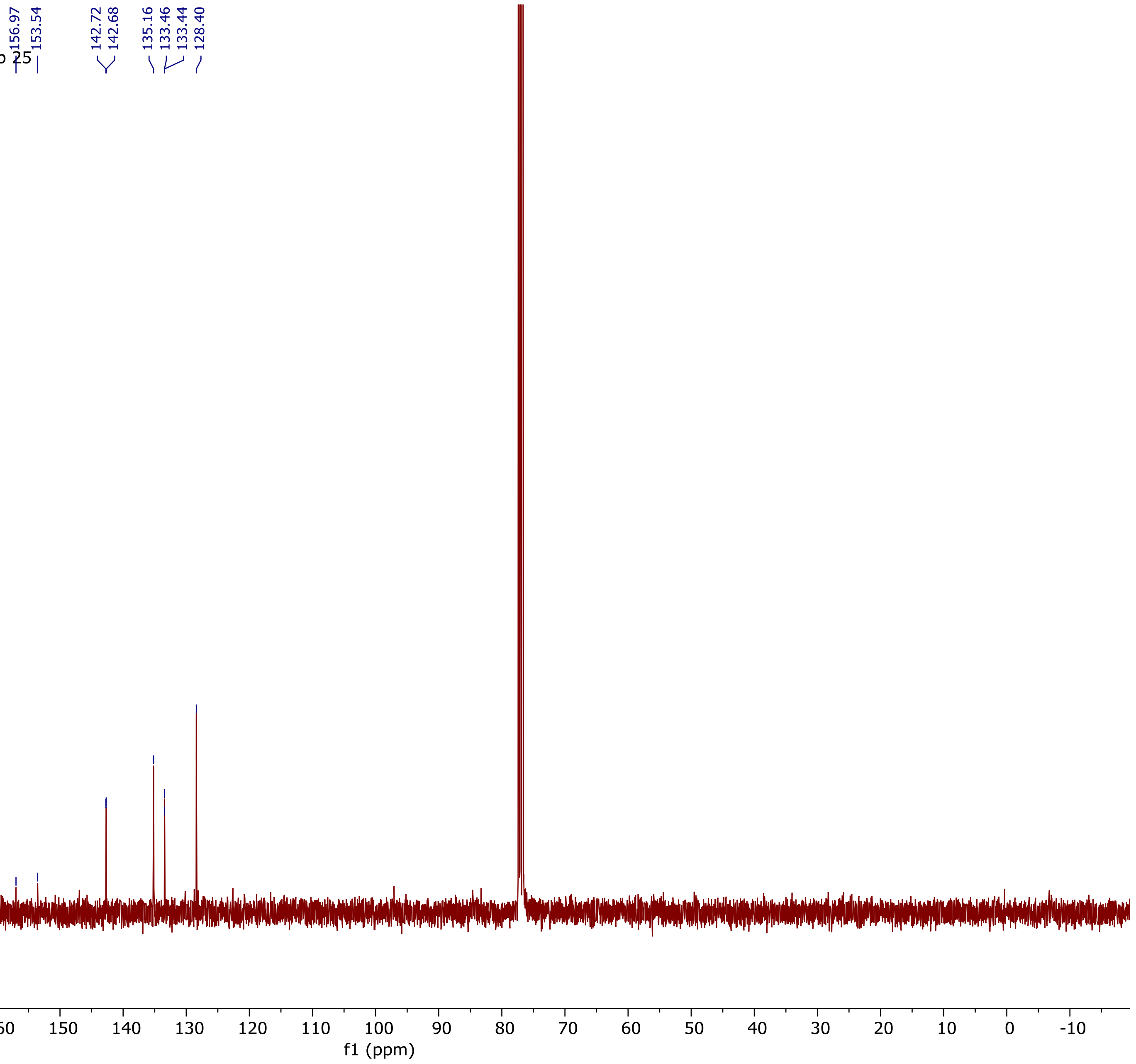

28153761.10.1.1r
SLC:WBD:WB7-66-4
Proton.dur CDCl3 /home/nmr/localdata walkup 38

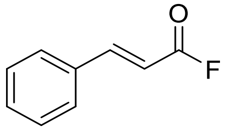

2e
400 MHz

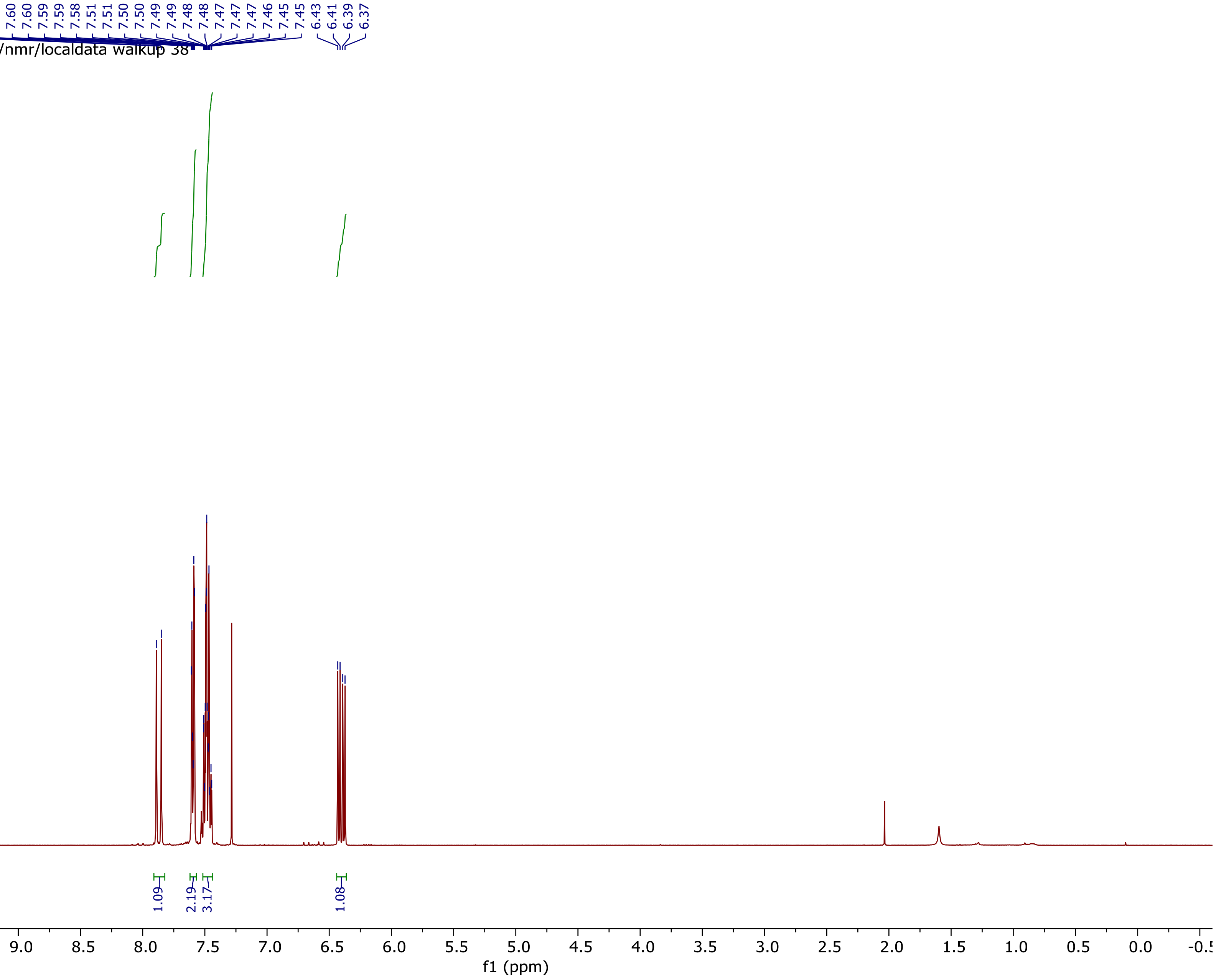

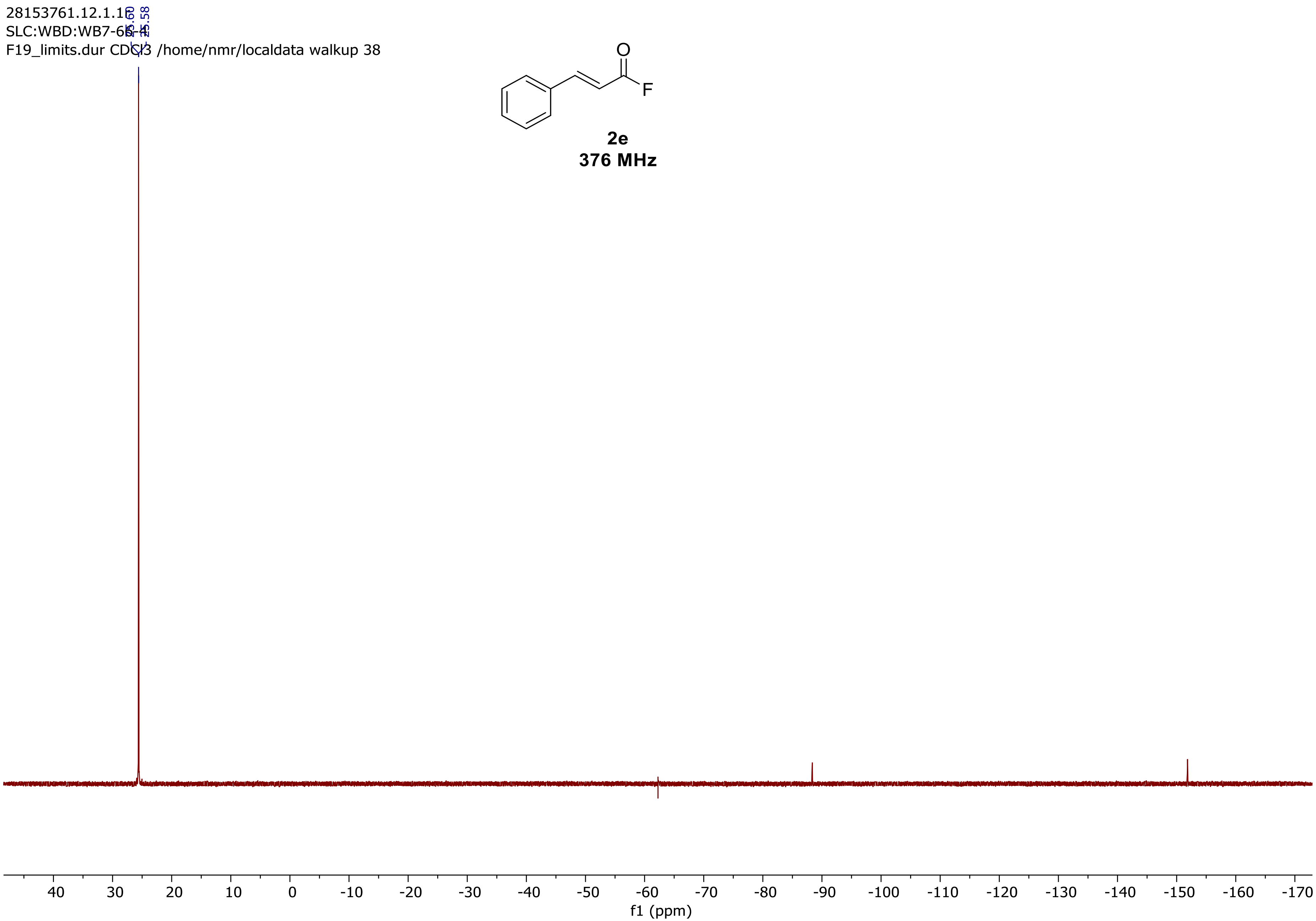
28153761.12.1.1r
SLC:WBD:WB7-66-4
F19_limits.dur CDCl3 /home/nmr/localdata walkup 38
25.60
25.58
O
F
2e
376 MHz
40 30 20 10 0 -10 -20 -30 -40 -50 -60 -70 -80 -90 -100 -110 -120 -130 -140 -150 -160 -170
f1 (ppm)

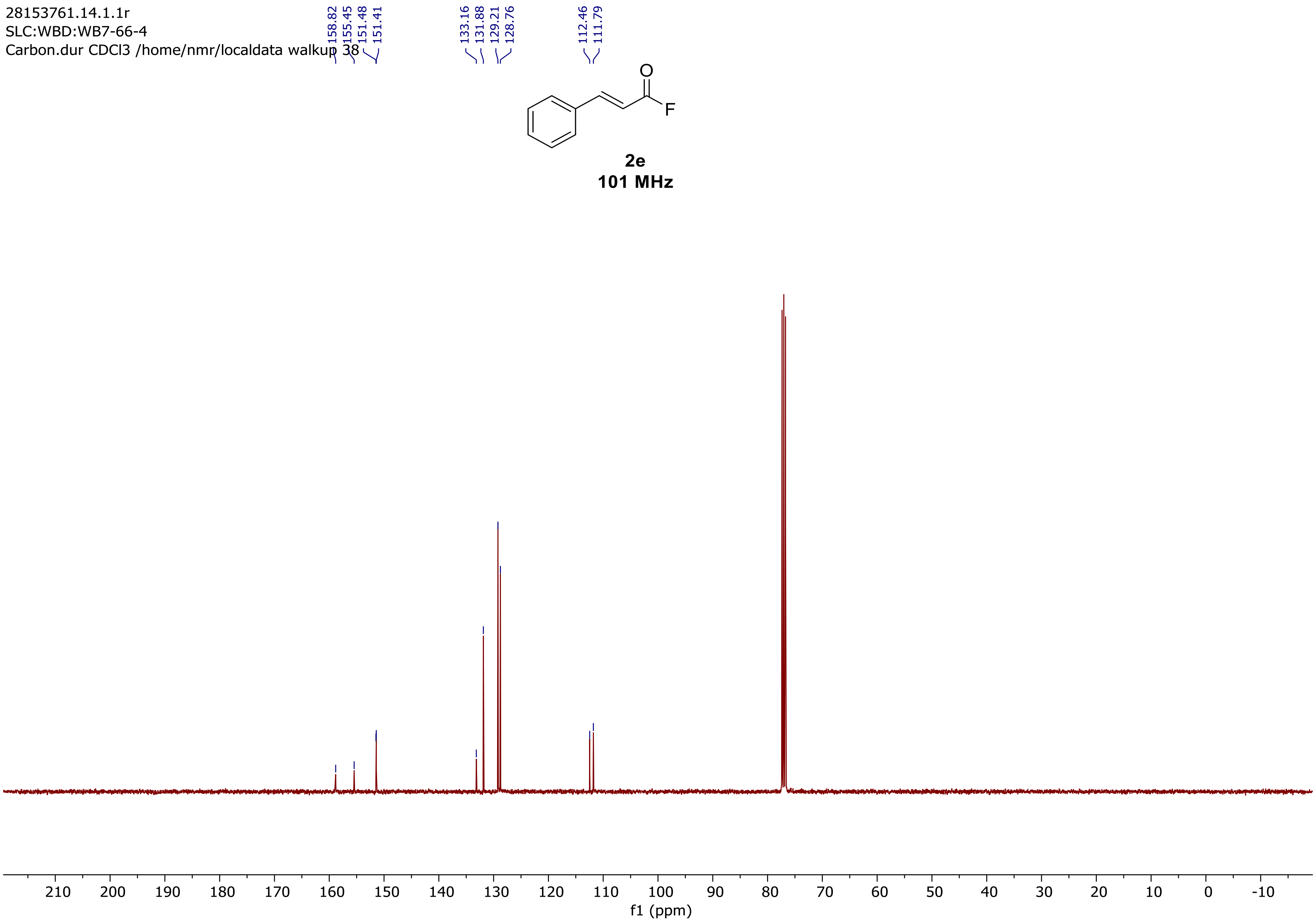
28153761.14.1.1r
SLC:WBD:WB7-66-4
Carbon.dur CDCl3 /home/nmr/localdata walkup 38
158.82
155.45
151.48
151.41
133.16
131.88
129.21
128.76
112.46
111.79
O
F
2e
101 MHz
210
200
190
180
170
160
150
140
130
120
110
100
90
80
70
60
50
40
30
20
10
0
-10
f1 (ppm)

29114966.10.1.1r
SLC:WDB:WB7-67-2
Proton.dur CDCl3 /home/nmr/localdata walkup 27

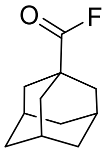

2f
400 MHz

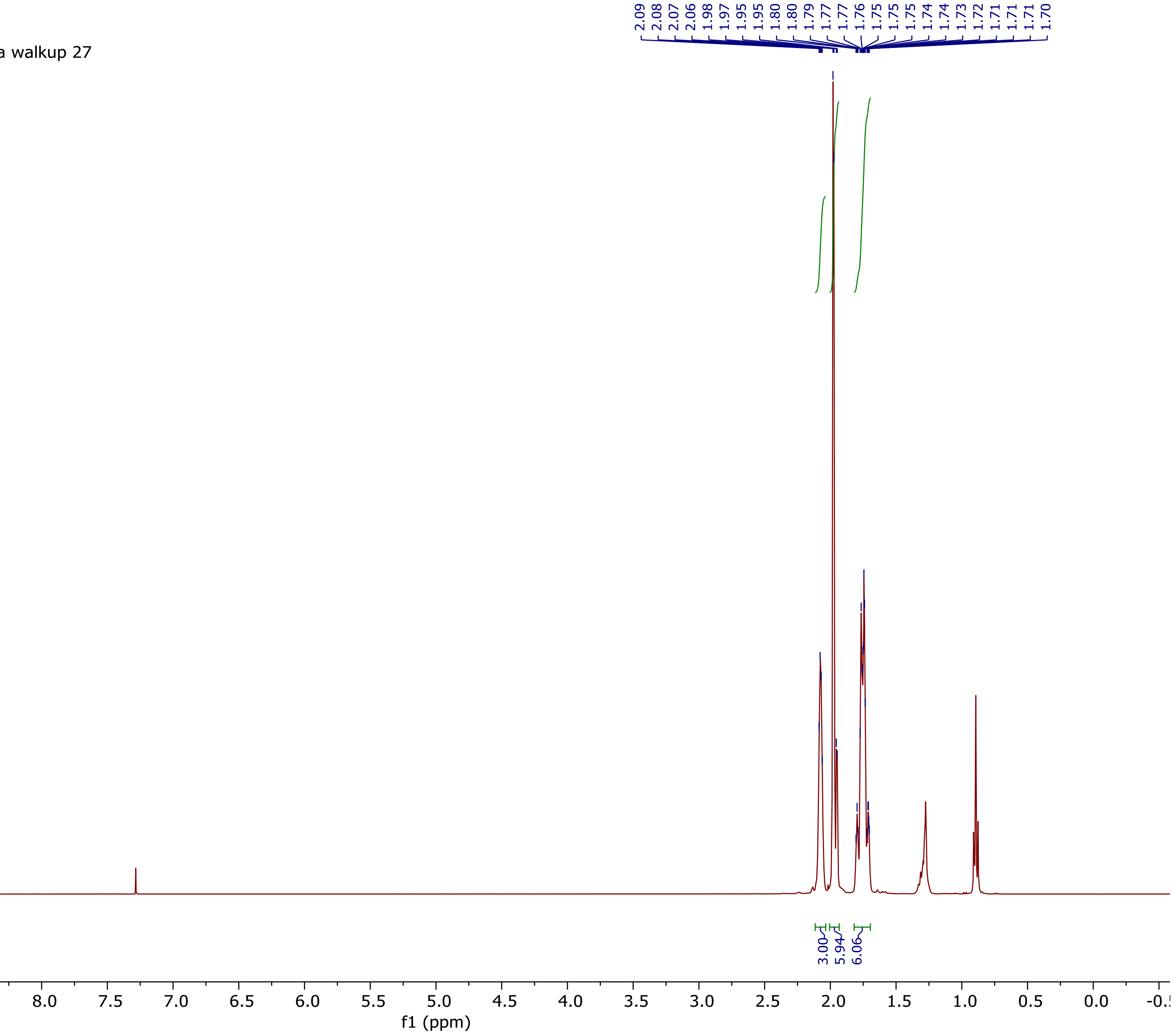

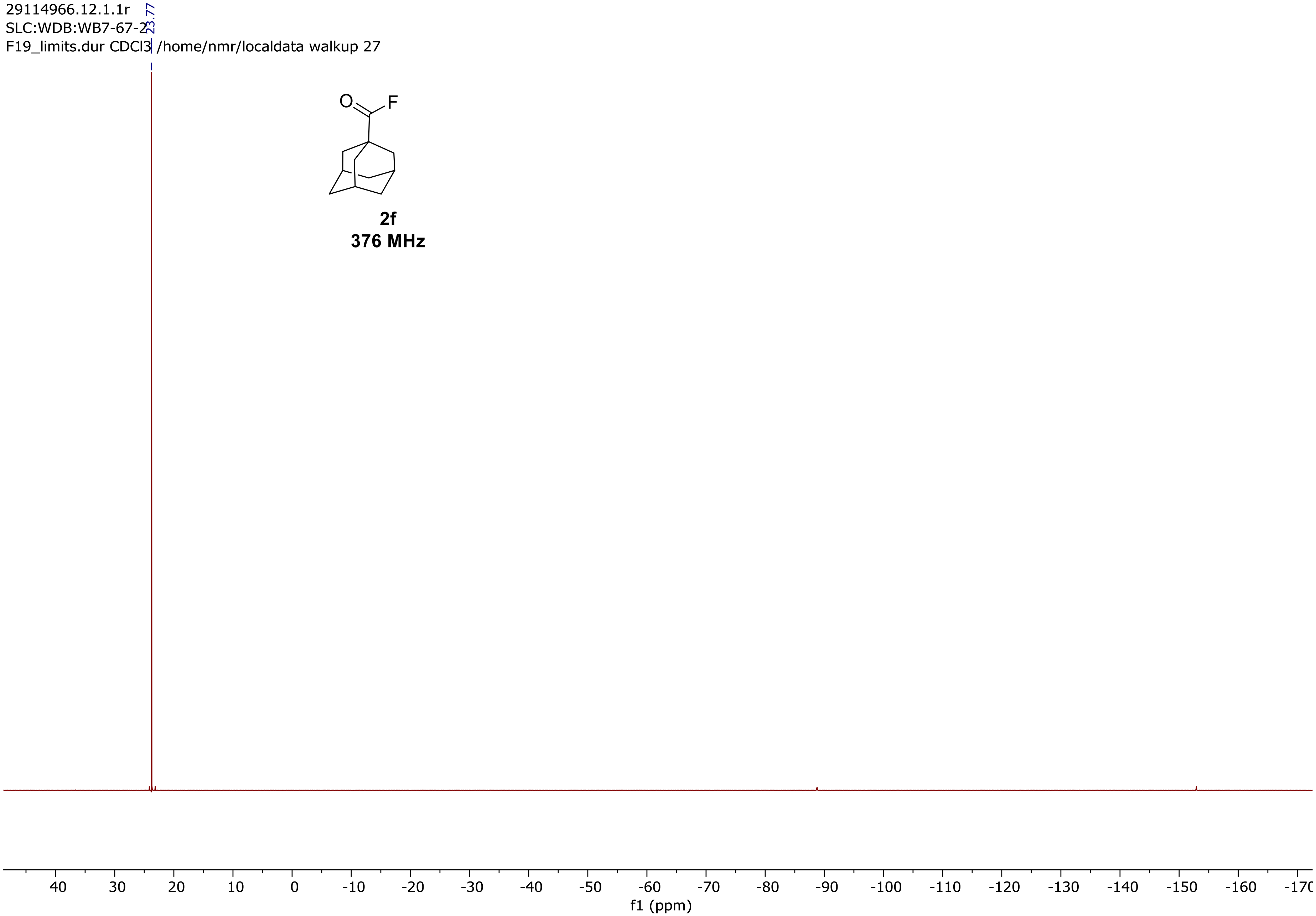
29114966.12.1.1r
SLC:WDB:WB7-67-2
F19_limits.dur CDCl3 /home/nmr/localdata walkup 27
23.77
O
F
2f
376 MHz
40 30 20 10 0 -10 -20 -30 -40 -50 -60 -70 -80 -90 -100 -110 -120 -130 -140 -150 -160 -170
f1 (ppm)

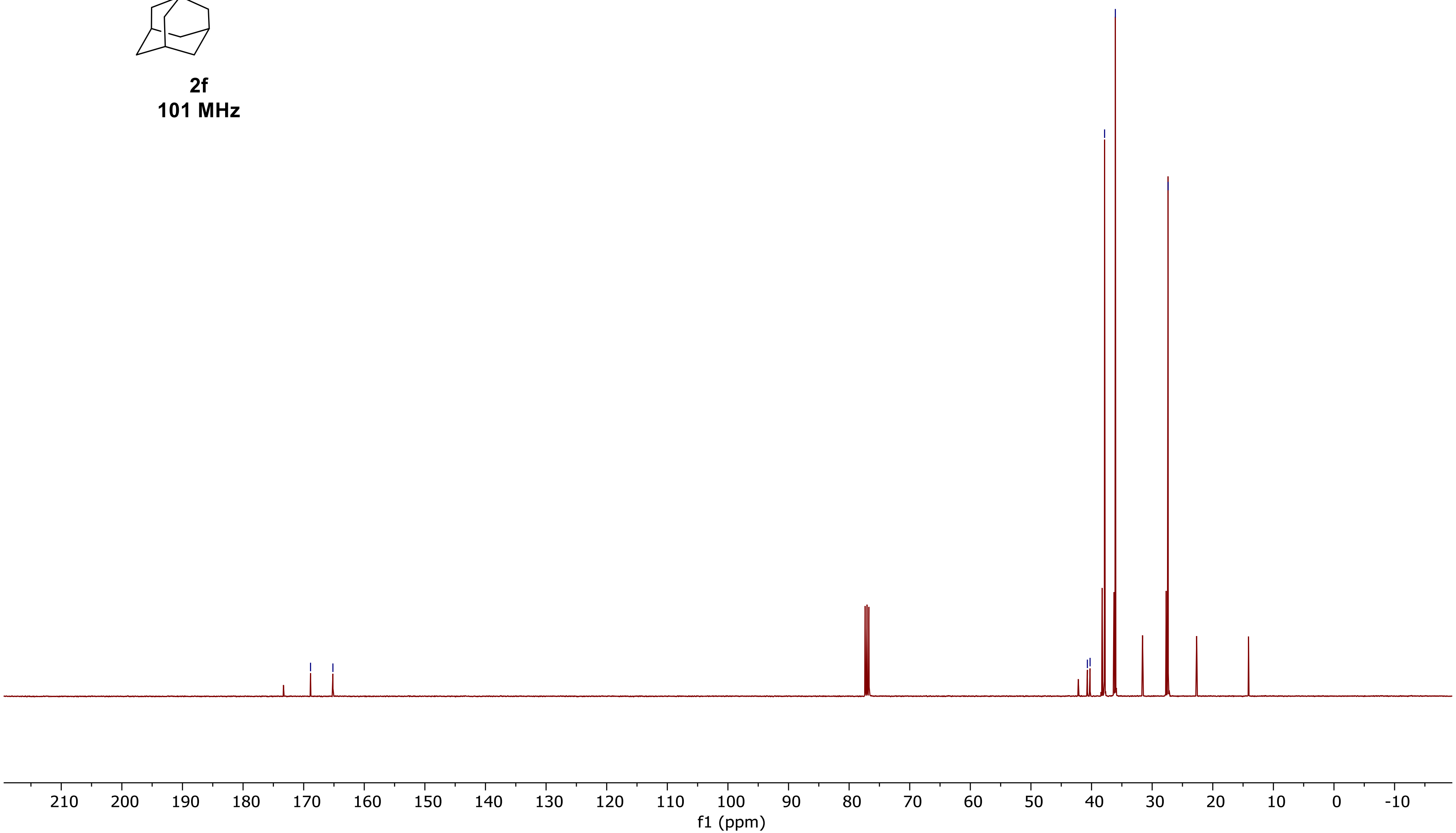
29114966.14.fid
SLC:WDB:WB7-67-2
Carbon.dur CDCl3 /home/nmr/localdata walkup 27
168.87
165.17
40.69
40.24
37.85
36.07
27.37
210 200 190 180 170 160 150 140 130 120 110 100 90 80 70 60 50 40 30 20 10 0 -10
f1 (ppm)

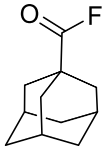
O
F

2f
101 MHz

29114660.10.1.1r
SLC:WDB:WB7-67-3
Proton.dur CDCl3 /home/nmr/localdata walkup 17

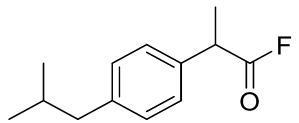

2g
400 MHz

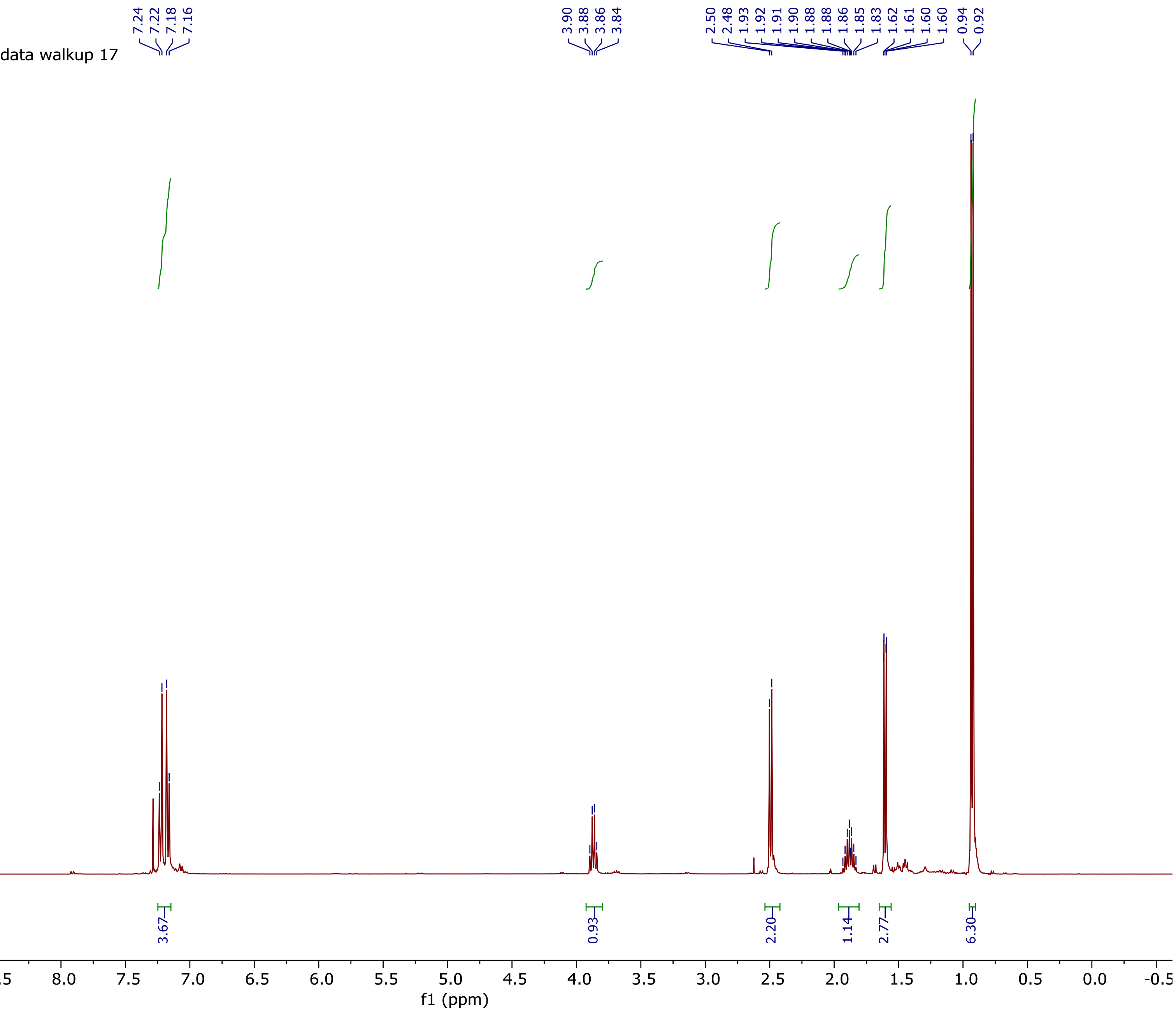

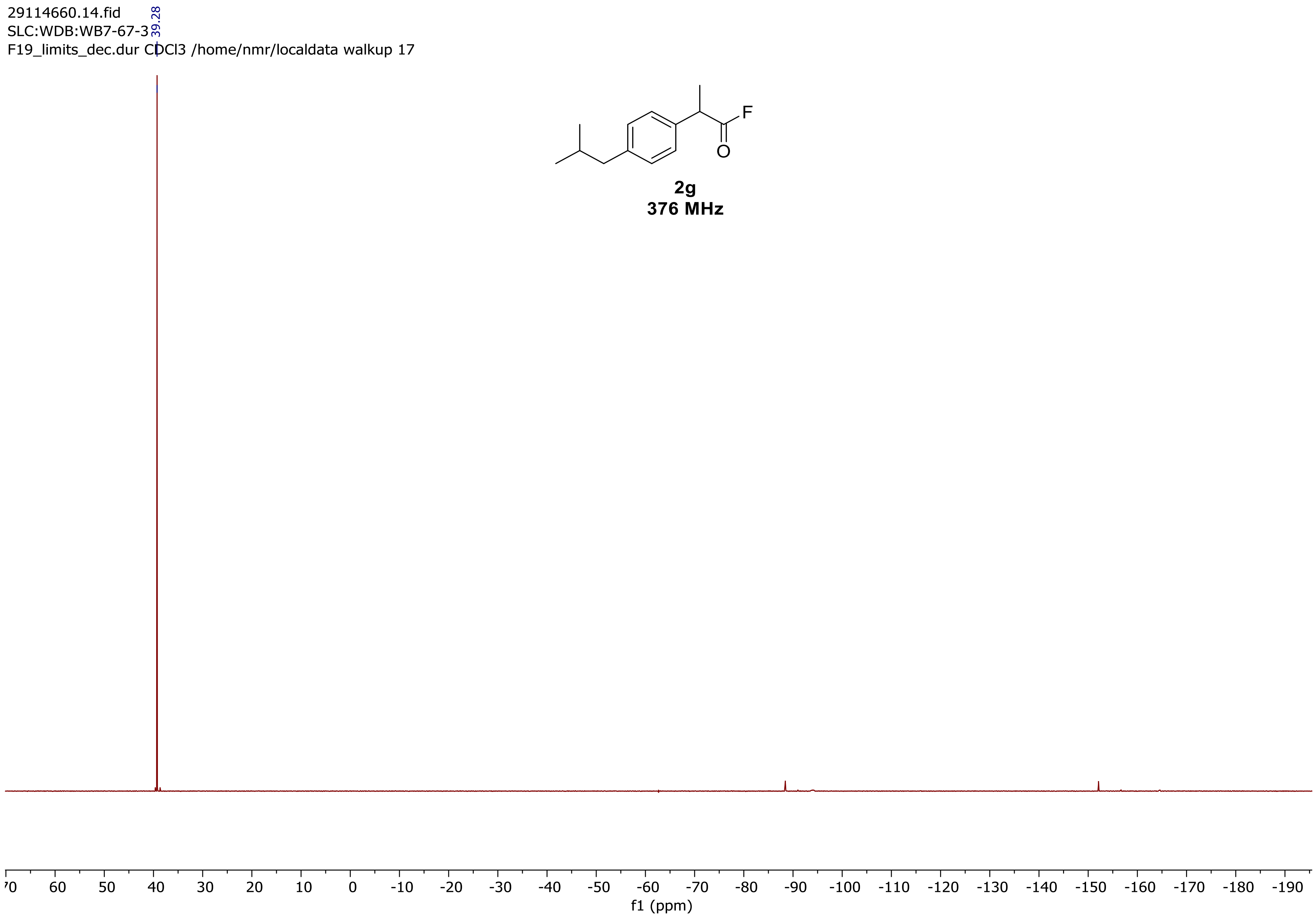
29114660.14.fid
SLC:WDB:WB7-67-3
F19_limits_dec.dur CDCl3 /home/nmr/localdata walkup 17
39.28
F
O
2g
376 MHz
70 60 50 40 30 20 10 0 -10 -20 -30 -40 -50 -60 -70 -80 -90 -100 -110 -120 -130 -140 -150 -160 -170 -180 -190
f1 (ppm)

29114660.11.fid
SLC:WDB:WB7-67-3
Carbon.dur CDCl3 /home/nmr/localdata walkup 17

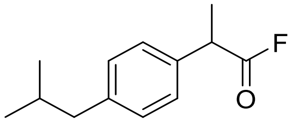

2g
101 MHz

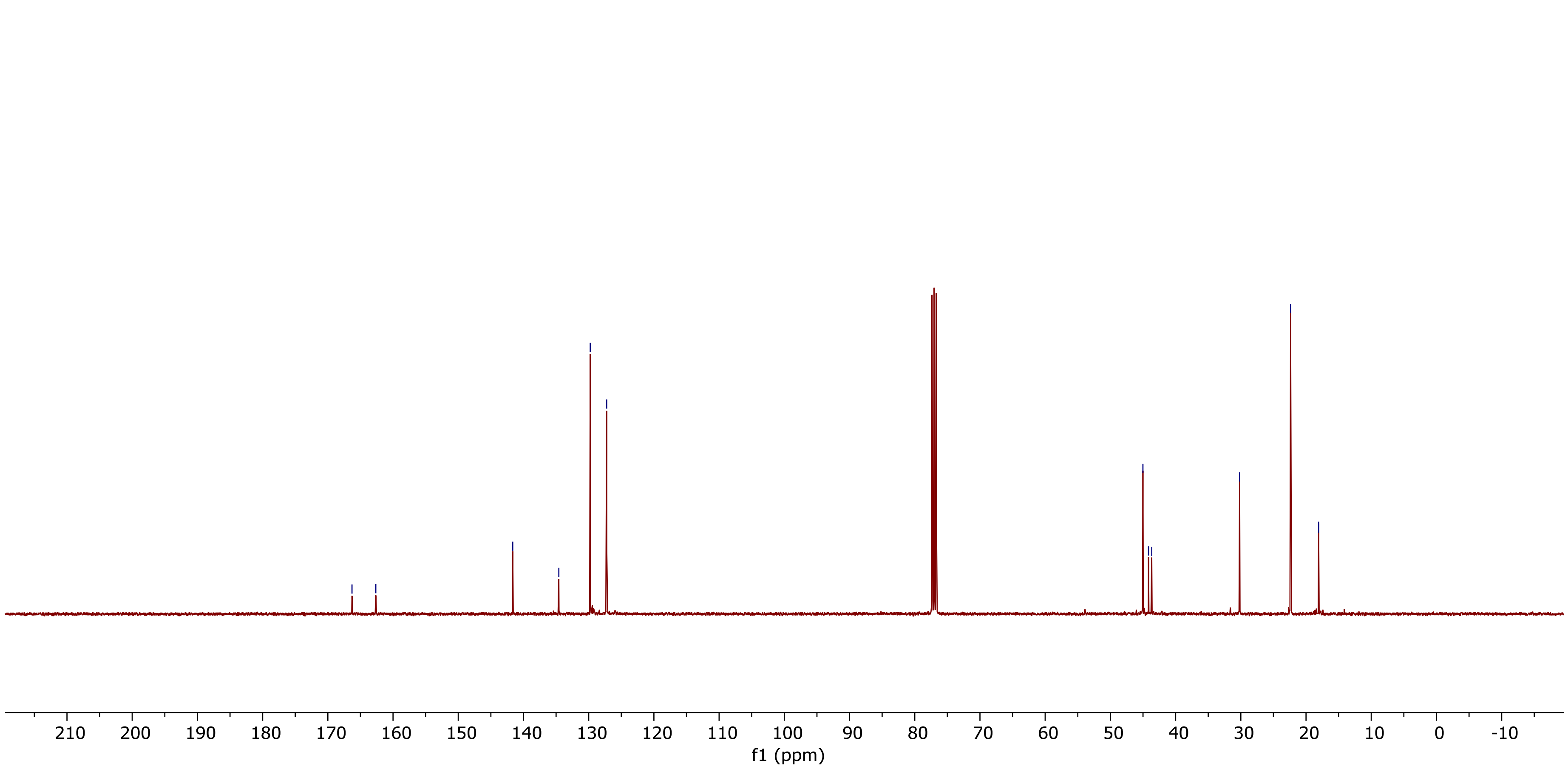

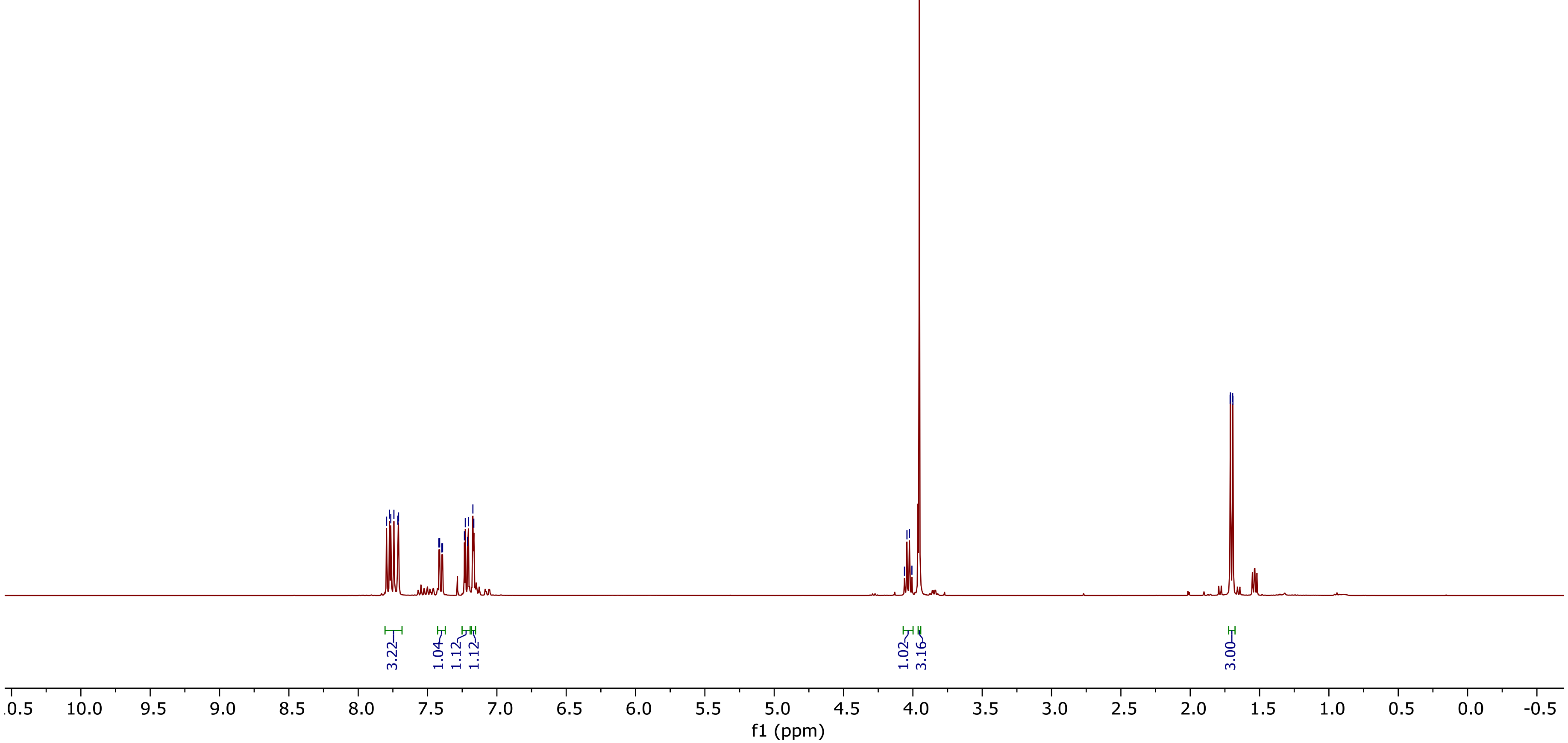
29114663.10.1.1r
SLC:WDB:WB7-67-4
Proton.dur CDCl3 /home/nmr/localdata walkup 20
7.80 7.77 7.76 7.74 7.71 7.71 7.42 7.41 7.40 7.39 7.23 7.23 7.21 7.21 7.17 7.17
4.06 4.04 4.02 4.01 3.95
1.71 1.71 1.69 1.69
3.22 1.04 1.12 1.12 1.02 3.16 3.00
f1 (ppm)

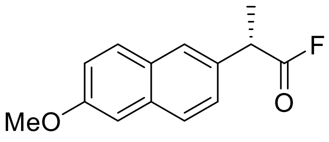
MeO
F
O

2h
400 MHz

29114663.14.fid
SLC:WDB:WB7-67-4
F19_limits_dec.dur CDCl3 /home/nmr/localdata walkup 20

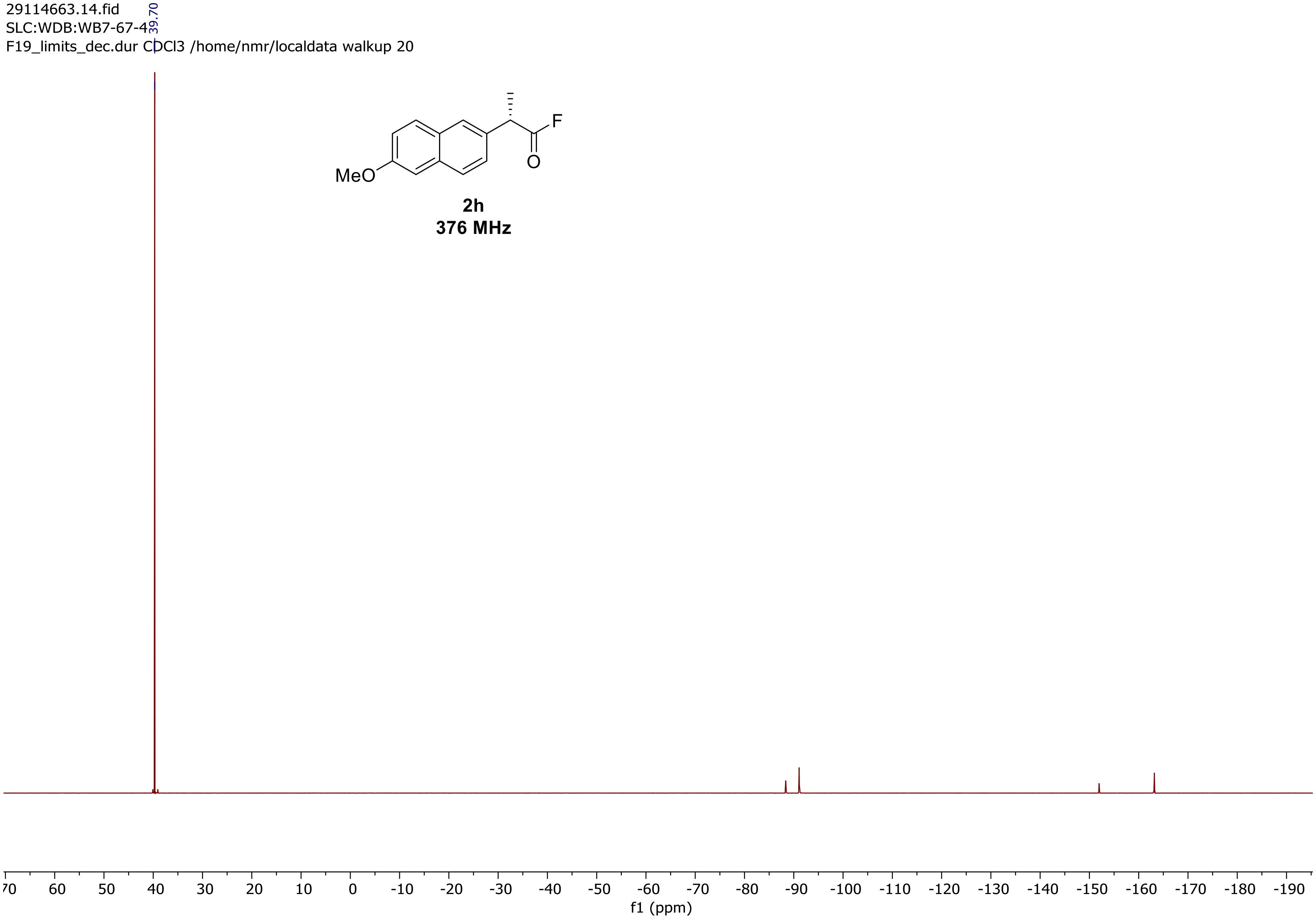

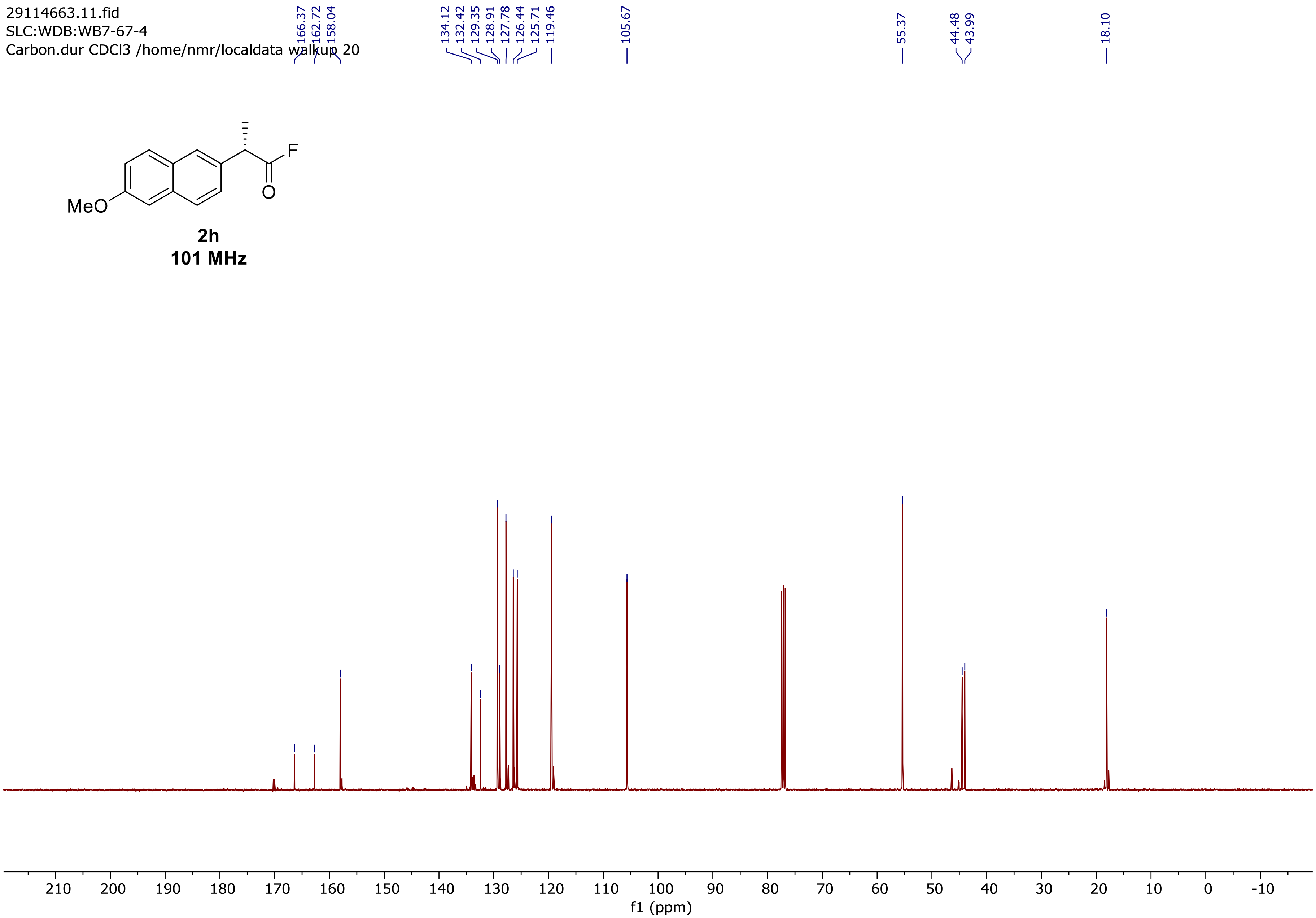
29114663.11.fid
SLC:WDB:WB7-67-4
Carbon.dur CDCl3 /home/nmr/localdata walkup 20
166.37
162.72
158.04
134.12
132.42
129.35
128.91
127.78
126.44
125.71
119.46
105.67
55.37
44.48
43.99
18.10
MeO
F
O
2h
101 MHz
210 200 190 180 170 160 150 140 130 120 110 100 90 80 70 60 50 40 30 20 10 0 -10
f1 (ppm)

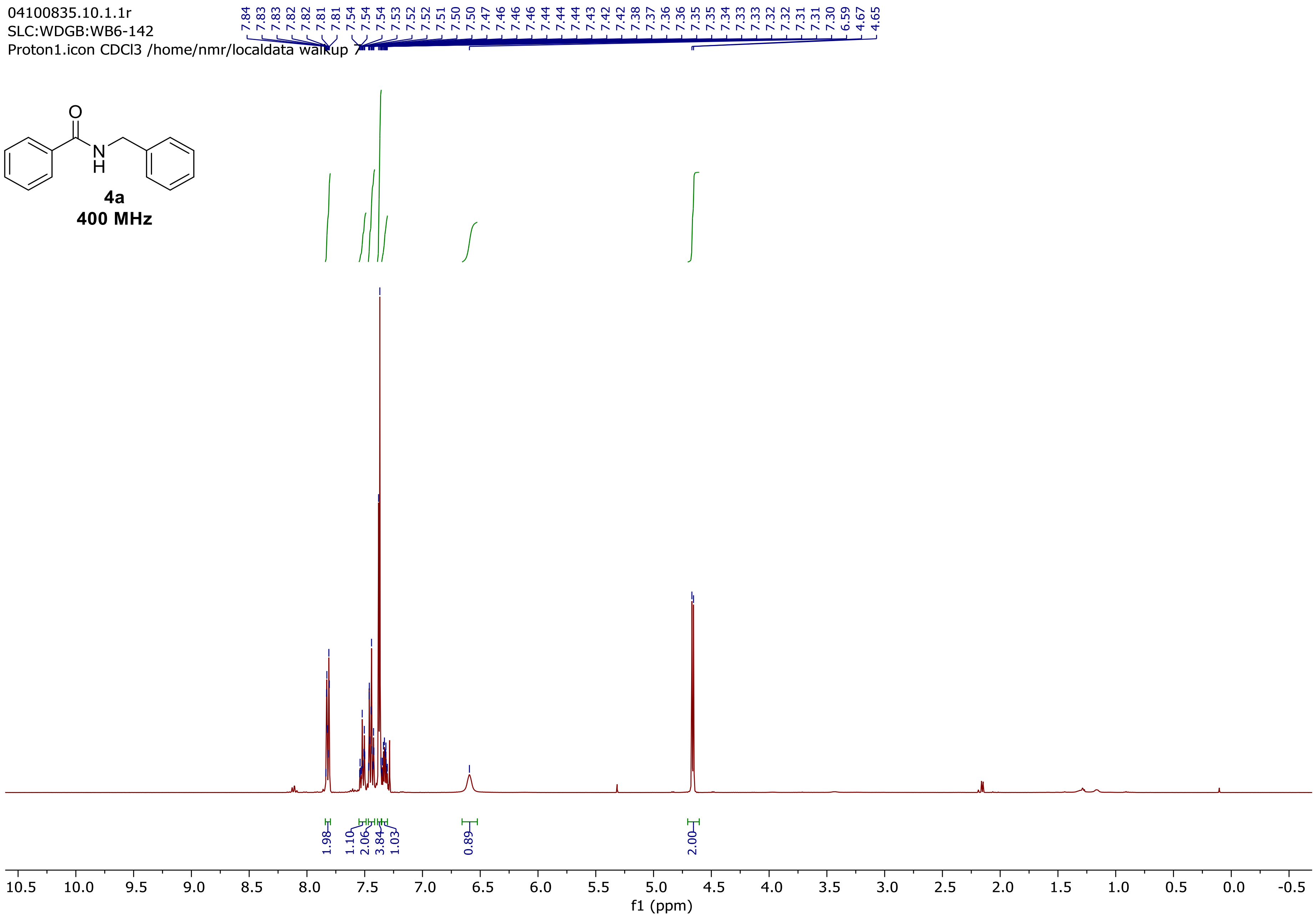
04100835.10.1.1r
SLC:WDGB:WB6-142
Proton1.icon CDCl3 /home/nmr/localdata walkup 7
7.84 7.83 7.83 7.82 7.82 7.81 7.81 7.54 7.54 7.54 7.53 7.52 7.52 7.51 7.50 7.50 7.47 7.46 7.46 7.44 7.44 7.44 7.43 7.42 7.42 7.38 7.37 7.36 7.36 7.35 7.35 7.34 7.33 7.33 7.32 7.32 7.31 7.31 7.30 6.59 4.67 4.65
O
N
H
4a
400 MHz
1.98
1.10
2.06
3.84
1.03
0.89
2.00
10.5 10.0 9.5 9.0 8.5 8.0 7.5 7.0 6.5 6.0 5.5 5.0 4.5 4.0 3.5 3.0 2.5 2.0 1.5 1.0 0.5 0.0 -0.5
f1 (ppm)

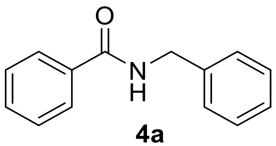

4a
101 MHz

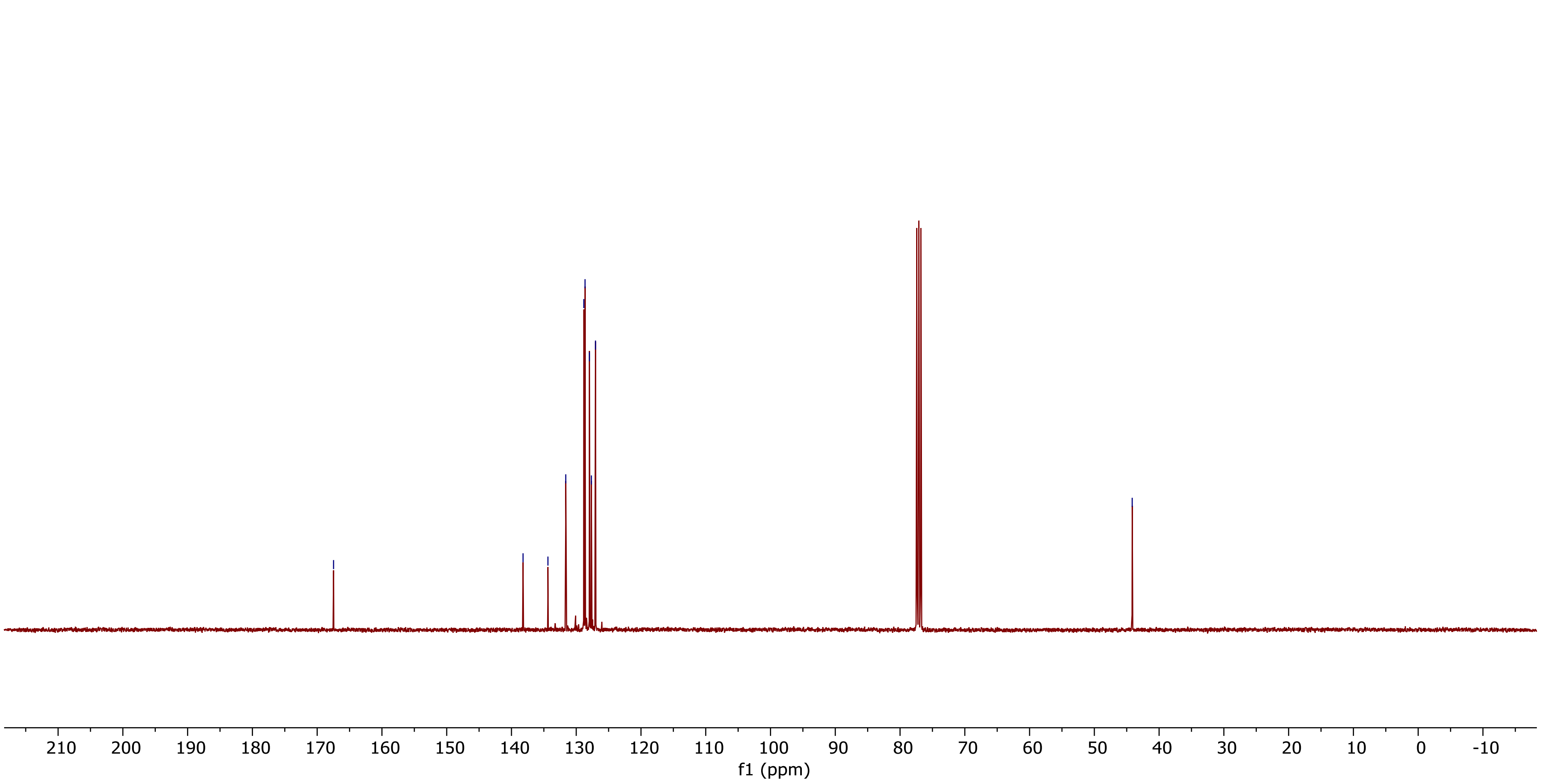
04100835.11.1.1r
SLC:WDGB:WB6-142
Carbon.dur CDCl3 /home/nmr/localdata walkup 7
167.47
138.21
134.37
131.61
128.83
128.64
127.96
127.66
127.03
44.15
210 200 190 180 170 160 150 140 130 120 110 100 90 80 70 60 50 40 30 20 10 0 -10
f1 (ppm)

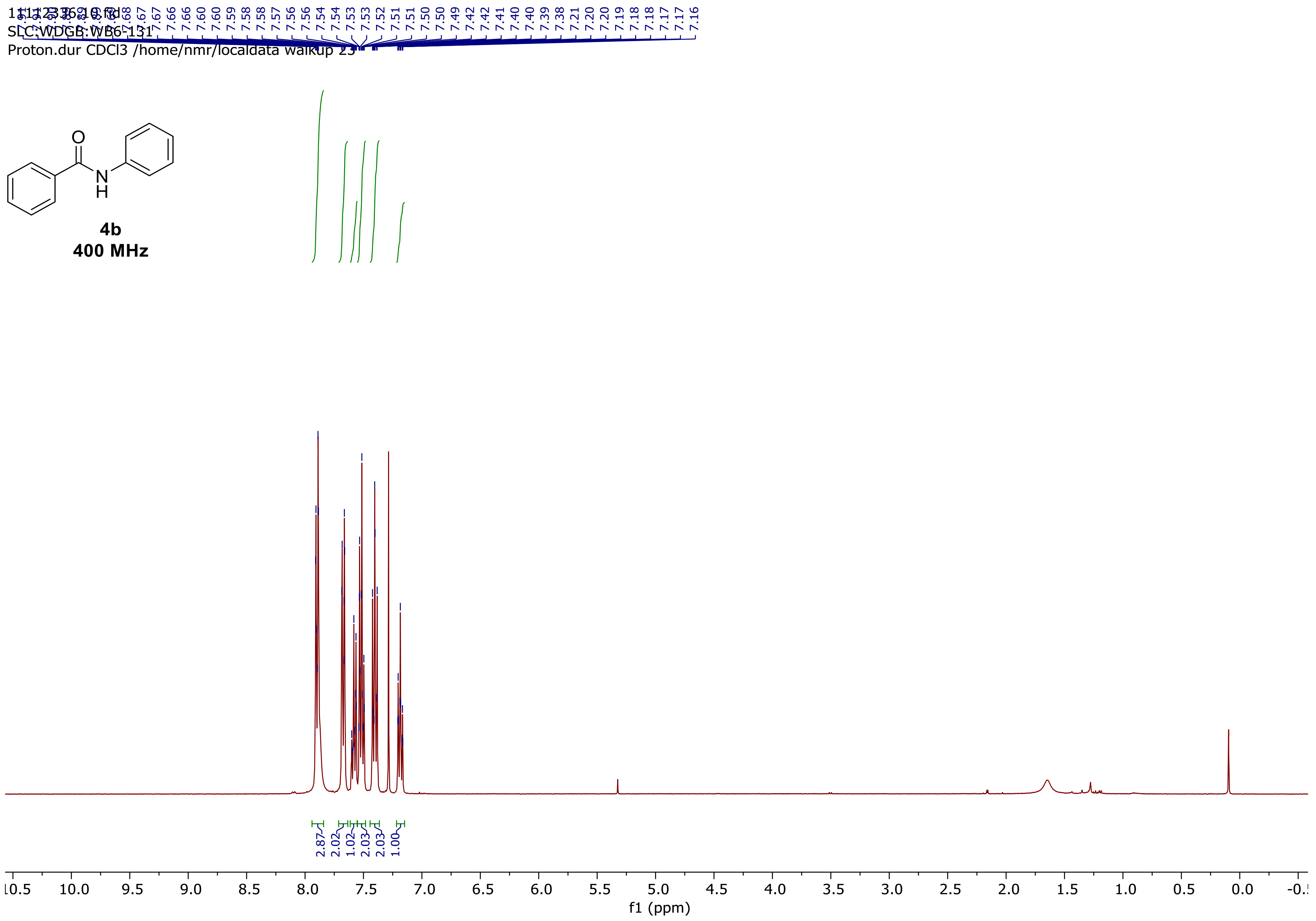
SLC.WDGB.WB6-131
Proton.dur CDCl3 /home/nmr/localdata walkup 23
7.68 7.67 7.67 7.66 7.66 7.60 7.60 7.59 7.58 7.58 7.57 7.56 7.56 7.54 7.54 7.53 7.53 7.52 7.51 7.51 7.50 7.49 7.42 7.42 7.41 7.40 7.40 7.39 7.38 7.21 7.20 7.20 7.19 7.18 7.18 7.17 7.17 7.16
O
N
H
4b
400 MHz
2.87
2.02
1.02
2.03
2.03
1.00
10.5 10.0 9.5 9.0 8.5 8.0 7.5 7.0 6.5 6.0 5.5 5.0 4.5 4.0 3.5 3.0 2.5 2.0 1.5 1.0 0.5 0.0
f1 (ppm)

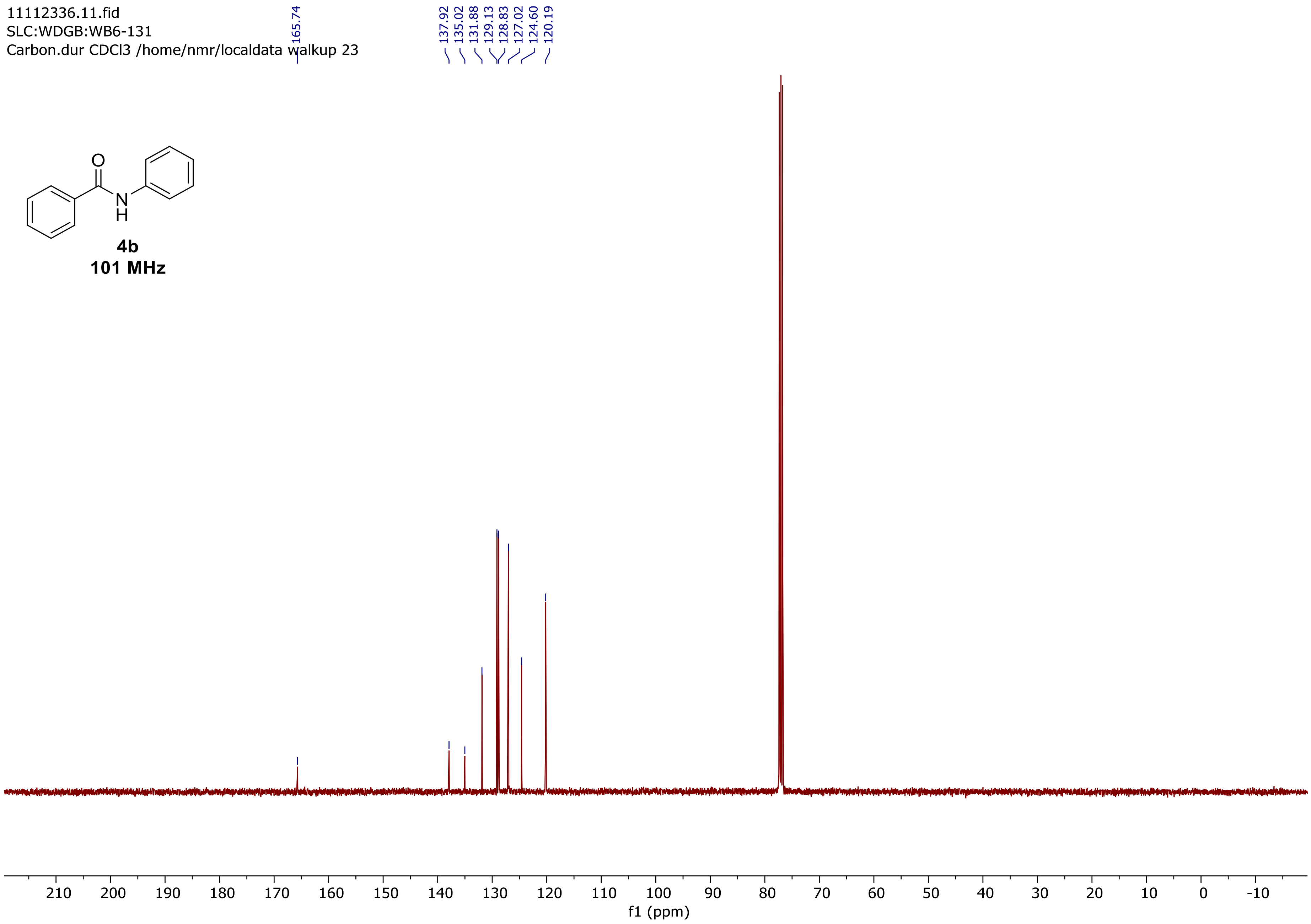
11112336.11.fid
SLC:WDGB:WB6-131
Carbon.dur CDCl3 /home/nmr/localdata walkup 23
165.74
137.92
135.02
131.88
129.13
128.83
127.02
124.60
120.19
O
N
H
4b
101 MHz
210 200 190 180 170 160 150 140 130 120 110 100 90 80 70 60 50 40 30 20 10 0 -10
f1 (ppm)

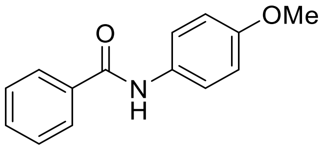
OMe
O
N
H

4c
400 MHz

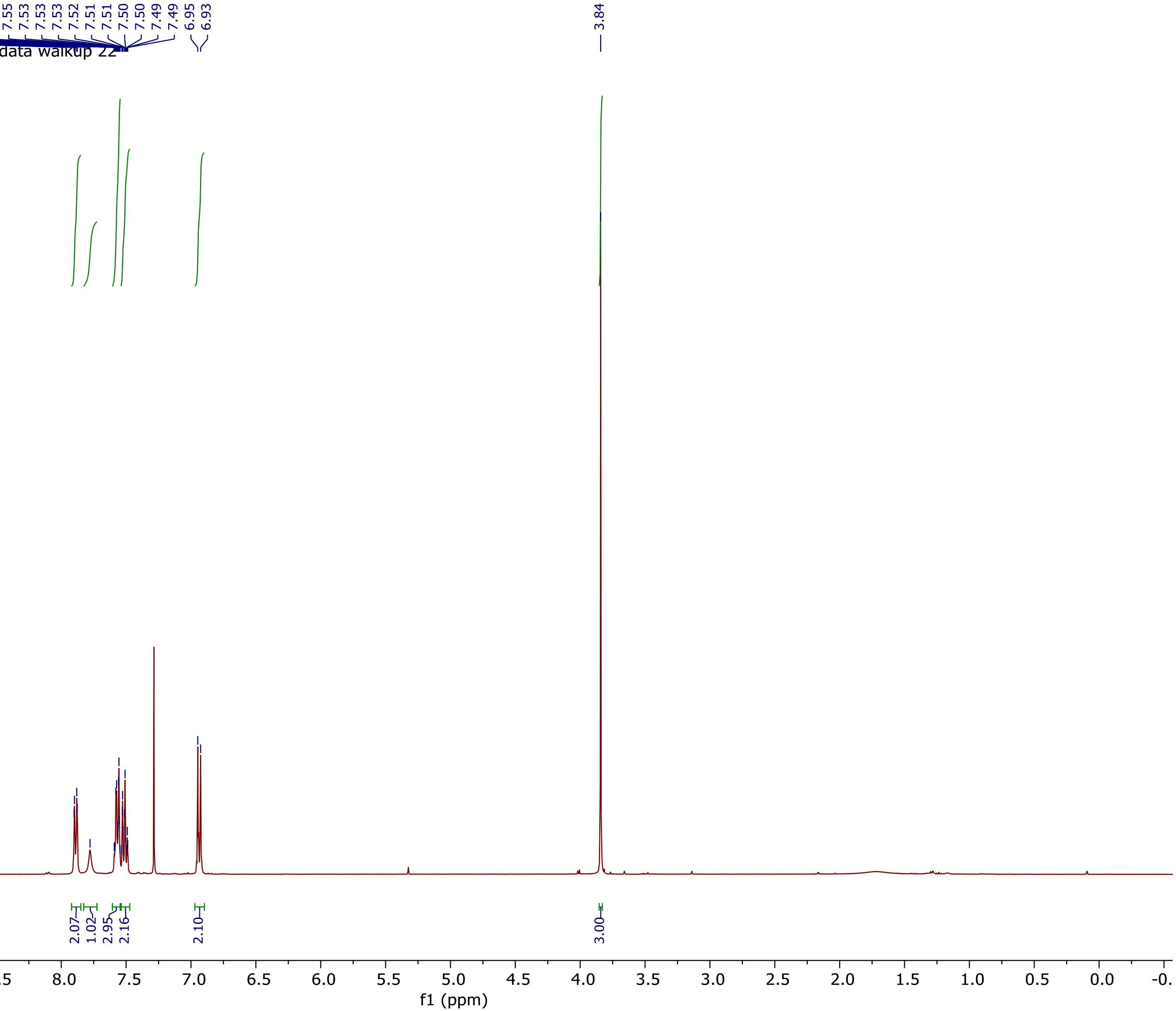
SLC:WDGB:WB7-4-3
Proton.dur CDCl3 /home/nmr/localdata walkup 22
7.59
7.58
7.57
7.57
7.56
7.56
7.56
7.55
7.55
7.53
7.53
7.53
7.52
7.51
7.51
7.50
7.50
7.49
7.49
6.95
6.93
3.84
2.07
1.02
2.95
2.16
2.10
3.00
10.5
10.0
9.5
9.0
8.5
8.0
7.5
7.0
6.5
6.0
5.5
5.0
4.5
4.0
3.5
3.0
2.5
2.0
1.5
1.0
0.5
0.0
f1 (ppm)

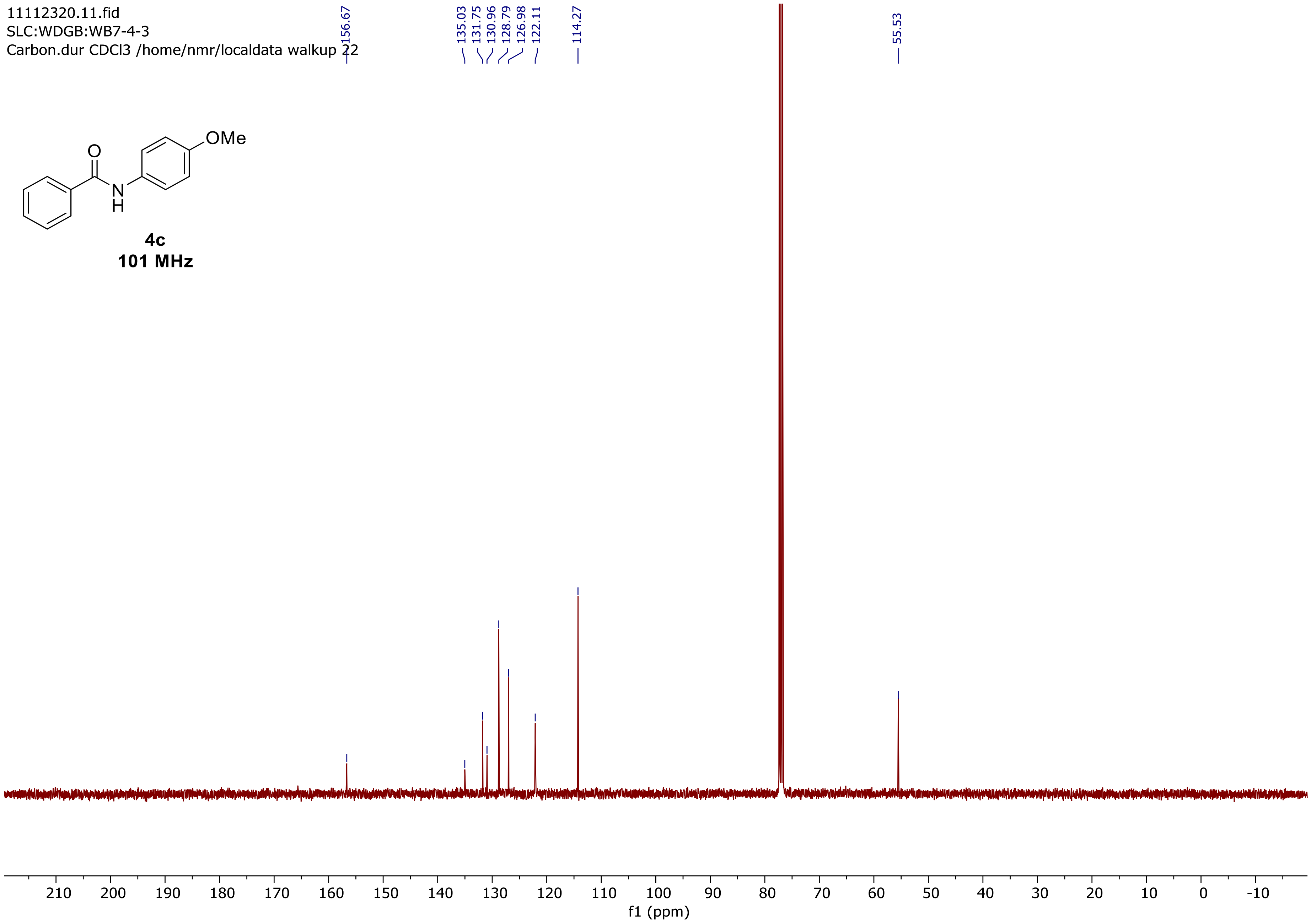
11112320.11.fid
SLC:WDGB:WB7-4-3
Carbon.dur CDCl3 /home/nmr/localdata walkup 22
156.67
135.03
131.75
130.96
128.79
126.98
122.11
114.27
55.53
OMe
O
N
H
4c
101 MHz
210 200 190 180 170 160 150 140 130 120 110 100 90 80 70 60 50 40 30 20 10 0 -10
f1 (ppm)

11112353.10.fid
SLC:WDGB:WB7-4-4
Proton.dur CDCl3 /home/nmr/localdata walkup 24

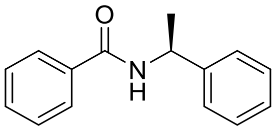

4d
400 MHz

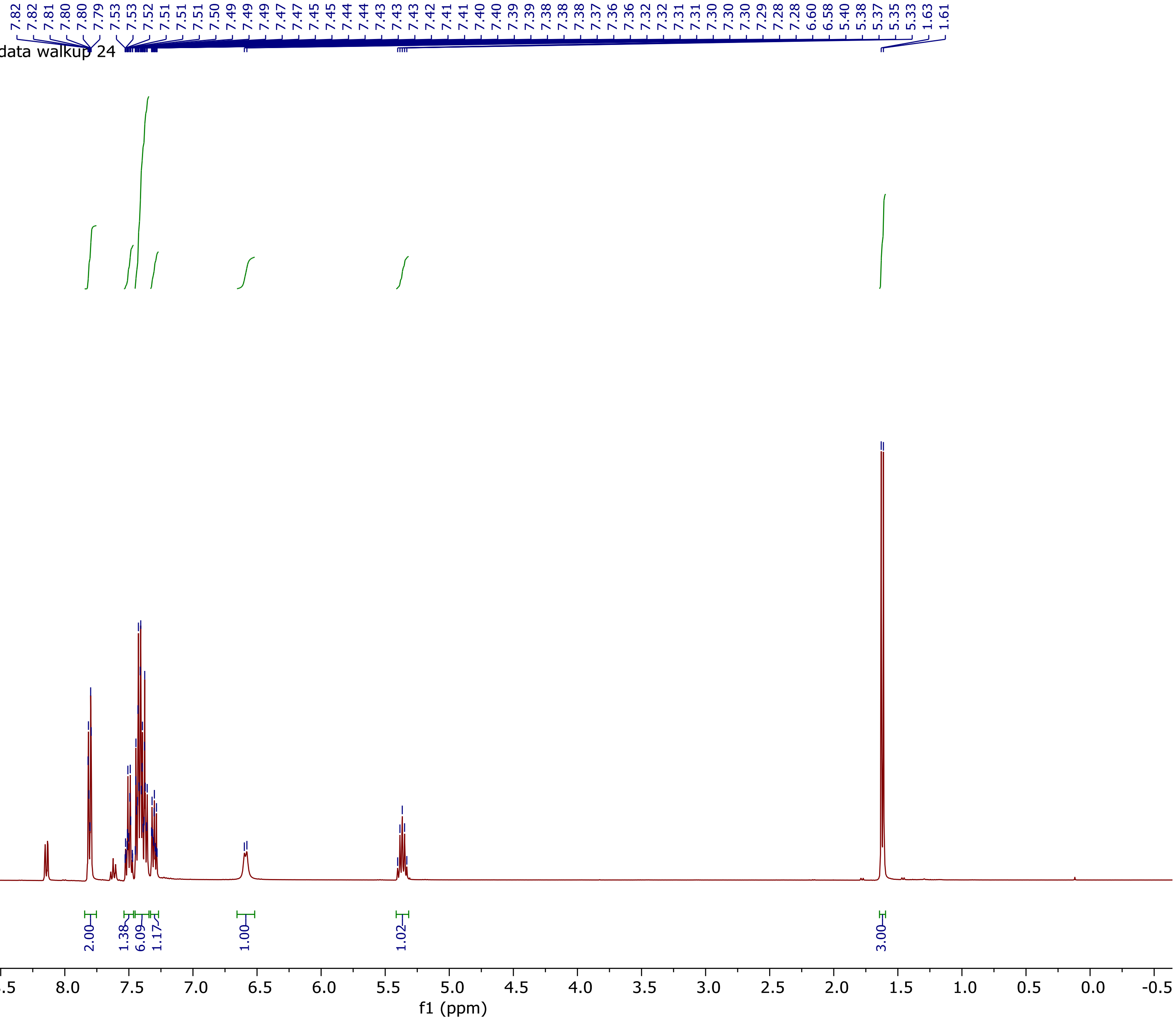

11112353.11.fid
SLC:WDGB:WB7-4-4
Carbon.dur CDCl3 /home/nmr/localdata walkup 24

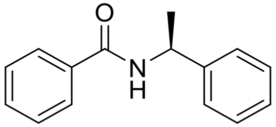

4d
101 MHz

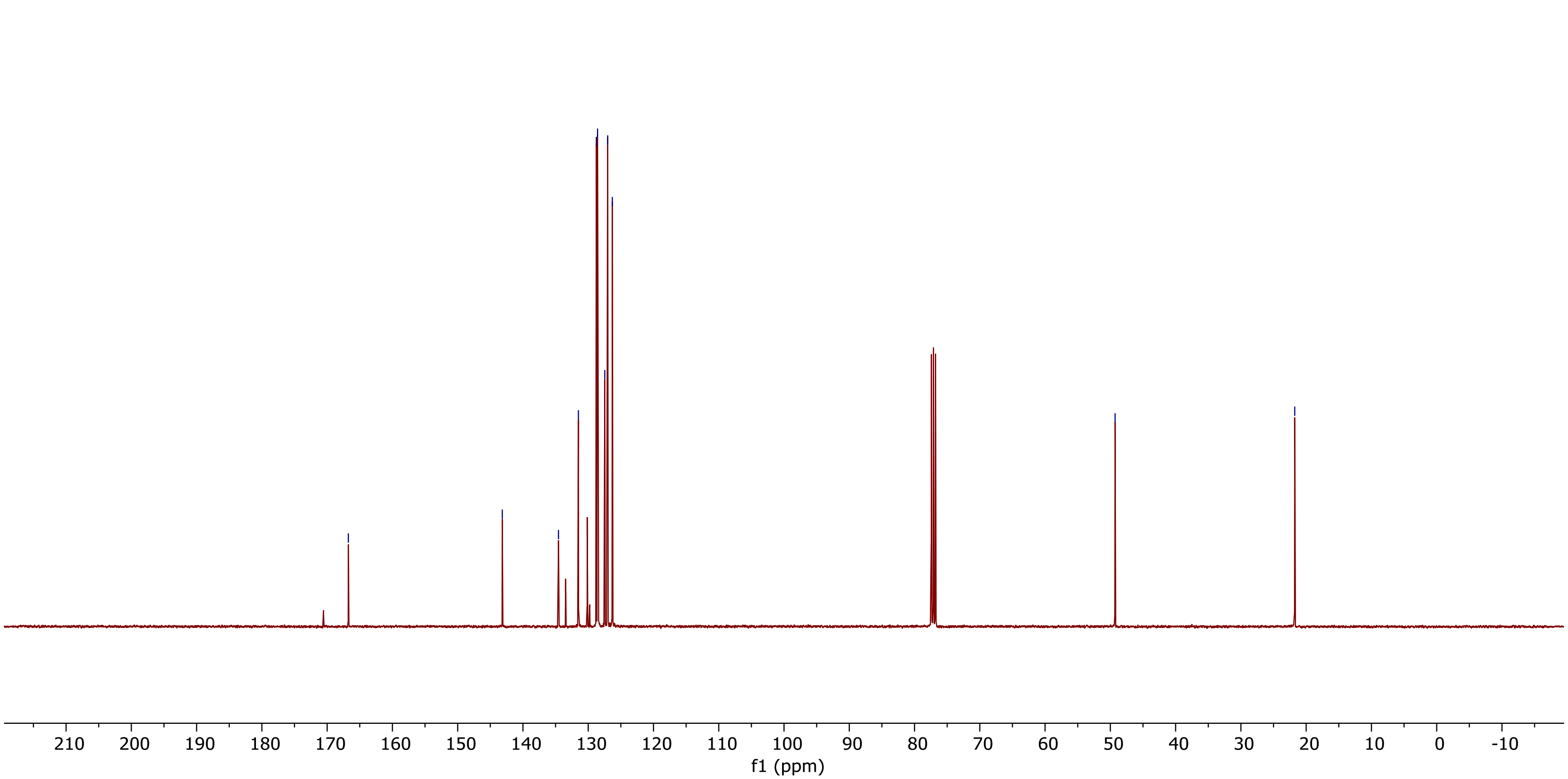

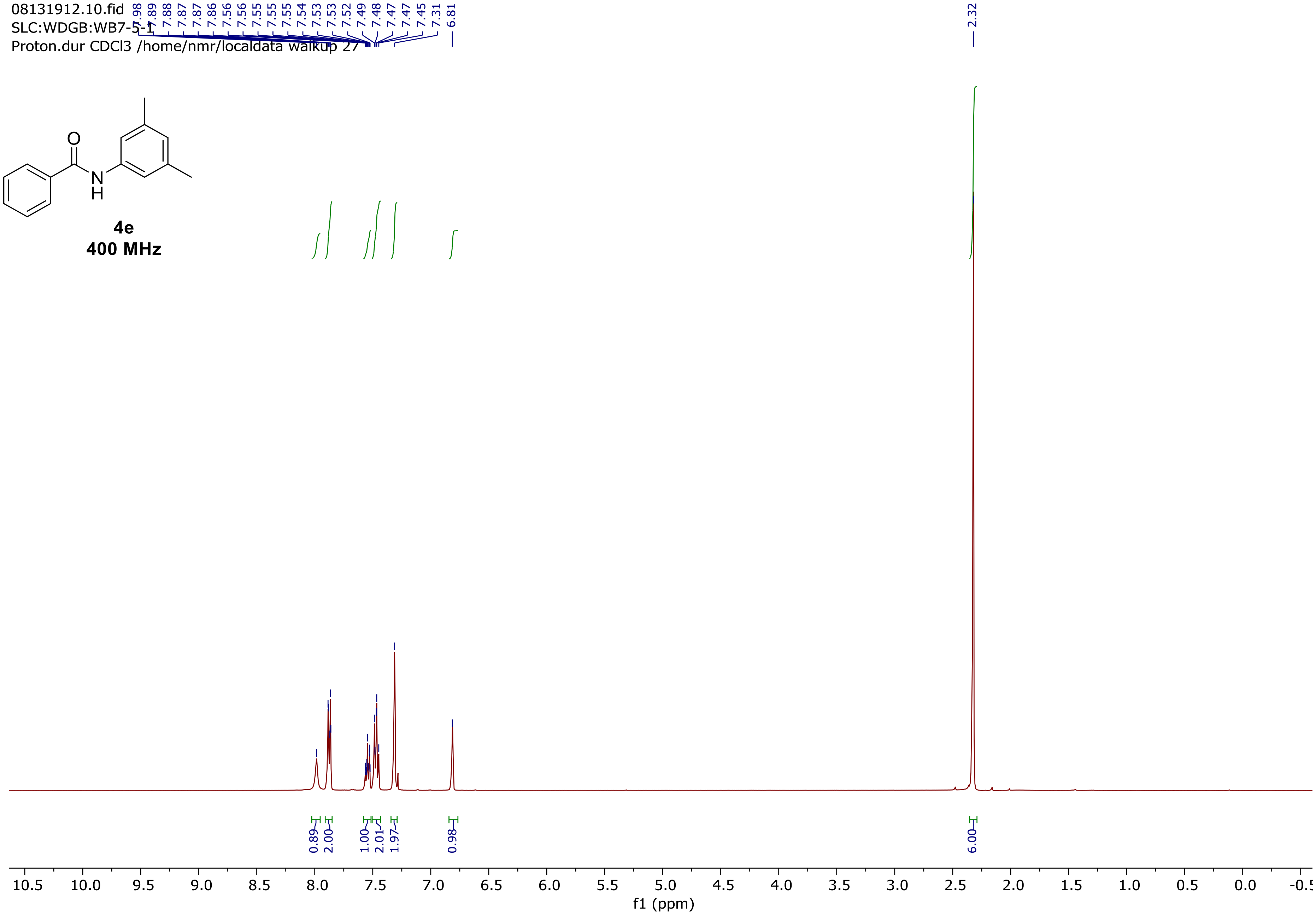
08131912.10.fid
SLC:WDGB:WB7-5-1
Proton.dur CDCl3 /home/nmr/localdata walkup 27
7.98
7.89
7.88
7.87
7.87
7.86
7.56
7.56
7.55
7.55
7.54
7.53
7.53
7.52
7.49
7.48
7.47
7.47
7.45
7.31
6.81
2.32
O
N
H
4e
400 MHz
0.89
2.00
1.00
2.01
1.97
0.98
6.00
10.5 10.0 9.5 9.0 8.5 8.0 7.5 7.0 6.5 6.0 5.5 5.0 4.5 4.0 3.5 3.0 2.5 2.0 1.5 1.0 0.5 0.0 -0.5
f1 (ppm)

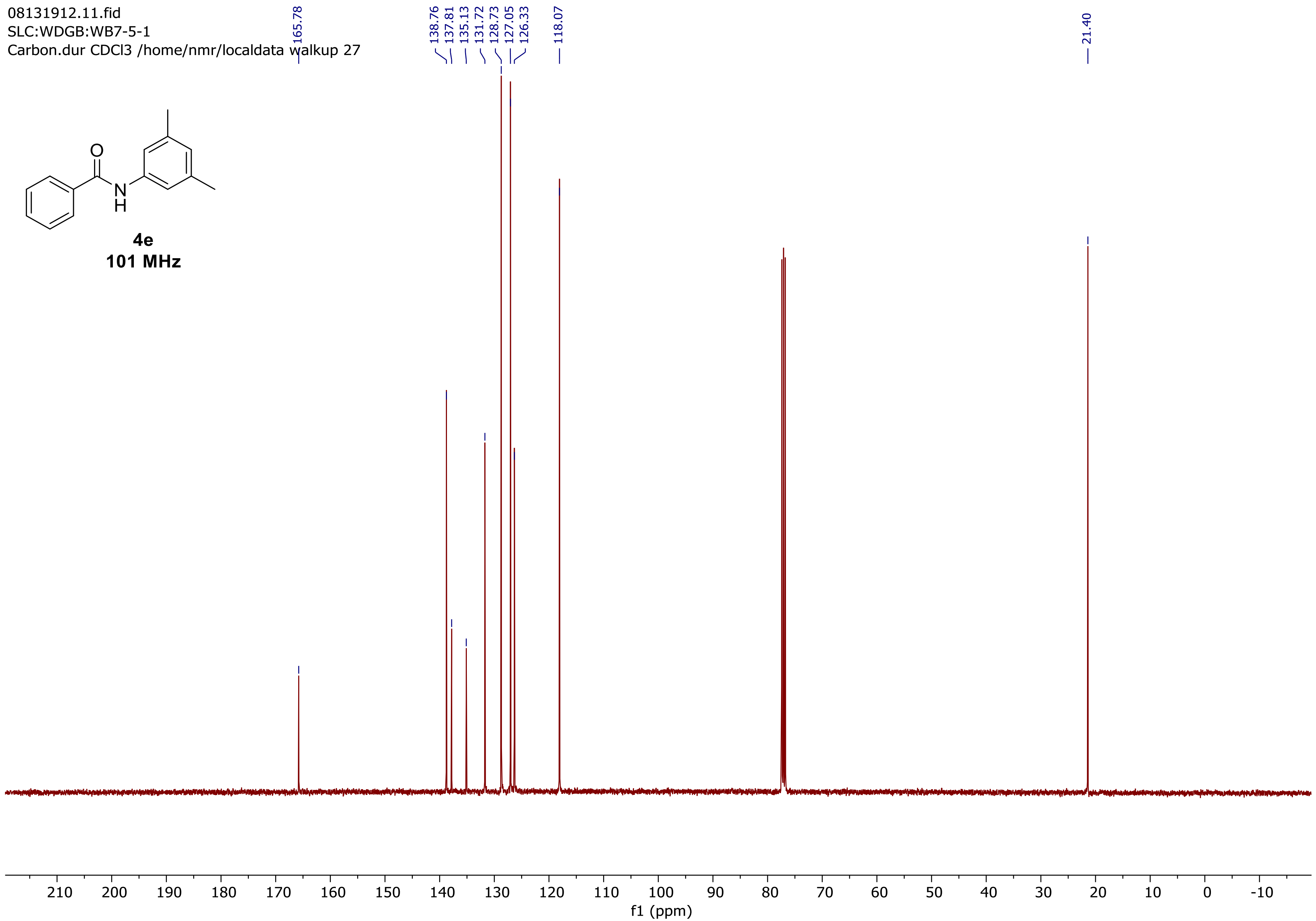

08131912.11.fid
SLC:WDGB:WB7-5-1
Carbon.dur CDCl3 /home/nmr/localdata walkup 27
165.78
138.76
137.81
135.13
131.72
128.73
127.05
126.33
118.07
21.40
O
N
H
4e
101 MHz
210 200 190 180 170 160 150 140 130 120 110 100 90 80 70 60 50 40 30 20 10 0 -10
f1 (ppm)

07120211.10.fid
SLC:WDGB:WB7-18
Proton.dur CDCl3 /home/nmr/localdata walkup 32

1H NMR (400 MHz, $CDCl_3$) δ 7.94 – 7.88 (m, 1H), 7.84 – 7.78 (m, 1H), 7.69 – 7.59 (m, 2H), 7.58 – 7.51 (m, 1H).

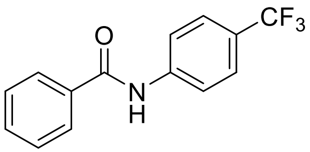

4f
400 MHz

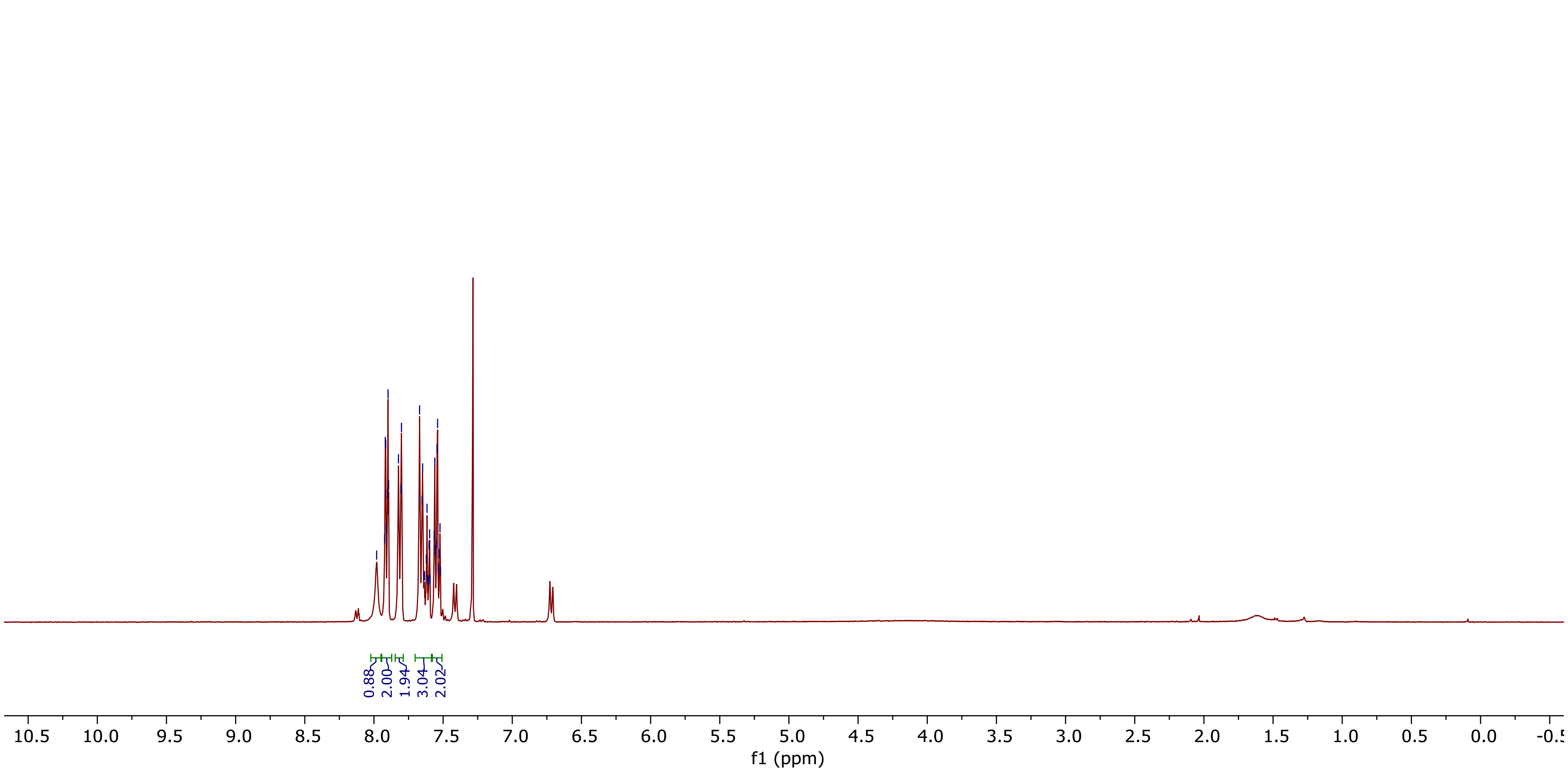

07120211.13.fid
SLC:WDGB:WB7-18
F19_limits_dec.dur CDCl3 /home/nmr/localdata walkup 32

4f
376 MHz

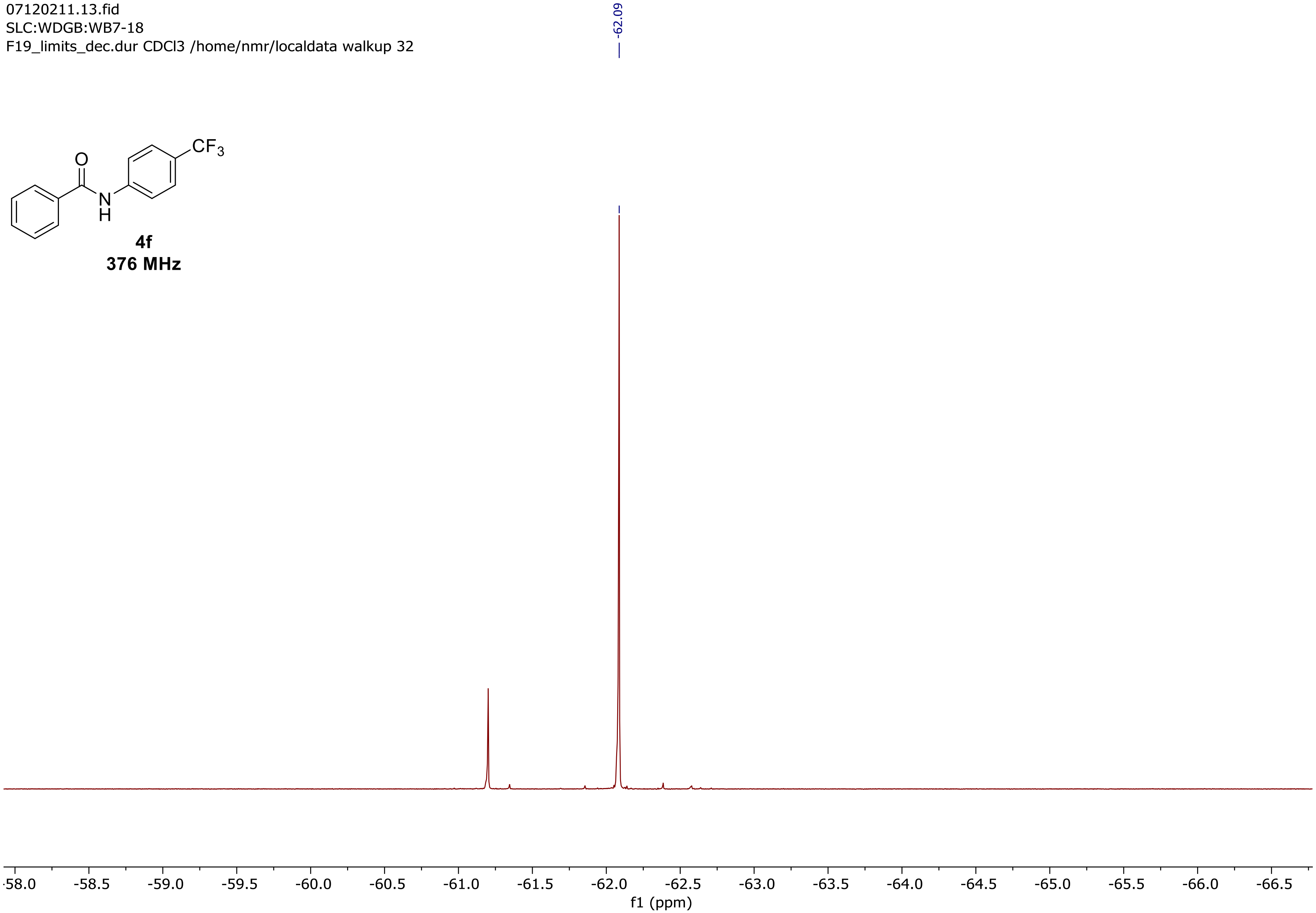

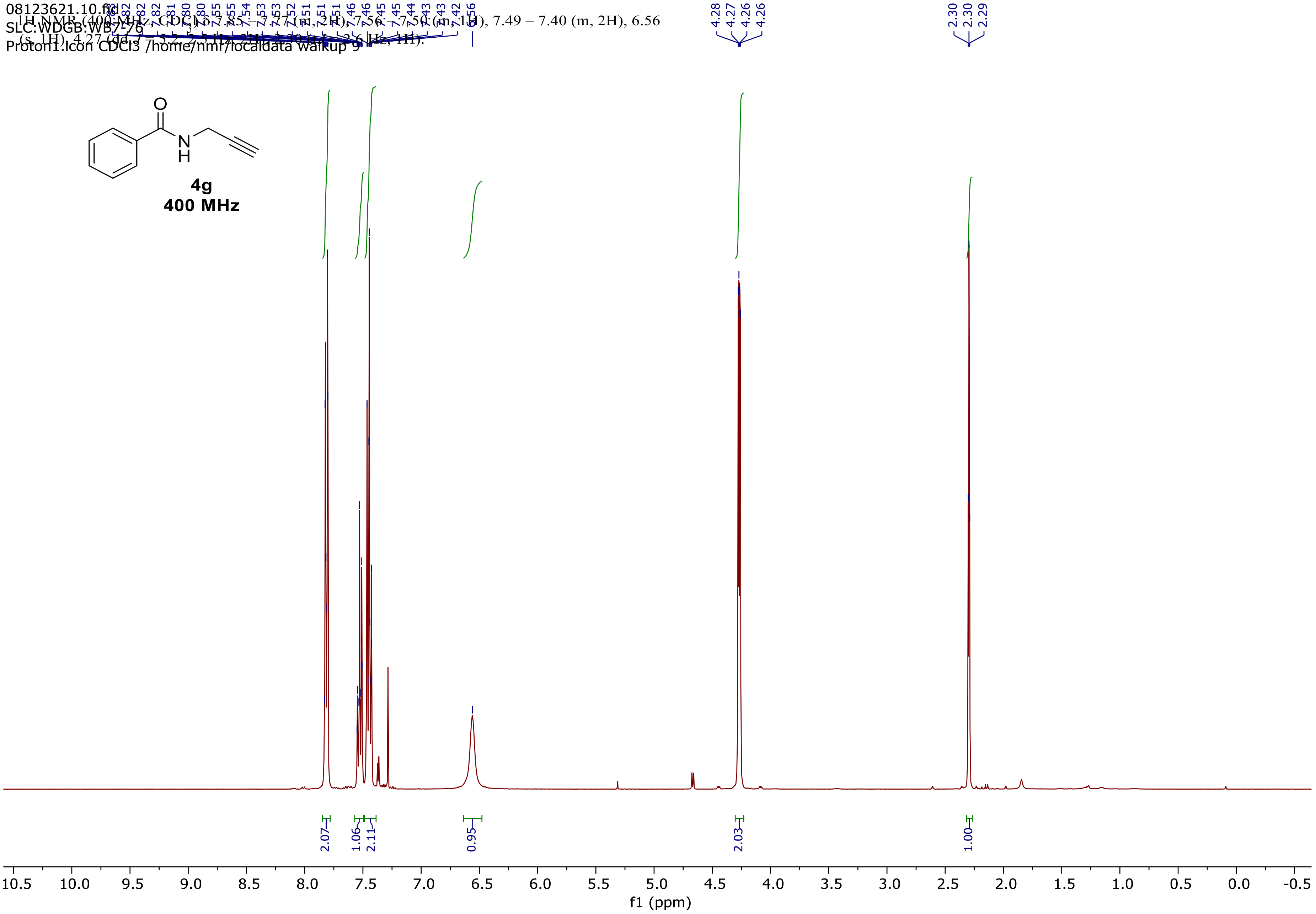
08123621.10.fid
SLC:WDGB:WB7-76
Proton1.icon CDCl3 /home/nmr/localdata walkup 9
1H NMR (400 MHz, $CDCl_3$) δ 7.85 – 7.77 (m, 2H), 7.56 – 7.50 (m, 1H), 7.49 – 7.40 (m, 2H), 6.56 (s, 1H), 4.27 (dd, J = 5.2, 2.5 Hz, 2H), 2.30 (t, J = 2.6 Hz, 1H).
4g
400 MHz
2.07
1.06
2.11
0.95
2.03
1.00
f1 (ppm)

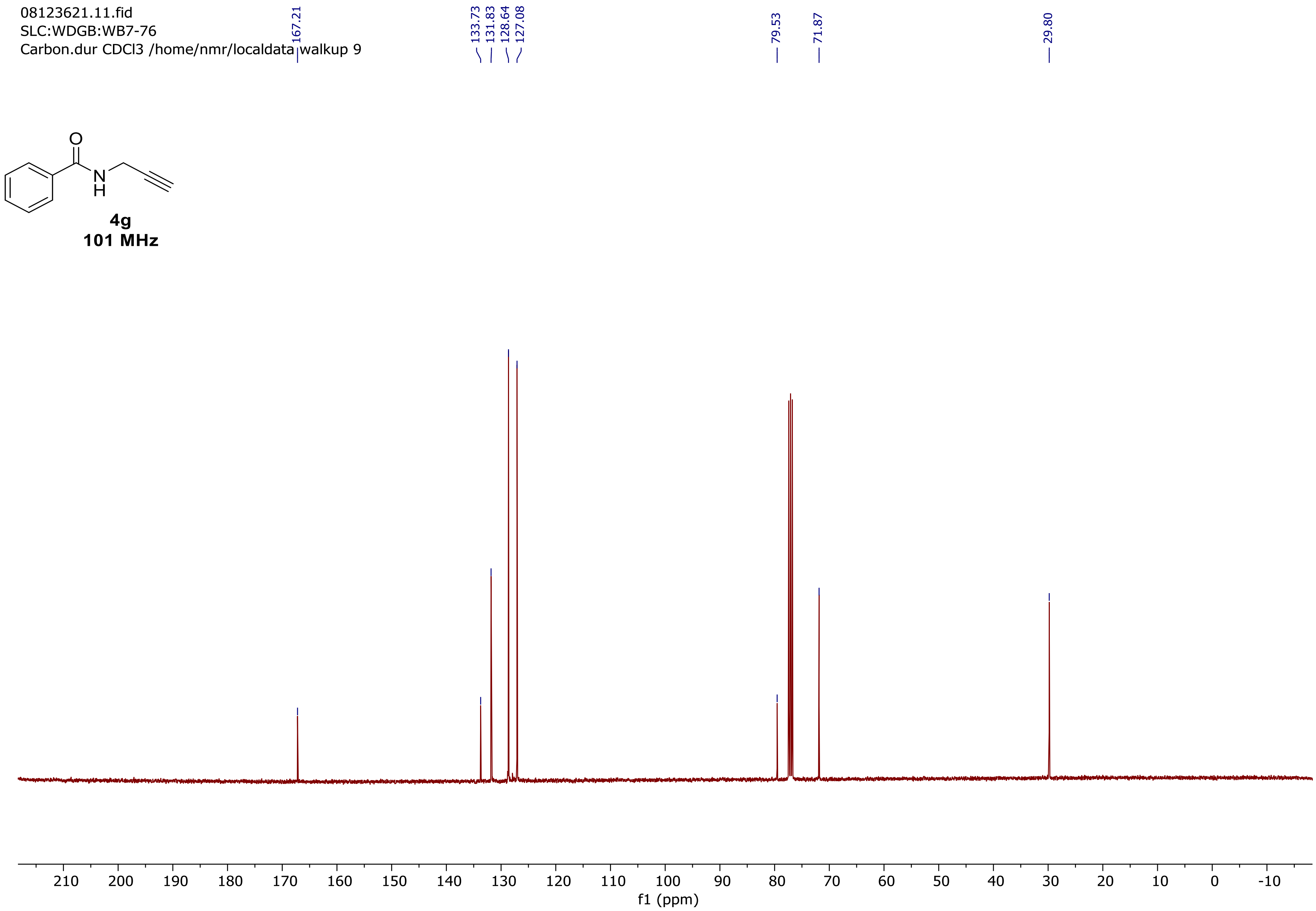
08123621.11.fid
SLC:WDGB:WB7-76
Carbon.dur CDCl3 /home/nmr/localdata walkup 9
167.21
133.73
131.83
128.64
127.08
79.53
71.87
29.80
4g
101 MHz
210 200 190 180 170 160 150 140 130 120 110 100 90 80 70 60 50 40 30 20 10 0 -10
f1 (ppm)

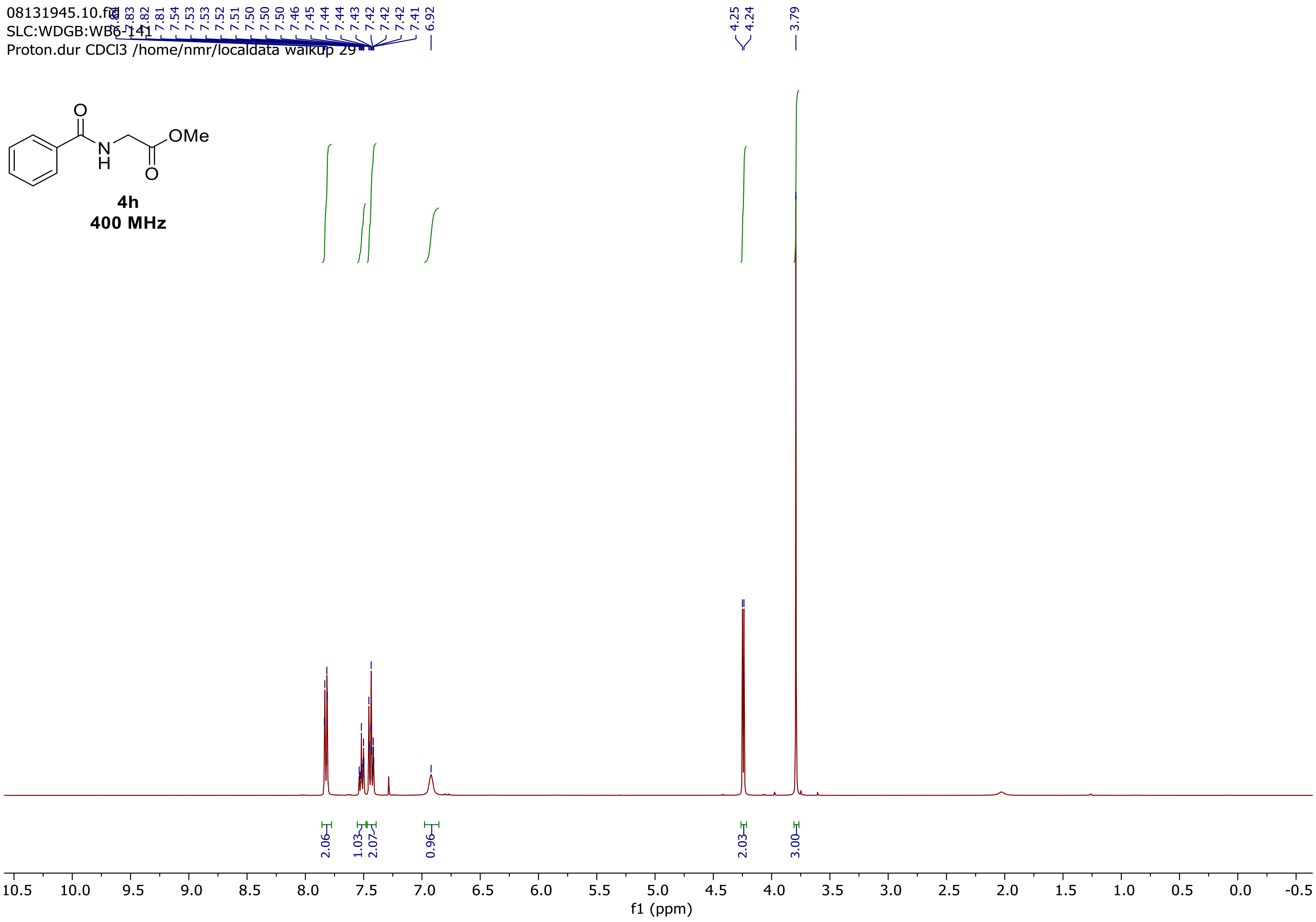
08131945.10.fid
SLC:WDGB:WB6-141
Proton.dur CDCl3 /home/nmr/localdata walkup 29
7.83
7.82
7.81
7.54
7.53
7.53
7.52
7.51
7.50
7.50
7.50
7.46
7.45
7.44
7.44
7.43
7.42
7.42
7.42
7.41
6.92
4.25
4.24
3.79
OMe
N
H
O
O
4h
400 MHz
2.06
1.03
2.07
0.96
2.03
3.00
10.5
10.0
9.5
9.0
8.5
8.0
7.5
7.0
6.5
6.0
5.5
5.0
4.5
4.0
3.5
3.0
2.5
2.0
1.5
1.0
0.5
0.0
-0.5
f1 (ppm)

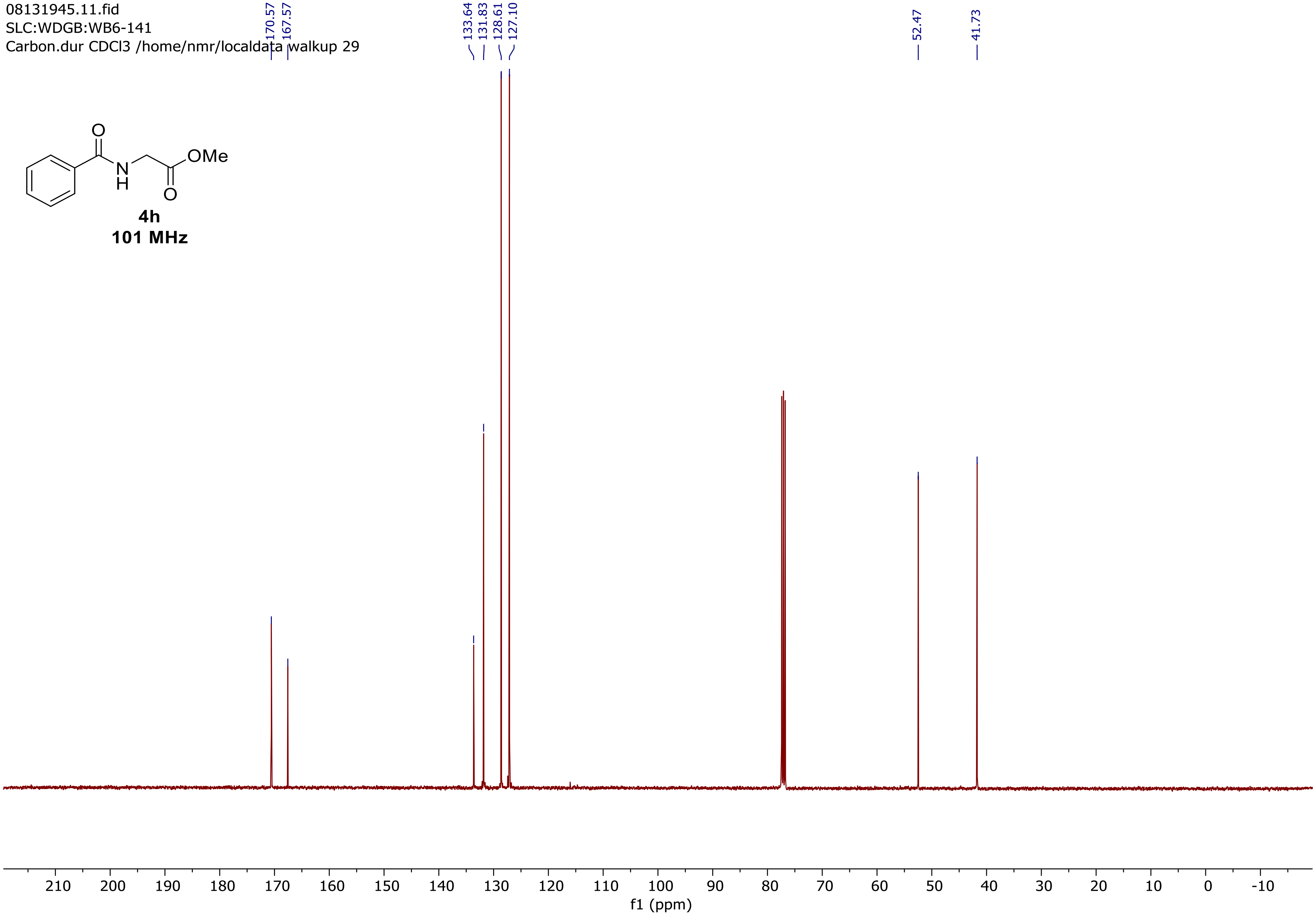
08131945.11.fid
SLC:WDGB:WB6-141
Carbon.dur CDCl3 /home/nmr/localdata walkup 29
170.57
167.57
133.64
131.83
128.61
127.10
52.47
41.73
OMe
4h
101 MHz
210 200 190 180 170 160 150 140 130 120 110 100 90 80 70 60 50 40 30 20 10 0 -10
f1 (ppm)

07173716.10.fid
SLC:WDGB:WB7-10-1
Proton.dur CDCl3 /home/nmr/localdata walkup 25

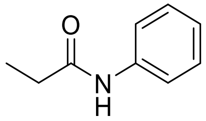

4i
400 MHz

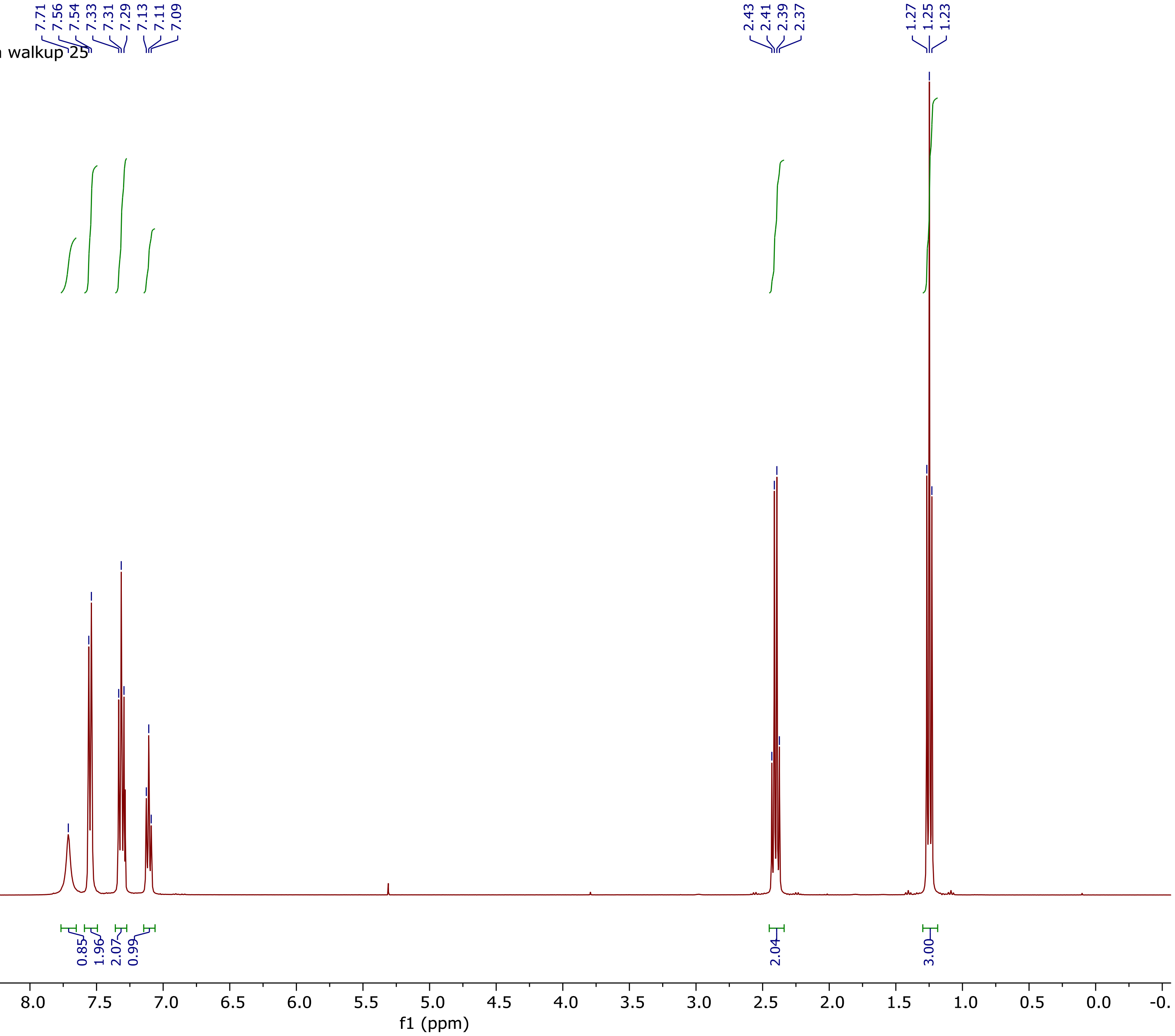

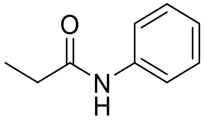

4i
101 MHz

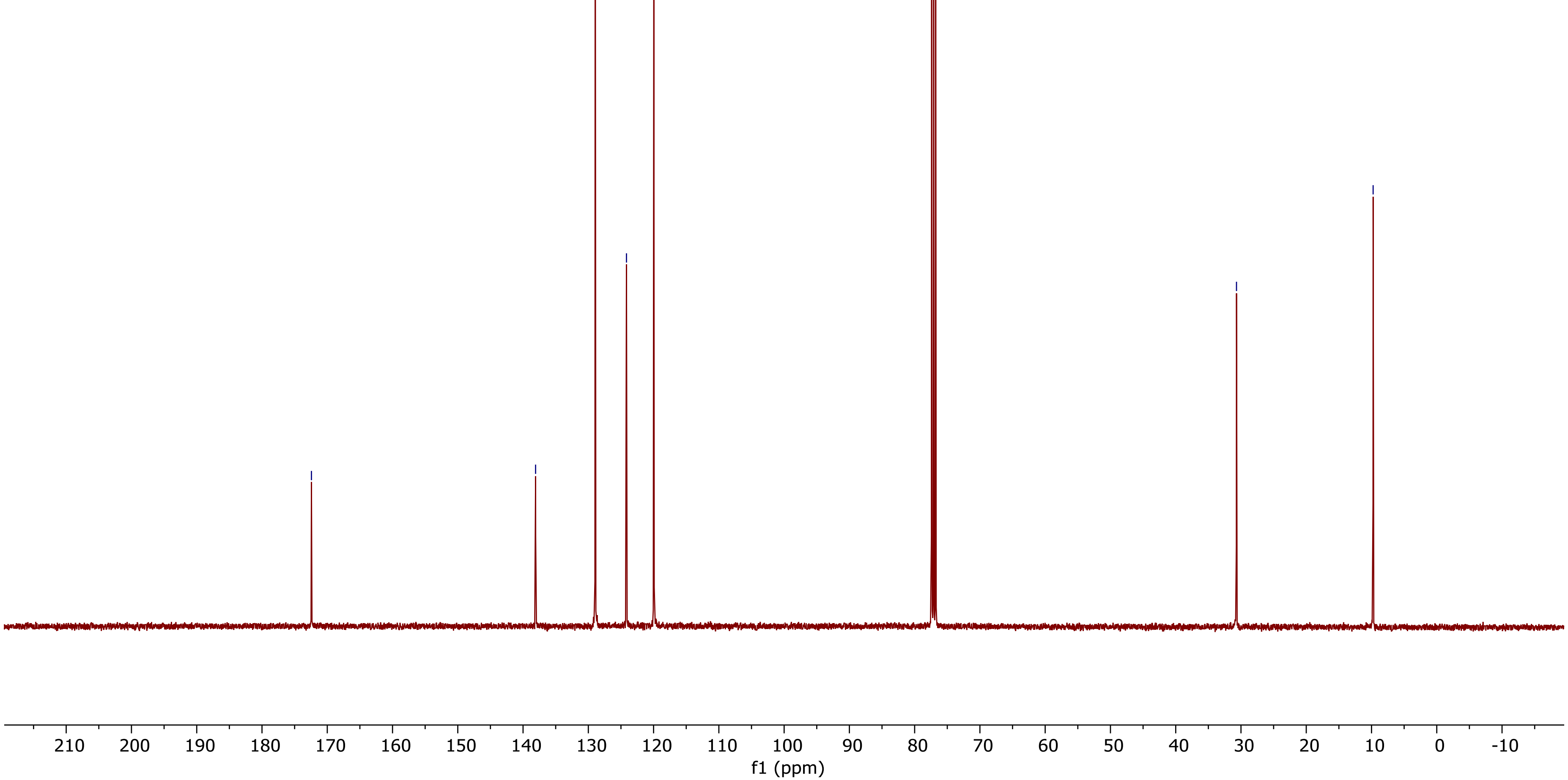
07173716.11.fid
SLC:WDGB:WB7-10-1
Carbon.dur CDCl3 /home/nmr/localdata walkup 25
172.42
138.08
128.94
124.15
119.96
30.69
9.75
210 200 190 180 170 160 150 140 130 120 110 100 90 80 70 60 50 40 30 20 10 0 -10
f1 (ppm)

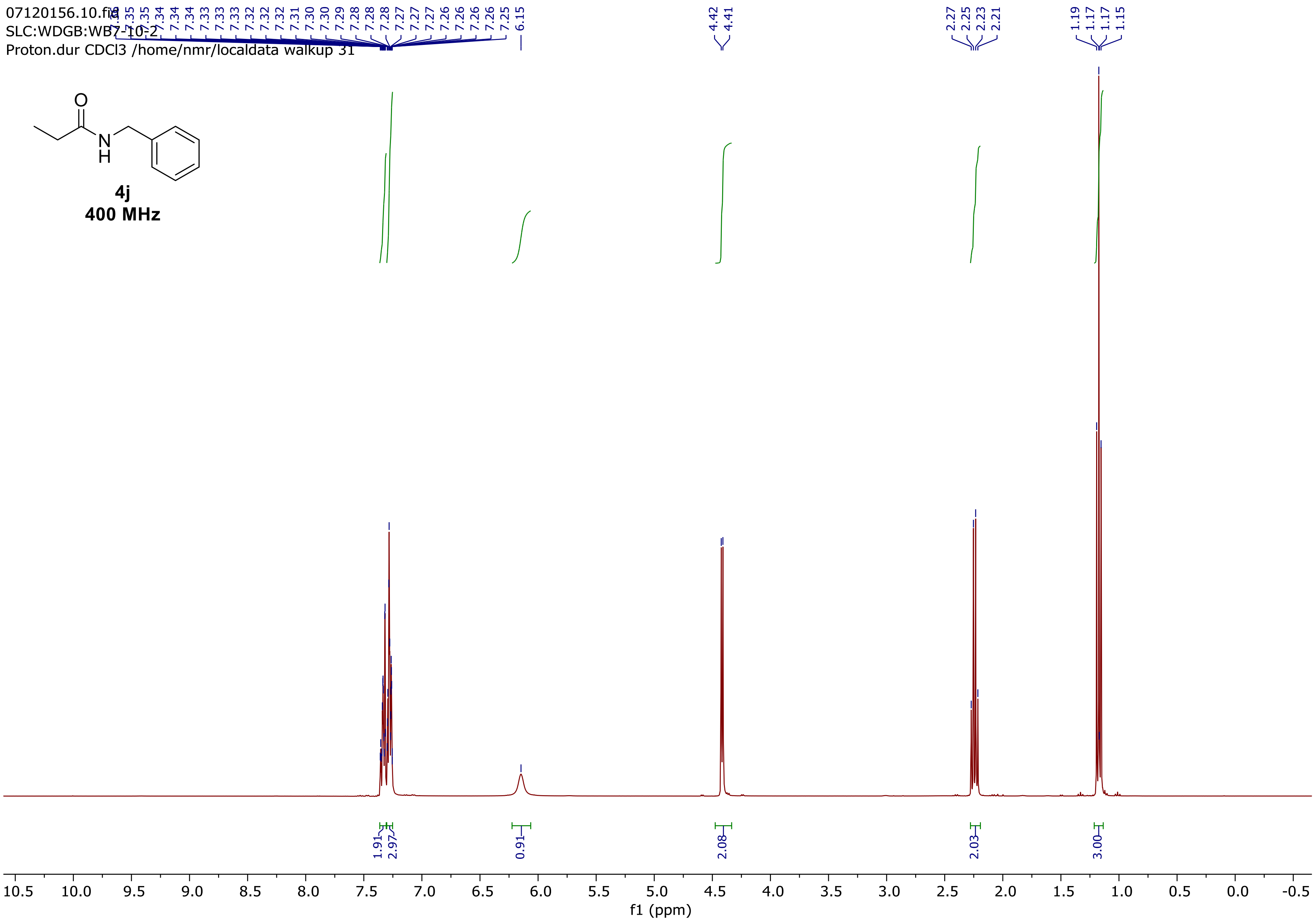
07120156.10.fid
SLC:WDGB:WB7-10-2
Proton.dur CDCl3 /home/nmr/localdata walkup 31
7.35 7.35 7.34 7.34 7.34 7.33 7.33 7.33 7.32 7.32 7.32 7.31 7.30 7.30 7.29 7.28 7.28 7.28 7.27 7.27 7.27 7.26 7.26 7.26 7.26 7.25 6.15
4.42 4.41
2.27 2.25 2.23 2.21
1.19 1.17 1.17 1.15
O
N
H
4j
400 MHz
1.91 2.97 0.91 2.08 2.03 3.00
10.5 10.0 9.5 9.0 8.5 8.0 7.5 7.0 6.5 6.0 5.5 5.0 4.5 4.0 3.5 3.0 2.5 2.0 1.5 1.0 0.5 0.0 -0.5
f1 (ppm)

07120156.11.fid
SLC:WDGB:WB7-10-2
Carbon.dur CDCl3 /home/nmr/localdata walkup 31

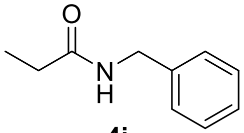

4j
101 MHz

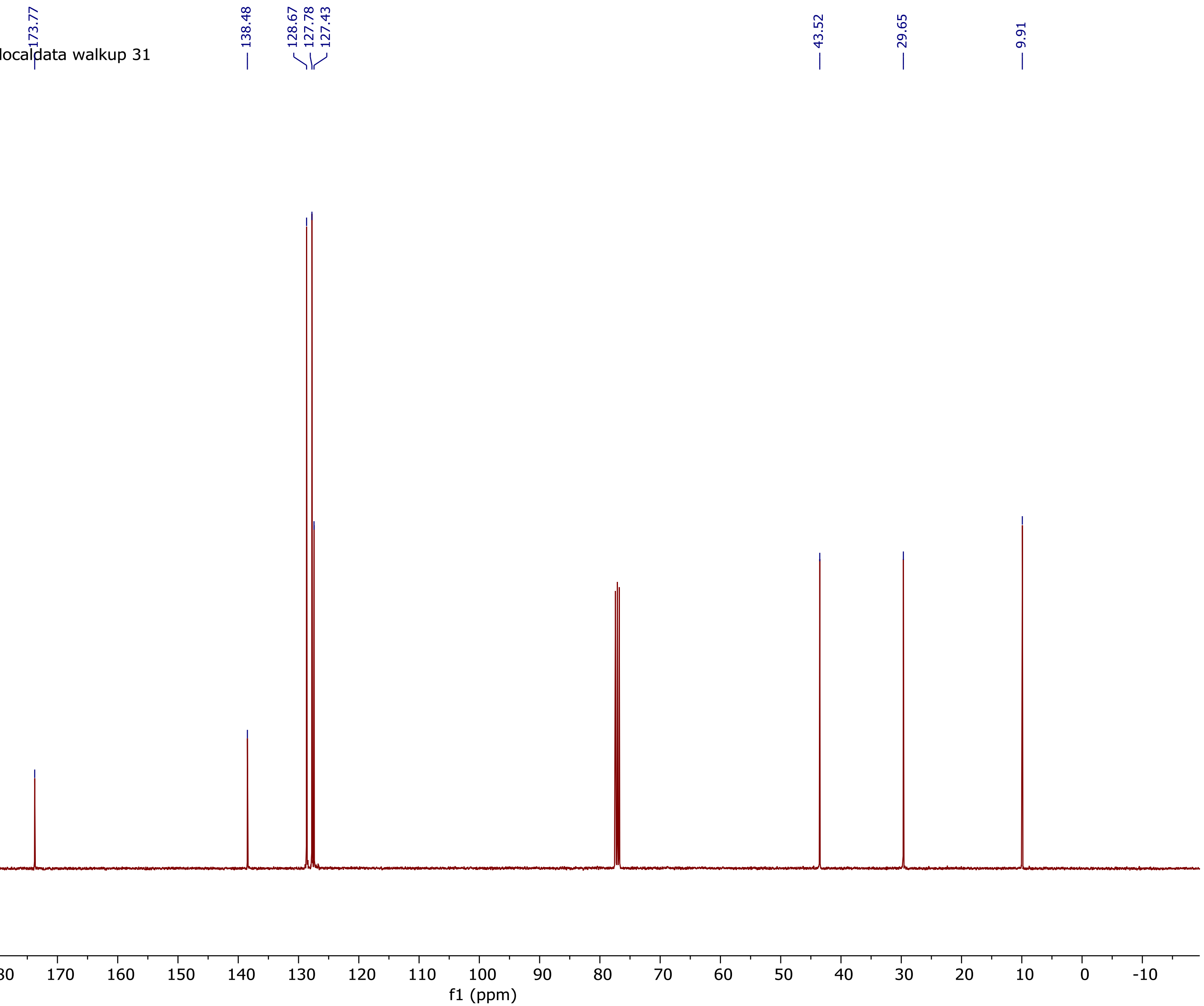

07173659.10.fid
SLC:WDGB:WB7-10-3
Proton.dur CDCl3 /home/nmr/localdata walkup 24

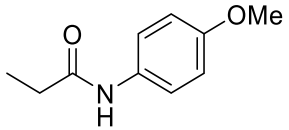

4k
400 MHz

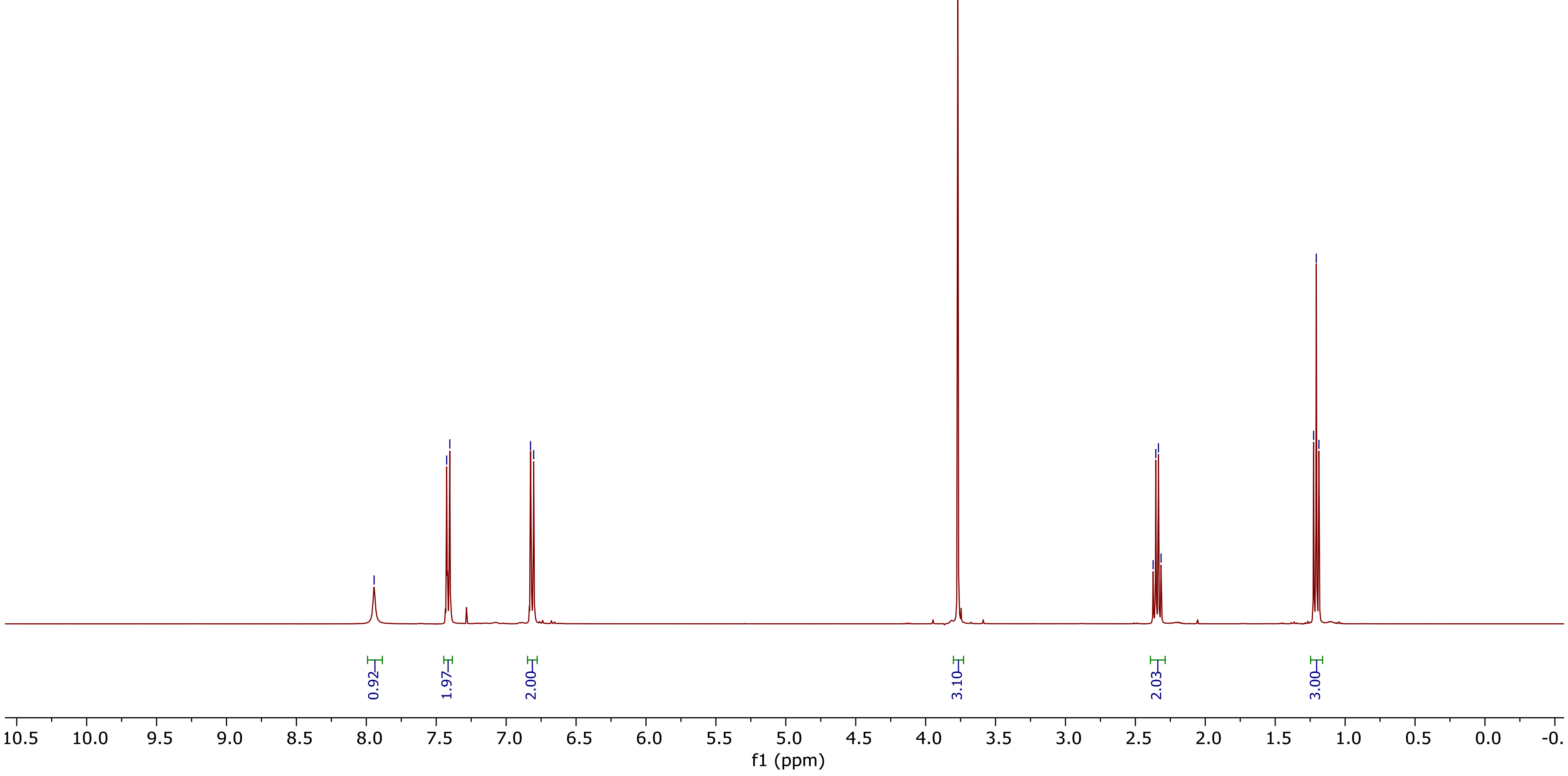

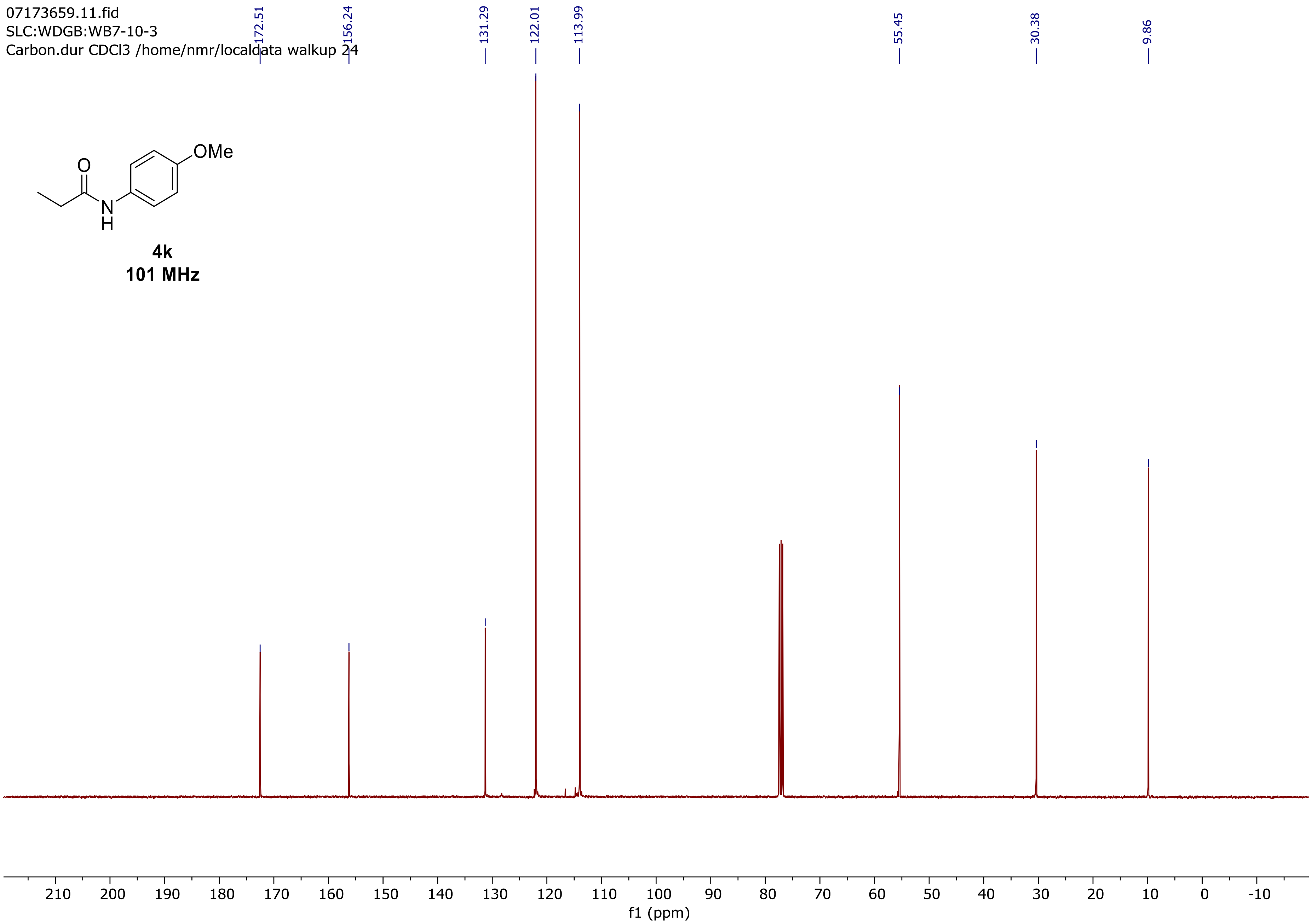
07173659.11.fid
SLC:WDGB:WB7-10-3
Carbon.dur CDCl3 /home/nmr/localdata walkup 24
172.51
156.24
131.29
122.01
113.99
55.45
30.38
9.86
OMe
O
N
H
4k
101 MHz
210 200 190 180 170 160 150 140 130 120 110 100 90 80 70 60 50 40 30 20 10 0 -10
f1 (ppm)

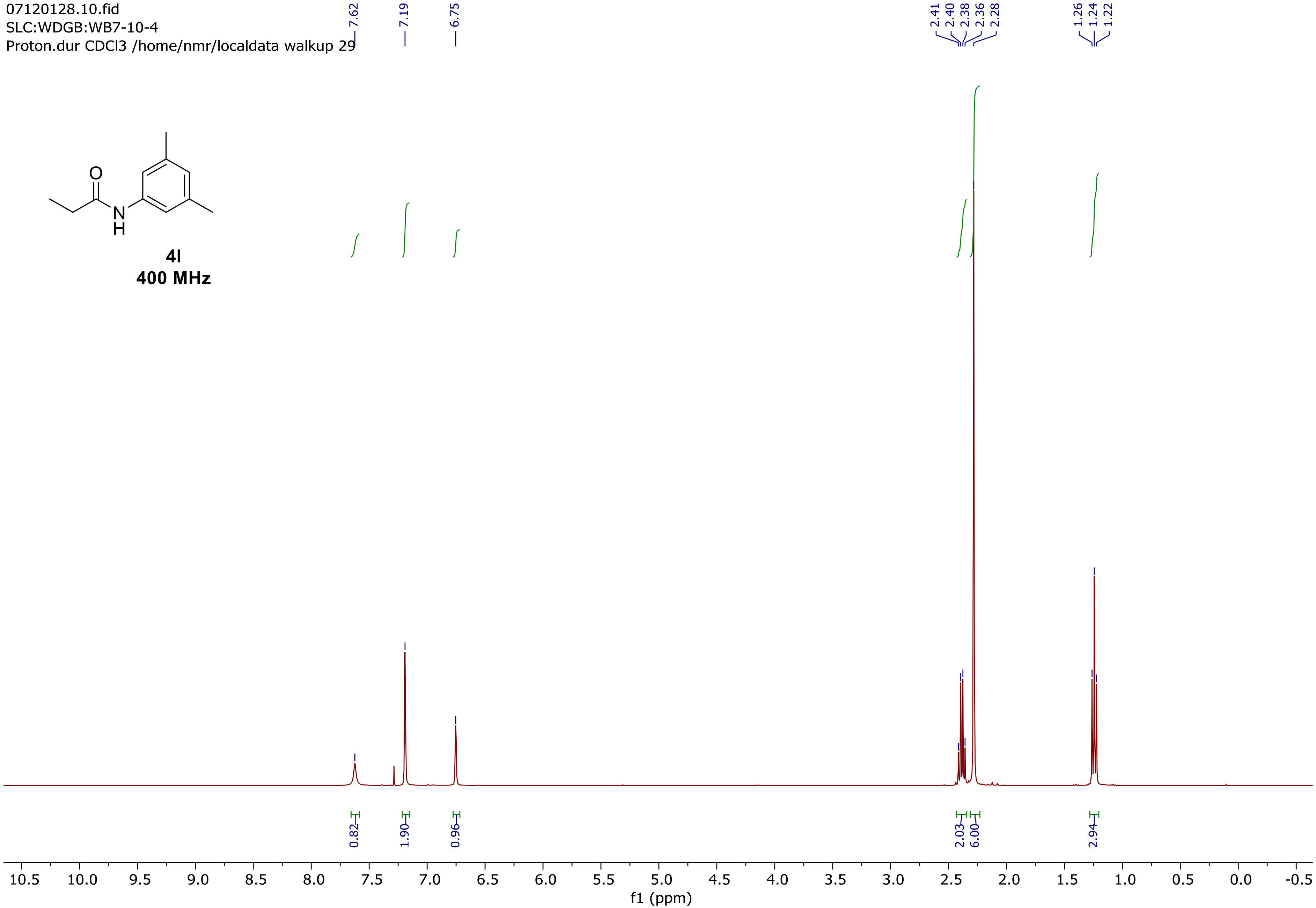
07120128.10.fid
SLC:WDGB:WB7-10-4
Proton.dur CDCl3 /home/nmr/localdata walkup 29
7.62
7.19
6.75
2.41
2.40
2.38
2.36
2.28
1.26
1.24
1.22
4l
400 MHz
0.82
1.90
0.96
2.03
6.00
2.94
10.5 10.0 9.5 9.0 8.5 8.0 7.5 7.0 6.5 6.0 5.5 5.0 4.5 4.0 3.5 3.0 2.5 2.0 1.5 1.0 0.5 0.0 -0.5
f1 (ppm)

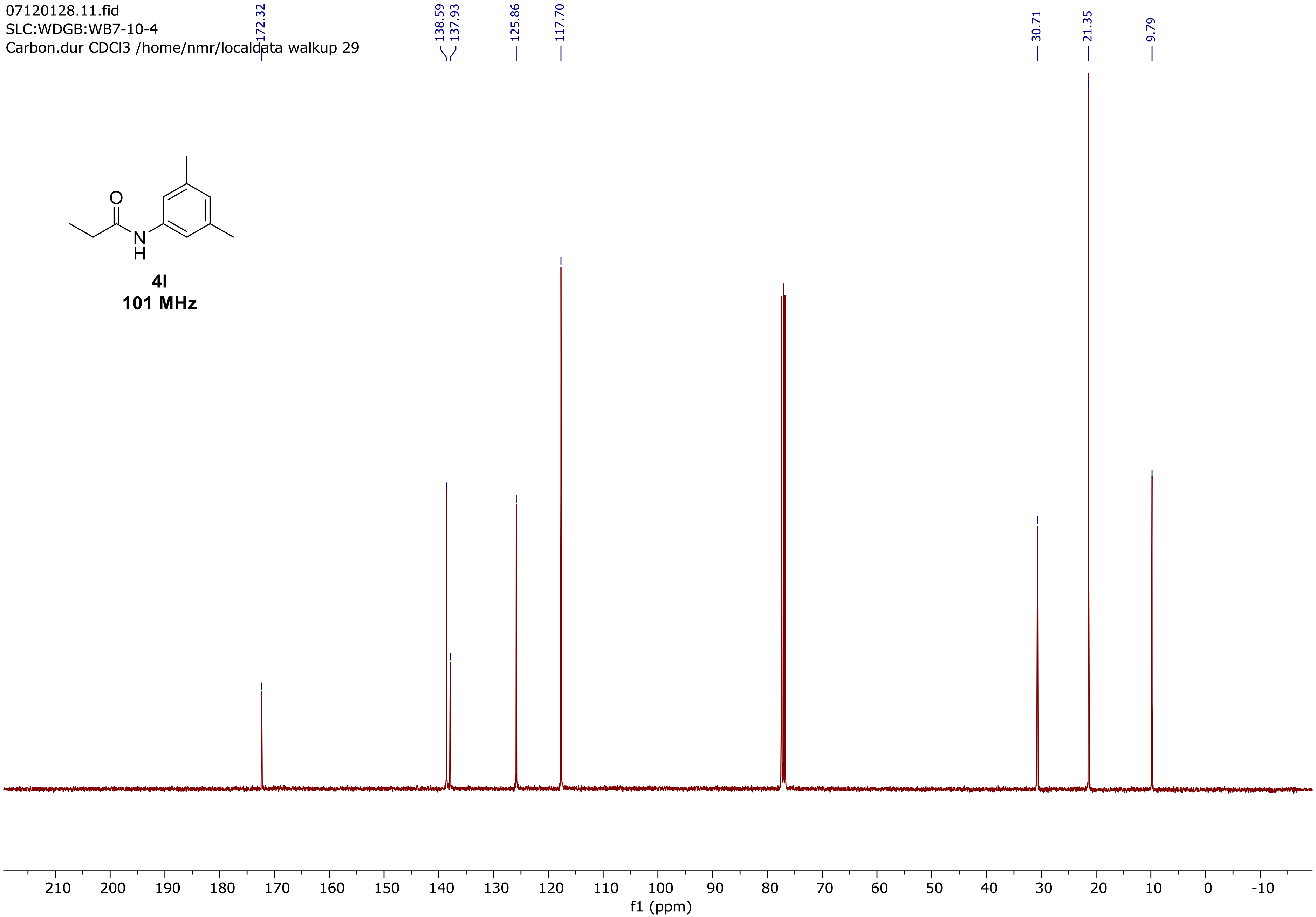
07120128.11.fid
SLC:WDGB:WB7-10-4
Carbon.dur CDCl3 /home/nmr/localdata walkup 29
172.32
138.59
137.93
125.86
117.70
30.71
21.35
9.79
4l
101 MHz
210 200 190 180 170 160 150 140 130 120 110 100 90 80 70 60 50 40 30 20 10 0 -10
f1 (ppm)

07173638.10.fid
SLC:WDGB:WB7-10-5
Proton.dur CDCl3 /home/nmr/localdata walkup 23

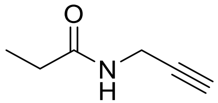

4m
400 MHz

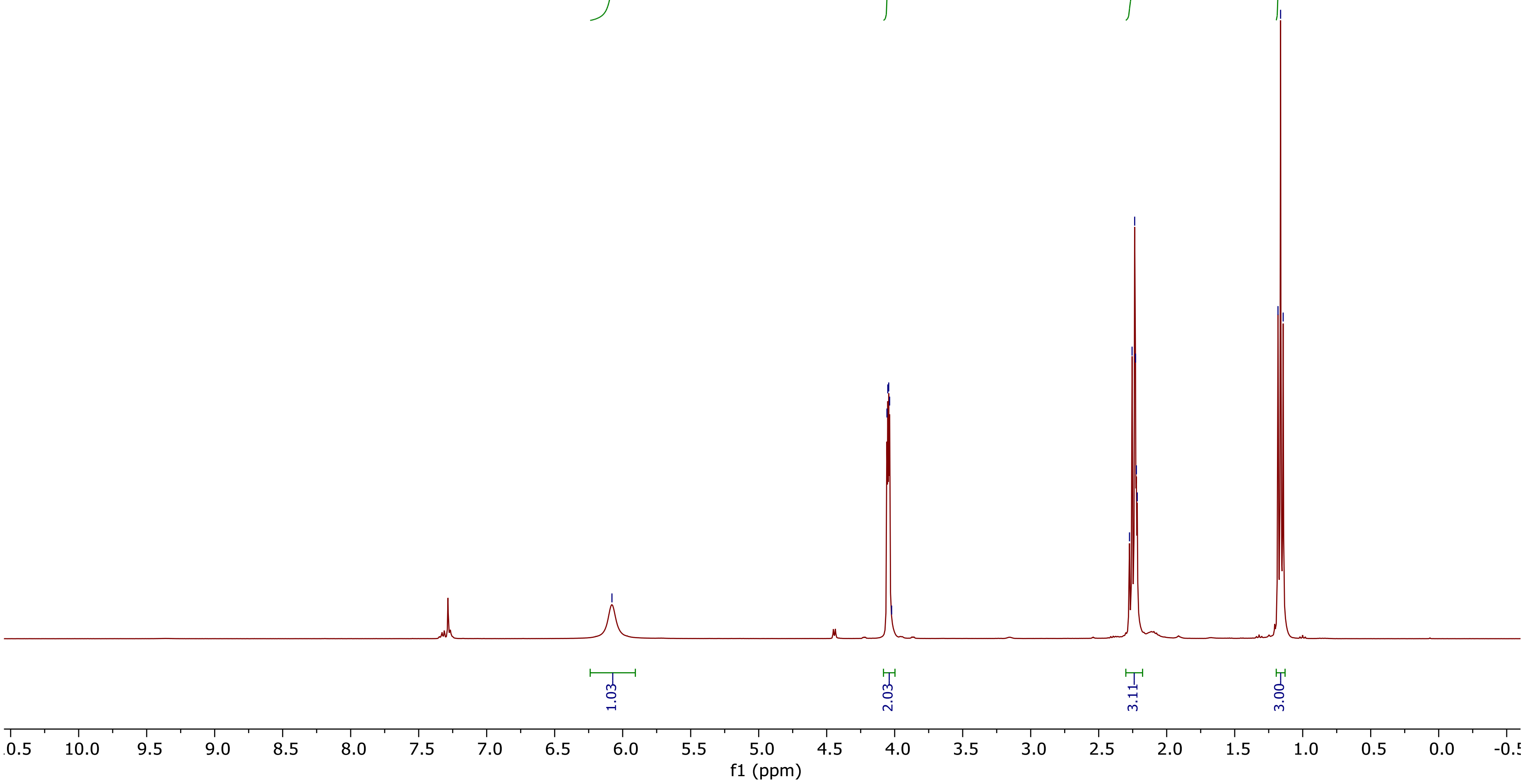

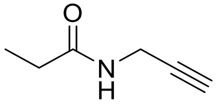
O
N
H

4m
101 MHz

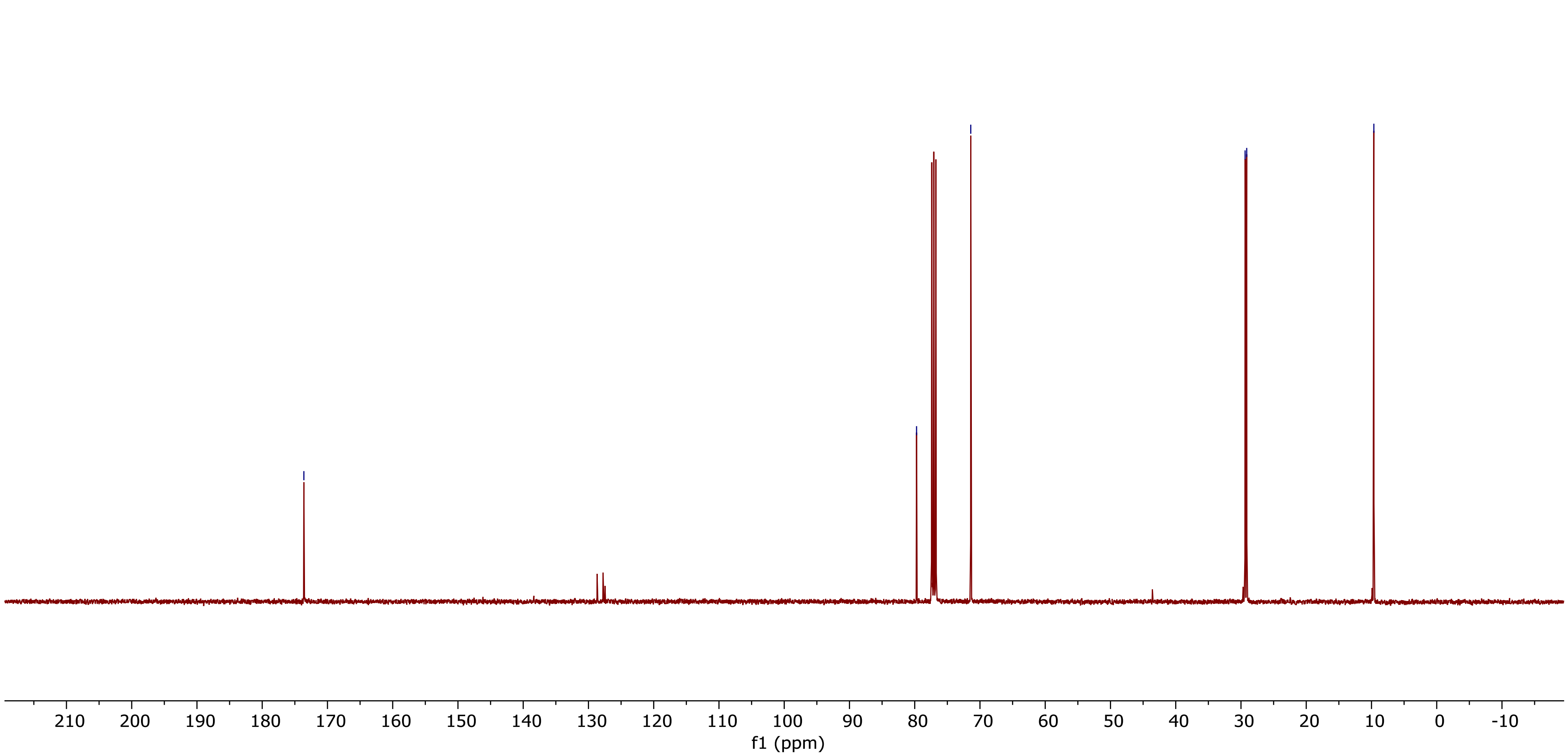
07173638.11.fid
SLC:WDGB:WB7-10-5
Carbon.dur CDCl3 /home/nmr/localdata walkup 23
173.62
79.72
71.41
29.37
29.12
9.64
210 200 190 180 170 160 150 140 130 120 110 100 90 80 70 60 50 40 30 20 10 0 -10
f1 (ppm)

07120142.10.fid
SLC:WDGB:WB7-10-6
Proton.dur CDCl3 /home/nmr/localdata walkup 30

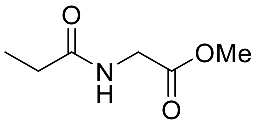

4n
400 MHz

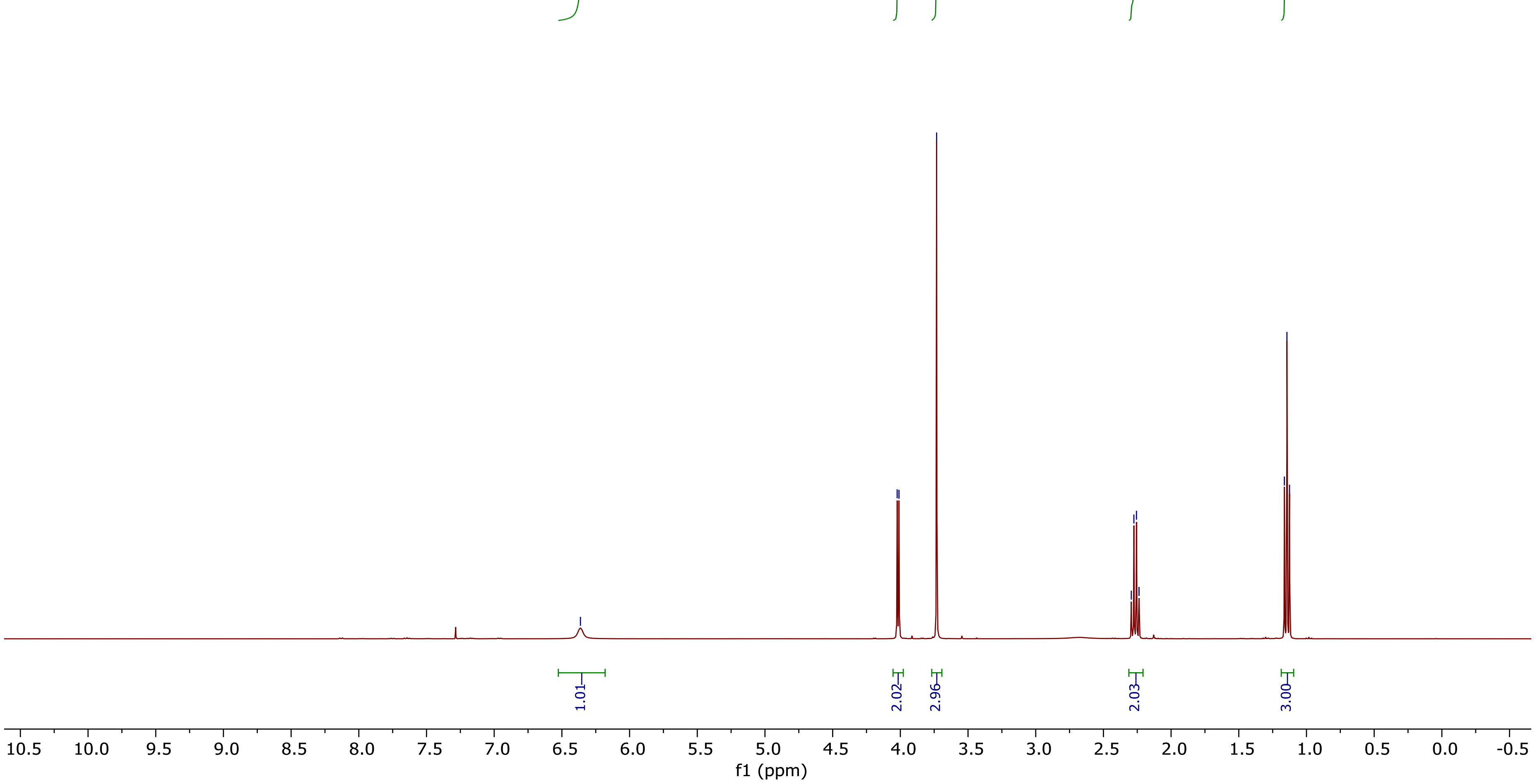

07120142.11.fid
SLC:WDGB:WB7-10-6
Carbon.dur CDCl3 /home/nmr/localdata walkup 30

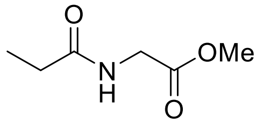

4n
101 MHz

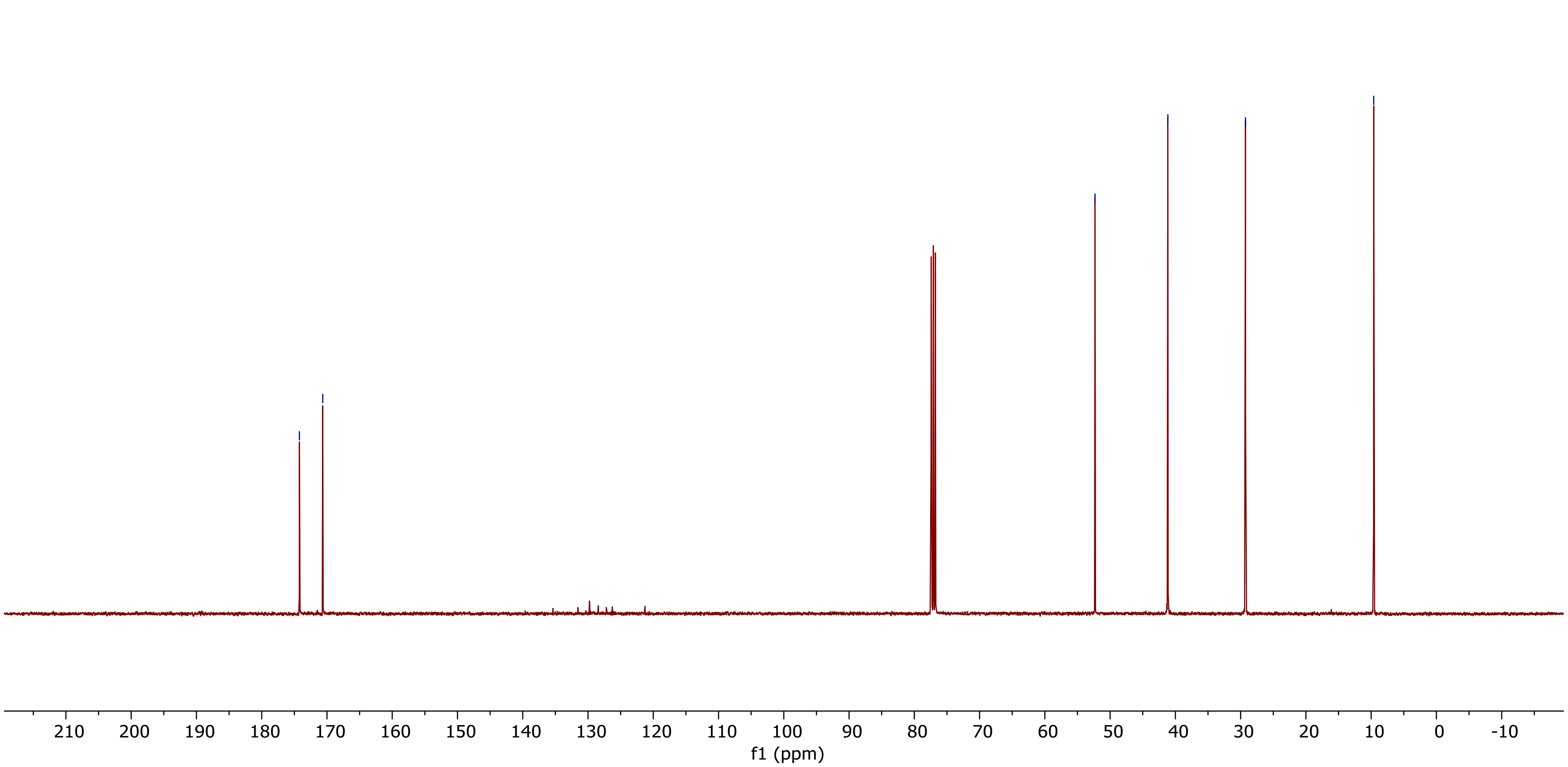

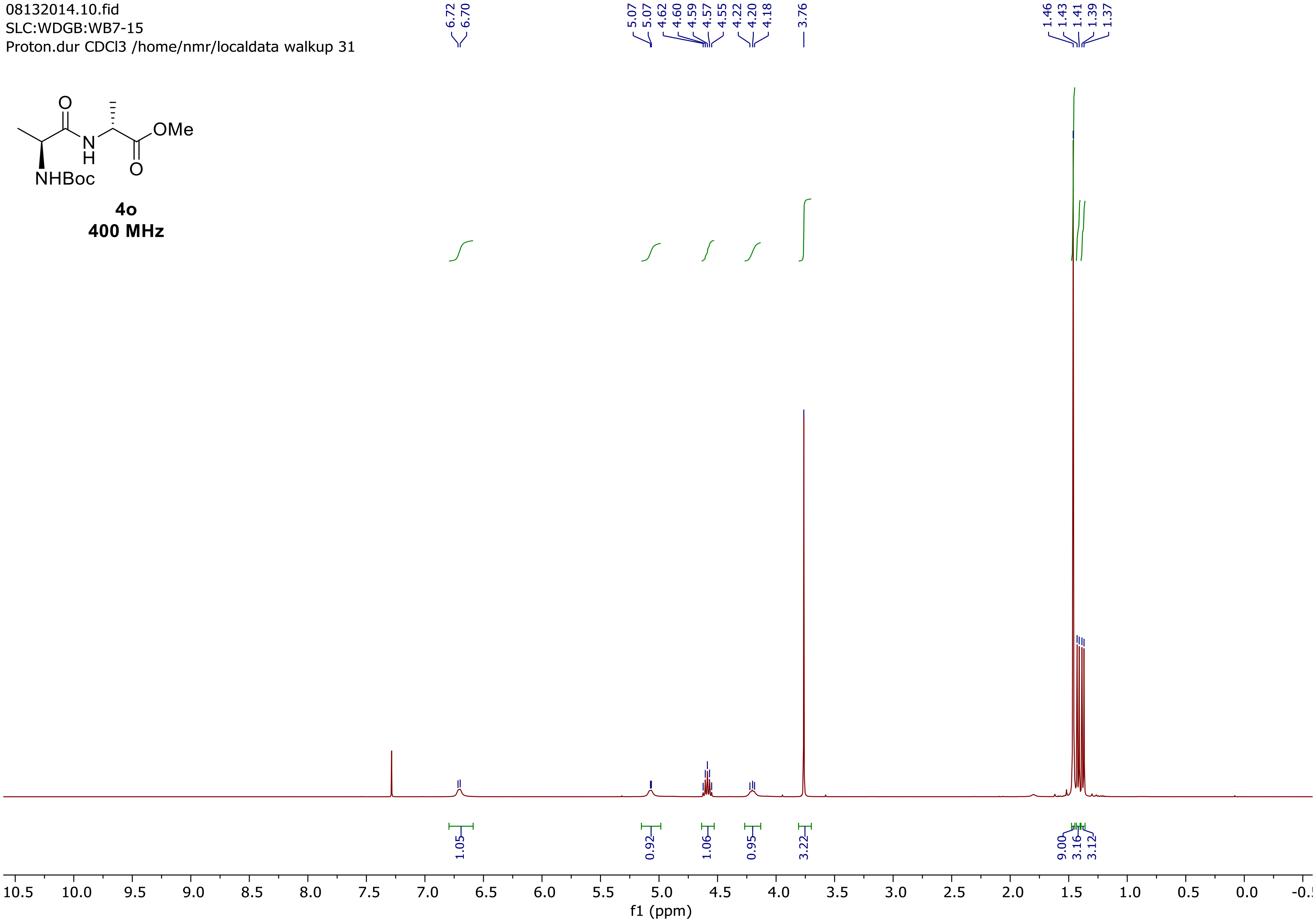
08132014.10.fid
SLC:WDGB:WB7-15
Proton.dur CDCl3 /home/nmr/localdata walkup 31
O
OMe
N
H
O
NHBoc
4o
400 MHz
6.72
6.70
5.07
5.07
4.62
4.60
4.59
4.57
4.55
4.22
4.20
4.18
3.76
1.46
1.43
1.41
1.39
1.37
1.05
0.92
1.06
0.95
3.22
9.00
3.16
3.12
10.5
10.0
9.5
9.0
8.5
8.0
7.5
7.0
6.5
6.0
5.5
5.0
4.5
4.0
3.5
3.0
2.5
2.0
1.5
1.0
0.5
0.0
f1 (ppm)

08132014.11.fid
SLC:WDGB:WB7-15
Carbon.dur CDCl3 /home/nmr/localdata walkup 31

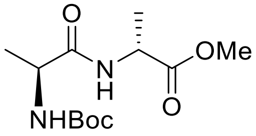

4o
101 MHz

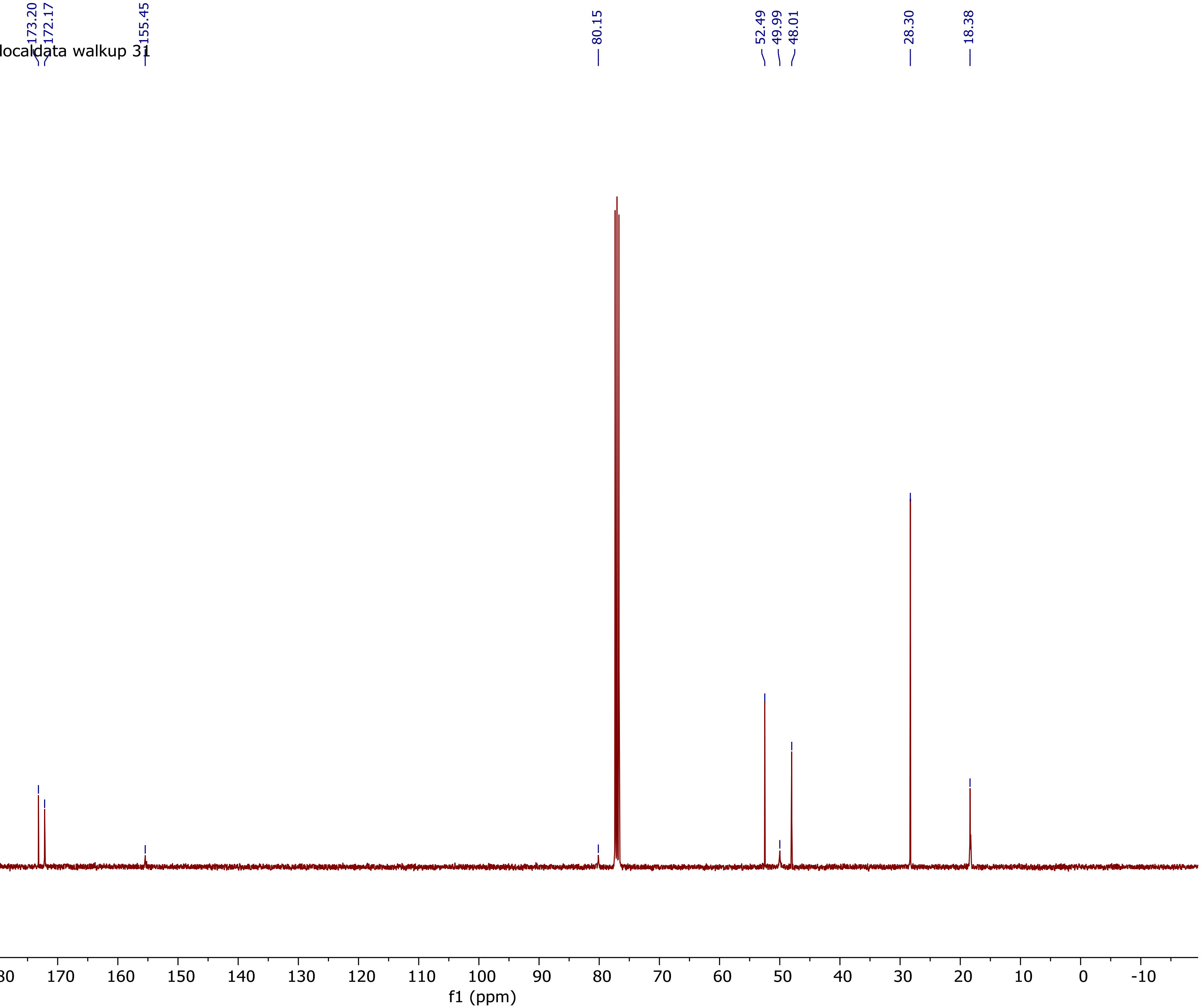

06110427.10.fid
SLC:WDGB:WB7-25-6
Proton.dur CDCl3 /home/nmr/localdata walkup 33

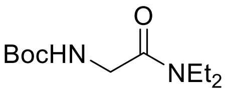

4p
400 MHz

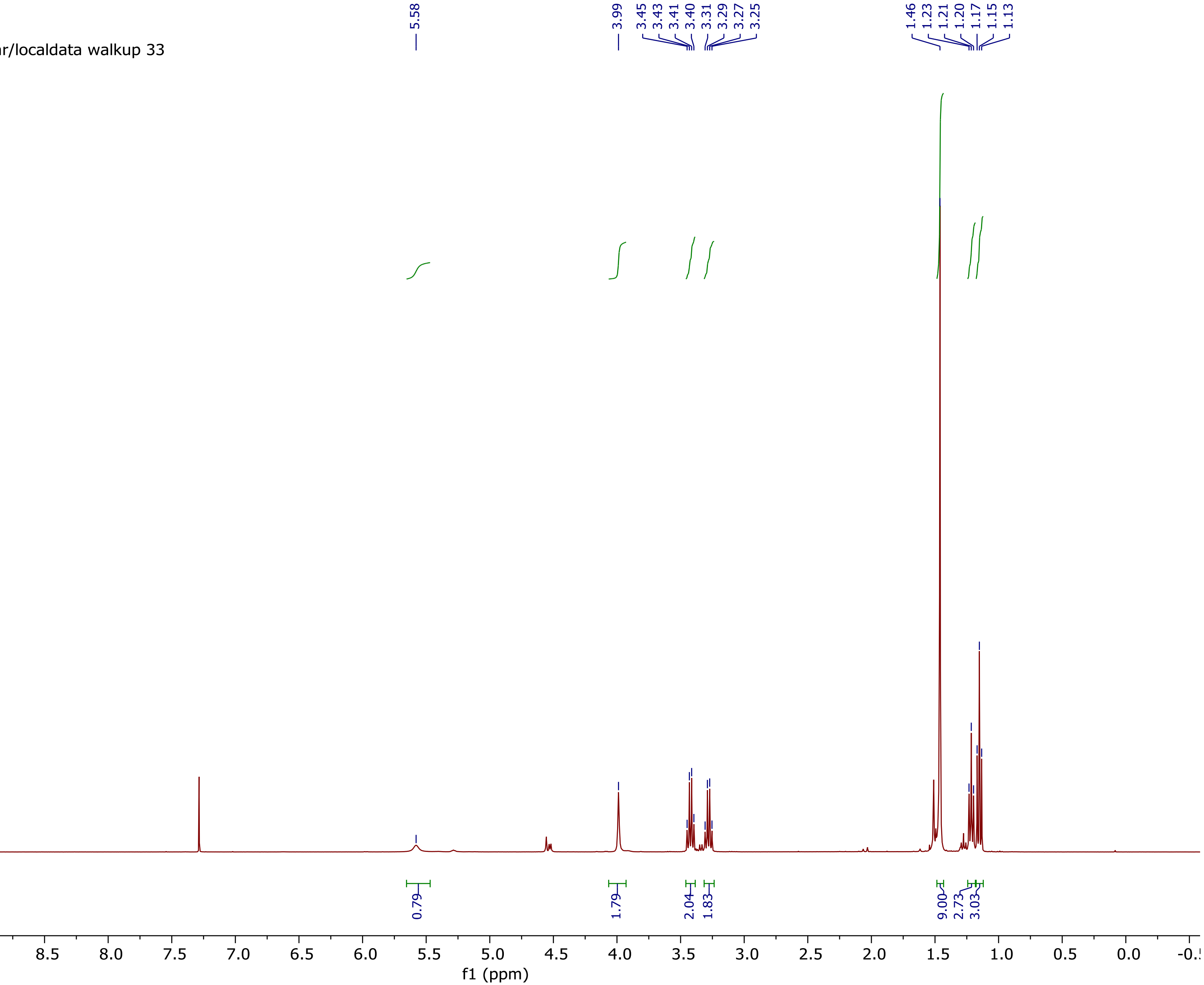

06110427.11.fid
SLC:WDGB:WB7-25-6
Carbon.dur CDCl3 /home/nmr/localdata walkup 33

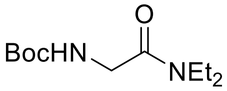

4p
101 MHz

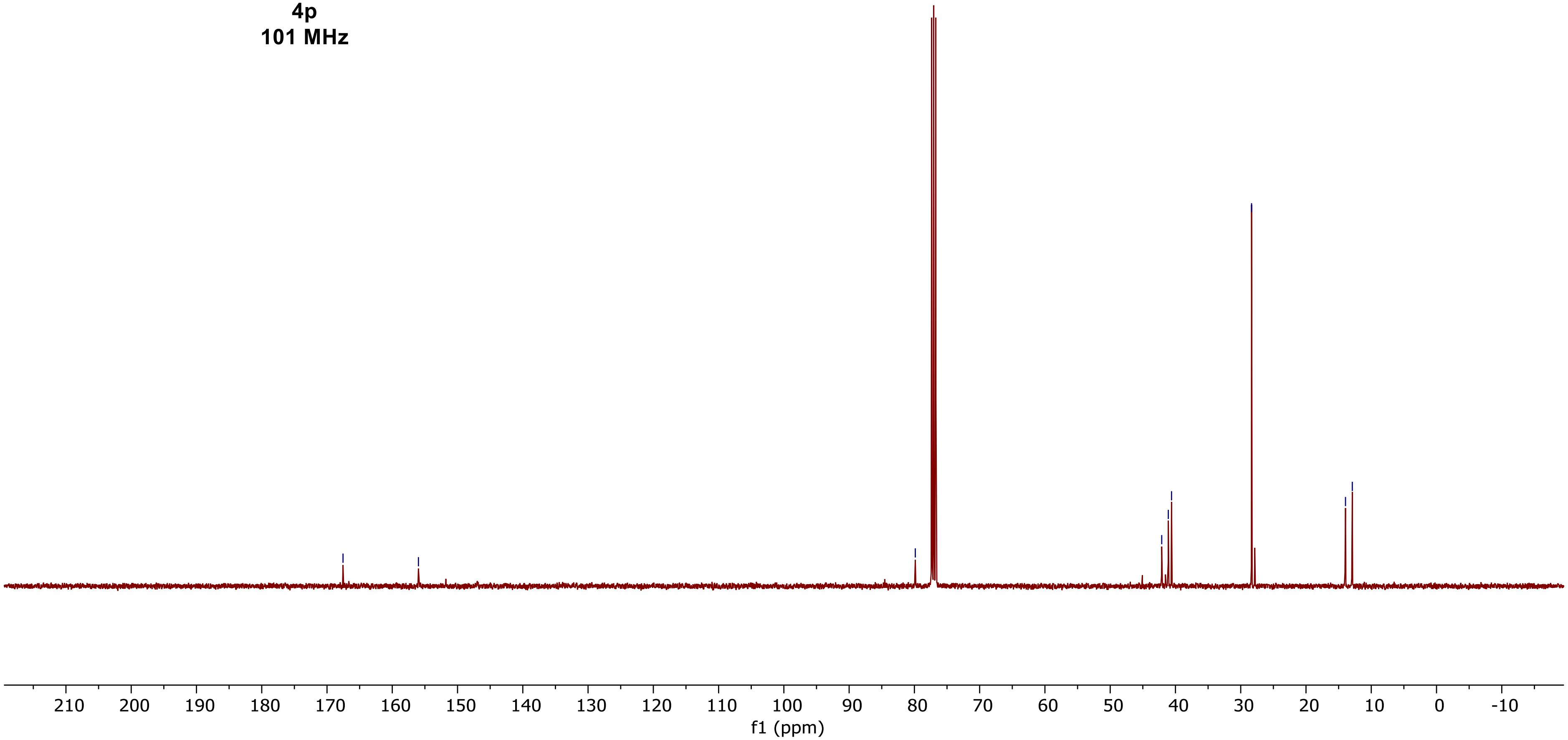

07173742.10.fid
SLC:WDGB:WB7-17-2
Proton.dur CDCl3 /home/nmr/localdata walkup 26

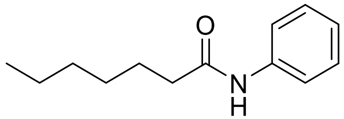

4q
400 MHz

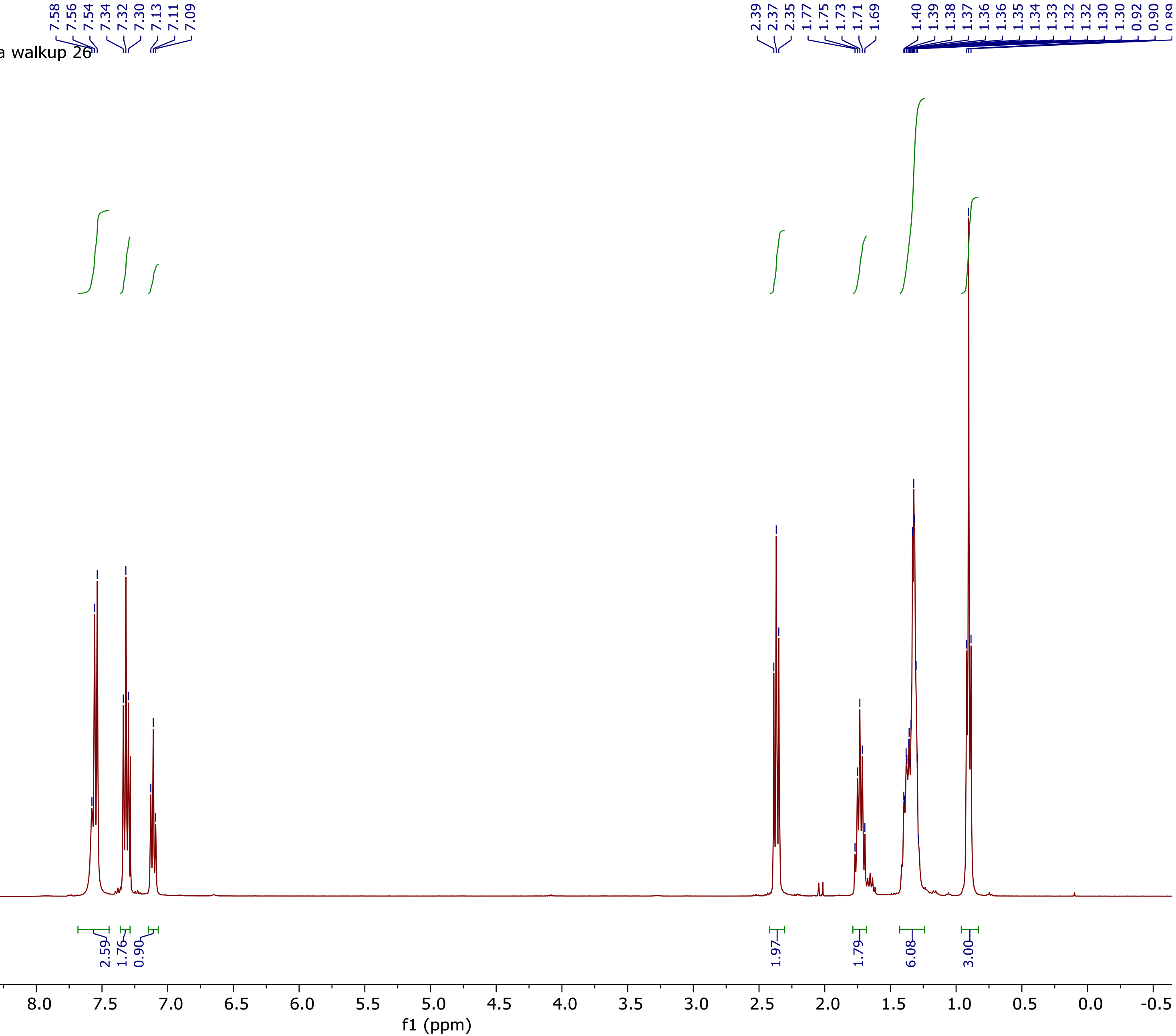

07173742.11.fid
SLC:WDGB:WB7-17-2
Carbon.dur CDCl3 /home/nmr/localdata walkup 26

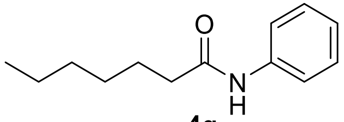

4q
101 MHz

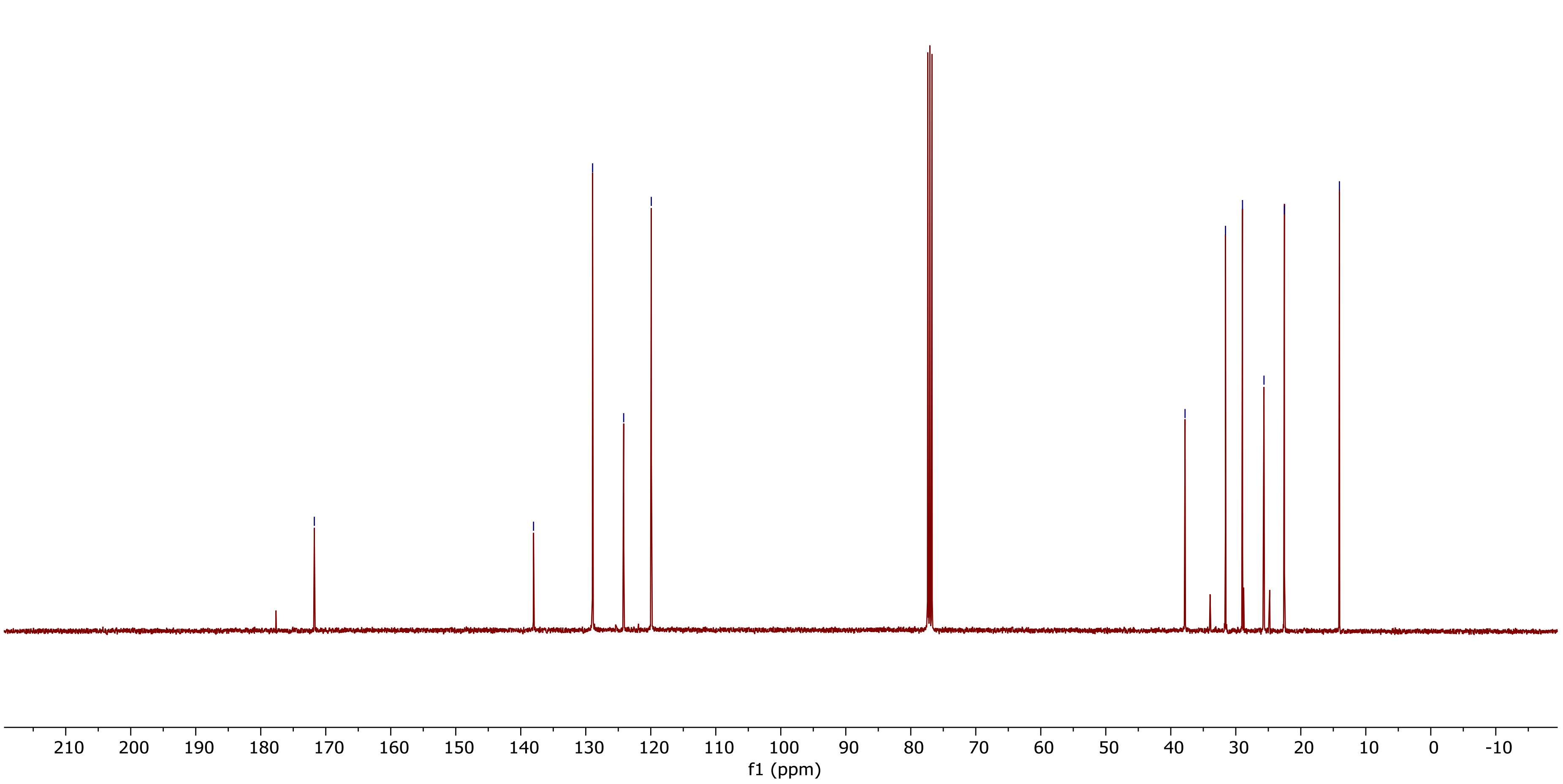

02111559.10.fid
SLC:WB:WB 7-14-4
Proton.dur CDCl3 /home/nmr/localdata walkup 30

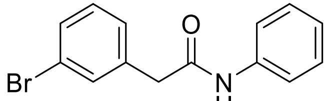
4r
400 MHz

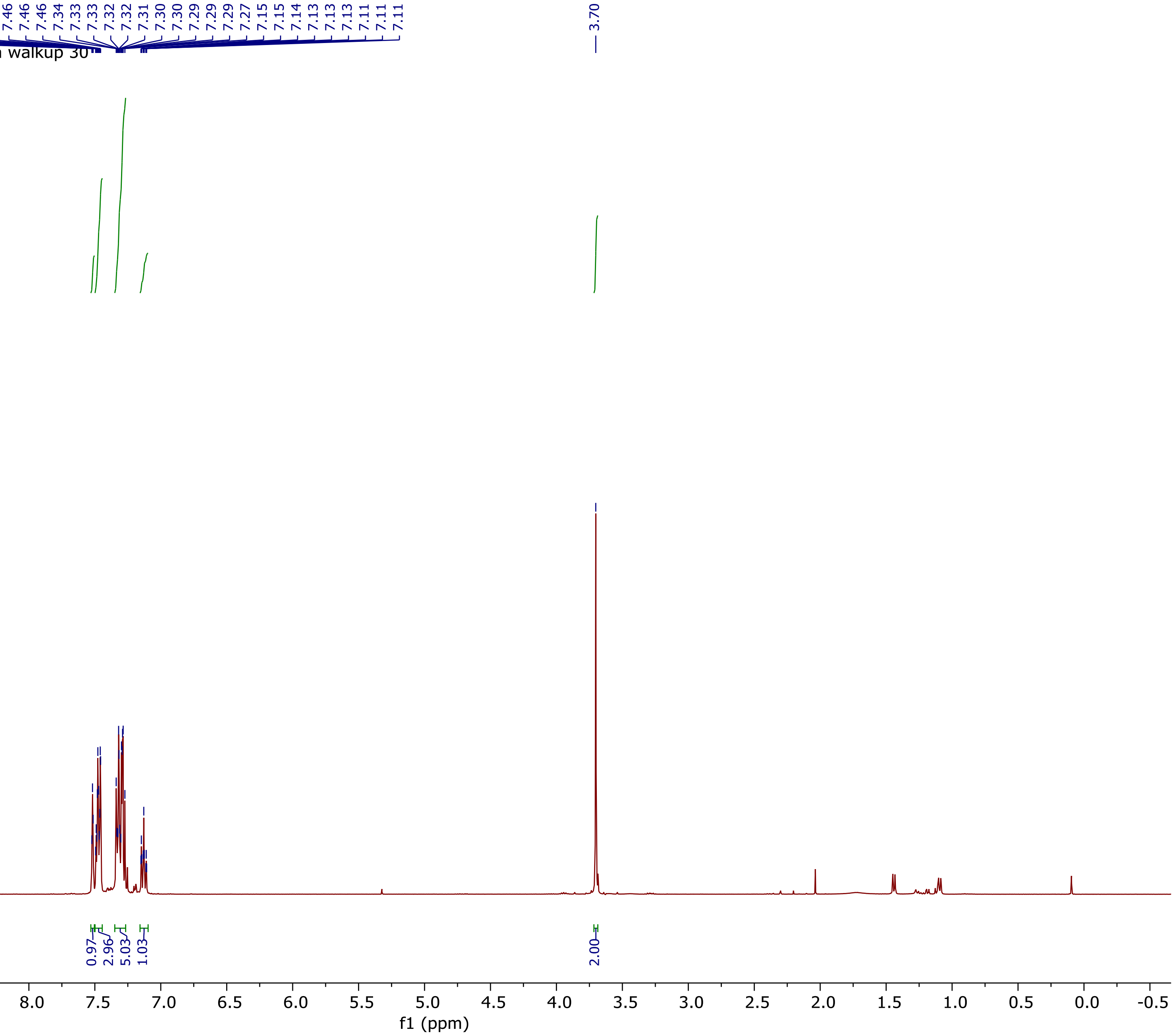

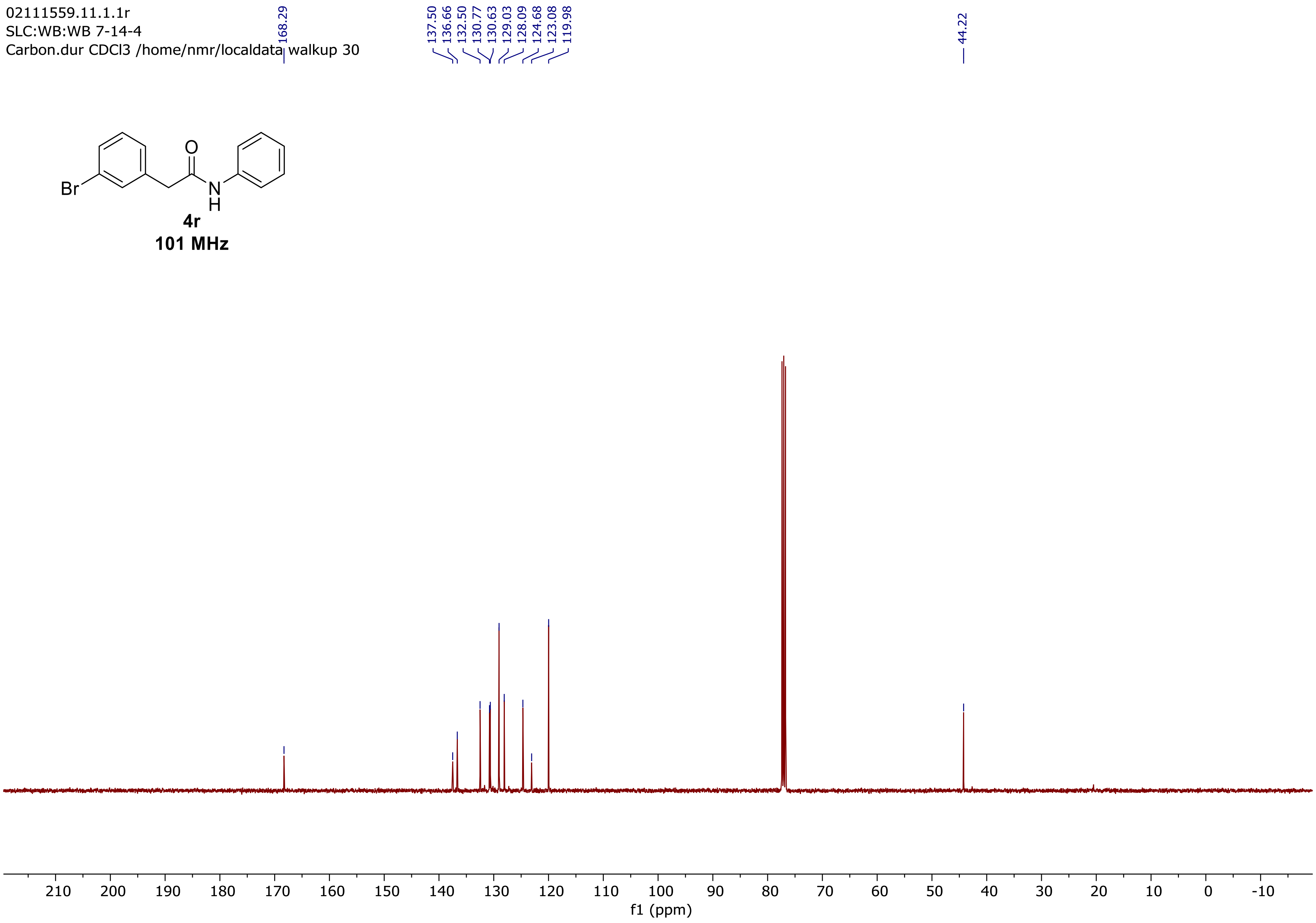
02111559.11.1.1r
SLC:WB:WB 7-14-4
Carbon.dur CDCl3 /home/nmr/localdata walkup 30
168.29
137.50
136.66
132.50
130.77
130.63
129.03
128.09
124.68
123.08
119.98
44.22
Br
O
N
H
4r
101 MHz
210 200 190 180 170 160 150 140 130 120 110 100 90 80 70 60 50 40 30 20 10 0 -10
f1 (ppm)

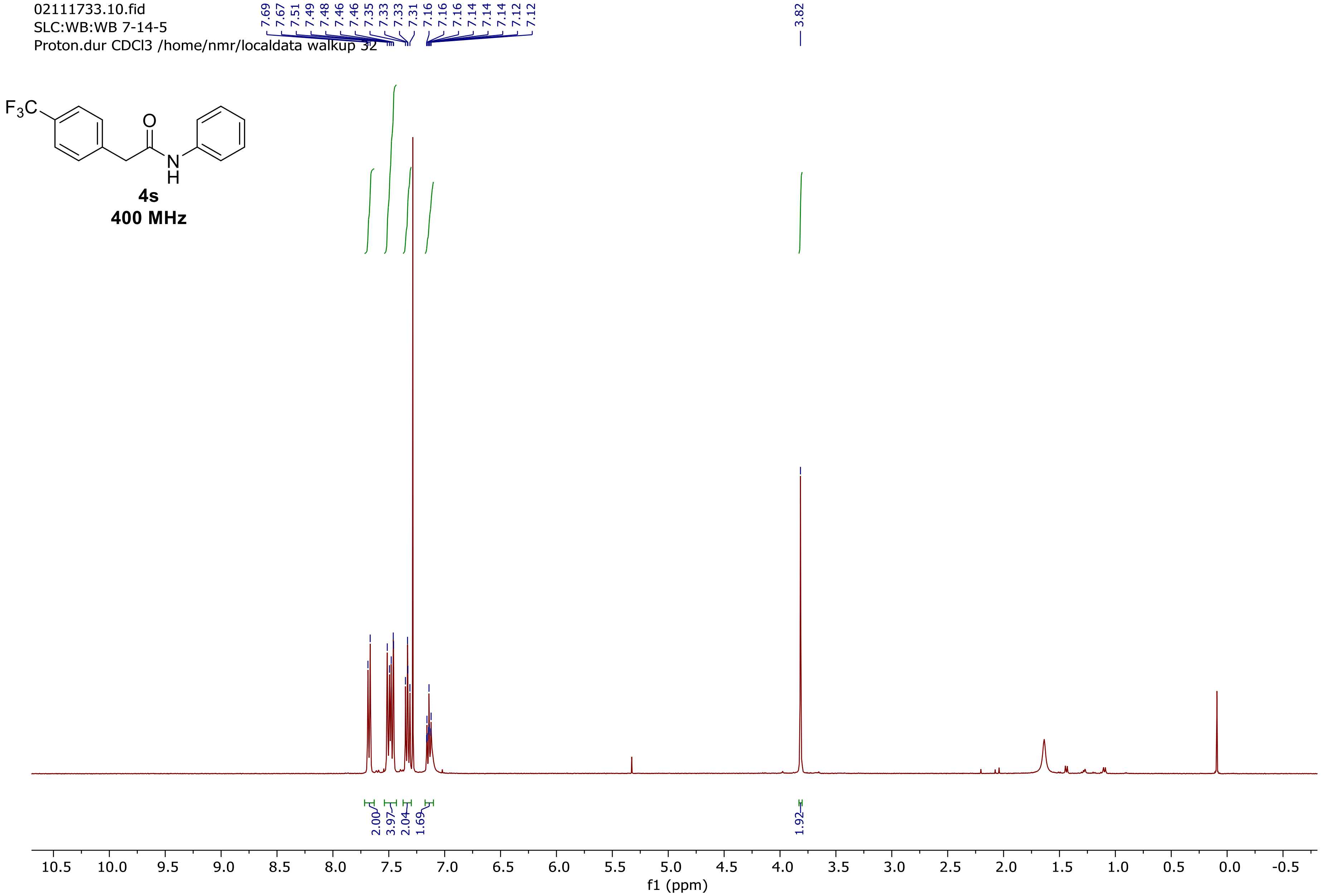
02111733.10.fid
SLC:WB:WB 7-14-5
Proton.dur CDCl3 /home/nmr/localdata walkup 32
7.69 7.67 7.51 7.49 7.48 7.46 7.46 7.35 7.33 7.33 7.31 7.16 7.16 7.16 7.14 7.14 7.14 7.12 7.12
3.82
F3C
O
N
H
4s
400 MHz
2.00 3.97 2.04 1.69
1.92
10.5 10.0 9.5 9.0 8.5 8.0 7.5 7.0 6.5 6.0 5.5 5.0 4.5 4.0 3.5 3.0 2.5 2.0 1.5 1.0 0.5 0.0 -0.5
f1 (ppm)

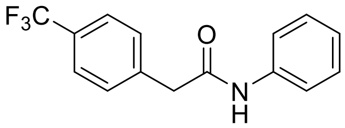

4s
376 MHz

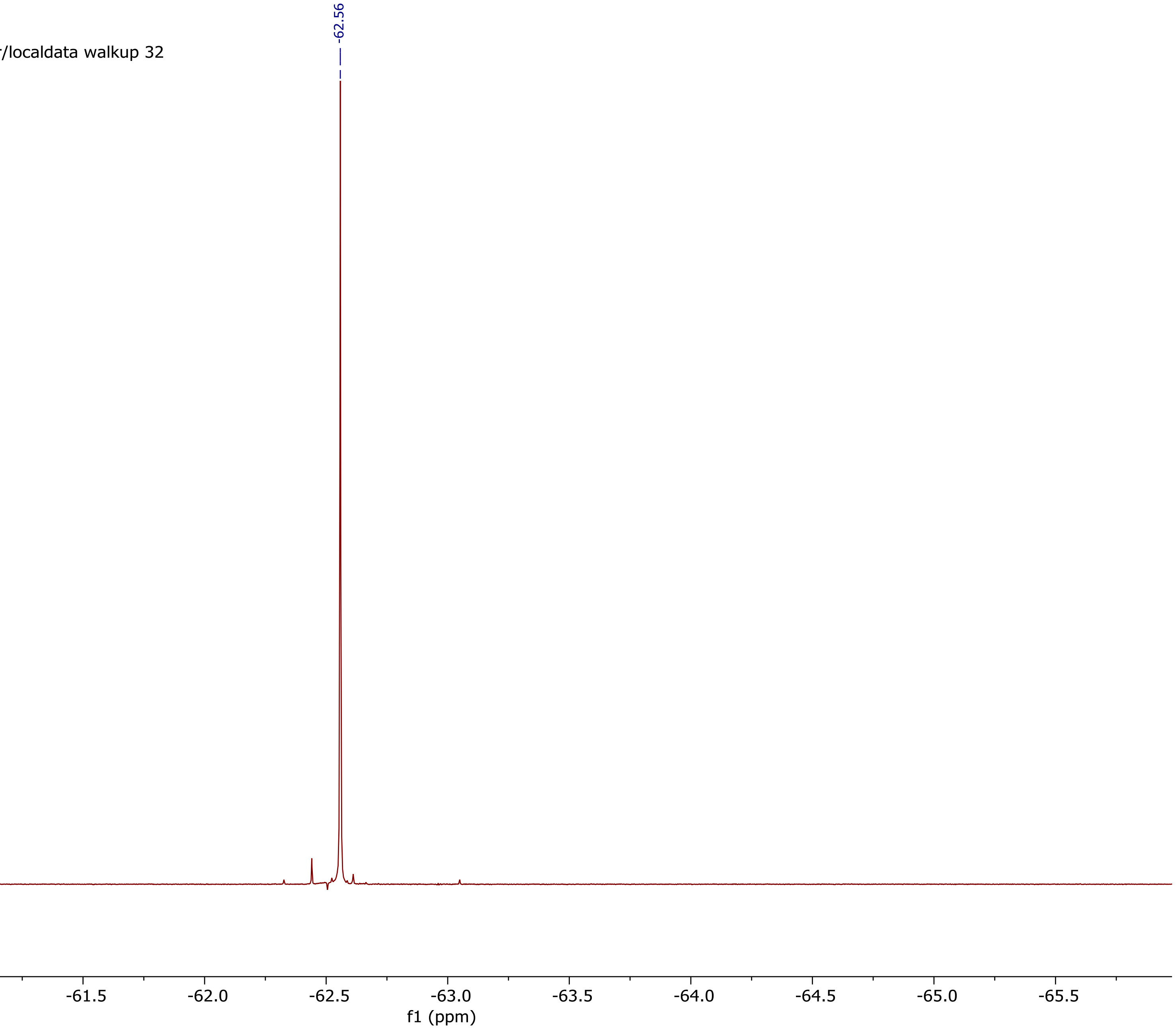
02111733.13.1.1r
SLC:WB:WB 7-14-5
F19_limits_dec.dur CDCl3 /home/nmr/localdata walkup 32
-62.56
-60.0
-60.5
-61.0
-61.5
-62.0
-62.5
-63.0
-63.5
-64.0
-64.5
-65.0
-65.5
f1 (ppm)

07120235.14.fid
SLC:WDGB:WB7-17-5
Carbon.dur CDCl3 /home/nmr/localdata walkup 33

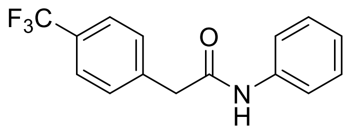

4s
101 MHz

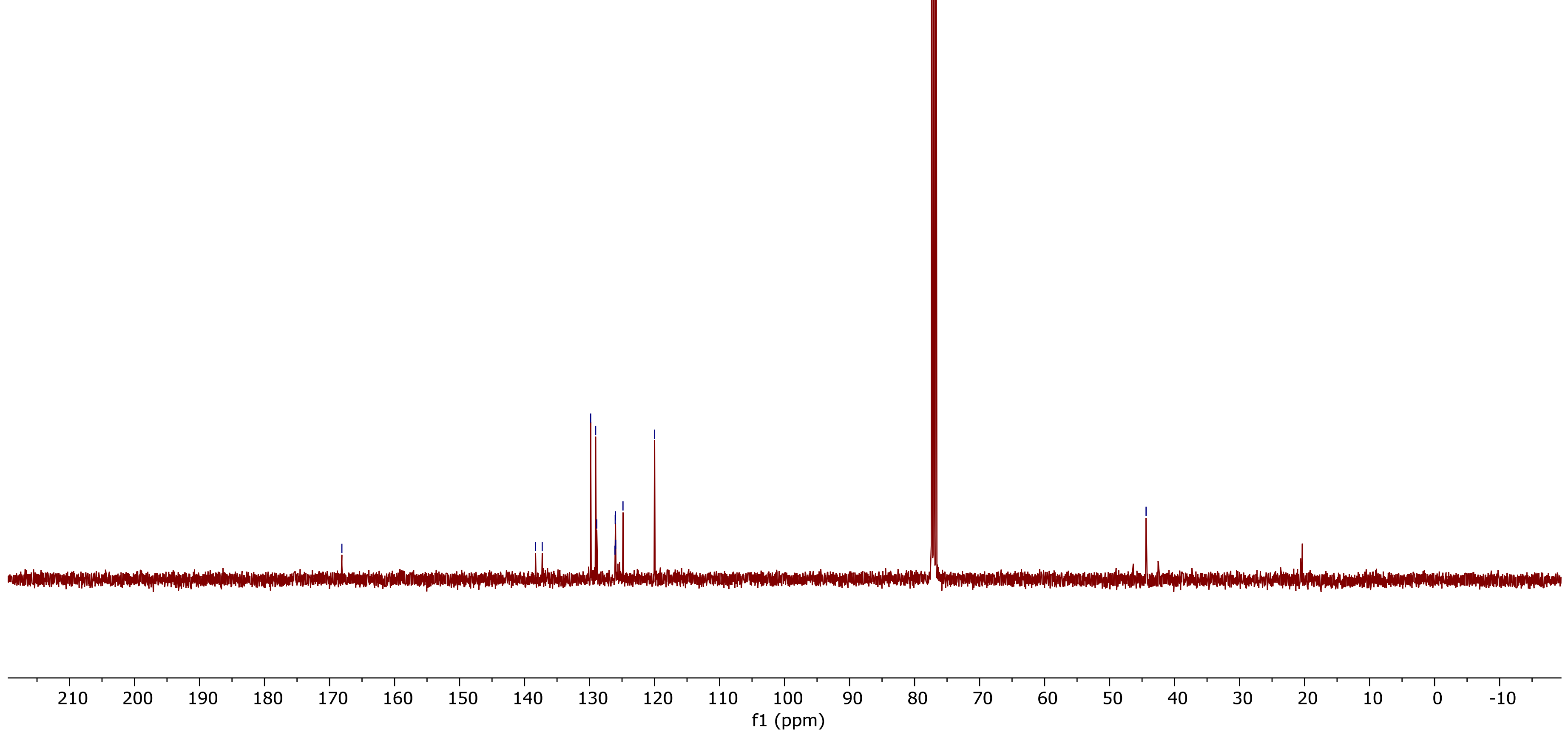

06132011.10.fid
SLC:WDGB:WB7-25-1
Proton.dur CDCl3 /home/nmr/localdata walkup 21

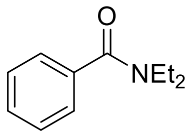

4t
400 MHz

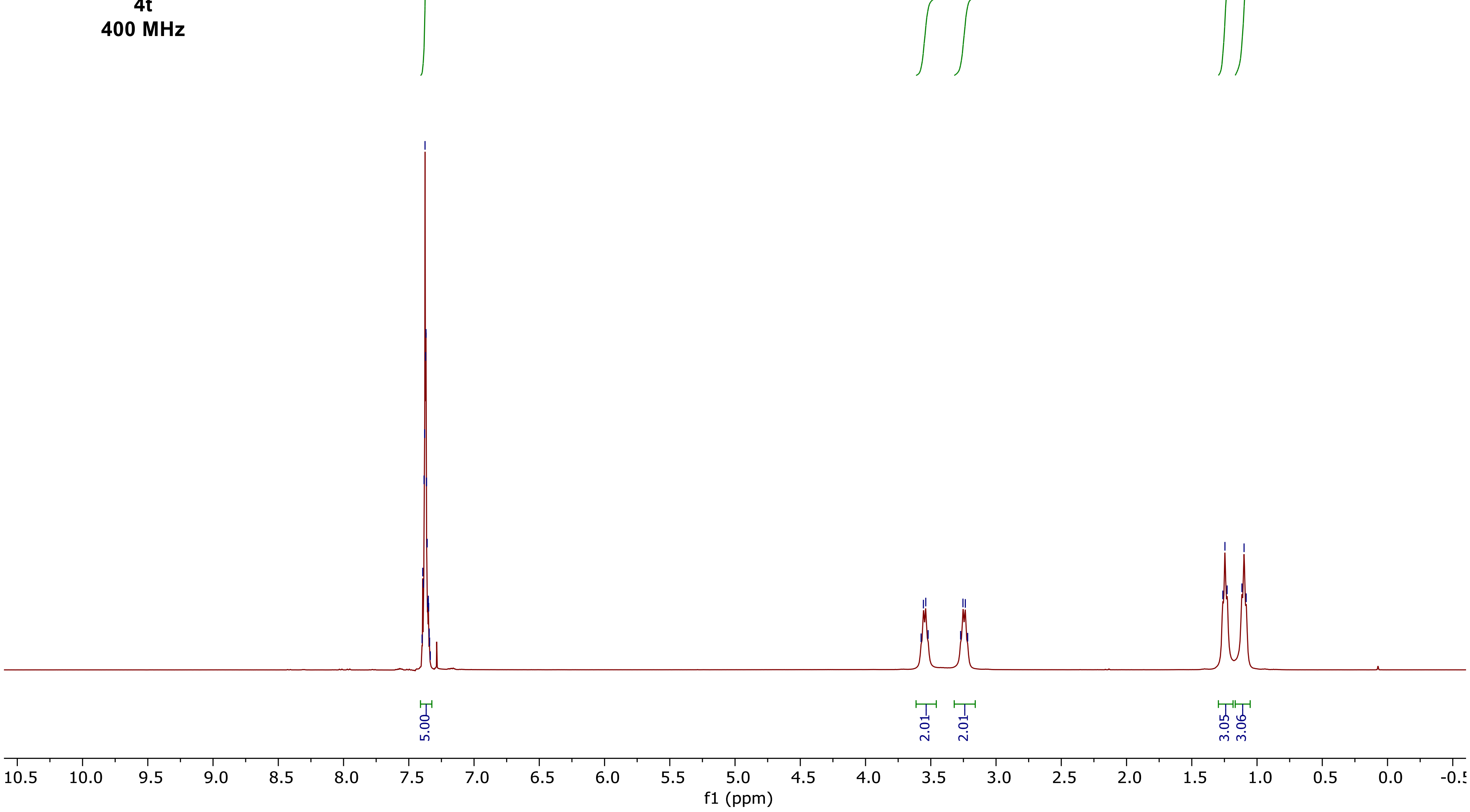

06132011.11.fid
SLC:WDGB:WB7-25-1
Carbon.dur CDCl3 /home/nmr/localdata walkup 21

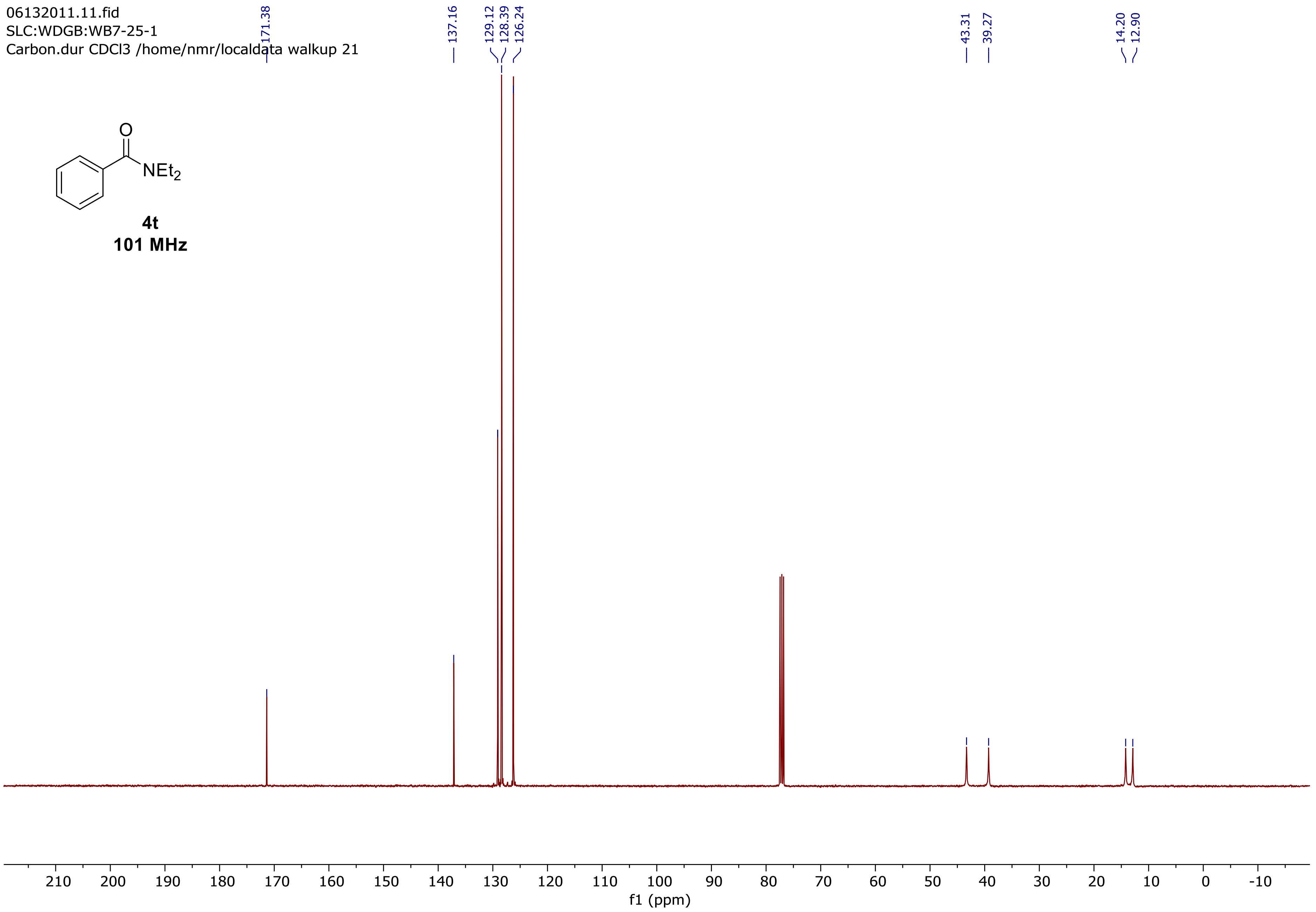

06110444.10.fid
SLC:WDGB:WB7-25-2
Proton.dur CDCl3 /home/nmr/localdata walkup 34

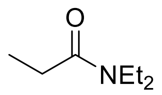

4u
400 MHz

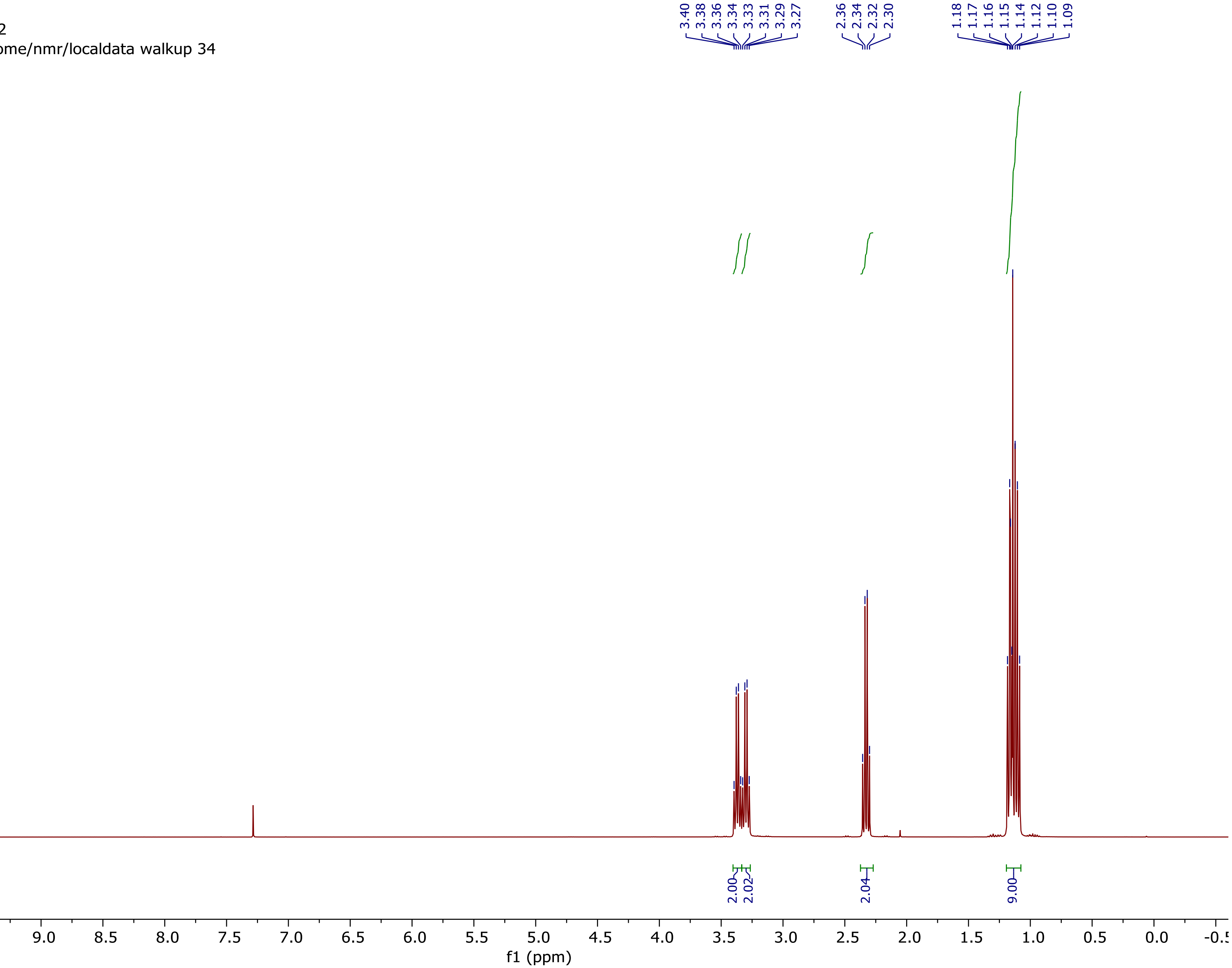

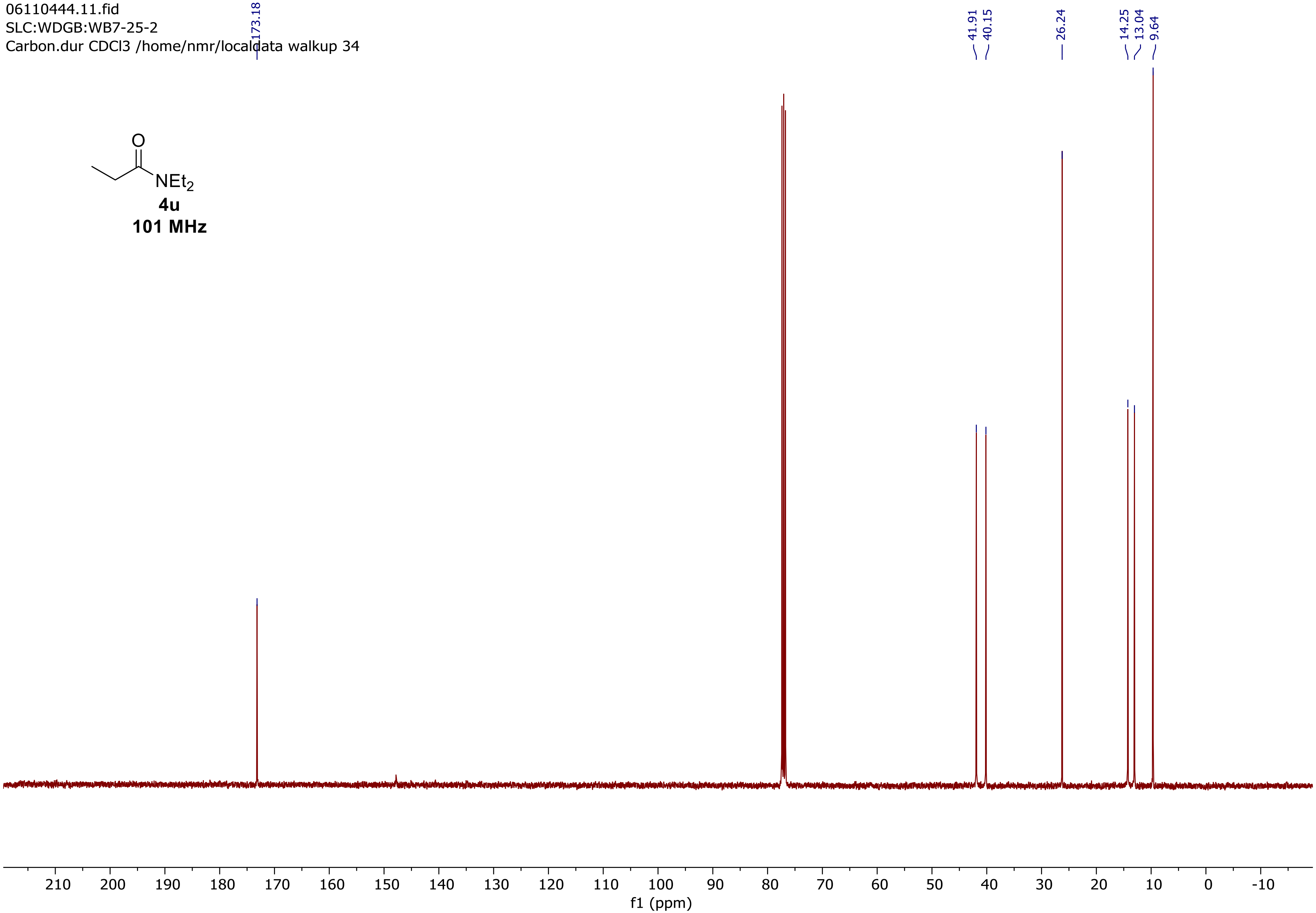
06110444.11.fid
SLC:WDGB:WB7-25-2
Carbon.dur CDCl3 /home/nmr/localdata walkup 34
173.18
41.91
40.15
26.24
14.25
13.04
9.64
O
NEt2
4u
101 MHz
210 200 190 180 170 160 150 140 130 120 110 100 90 80 70 60 50 40 30 20 10 0 -10
f1 (ppm)

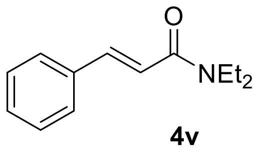

4v
400 MHz

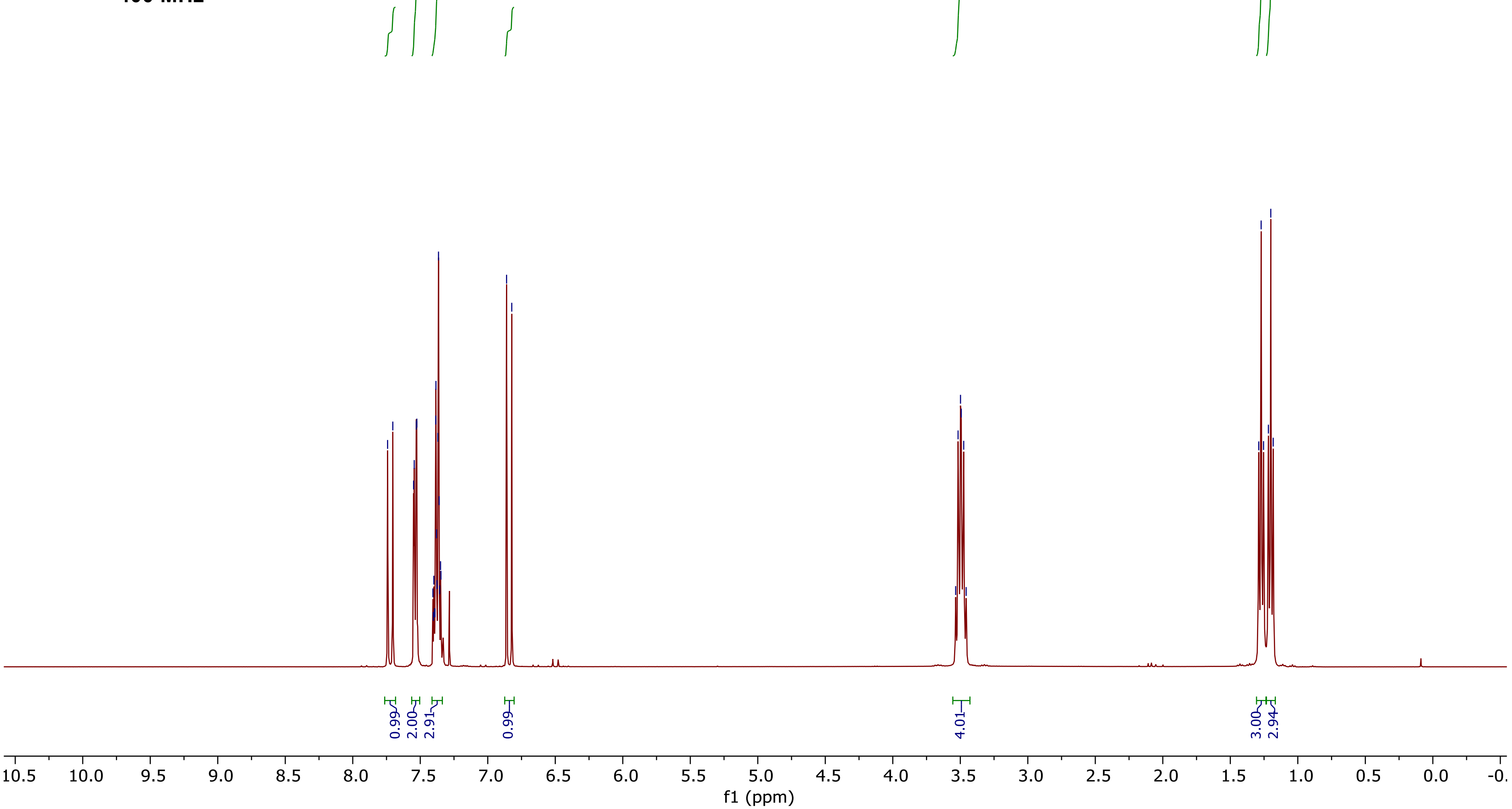

06110406.10.fid
SLC:WDGB:WB7-25-4
Proton.dur CDCl3 /home/nmr/localdata walkup 32
7.74 7.70 7.55 7.54 7.53 7.53 7.41 7.40 7.40 7.39 7.39 7.39 7.38 7.38 7.38 7.37 7.36 7.36 7.36 7.35 7.35 6.86 6.82
3.53 3.52 3.50 3.49 3.47 3.46
1.29 1.27 1.25 1.22 1.20 1.18
0.99 2.00 2.91 0.99 4.01 3.00 2.94
10.5 10.0 9.5 9.0 8.5 8.0 7.5 7.0 6.5 6.0 5.5 5.0 4.5 4.0 3.5 3.0 2.5 2.0 1.5 1.0 0.5 0.0 -0.
f1 (ppm)

06110406.11.fid
SLC:WDGB:WB7-25-4
Carbon.dur CDCl3 /home/nmr/localdata walkup 32

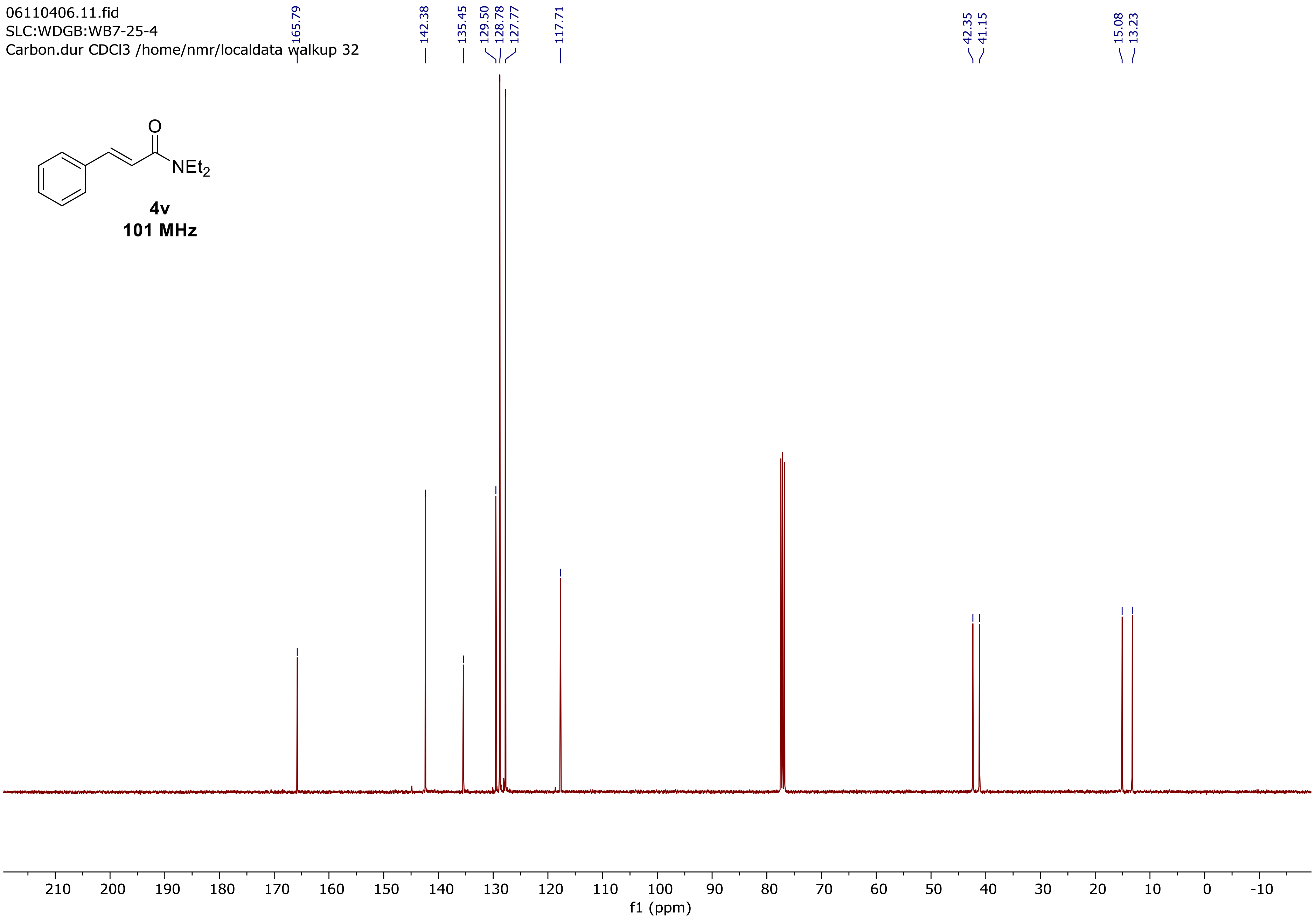

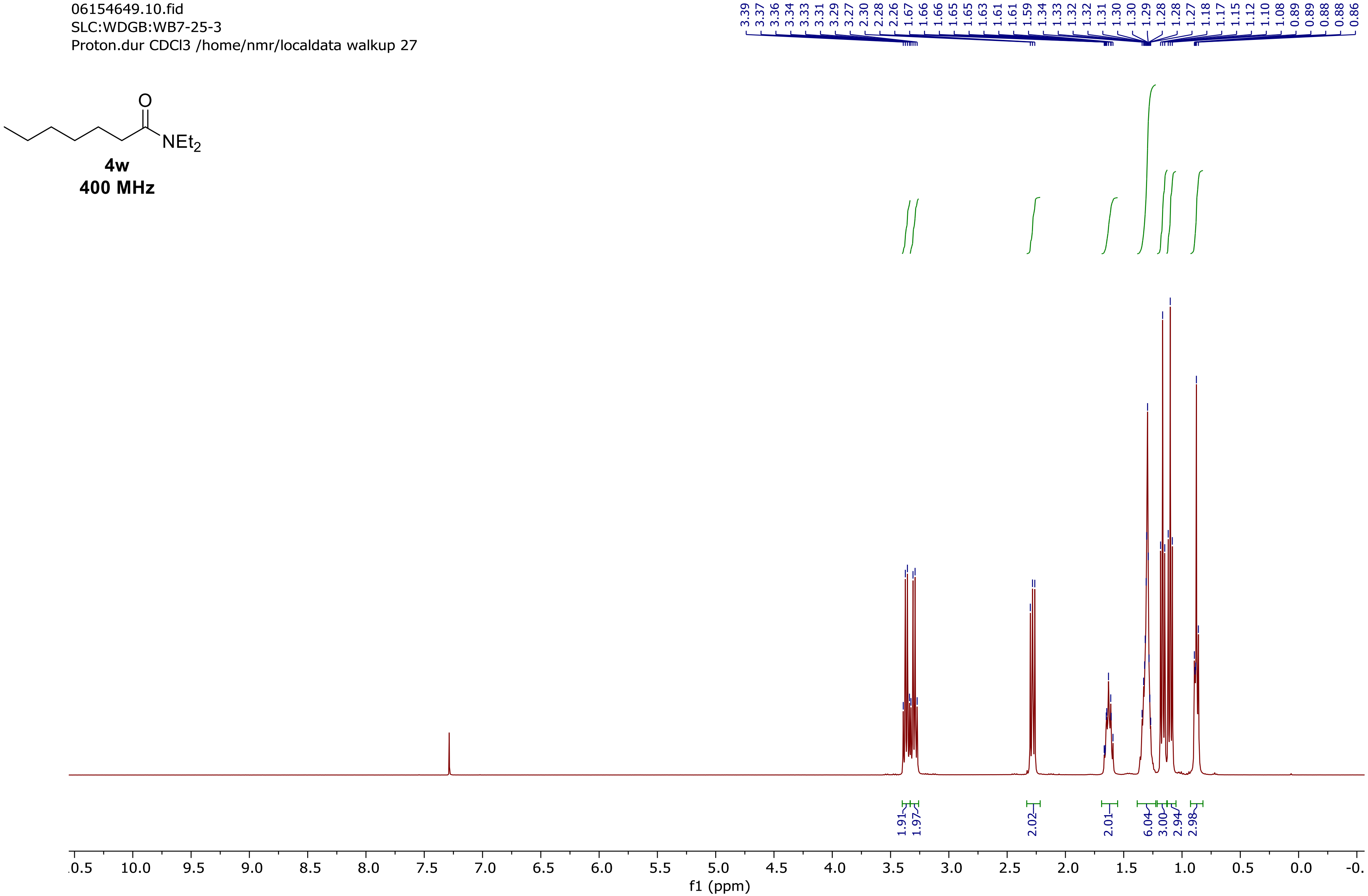
O
NEt2
4w
400 MHz
06154649.10.fid
SLC:WDGB:WB7-25-3
Proton.dur CDCl3 /home/nmr/localdata walkup 27
3.39 3.37 3.36 3.34 3.33 3.31 3.29 3.27 2.30 2.28 2.26 1.67 1.66 1.65 1.63 1.61 1.61 1.59 1.34 1.33 1.32 1.32 1.31 1.30 1.29 1.28 1.28 1.27 1.18 1.17 1.15 1.12 1.10 1.08 0.89 0.89 0.88 0.88 0.86
1.91
1.97
2.02
2.01
6.04
3.00
2.94
2.98
f1 (ppm)

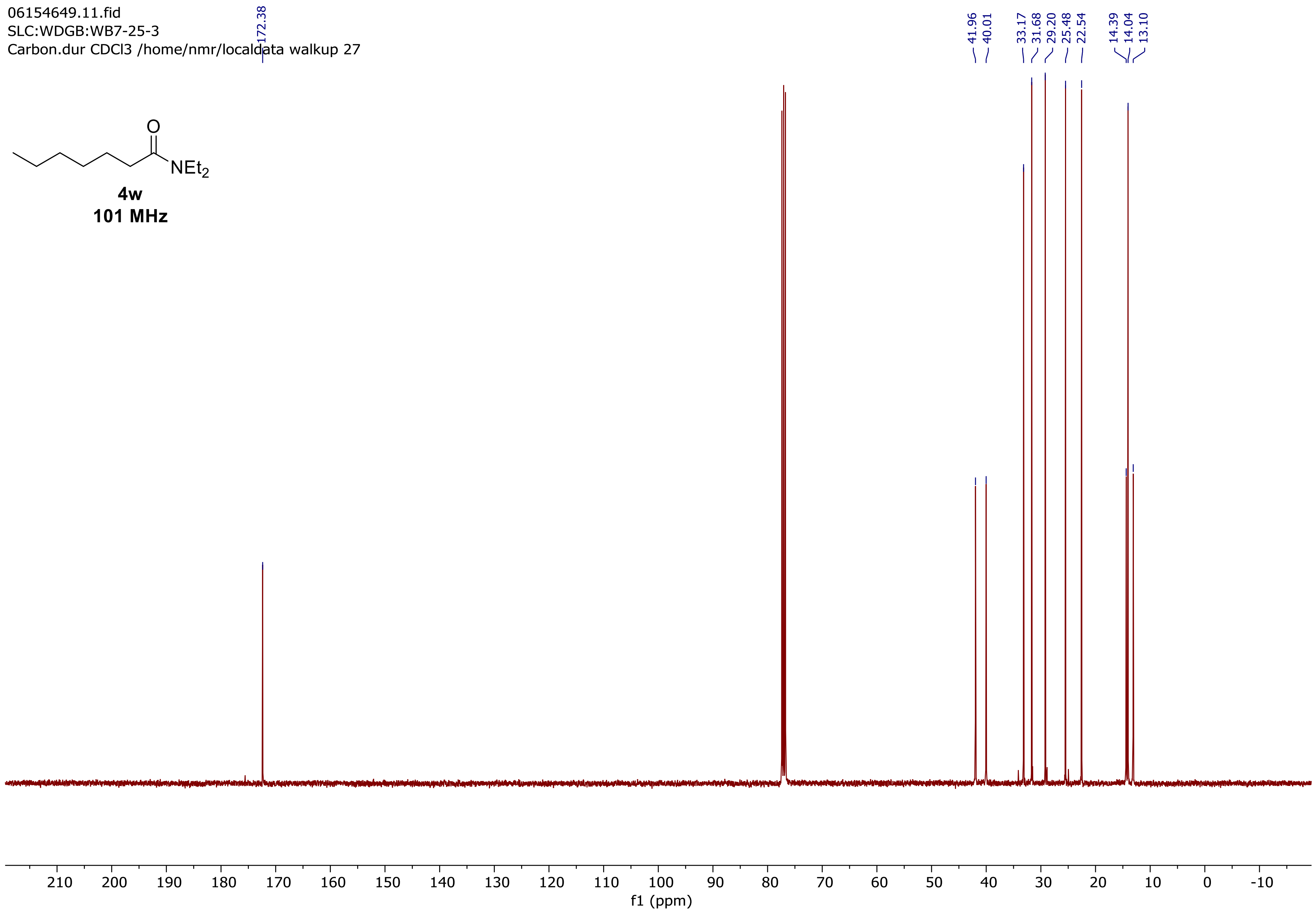
06154649.11.fid
SLC:WDGB:WB7-25-3
Carbon.dur CDCl3 /home/nmr/localdata walkup 27
172.38
41.96
40.01
33.17
31.68
29.20
25.48
22.54
14.39
14.04
13.10
O
NEt2
4w
101 MHz
210 200 190 180 170 160 150 140 130 120 110 100 90 80 70 60 50 40 30 20 10 0 -10
f1 (ppm)

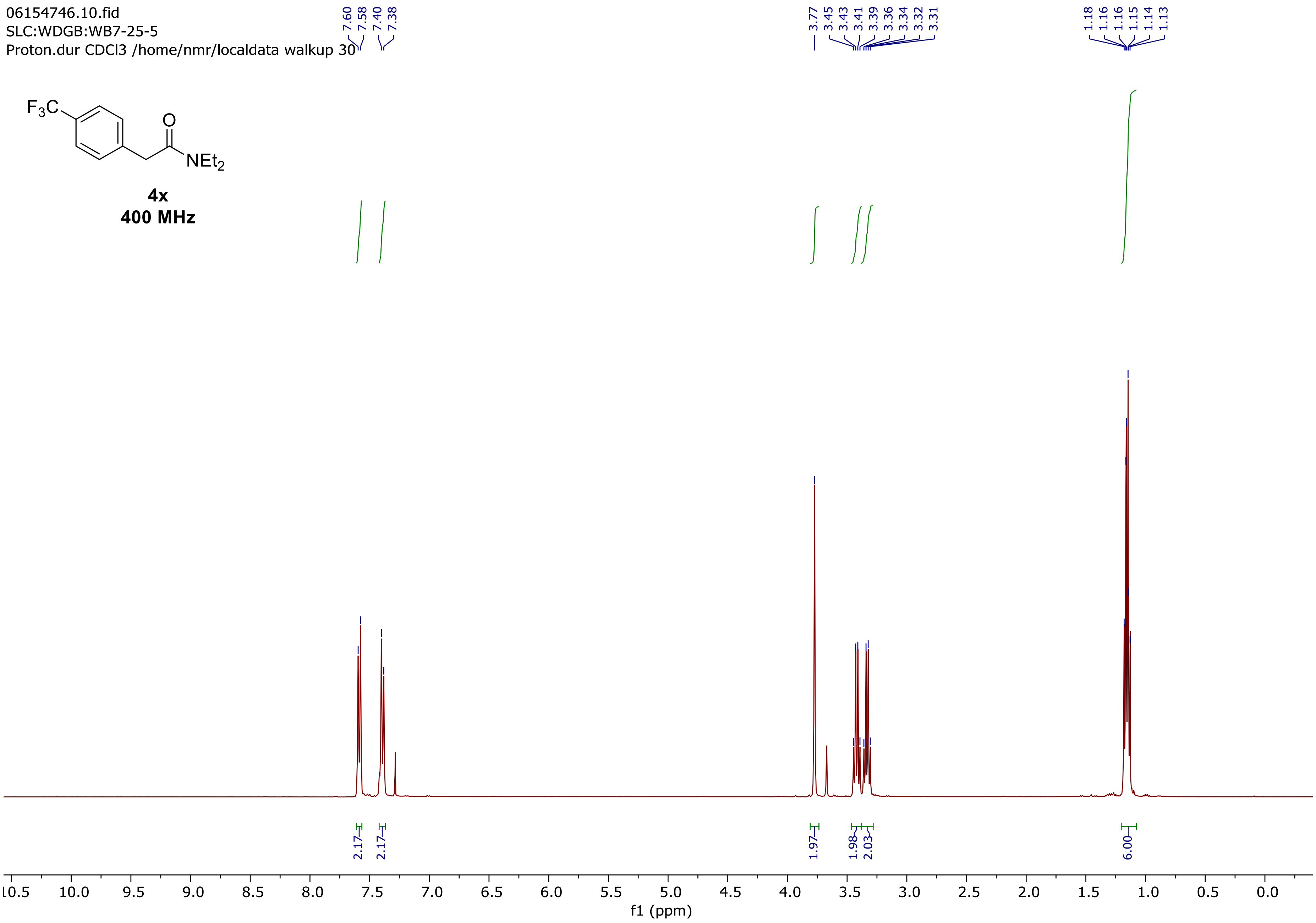
06154746.10.fid
SLC:WDGB:WB7-25-5
Proton.dur CDCl3 /home/nmr/localdata walkup 30
F3C
O
NEt2
4x
400 MHz
7.60
7.58
7.40
7.38
3.77
3.45
3.43
3.41
3.39
3.36
3.34
3.32
3.31
1.18
1.16
1.16
1.15
1.14
1.13
2.17
2.17
1.97
1.98
2.03
6.00
f1 (ppm)

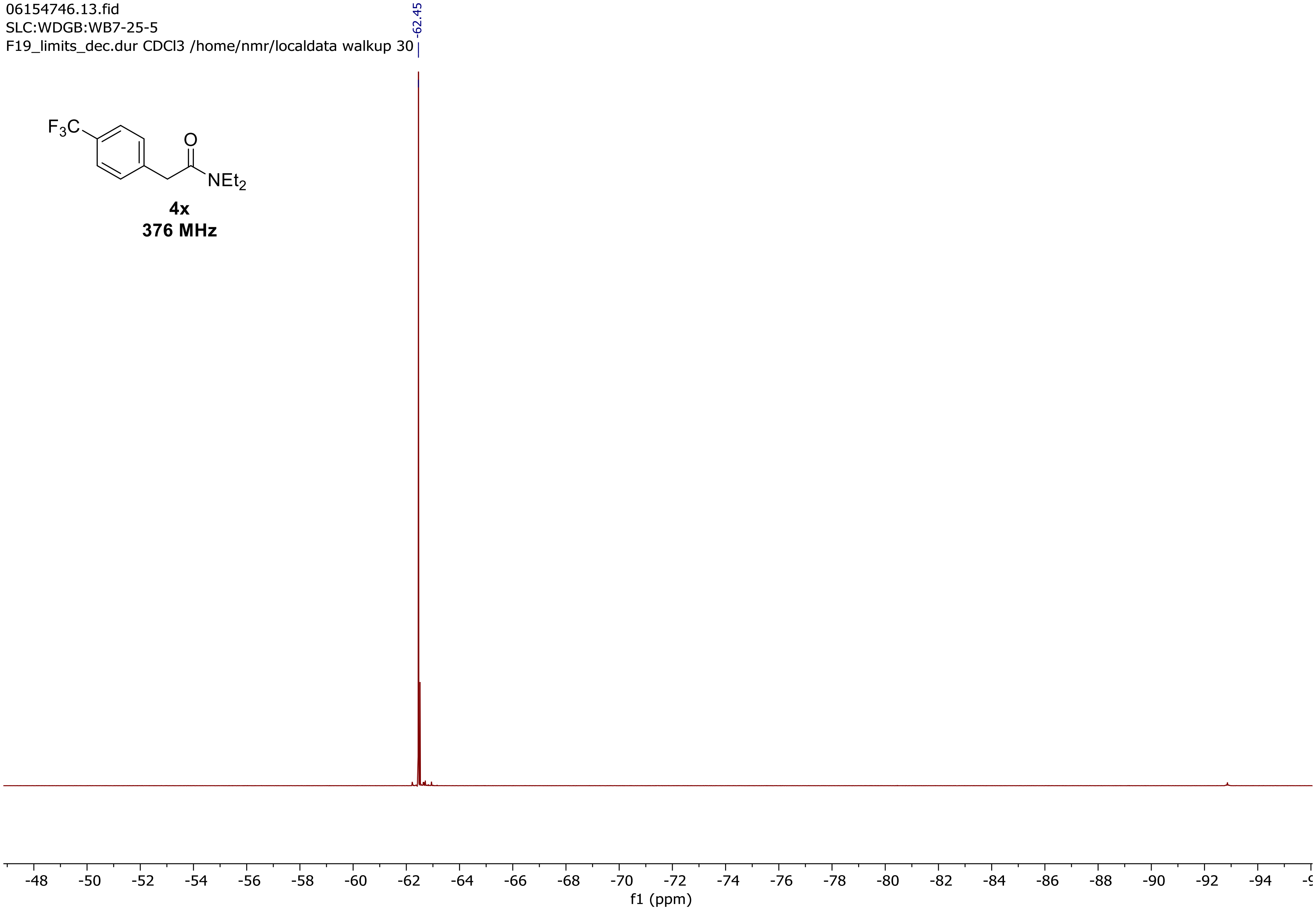
06154746.13.fid
SLC:WDGB:WB7-25-5
F19_limits_dec.dur CDCl3 /home/nmr/localdata walkup 30
-62.45
F3C
O
NEt2
-48 -50 -52 -54 -56 -58 -60 -62 -64 -66 -68 -70 -72 -74 -76 -78 -80 -82 -84 -86 -88 -90 -92 -94
f1 (ppm)

4x
376 MHz

06154746.14.fid
SLC:WDGB:WB7-25-5
Carbon.dur CDCl3 /home/nmr/localdata walkup 30

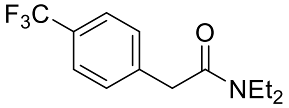

4x
101 MHz

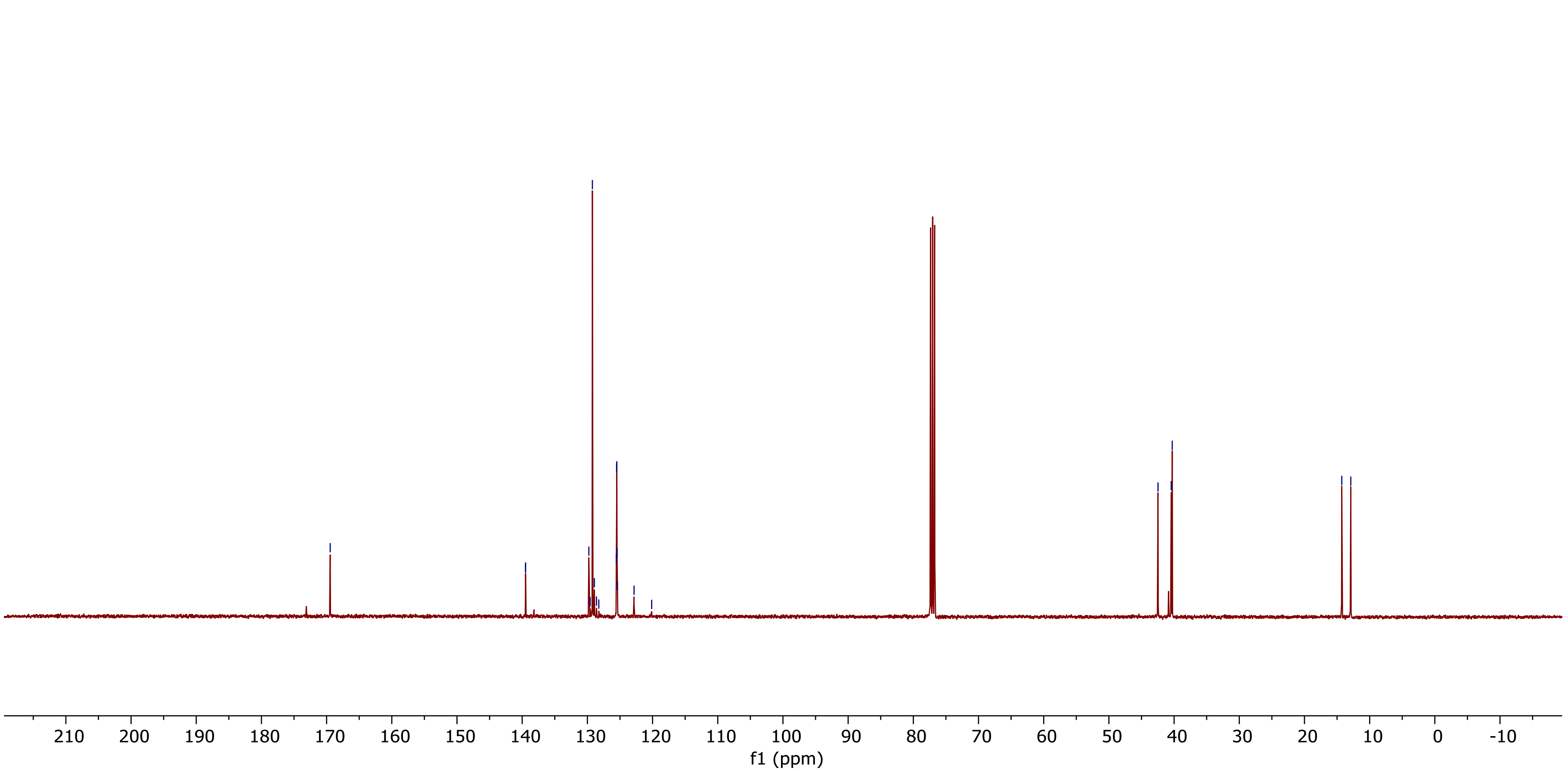

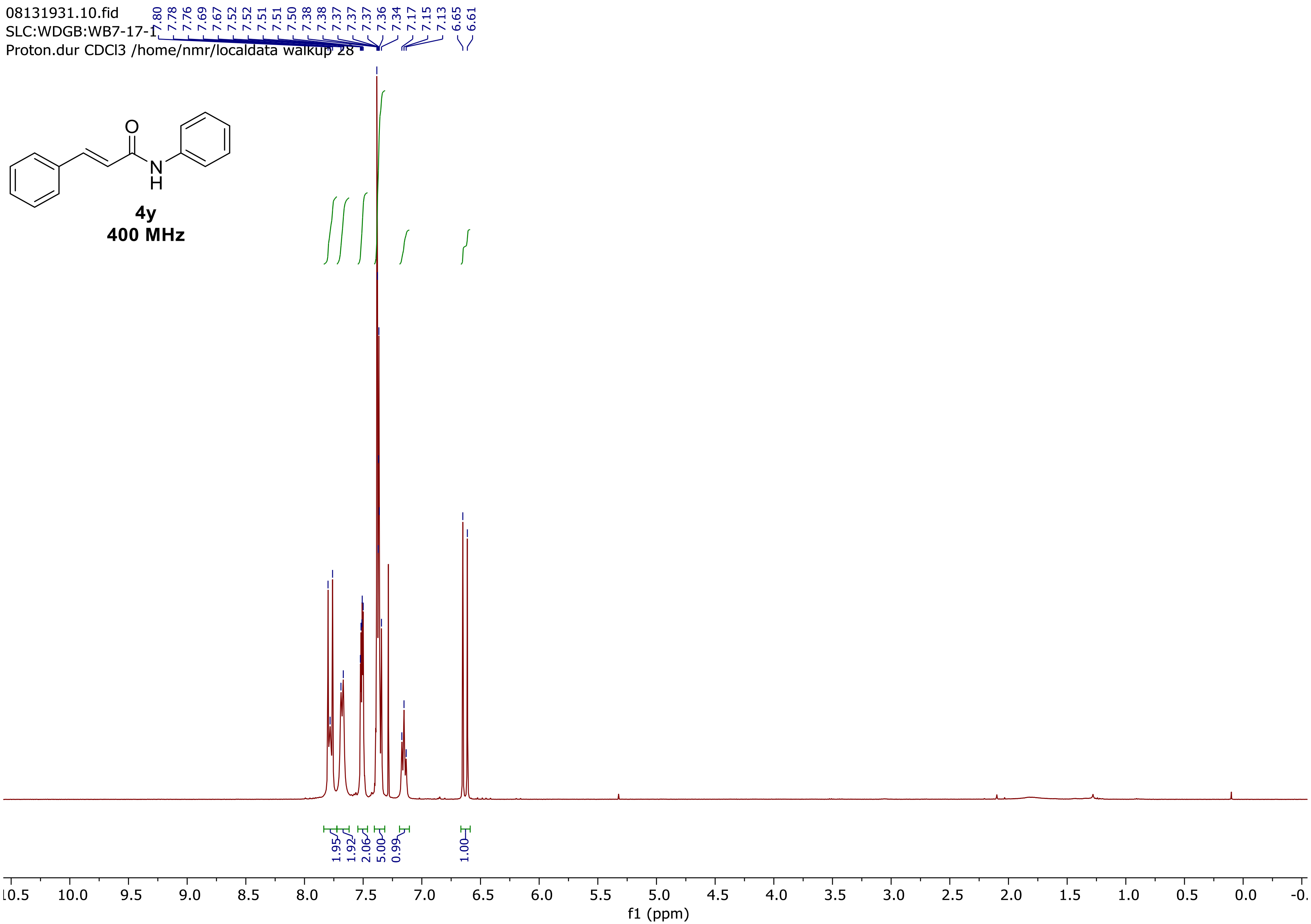
08131931.10.fid
SLC:WDGB:WB7-17-1
Proton.dur CDCl3 /home/nmr/localdata walkup 28
7.80 7.78 7.76 7.69 7.67 7.52 7.52 7.51 7.51 7.50 7.38 7.38 7.37 7.37 7.37 7.36 7.34 7.17 7.15 7.13 6.65 6.61
O
N
H
4y
400 MHz
1.95
1.92
2.06
5.00
0.99
1.00
f1 (ppm)

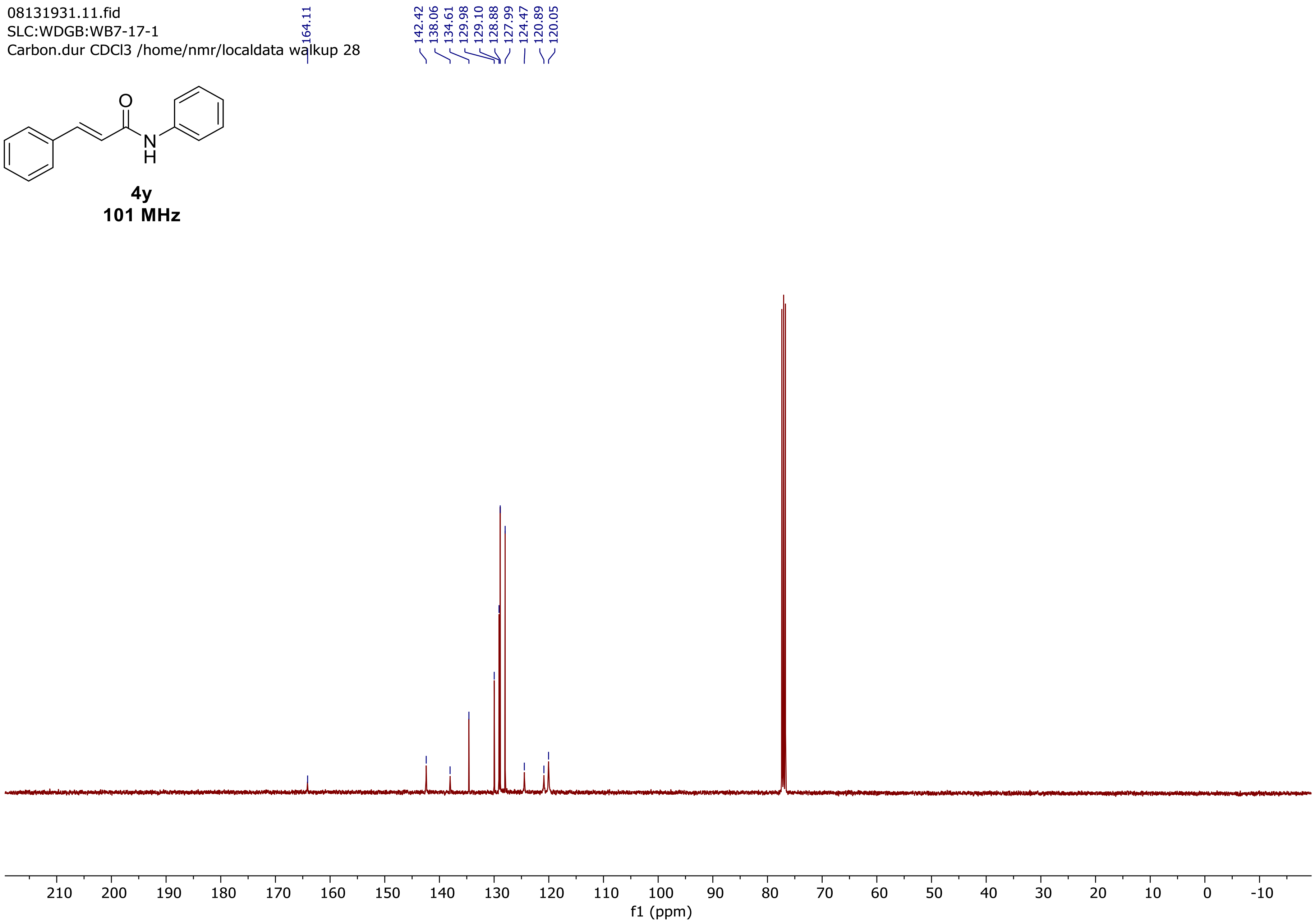
08131931.11.fid
SLC:WDGB:WB7-17-1
Carbon.dur CDCl3 /home/nmr/localdata walkup 28
164.11
142.42
138.06
134.61
129.98
129.10
128.88
127.99
124.47
120.89
120.05
O
N
H
4y
101 MHz
210 200 190 180 170 160 150 140 130 120 110 100 90 80 70 60 50 40 30 20 10 0 -10
f1 (ppm)

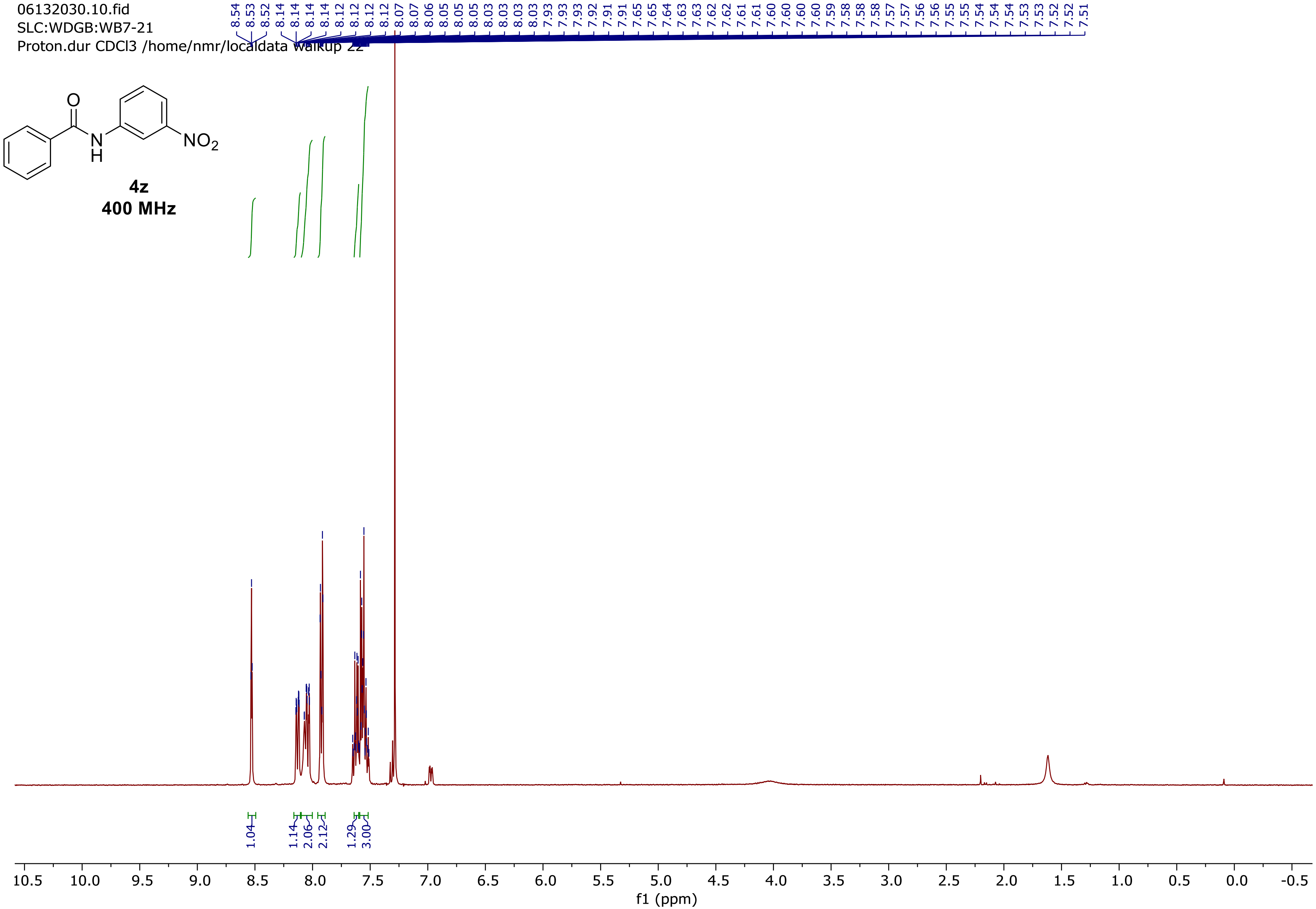
06132030.10.fid
SLC:WDGB:WB7-21
Proton.dur CDCl3 /home/nmr/localdata walkup 22
8.54
8.53
8.52
8.14
8.14
8.14
8.14
8.12
8.12
8.12
8.12
8.07
8.07
8.06
8.05
8.05
8.05
8.03
8.03
8.03
8.03
7.93
7.93
7.93
7.92
7.91
7.91
7.65
7.65
7.64
7.63
7.63
7.62
7.62
7.61
7.61
7.60
7.60
7.60
7.60
7.59
7.58
7.58
7.58
7.57
7.57
7.56
7.56
7.55
7.55
7.54
7.54
7.54
7.53
7.53
7.52
7.52
7.51
O
N
H
NO2
4z
400 MHz
1.04
1.14
2.06
2.12
1.29
3.00
10.5
10.0
9.5
9.0
8.5
8.0
7.5
7.0
6.5
6.0
5.5
5.0
4.5
4.0
3.5
3.0
2.5
2.0
1.5
1.0
0.5
0.0
-0.5
f1 (ppm)

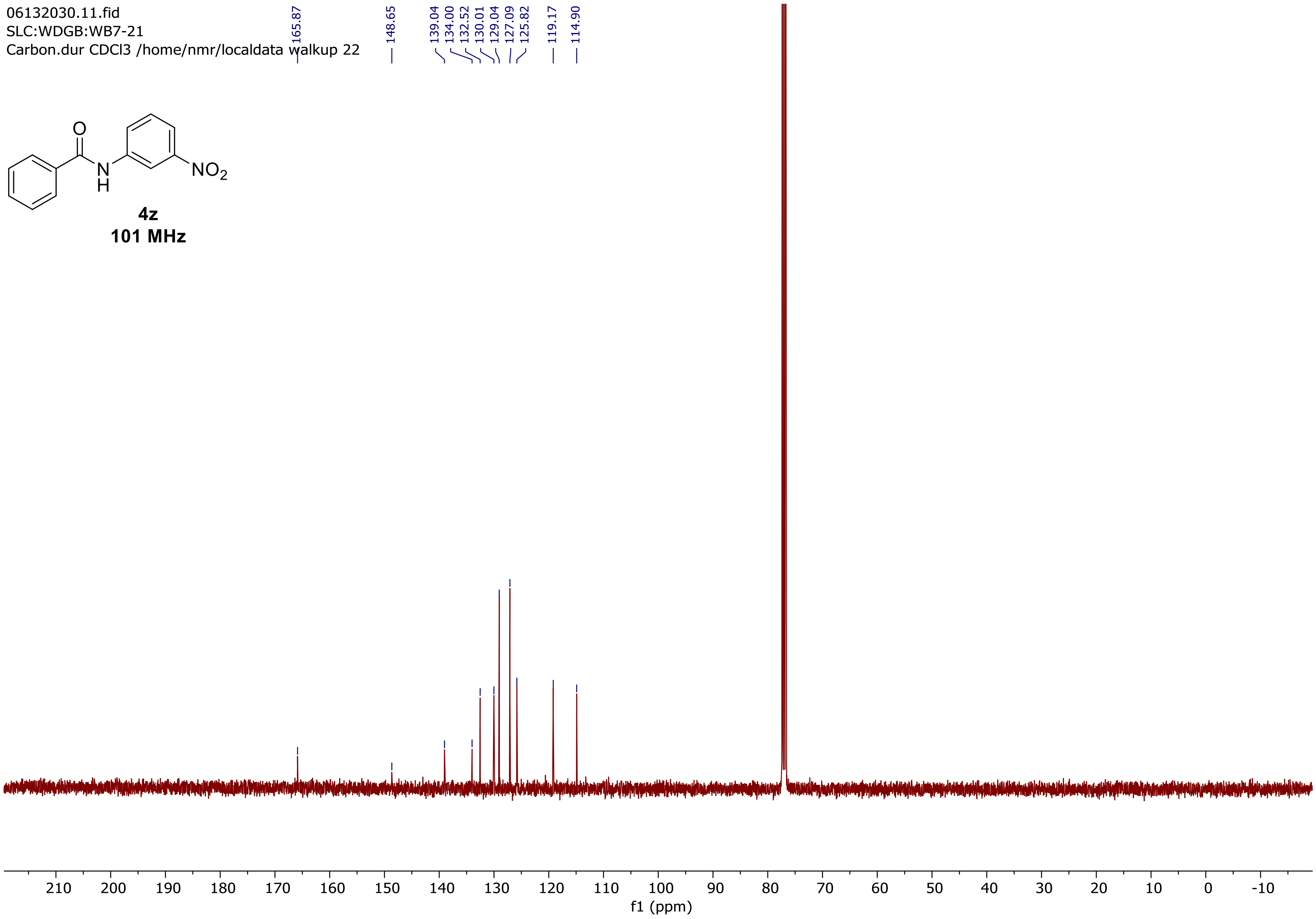
06132030.11.fid
SLC:WDGB:WB7-21
Carbon.dur CDCl3 /home/nmr/localdata walkup 22
165.87
148.65
139.04
134.00
132.52
130.01
129.04
127.09
125.82
119.17
114.90
NO2
N
H
O
4z
101 MHz
210
200
190
180
170
160
150
140
130
120
110
100
90
80
70
60
50
40
30
20
10
0
-10
f1 (ppm)

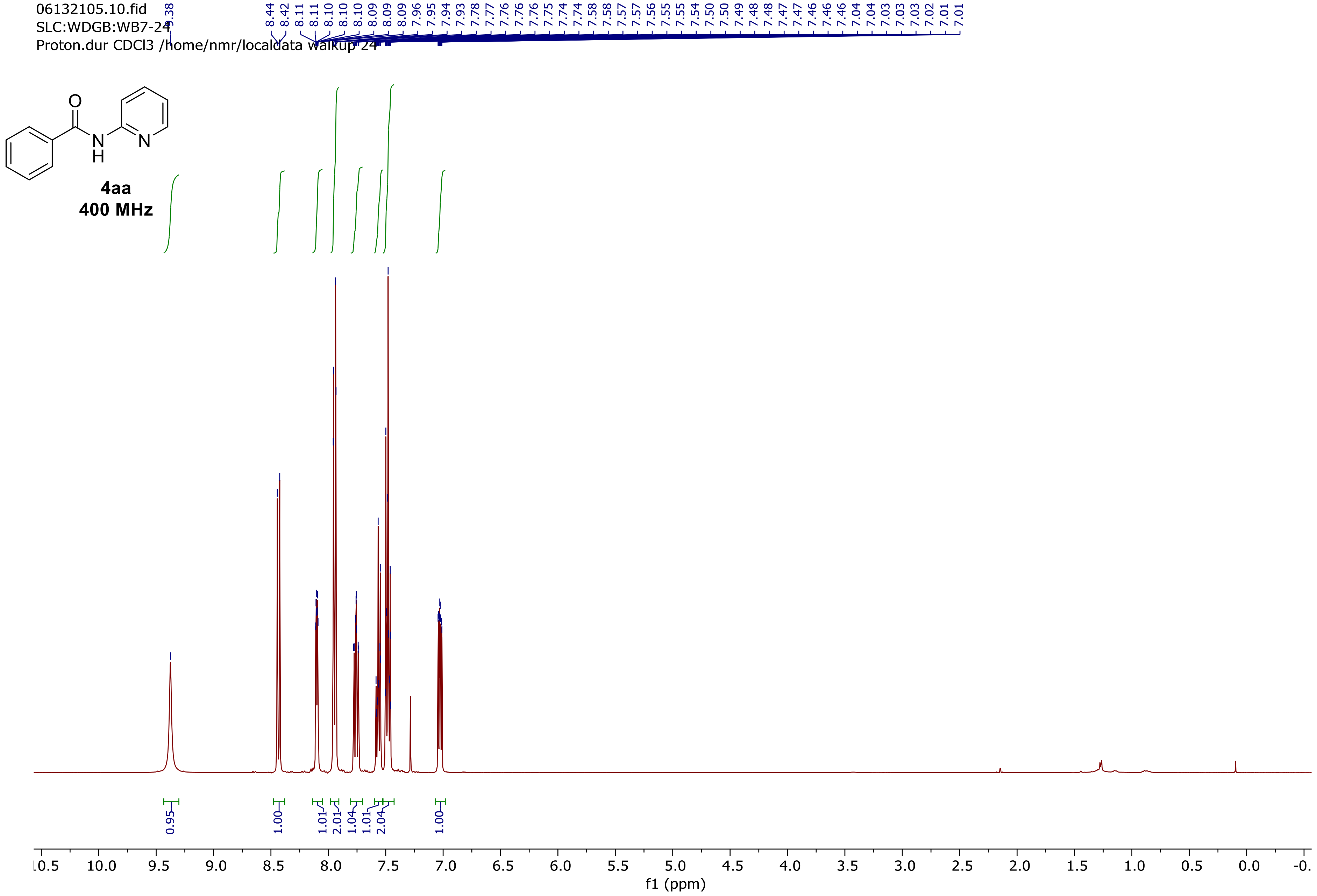
06132105.10.fid
SLC:WDGB:WB7-24
Proton.dur CDCl3 /home/nmr/localdata walkup 24
9.38
8.44 8.42 8.11 8.11 8.10 8.10 8.10 8.09 8.09 8.09 7.96 7.95 7.94 7.93 7.78 7.77 7.76 7.76 7.75 7.74 7.74 7.58 7.58 7.57 7.57 7.56 7.55 7.55 7.54 7.50 7.49 7.48 7.48 7.47 7.47 7.46 7.46 7.46 7.04 7.04 7.03 7.03 7.02 7.01 7.01
4aa
400 MHz
0.95 1.00 1.01 2.01 1.04 1.01 2.04 1.00
f1 (ppm)

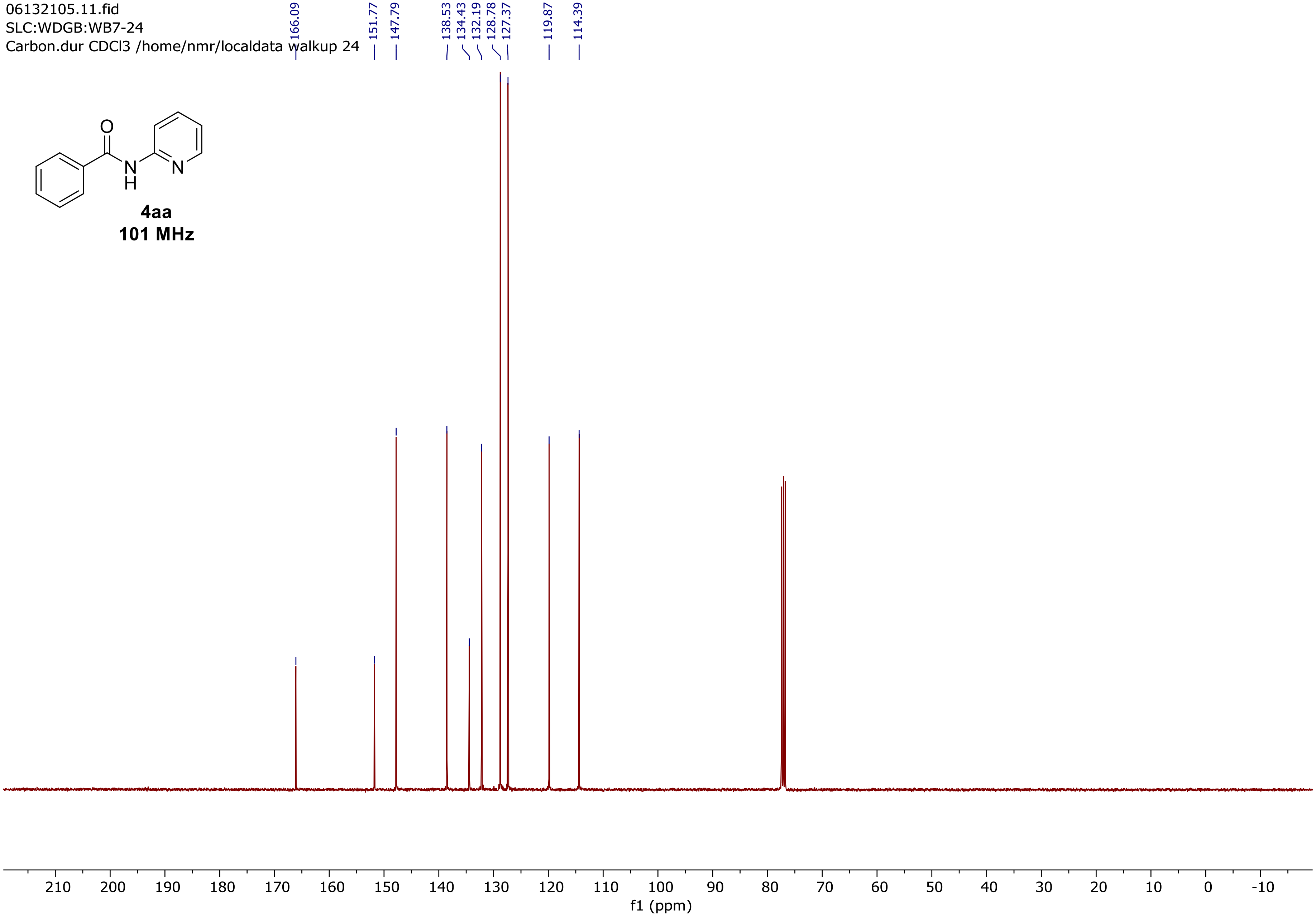
06132105.11.fid
SLC:WDGB:WB7-24
Carbon.dur CDCl3 /home/nmr/localdata walkup 24
166.09
151.77
147.79
138.53
134.43
132.19
128.78
127.37
119.87
114.39
O
N
H
N
4aa
101 MHz
210
200
190
180
170
160
150
140
130
120
110
100
90
80
70
60
50
40
30
20
10
0
-10
f1 (ppm)

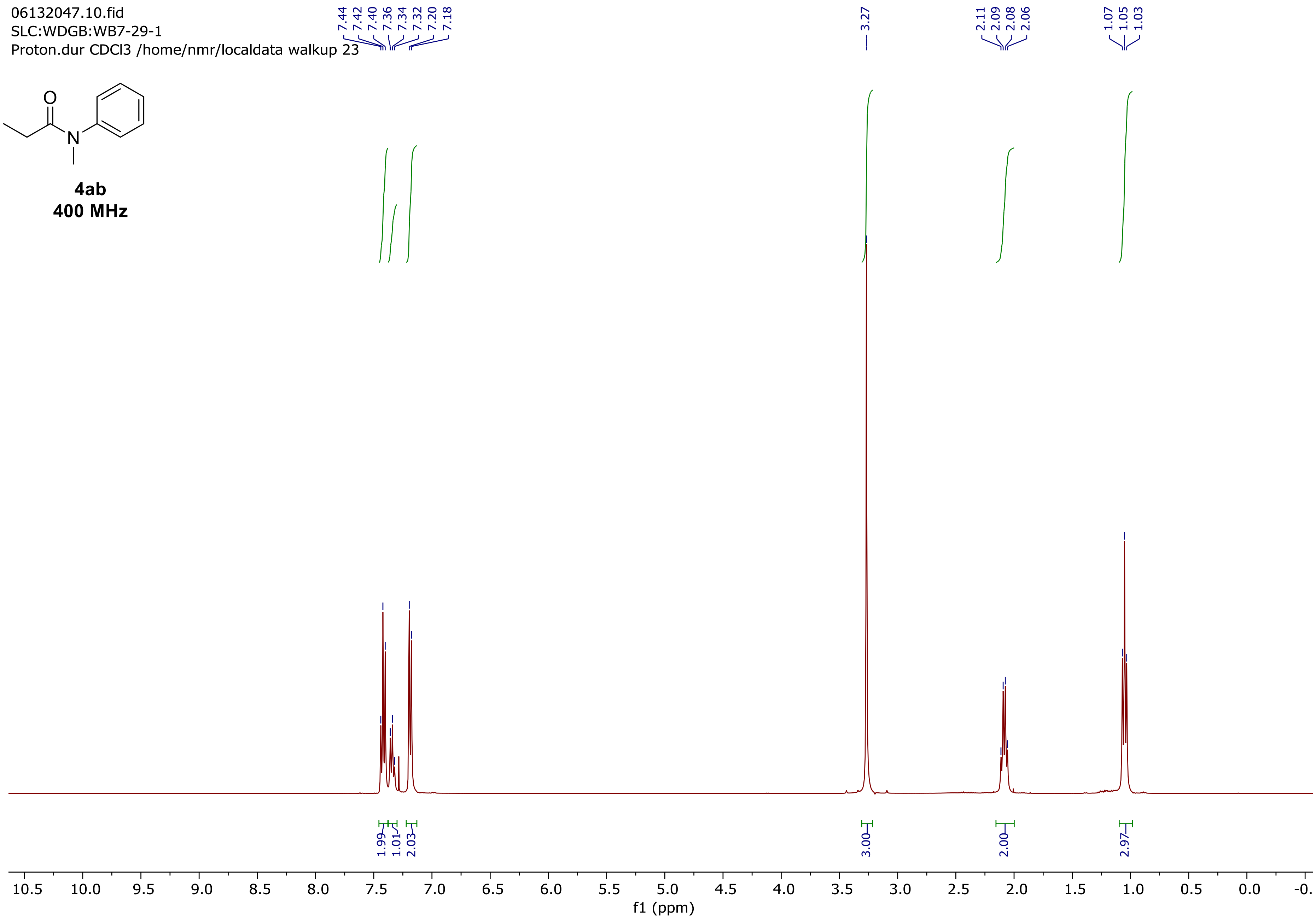
06132047.10.fid
SLC:WDGB:WB7-29-1
Proton.dur CDCl3 /home/nmr/localdata walkup 23
4ab
400 MHz
7.44
7.42
7.40
7.36
7.34
7.32
7.20
7.18
3.27
2.11
2.09
2.08
2.06
1.07
1.05
1.03
1.99
1.01
2.03
3.00
2.00
2.97
10.5 10.0 9.5 9.0 8.5 8.0 7.5 7.0 6.5 6.0 5.5 5.0 4.5 4.0 3.5 3.0 2.5 2.0 1.5 1.0 0.5 0.0 -0.
f1 (ppm)

06132047.11.fid
SLC:WDGB:WB7-29-1
Carbon.dur CDCl3 /home/nmr/localdata walkup 23

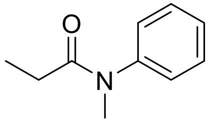

4ab
101 MHz

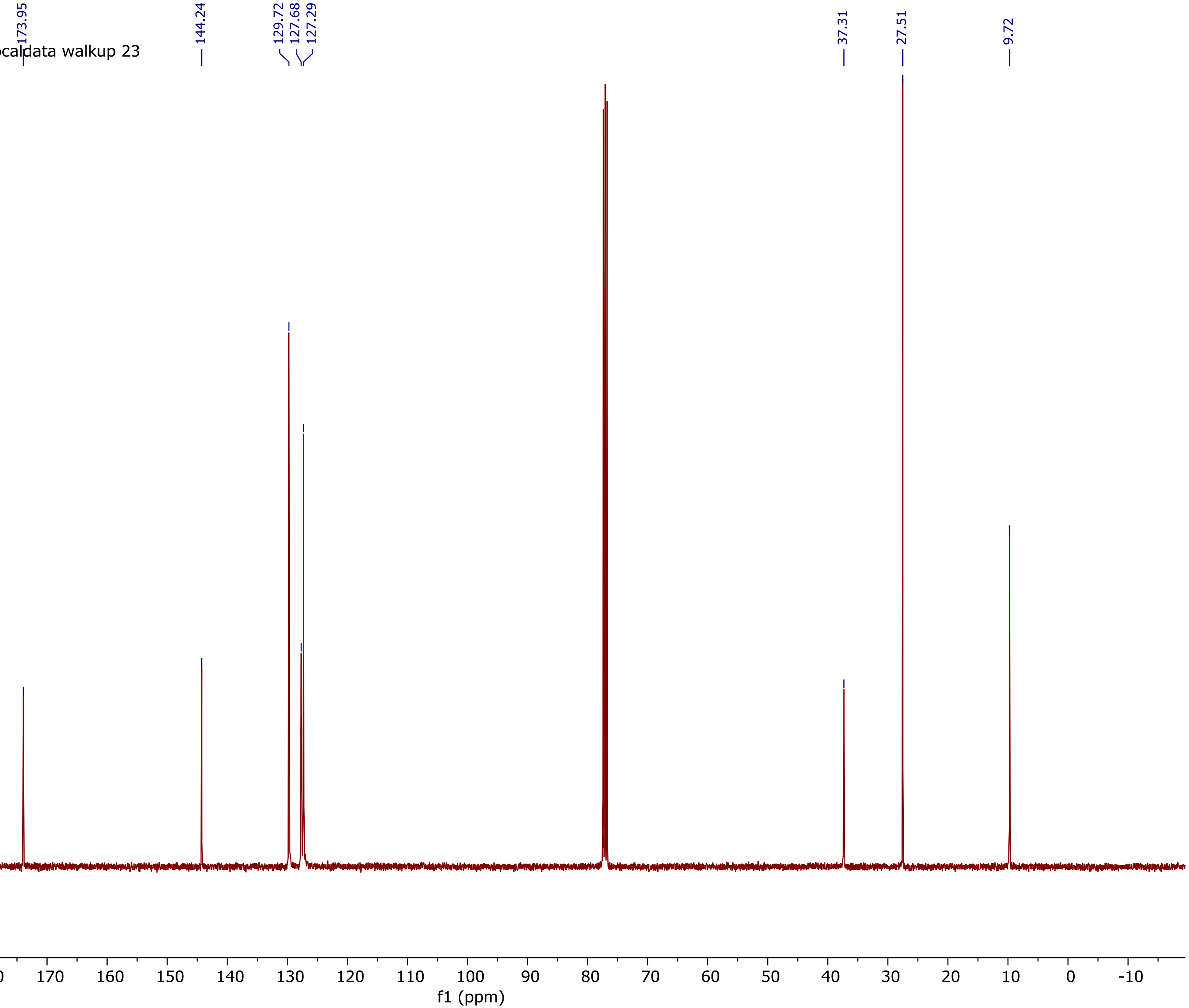

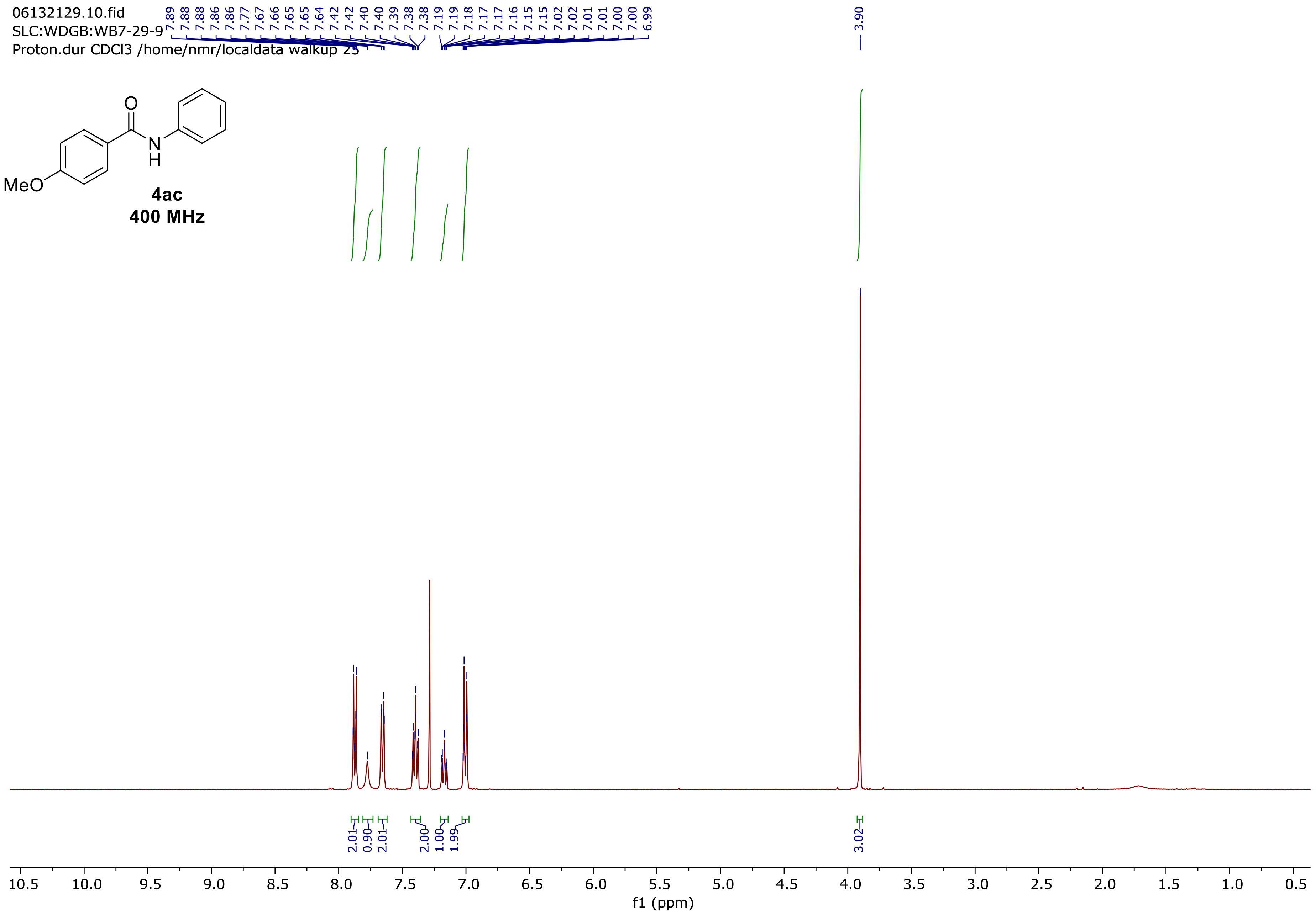
06132129.10.fid
SLC:WDGB:WB7-29-9
Proton.dur CDCl3 /home/nmr/localdata walkup 25
7.89 7.88 7.88 7.86 7.86 7.77 7.67 7.66 7.65 7.65 7.64 7.42 7.42 7.40 7.40 7.39 7.38 7.38 7.19 7.19 7.18 7.17 7.17 7.16 7.15 7.15 7.02 7.02 7.01 7.01 7.00 7.00 6.99
3.90
O
N
H
MeO
4ac
400 MHz
2.01
0.90
2.01
2.00
1.00
1.99
3.02
10.5 10.0 9.5 9.0 8.5 8.0 7.5 7.0 6.5 6.0 5.5 5.0 4.5 4.0 3.5 3.0 2.5 2.0 1.5 1.0 0.5
f1 (ppm)

06132129.11.fid
SLC:WDGB:WB7-29-9
Carbon.dur CDCl3 /home/nmr/localdata walkup 25

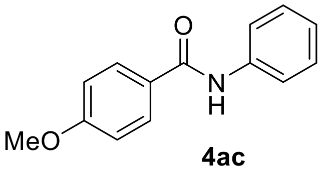

4ac
101 MHz

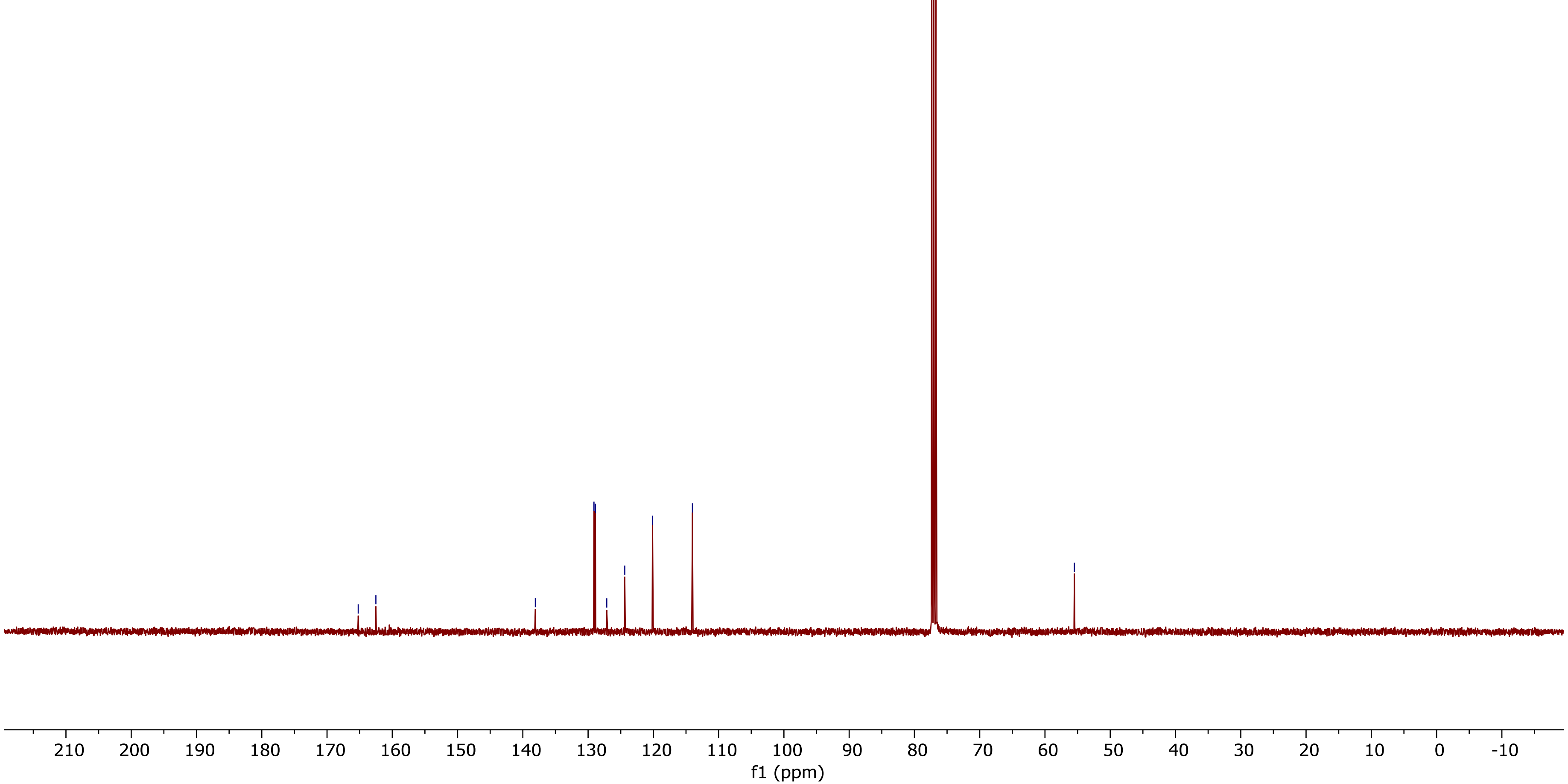

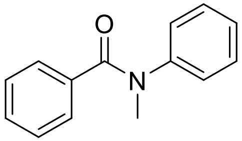

4ad
400 MHz

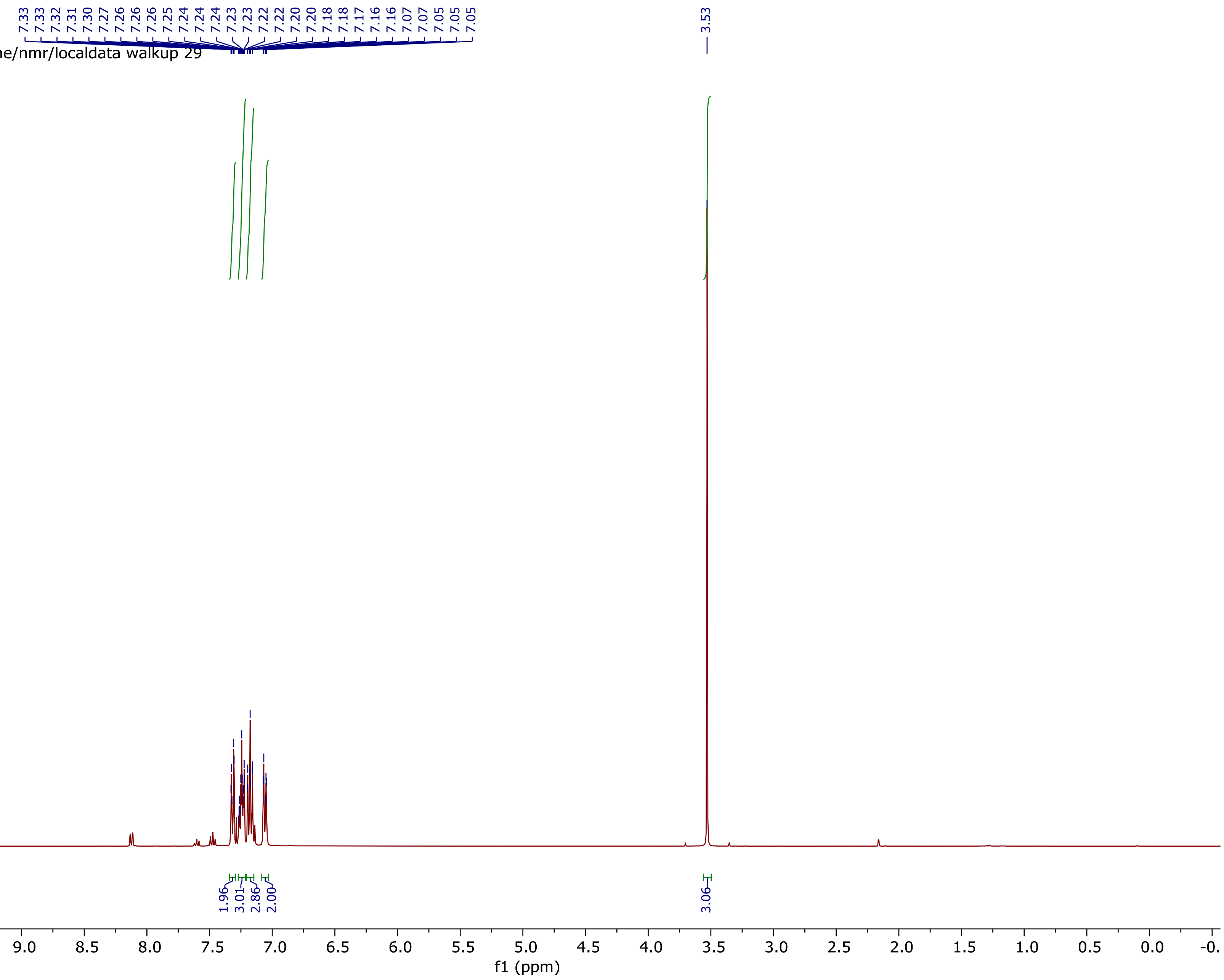

06154729.10.fid
SLC:WDGB:WB7-29-2
Proton.dur CDCl3 /home/nmr/localdata walkup 29
7.33 7.33 7.32 7.31 7.30 7.27 7.26 7.26 7.26 7.25 7.24 7.24 7.24 7.23 7.23 7.22 7.22 7.20 7.18 7.18 7.17 7.16 7.16 7.07 7.07 7.05 7.05 7.05
3.53
1.96
3.01
2.86
2.00
3.06
f1 (ppm)

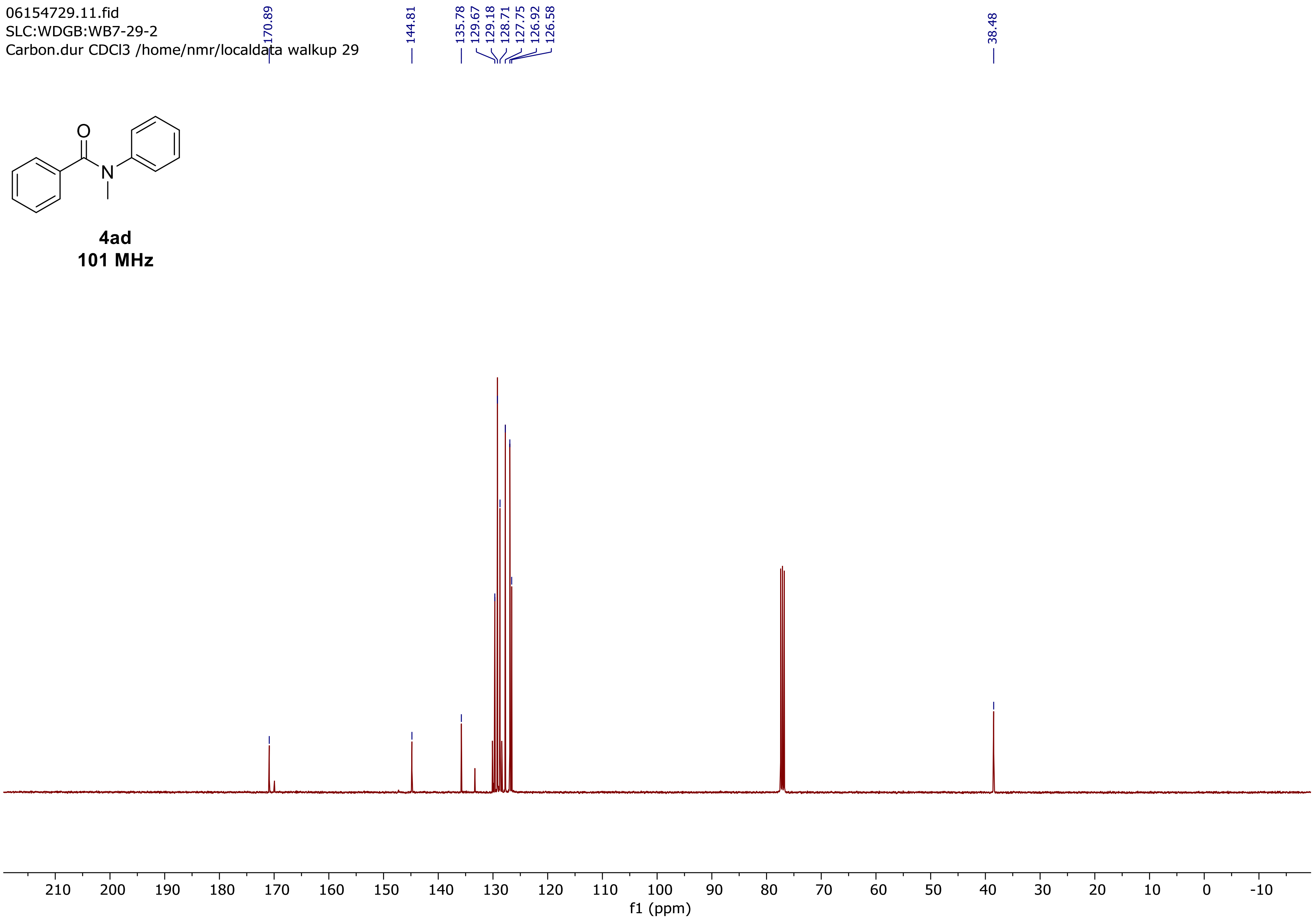
06154729.11.fid
SLC:WDGB:WB7-29-2
Carbon.dur CDCl3 /home/nmr/localdata walkup 29
170.89
144.81
135.78
129.67
129.18
128.71
127.75
126.92
126.58
38.48
O
N
4ad
101 MHz
210 200 190 180 170 160 150 140 130 120 110 100 90 80 70 60 50 40 30 20 10 0 -10
f1 (ppm)

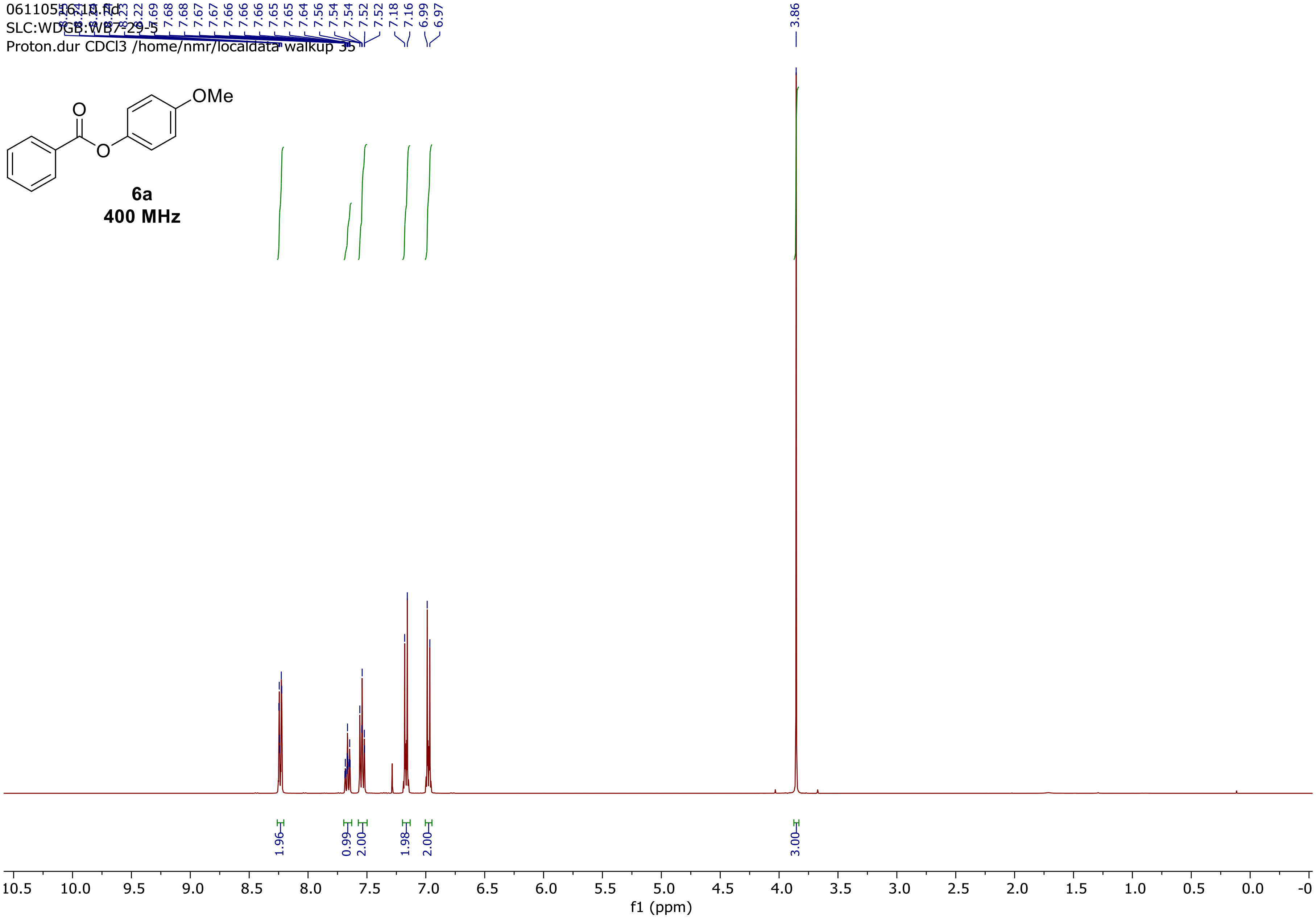
Proton.dur CDCl3 /home/nmr/localdata walkup 35
8.22
7.69
7.68
7.68
7.67
7.67
7.66
7.66
7.66
7.65
7.65
7.64
7.56
7.54
7.54
7.52
7.52
7.18
7.16
6.99
6.97
3.86
OMe
O
O
6a
400 MHz
1.96
0.99
2.00
1.98
2.00
3.00
10.5
10.0
9.5
9.0
8.5
8.0
7.5
7.0
6.5
6.0
5.5
5.0
4.5
4.0
3.5
3.0
2.5
2.0
1.5
1.0
0.5
0.0
f1 (ppm)

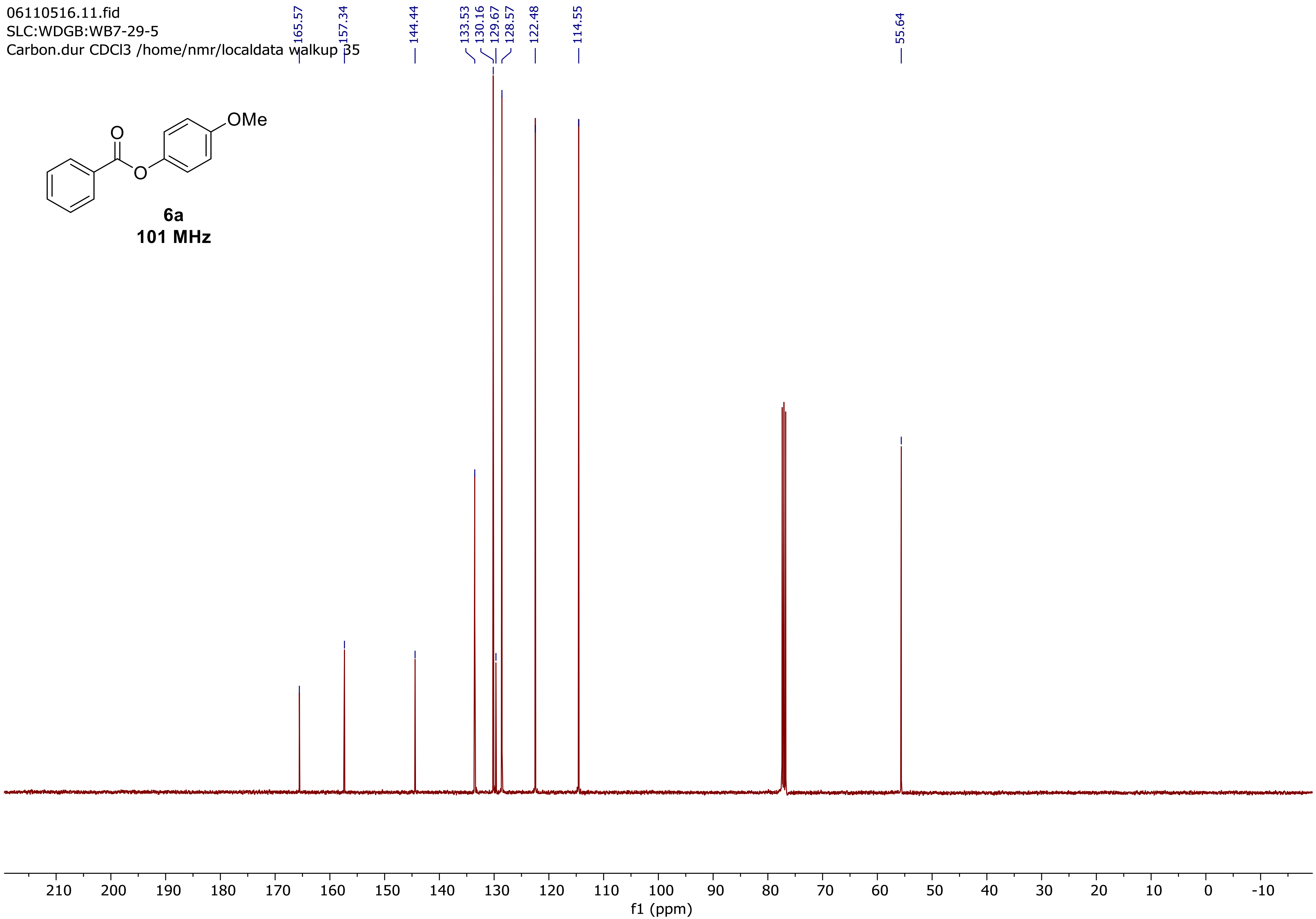
06110516.11.fid
SLC:WDGB:WB7-29-5
Carbon.dur CDCl3 /home/nmr/localdata walkup 35
165.57
157.34
144.44
133.53
130.16
129.67
128.57
122.48
114.55
55.64
OMe
O
O
6a
101 MHz
210 200 190 180 170 160 150 140 130 120 110 100 90 80 70 60 50 40 30 20 10 0 -10
f1 (ppm)

06110543.10.fid
SLC:WDGB:WB7-29-8
Proton.dur CDCl3 /home/nmr/localdata walkup 36

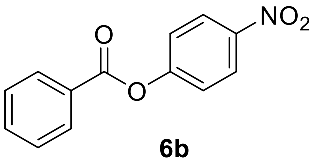

6b
400 MHz

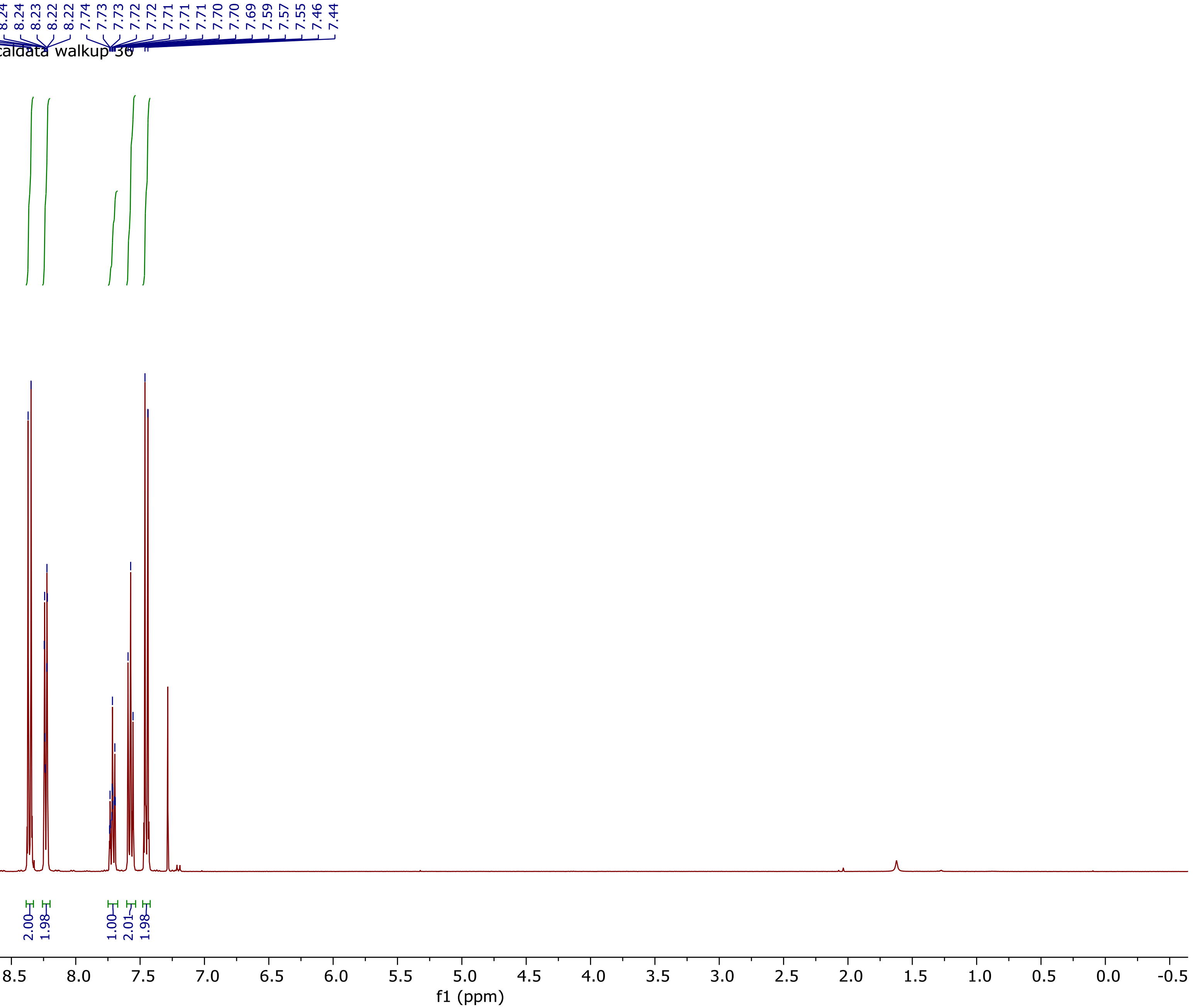

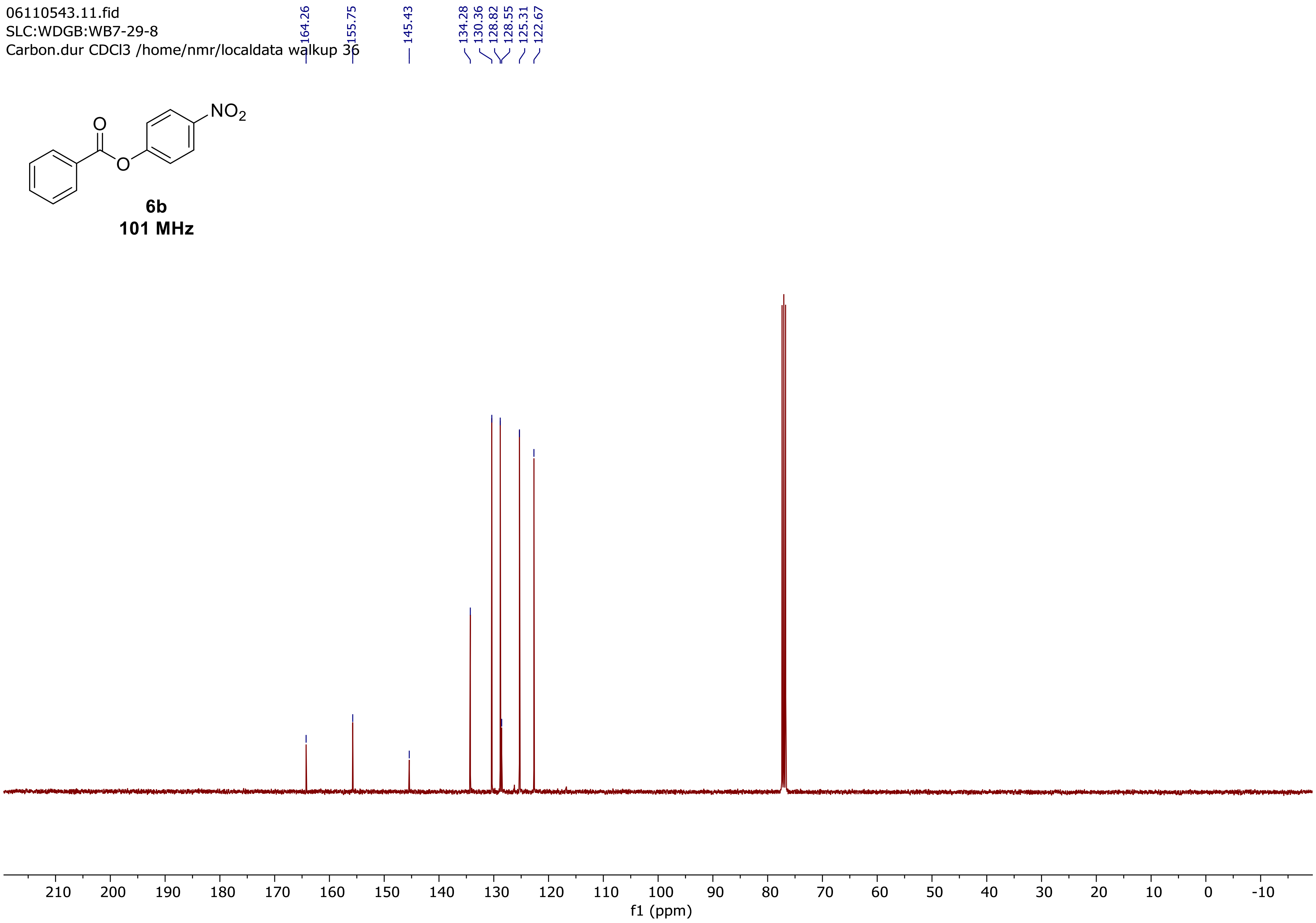
06110543.11.fid
SLC:WDGB:WB7-29-8
Carbon.dur CDCl3 /home/nmr/localdata walkup 36
164.26
155.75
145.43
134.28
130.36
128.82
128.55
125.31
122.67
NO2
O
O
6b
101 MHz
210
200
190
180
170
160
150
140
130
120
110
100
90
80
70
60
50
40
30
20
10
0
-10
f1 (ppm)

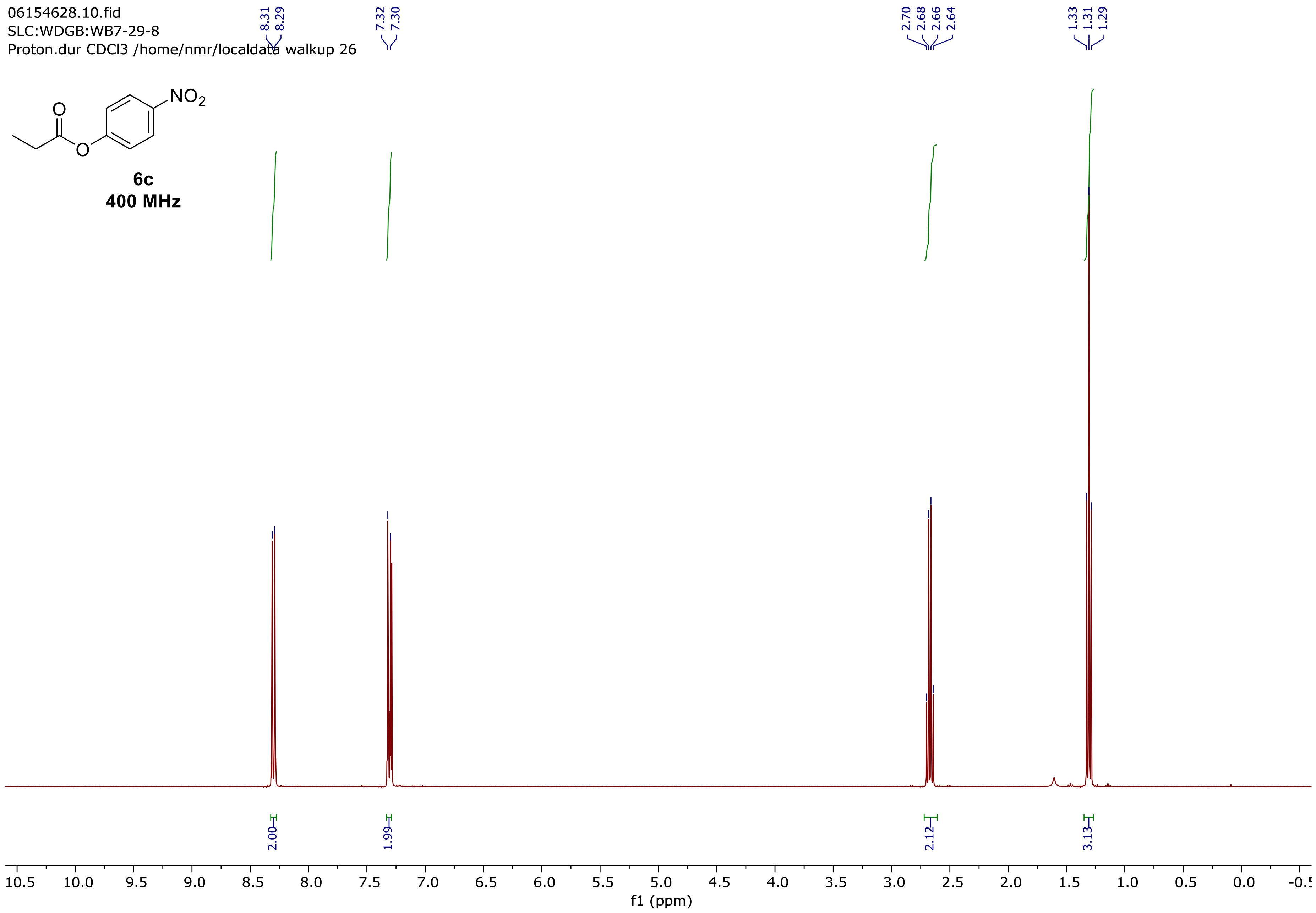
06154628.10.fid
SLC:WDGB:WB7-29-8
Proton.dur CDCl3 /home/nmr/localdata walkup 26
NO2
O
O
6c
400 MHz
8.31
8.29
7.32
7.30
2.70
2.68
2.66
2.64
1.33
1.31
1.29
2.00
1.99
2.12
3.13
10.5
10.0
9.5
9.0
8.5
8.0
7.5
7.0
6.5
6.0
5.5
5.0
4.5
4.0
3.5
3.0
2.5
2.0
1.5
1.0
0.5
0.0
f1 (ppm)

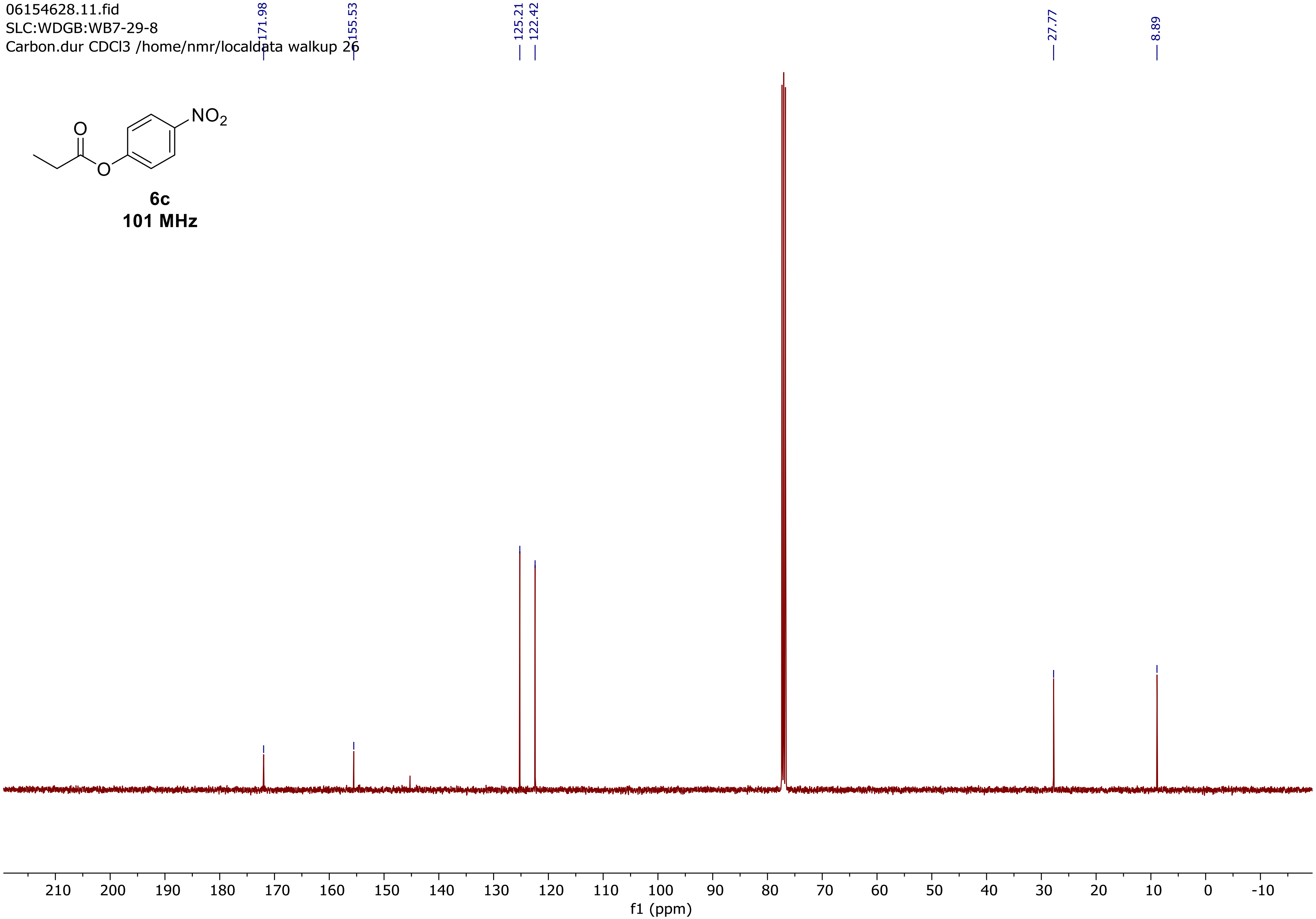
06154628.11.fid
SLC:WDGB:WB7-29-8
Carbon.dur CDCl3 /home/nmr/localdata walkup 26
171.98
155.53
125.21
122.42
27.77
8.89
NO2
O
O
6c
101 MHz
210 200 190 180 170 160 150 140 130 120 110 100 90 80 70 60 50 40 30 20 10 0 -10
f1 (ppm)

06154707.10.fid
SLC:WDGB:WB7-29-7
Proton.dur CDCl3 /home/nmr/localdata walkup 28

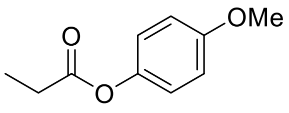

6d
400 MHz

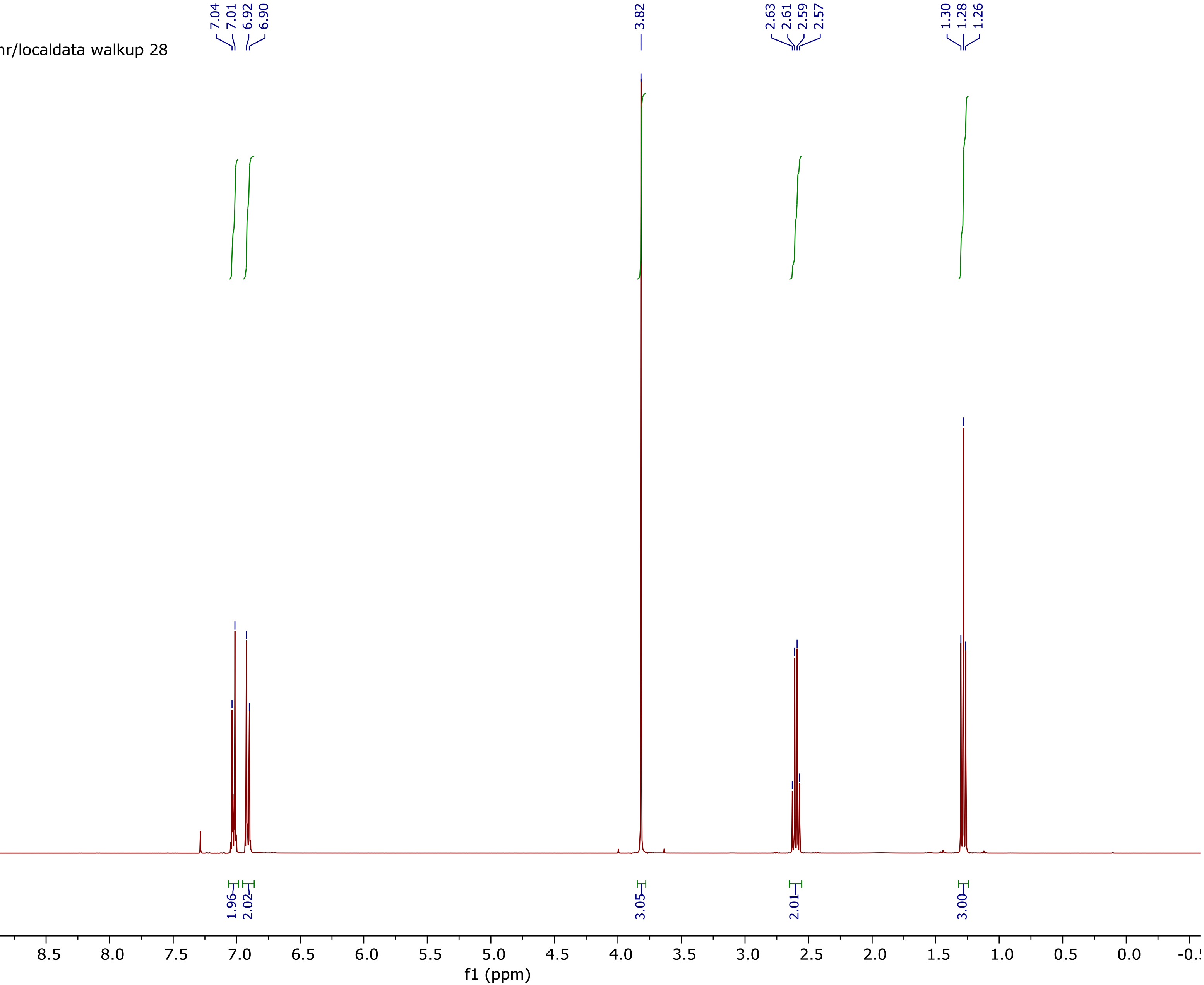

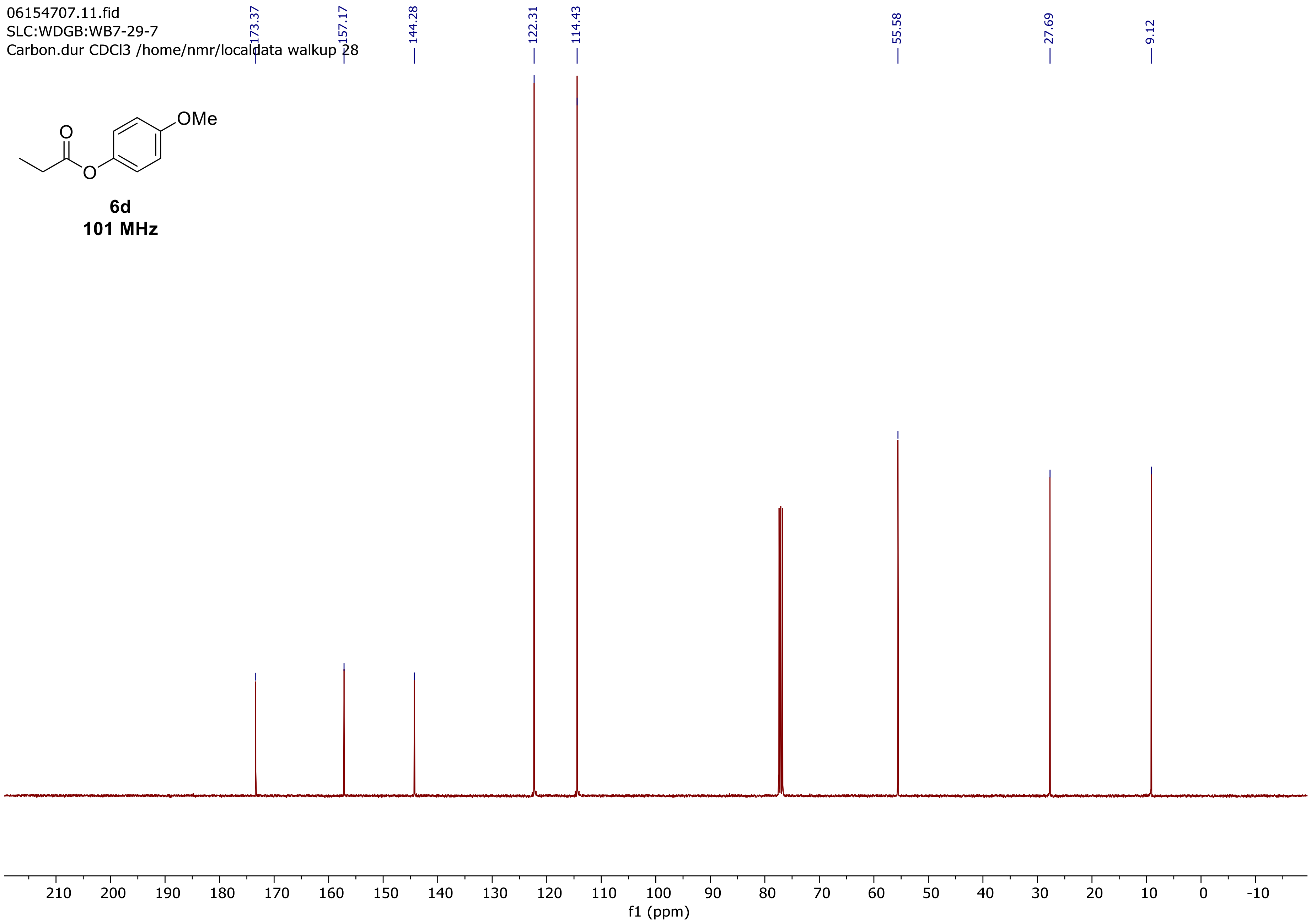
06154707.11.fid
SLC:WDGB:WB7-29-7
Carbon.dur CDCl3 /home/nmr/localdata walkup 28
173.37
157.17
144.28
122.31
114.43
55.58
27.69
9.12
OMe
O
O
6d
101 MHz
210 200 190 180 170 160 150 140 130 120 110 100 90 80 70 60 50 40 30 20 10 0 -10
f1 (ppm)

NMR Study of Acyl Fluoride Formation

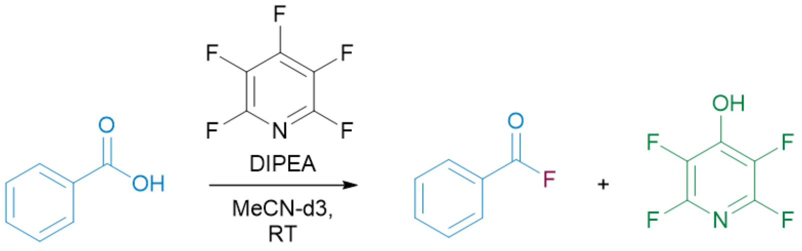

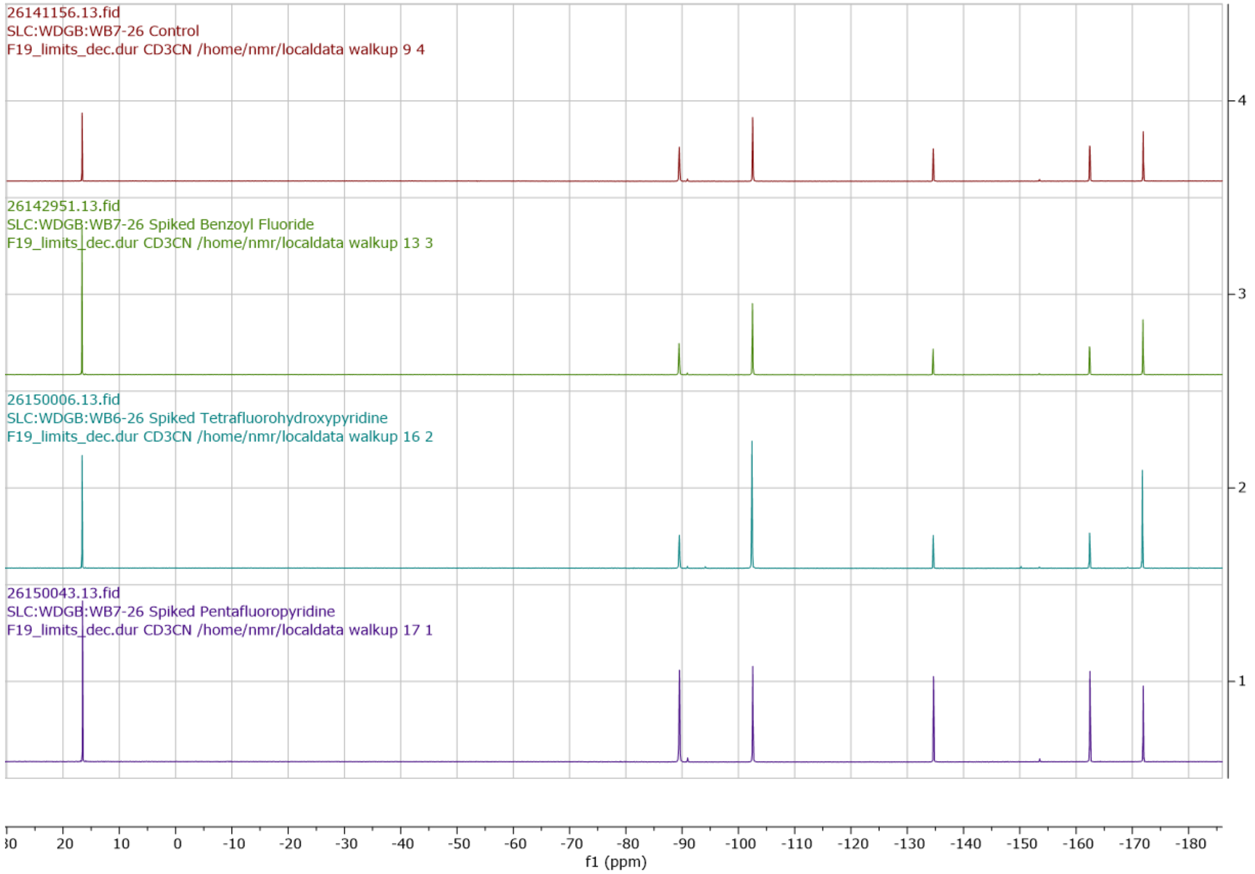

After activation period – no amine

After activation period – spiked with benzoyl fluoride

After activation period – spiked with tetrafluorohydroxypyridine

After activation period – spiked with pentafluoropyridine

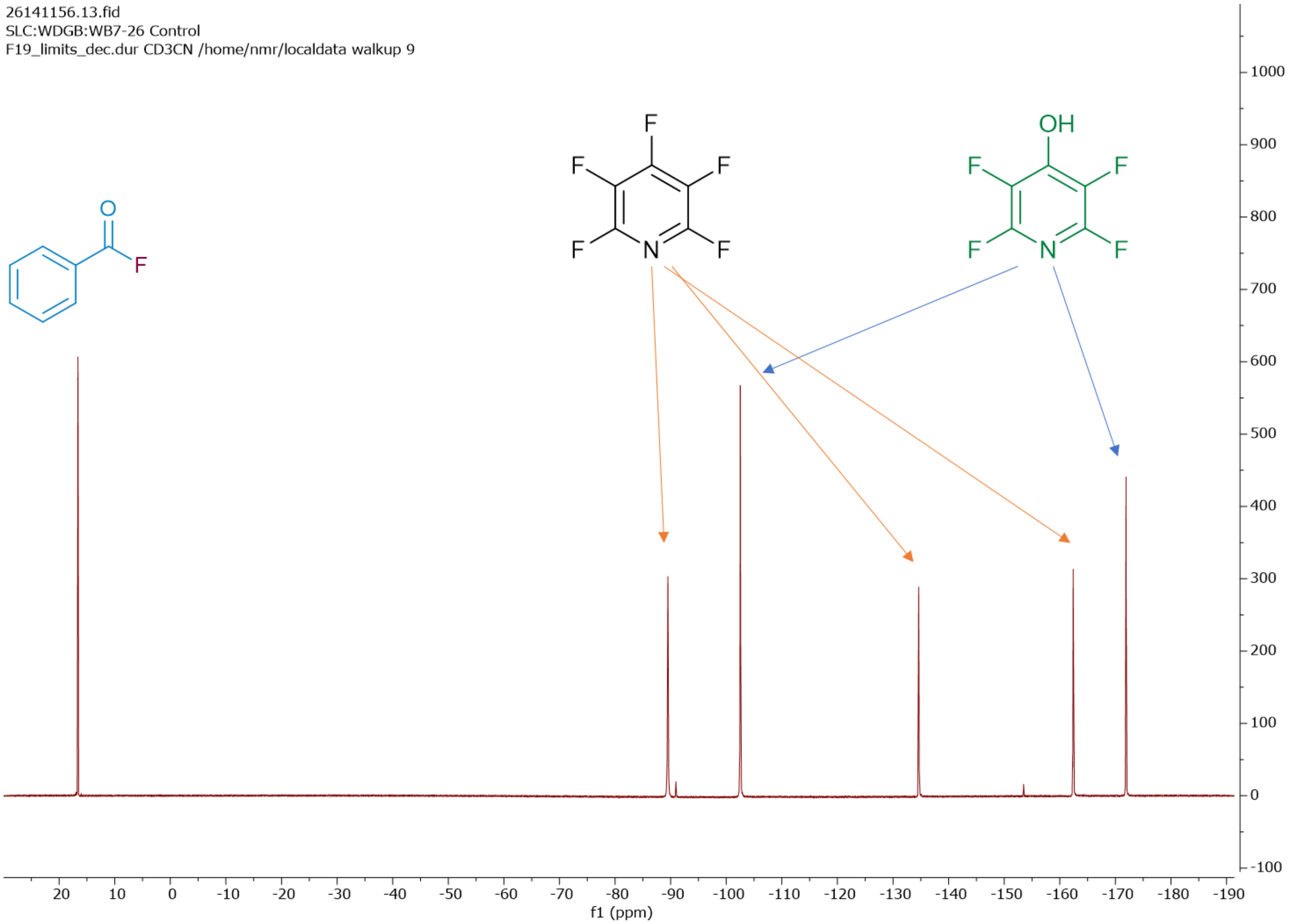
26141156.13.fid
SLC:WDGB:WB7-26 Control
F19_limits_dec.dur CD3CN /home/nmr/localdata walkup 9
O
F
F
F
F
F
F
N
OH
F
F
F
F
N
1000
900
800
700
600
500
400
300
200
100
0
-100
20
10
0
-10
-20
-30
-40
-50
-60
-70
-80
-90
-100
-110
-120
-130
-140
-150
-160
-170
-180
-190
f1 (ppm)

LCMS of Crude Reaction Mixture

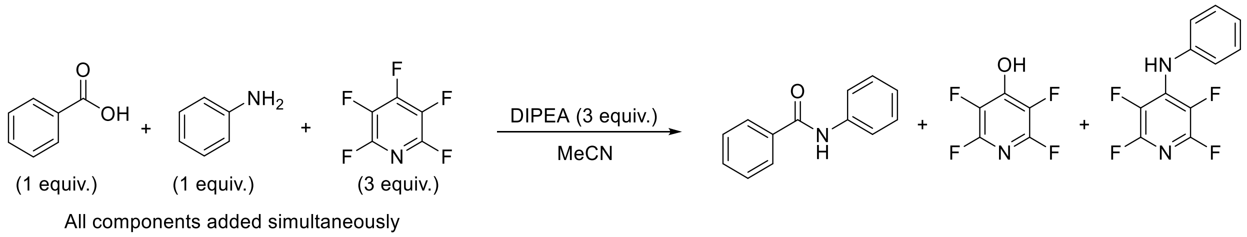

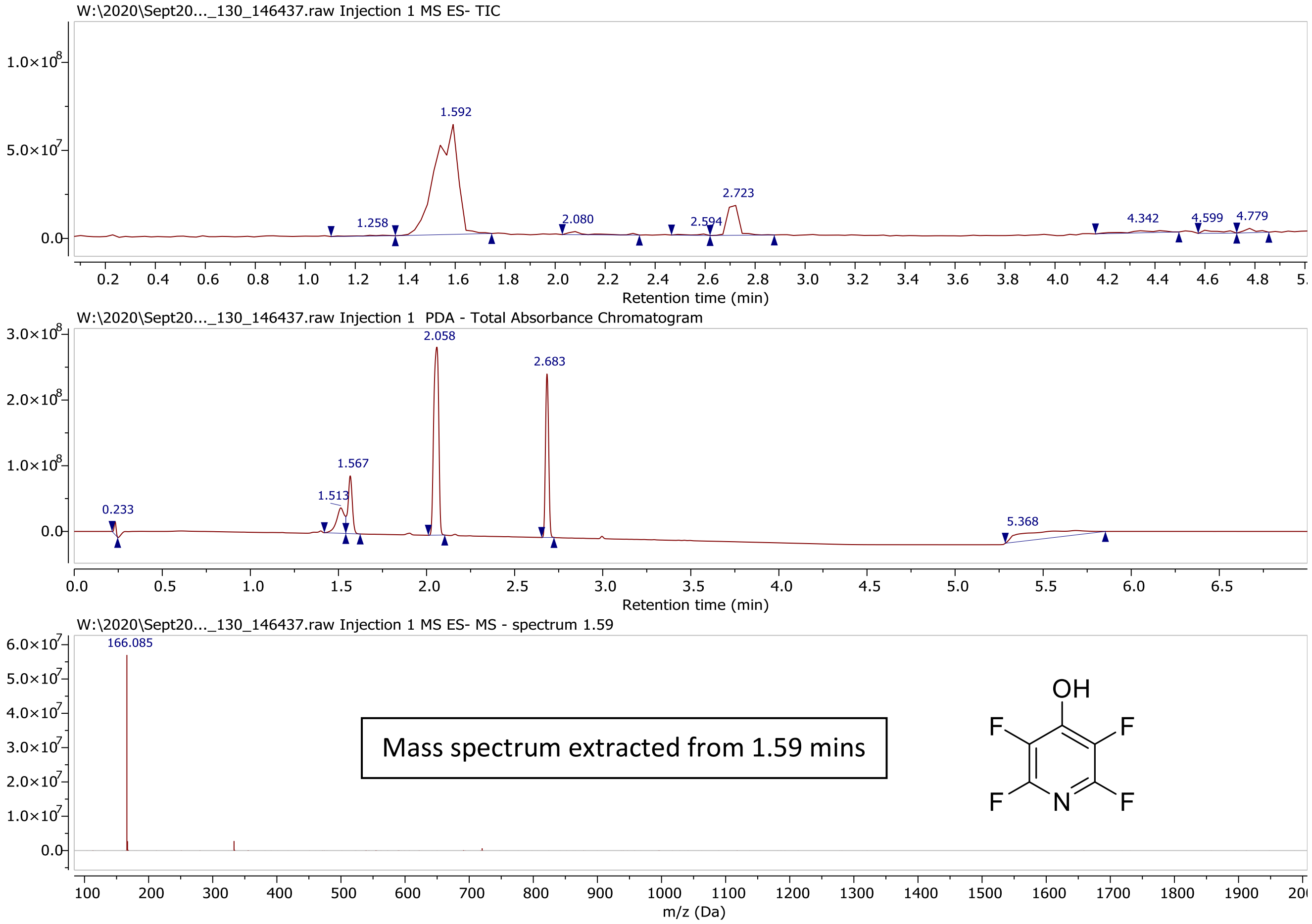

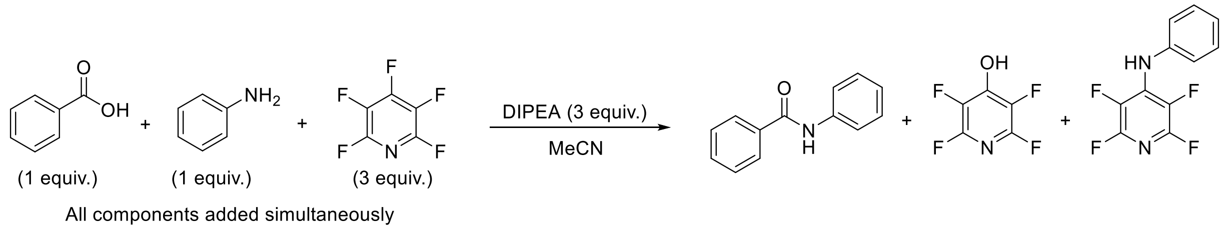
DIPEA (3 equiv.)
MeCN
(1 equiv.)
(1 equiv.)
(3 equiv.)
All components added simultaneously

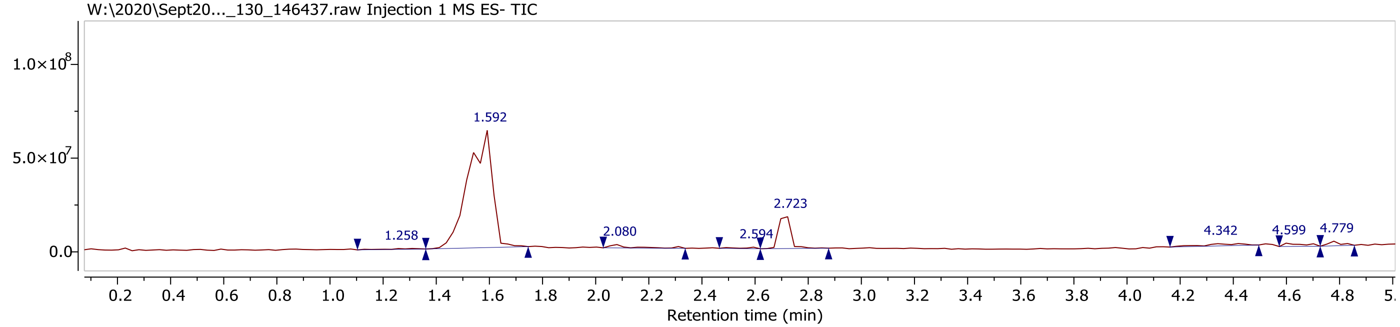
W:\2020\Sept20..._130_146437.raw Injection 1 MS ES- TIC
1.592
1.258
2.080
2.594
2.723
4.342
4.599
4.779
Retention time (min)

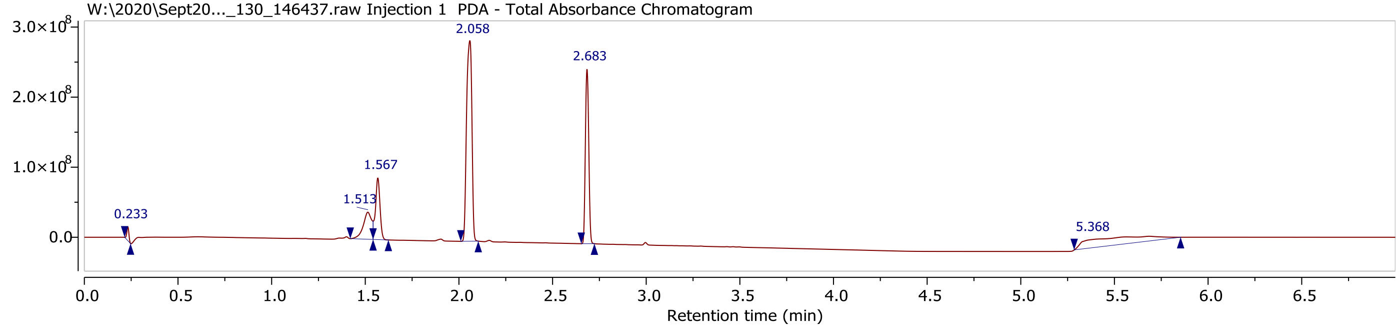
W:\2020\Sept20..._130_146437.raw Injection 1 PDA - Total Absorbance Chromatogram
2.058
2.683
1.567
1.513
0.233
5.368
Retention time (min)

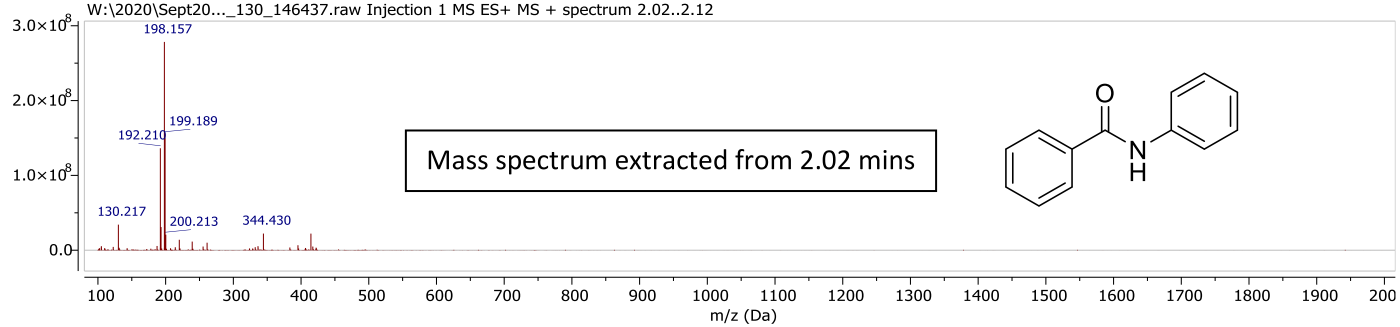
W:\2020\Sept20..._130_146437.raw Injection 1 MS ES+ MS + spectrum 2.02..2.12
198.157
199.189
192.210
130.217
200.213
344.430
Mass spectrum extracted from 2.02 mins
m/z (Da)

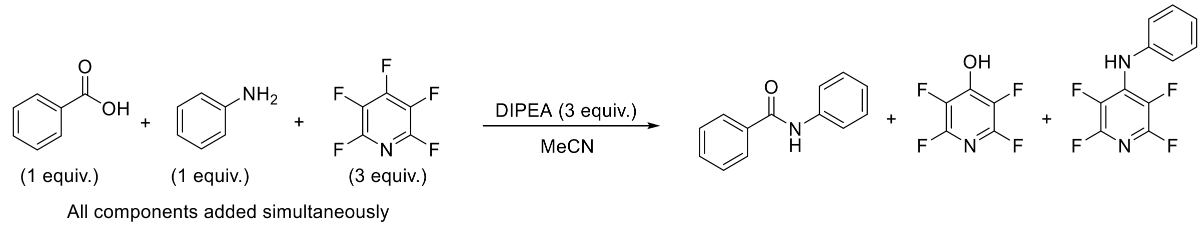

O
OH
NH2
F
N
(1 equiv.)
(1 equiv.)
(3 equiv.)
All components added simultaneously
DIPEA (3 equiv.)
MeCN
H
OH
HN

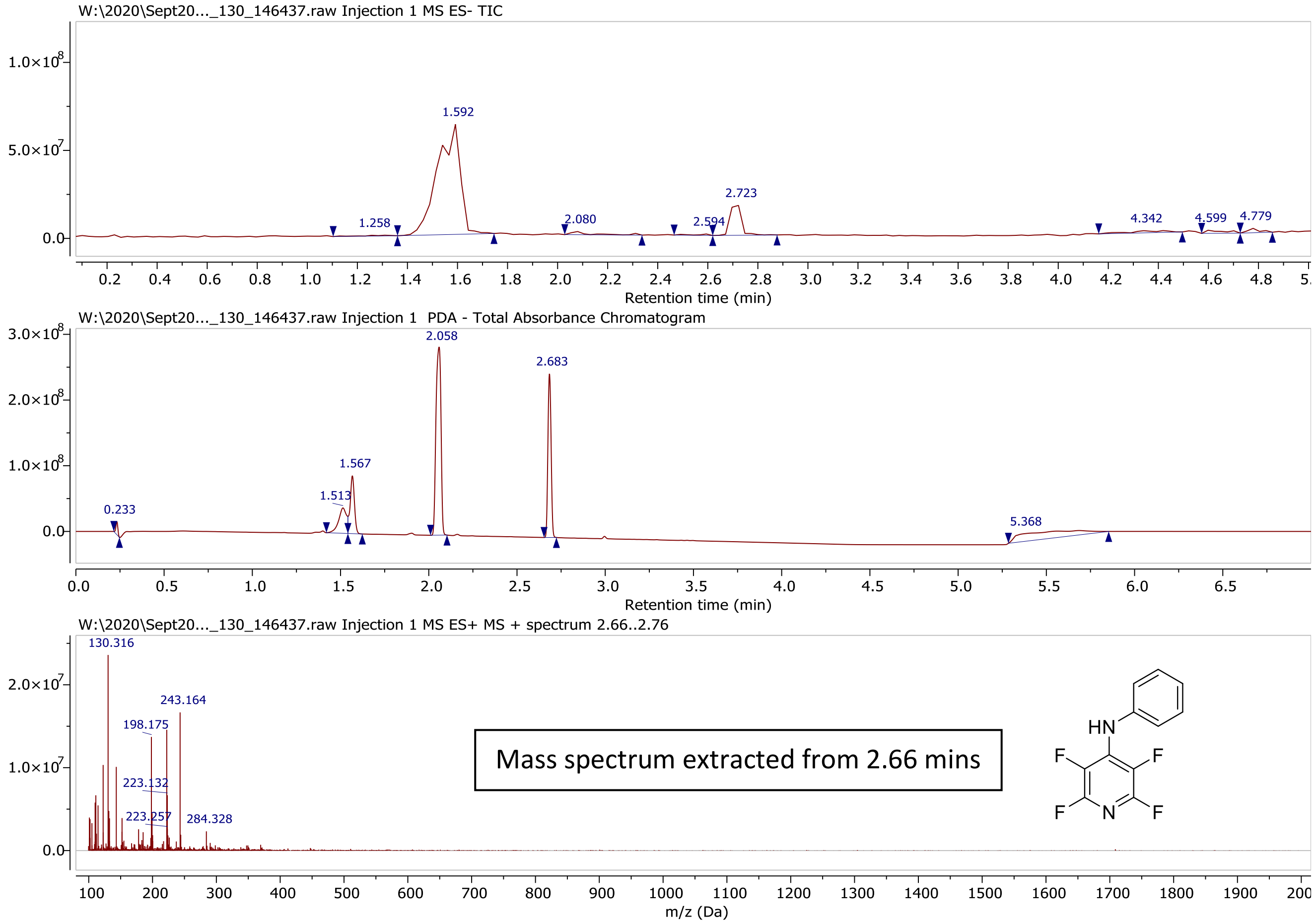

W:\2020\Sept20..._130_146437.raw Injection 1 MS ES- TIC
1.592
1.258
2.080
2.594
2.723
4.342
4.599
4.779
Retention time (min)
W:\2020\Sept20..._130_146437.raw Injection 1 PDA - Total Absorbance Chromatogram
2.058
2.683
1.567
1.513
0.233
5.368
Retention time (min)
W:\2020\Sept20..._130_146437.raw Injection 1 MS ES+ MS + spectrum 2.66..2.76
130.316
243.164
198.175
223.132
223.257
284.328
Mass spectrum extracted from 2.66 mins
m/z (Da)

4-Nitroaniline Sealed Tube Reaction Crude NMRs

13110345.10.fid
SLC:WDB:WB7-11
Proton.dur CDCl3 /home/nmr/localdata walkup 7

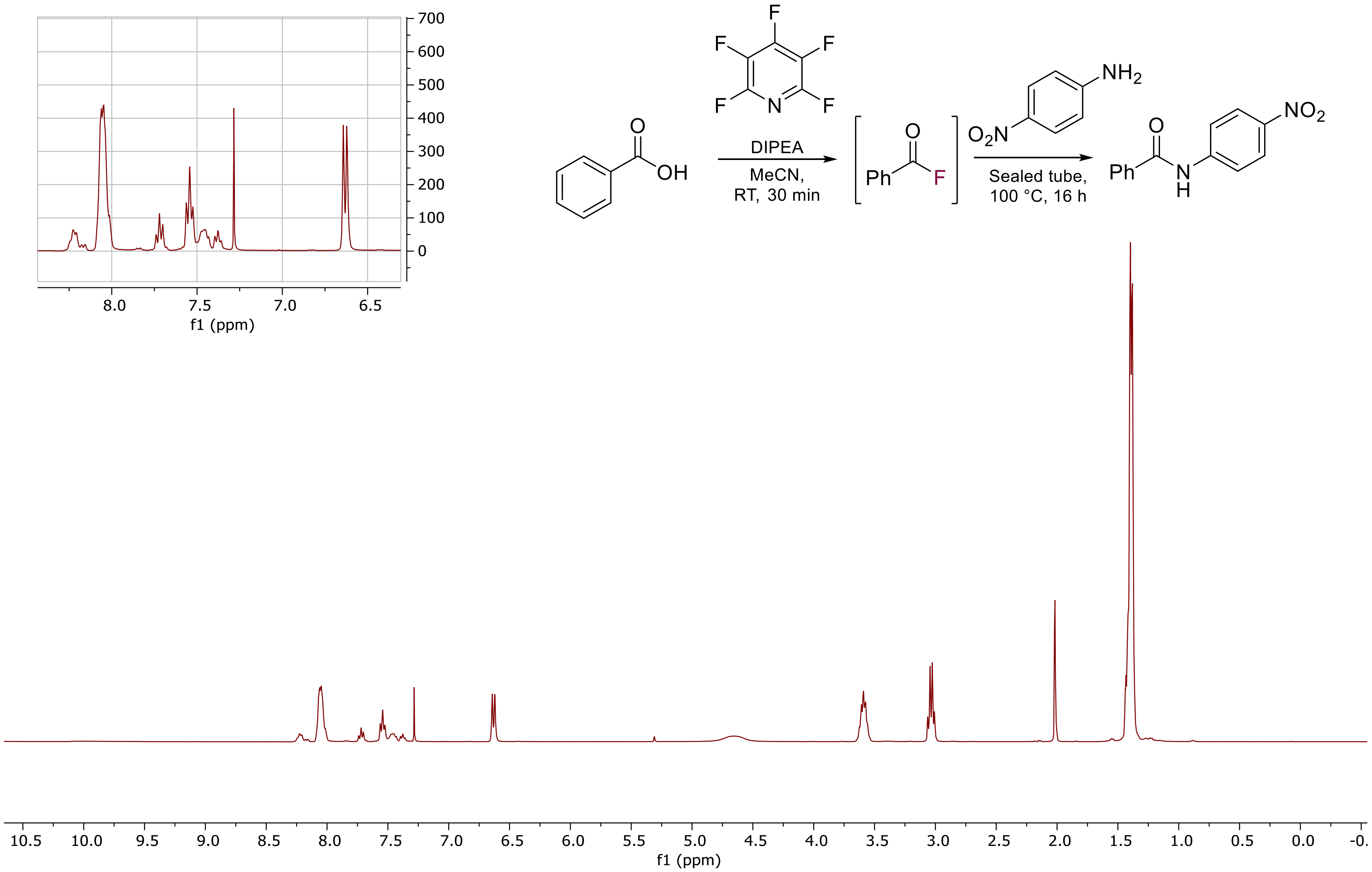

13110345.13.fid
SLC:WDB:WB7-11
F19_limits_dec.dur CDCl3 /home/nmr/localdata walkup 7

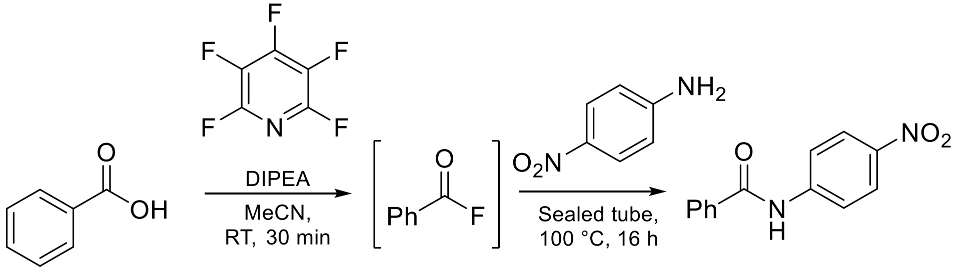

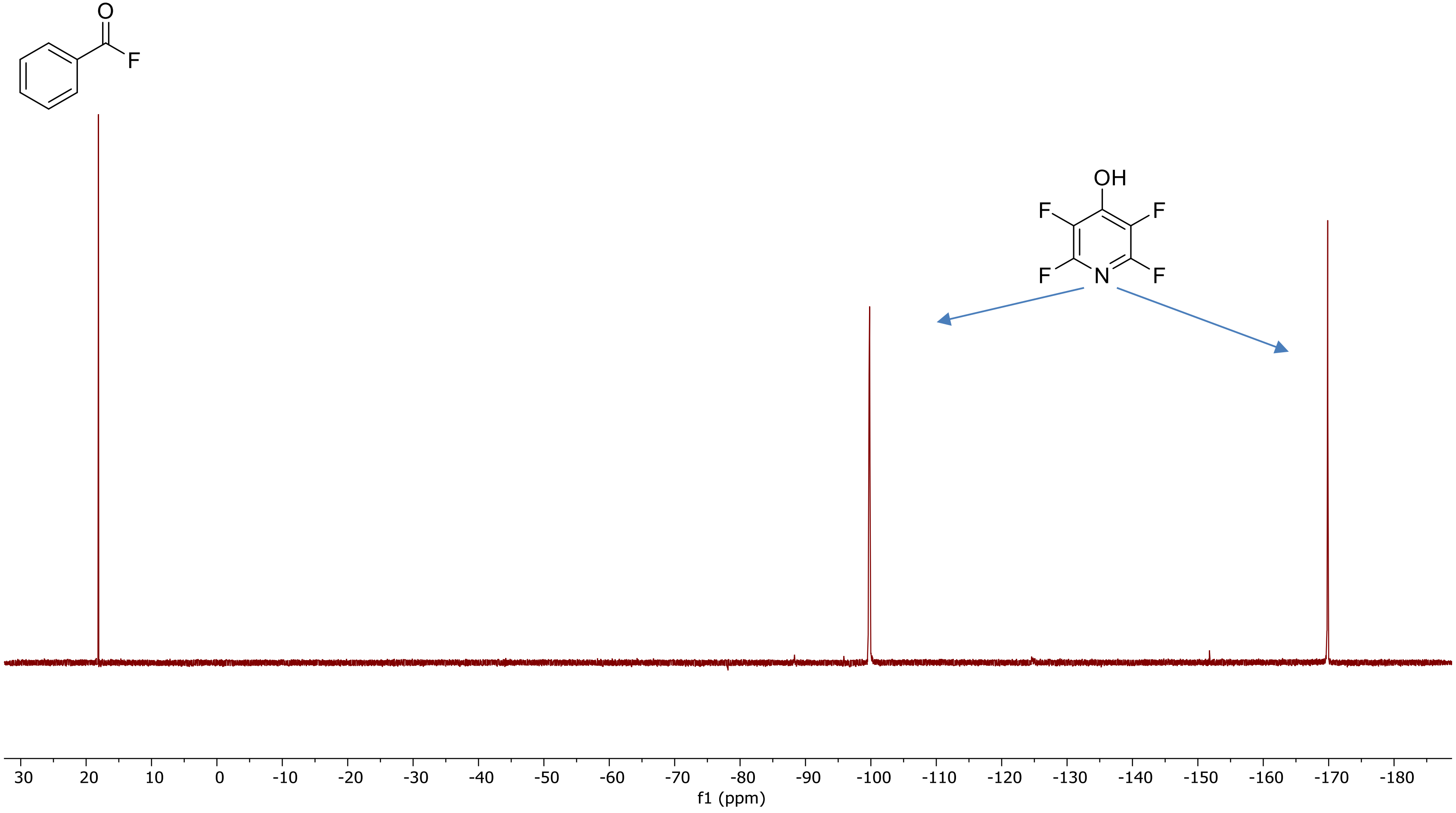

References

[1] P. J. Foth, T. C. Malig, H. Yu, T. G. Bolduc, J. E. Hein, G. M. Sammis, *Org. Lett.* **2020**, *22*, 6682-6686.

[2] S. J. Ryan, S. D. Schimler, D. C. Bland, M. S. Sanford, *Org. Lett.* **2015**, *17*, 1866-1869.

[3] S. B. Munoz, H. Dang, X. Ispizua-Rodriguez, T. Mathew, G. K. S. Prakash, *Org. Lett.* **2019**, *21*, 1659-1663.

[4] B. K. Zambron, S. R. Dubbaka, D. Markovic, E. Moreno-Clavijo, P. Vogel, *Org. Lett.* **2013**, *15*, 2550-2553.

[5] Y. Rao, X. Li, S. J. Danishefsky, *J. Am. Chem. Soc.* **2009**, *131*, 12924-12926.

[6] Y. C. Yuan, R. Kamaraj, C. Bruneau, T. Labasque, T. Roisnel, R. Gramage-Doria, *Org. Lett.* **2017**, *19*, 6404-6407.

[7] T. X. Metro, J. Bonnamour, T. Reidon, A. Duprez, J. Sarpoulet, J. Martinez, F. Lamaty, *Chemistry* **2015**, *21*, 12787-12796.

[8] C. L. Magyar, T. J. Wall, S. B. Davies, M. V. Campbell, H. A. Barna, S. R. Smith, C. J. Savich, R. A. Mosey, *Org Biomol Chem* **2019**, *17*, 7995-8000.

[9] G. Meng, P. Lei, M. Szostak, *Org. Lett.* **2017**, *19*, 2158-2161.

[10] S. M. Wang, C. Zhao, X. Zhang, H. L. Qin, *Org Biomol Chem* **2019**, *17*, 4087-4101.

[11] G. P. Yang, K. Li, W. Liu, K. Zeng, Y. F. Liu, *Org Biomol Chem* **2020**, *18*, 6958-6964.

[12] E. D. Funder, J. B. Trads, K. V. Gothelf, *Org Biomol Chem* **2015**, *13*, 185-198.

[13] J. Yin, J. Zhang, C. Cai, G. J. Deng, H. Gong, *Org. Lett.* **2019**, *21*, 387-392.

[14] X. Zhang, Z. Liu, Y. Gao, F. Li, Y. Tian, C. Li, X. Jia, J. Li, *Adv. Synth. Catal.* **2018**, *360*, 272-277.

[15] A. Sinai, D. Vangel, T. Gati, P. Bombicz, Z. Novak, *Org. Lett.* **2015**, *17*, 4136-4139.

[16] S. Scapecchi, C. Martelli, C. Ghelardini, L. Guandalini, E. Martini, F. Gualtieri, *Il Farmaco* **2003**, *58*, 715-722.

[17] E. C. L. Marrs, L. Varadi, A. F. Bedernjak, K. M. Day, M. Gray, A. L. Jones, S. P. Cummings, R. J. Anderson, J. D. Perry, *Molecules* **2020**, *25*.

[18] H. Fu, P. Li, Z. Wang, X. Li, Q. Dai, C. Hu, *Org Biomol Chem* **2020**, *18*, 4439-4446.

[19] W. Song, K. Dong, M. Li, *Org. Lett.* **2020**, *22*, 371-375.

[20] C. Xu, J. Xu, *Org Biomol Chem* **2020**, *18*, 127-134.

[21] W. Yu, S. Yang, F. Xiong, T. Fan, Y. Feng, Y. Huang, J. Fu, T. Wang, *Org Biomol Chem* **2018**, *16*, 3099-3103.

[22] S. C. Ghosh, J. S. Ngiam, A. M. Seayad, D. T. Tuan, C. L. Chai, A. Chen, *J. Org. Chem.* **2012**, *77*, 8007-8015.

[23] A. Dondoni, L. Kniezo, A. Medici, *J. Org. Chem* **2002**, *47*, 3994-3998.

[24] M. M. Wang, G. H. Sui, X. C. Cui, H. Wang, J. P. Qu, Y. B. Kang, *J. Org. Chem.* **2019**, *84*, 8267-8274.

[25] J. M. White, A. R. Tunoori, B. J. Turunen, G. I. Georg, *J. Org. Chem.* **2004**, *69*, 2573-2576.

[26] T. Hama, D. A. Culkin, J. F. Hartwig, *J. Am. Chem. Soc.* **2006**, *128*, 4976-4985.

[27] J. B. Peng, H. Q. Geng, D. Li, X. Qi, J. Ying, X. F. Wu, *Org. Lett.* **2018**, *20*, 4988-4993.

[28] S. Roy, M. J. Sarma, B. Kashyap, P. Phukan, *Chem Commun* **2016**, *52*, 1170-1173.

[29] B. Cheng, J. Wei, B. Zu, J. Zhao, T. Wang, X. Duan, R. Wang, Y. Li, H. Zhai, *J. Org. Chem.* **2017**, *82*, 9410-9417.

[30] F. Ma, X. Xie, L. Zhang, Z. Peng, L. Ding, L. Fu, Z. Zhang, *J. Org. Chem.* **2012**, *77*, 5279-5285.

[31] Y. Li, F. Jia, Z. Li, *Chemistry* **2013**, *19*, 82-86.

[32] Y. Tu, L. Yuan, T. Wang, C. Wang, J. Ke, J. Zhao, *J. Org. Chem.* **2017**, *82*, 4970-4976.

[33] G. Siano, S. Crespi, M. Mella, S. M. Bonesi, *J. Org. Chem.* **2019**, *84*, 4338-4352.

[34] F. Luo, C. Pan, J. Cheng, F. Chen, *Tetrahedron* **2011**, *67*, 5878-5882.

[35] H. Kitano, H. Ito, K. Itami, *Org. Lett.* **2018**, *20*, 2428-2432.
